# Assessing the degradation of ancient milk proteins through site-specific deamidation patterns

**DOI:** 10.1038/s41598-021-87125-x

**Published:** 2021-04-08

**Authors:** Abigail Ramsøe, Mia Crispin, Meaghan Mackie, Krista McGrath, Roman Fischer, Beatrice Demarchi, Matthew J. Collins, Jessica Hendy, Camilla Speller

**Affiliations:** 1 BioArCh, Department of Archaeology, University of York, York, UK; 2 Department of Earth Sciences, Natural History Museum, London, UK; 3 The GLOBE Institute, University of Copenhagen, Copenhagen, Denmark; 4 The Novo Nordisk Foundation Center for Protein Research, University of Copenhagen, Copenhagen, Denmark; 5 Department of Prehistory and Institute of Environmental Science and Technology (ICTA), Universitat Autònoma de Barcelona, Bellaterra, Spain; 6 Target Discovery Institute, Nuffield Department of Medicine, University of Oxford, Oxford, UK; 7 Department of Life Sciences and Systems Biology, University of Turin, Turin, Italy; 8 McDonald Institute for Archaeological Research, University of Cambridge, Cambridge, UK; 9 Department of Anthropology, University of British Columbia, Vancouver, Canada

**Keywords:** Proteins, Proteome informatics, Environmental social sciences

## Abstract

The origins, prevalence and nature of dairying have been long debated by archaeologists. Within the last decade, new advances in high-resolution mass spectrometry have allowed for the direct detection of milk proteins from archaeological remains, including ceramic residues, dental calculus, and preserved dairy products. Proteins recovered from archaeological remains are susceptible to post-excavation and laboratory contamination, a particular concern for ancient dairying studies as milk proteins such as beta-lactoglobulin (BLG) and caseins are potential laboratory contaminants. Here, we examine how site-specific rates of deamidation (i.e., deamidation occurring in specific positions in the protein chain) can be used to elucidate patterns of peptide degradation, and authenticate ancient milk proteins. First, we characterize site-specific deamidation patterns in modern milk products and experimental samples, confirming that deamidation occurs primarily at low half-time sites. We then compare this to previously published palaeoproteomic data from six studies reporting ancient milk peptides. We confirm that site-specific deamidation rates, on average, are more advanced in BLG  recovered from ancient dental calculus and pottery residues. Nevertheless, deamidation rates displayed a high degree of variability, making it challenging to authenticate samples with relatively few milk peptides. We demonstrate that site-specific deamidation is a useful tool for identifying modern contamination but highlight the need for multiple lines of evidence to authenticate ancient protein data.

## Introduction

The adoption of animal milk into the human diet has long intrigued archaeologists as it represents a vital source of nutrition for past and contemporary populations. It is unsurprising, therefore, that bioarchaeologists have sought out new molecular tools for identifying trace residues of dairy products preserved on or within material culture or skeletal remains. Whilst much of the previous work on the biomolecular detection of dairying has focused on the identification of milk fats^1–6^, the detection of ancient milk proteins is a growing approach^7–11^. Although the survival of food proteins in archaeological artefacts and human remains is less well-studied than their lipid counterparts, proteins can often be more taxonomically specific, enabling the identification of specific animal taxa utilized and consumed—a ‘zooarchaeology by proxy’ approach. Ancient milk proteins have been detected from a suite of archaeological artefacts, human remains, and objects of cultural heritage, including well-preserved whole cheese-like remains from Early Bronze Age China^12^, as well as from residues adhering to archaeological artefacts^13–15^. Milk proteins have also been extracted from ancient dental calculus in Europe^7,8,10,11^, Northeastern Africa^16^, and Central Asia^9^, providing direct evidence of dairy consumption as opposed to the evidence of dairy processing which is obtained from ceramic residues^15^ or well-preserved remains^12^. Recently, dog-specific milk proteins were detected in the bones of a neonate dog, indicating the power of this methodology for detecting milk consumption from skeletal remains alone^17^. Milk proteins have also been detected in ancient mortars, binders and paints, e.g.,^18^ providing valuable insight into past manufacturing and informing contemporary conservation practices.

Archaeological artefacts are susceptible to contamination from modern (and ancient) biological materials as well as laboratory reagents^19,20^. The challenge of contamination has been explored thoroughly in ancient DNA studies, where contamination from modern sources of DNA or PCR-product carry-over has been well documented^21^. To date, the question of contamination in ancient protein sequences has received less attention; nevertheless, its potential is high and can occur at any point from excavation and curation through to extraction and injection into the liquid chromatography column in LC–MS/MS analysis^22^. For example, post-excavation handling or conservation treatments with plasters or glues that contain milk, or laboratory contamination by milk powders (e.g., blotting agents) can easily introduce a modern milk proteome to ancient artefacts or skeletal remains. Additionally, genuine archaeological milk proteins are often detected at low abundance, which makes it difficult to differentiate them from sporadic post-excavation contamination.

Because proteins degrade over time, damage identified in recovered proteins has been used to argue for or against their authenticity^23^. Recently, the software tool deamiDATE^24^ has been developed in order to assess patterns of asparagine and glutamine deamidation in proteins in relation to the expected rate of the deamidation reaction^25^. The rates calculated by Robinson and Robinson^26^ and used by deamiDATE refer to short artificial peptides under controlled pH and temperature conditions (37 °C, pH 7.4). We note that these conditions are not those typically experienced by archaeological specimens, but they do provide a baseline for calculations. DeamiDATE is based on the idea that high levels of deamidation in peptides that are expected to take a long time to deamidate under these standard conditions should only be seen in genuinely old proteins; whereas deamidation events that have a theoretical fast rate are less reliable as a measure of authentication. Here, we use deamiDATE to investigate to what extent asparagine and glutamine deamidation rates can be used as a measure of authenticity for ancient milk proteins. First, we apply deamiDATE to estimate deamidation rates in two modern experimental datasets: laboratory milk powder and archaeological artefacts that have been intentionally exposed to potential laboratory contamination, in order to identify deamidation signatures of possible contaminating sources. Next, we compare these rates to those displayed by ancient proteomic data, specifically 274 samples from previously published studies reporting the detection of milk proteins (caseins, beta-lactoglobulin (BLG)) in archaeological dental calculus^7–9,11,27^ and ceramic artefacts^15^. Our analysis reveals that deamidation rates display a high degree of variability, making it challenging to authenticate samples with relatively few milk peptides, although in general site-specific deamidation rates are more advanced in BLG recovered from ancient dental calculus and pottery residues.

## Materials and methods

### Experimental datasets

Two experimental milk datasets were used to characterize deamidation rates in potential contamination sources. Firstly, proteins from skim milk powder were extracted and analysed with the aim of understanding the deamidation patterns in milk-based laboratory reagents. We then undertook an experiment that exposed archaeological samples to potential laboratory contamination to examine if their patterns of deamidation differed meaningfully from those of putative endogenous ancient milk proteins.

### Skim milk powder

We extracted and analyzed proteins from Sigma Aldrich skim milk powder [Cat. no. 70166] to: a) investigate the damage caused by the evaporation processes and b) to quantify deamidation patterns of milk powder that is used as a lab reagent, in order to estimate the likelihood of detecting it as a contaminant in archaeological samples.

The protein extraction of skim milk powder was based on the protocol published by Jersie-Christensen et al.^28^ for ancient dental calculus analysis, without the demineralisation step. Approximately 500 µg of skim milk powder was suspended in guanidine buffer (2 M guanidine hydrochloride solution, 20 mM chloroacetamide, 10 mM tris (2-carboxyethyl)phosphine) with 100 mM Tris) and ammonium hydroxide was used to adjust the pH to 7.5–8.5. The sample was heated for 10 min at 99 °C to denature the proteins, and then subsequently cooled for 10 min. The sample was then digested at 37 °C for 1 h with 0.2 μg of rLysC (Promega) under agitation. The sample was then diluted to a final concentration of 0.6 M guanidine hydrochloride using 25 mM Tris in 10% acetonitrile (ACN). The sample was then digested overnight with 0.8 μg of trypsin (Promega). To inactivate the trypsin 10% trifluoroacetic acid (TFA) was added until the pH was < 2. The peptides were washed and collected on in-house made C18 StageTips and stored in the freezer until mass spectrometry analysis. The sample was eluted with 20 μL of 40% ACN followed by 10 μL of 60% ACN directly into a 96 well plate, and subsequently evaporated in a SpeedVac Concentrator (Thermo Fisher Scientific) until ~ 3 μL was left and 5 μL of 0.1% TFA, 5% ACN was added.

The sample was then analysed using an EASY-nLC 1200 system connected to a Q-Exactive HF (Thermo Scientific) mass spectrometer at the Novo Nordisk Center for Protein Research, University of Copenhagen, according to previously published parameters^29^.

### Contamination experiment

We undertook an experiment to identify potential milk contamination that can result from exposure of archaeological samples within a modern laboratory setting. We selected five archaeological samples for shotgun proteomic analysis, including three dental calculus samples; one ostrich (*Struthio kakesiensis*) eggshell and one limescale deposit from an historic sewer pipe (Table 1). These samples were selected since: (1) they represent a range of archaeological or paleontological substrates previously demonstrated to preserve ancient proteins; and (2) based on their origin and provenance, the majority are unlikely to preserve endogenous milk proteins. The dental calculus samples were recovered from mid-nineteenth century contexts in Rupert’s Valley, St. Helena^30^. Previous proteomic analyses of dental calculus from the same archeological context (and extracted within a dedicated ancient proteins laboratory) did not produce any evidence of milk proteins^7^. The ostrich eggshell was recovered from the Laetoli site in Tanzania and dates to ~ 3.85 to 4.3 mya^31^; as above the eggshell, previously analyzed in Demarchi et al.^32^, displayed no evidence of milk proteins or analogues. The fifth sample represented a limescale deposit on an early nineteenth century water pipe from Hungate, a ‘slum’ part of York^33^, where milk products are unlikely to have been present in large quantities.

**Table 1:** Contamination experiment sample extracted and analyzed in this study.

Sample code	Weight (mg)	Archaeological site [identifier]	Age
Dental Calculus A	50	Rupert’s Valley, St Helena. SK358 (8786)	Mid-nineteenth century
Dental Calculus B	81.9	Rupert’s Valley, St Helena. SK520 (8836)	Mid-nineteenth century
Dental Calculus C	39.8	Rupert’s Valley, St Helena. SK245 (8753)	Mid-nineteenth century
Limescale	102.1	Hungate, York. Subsampled from S Bend drain	Early-nineteenth century
Egg Shell	89.8	Lot 13898, Kakesio 1–6, Lower Laetoli Beds, Tanzania	~ 3.85 to 4.3 mya
Blank	N/A	Extraction blank control	

The samples were weighed out as whole pieces and were crushed into powder in a 2.0 ml Eppendorf tube using a sterile micropestle for each sample. 1800 μL of ethylenediaminetetraacetic acid (EDTA) 0.5 M pH 8.0 was added to each sample. The samples were then parafilmed and incubated at room temperature on a rotator for 5 days. Protein extraction was undertaken at the Discovery Proteomics Facility at the Target Discovery Institute (TDI) in Oxford, within their protein extraction laboratory. The laboratory follows standard procedures for molecular biology laboratories: protective equipment was worn (i.e., gloves, lab coats) but other contamination controls recommended for degraded samples were omitted (i.e., a separate space for the analysis of archaeological samples, aerosol resistant pipette tips, or extraction hoods were not used).

Samples were vortexed for 30 s and then centrifuged for 5 min; proteins were extracted from the pellet and from 100 μL of supernatant using a gel-aided sample preparation (GASP) method^34^. To each sample, 100 μL of Pierce IP Lysis Buffer (Thermo Fisher Scientific; Catalogue Number 87787) was added, before the addition of 100 μL of acrylamide (30%). The samples were vortexed for 30 s and left for 5–10 min. 2 μL of TEMED (tetramethylethylenediamine) followed by 2 μL of 10% APS (ammonium persulfate) was added to samples, and left for 10–20 min until a gel had formed. Once set, the gel was shredded through a filter membrane and fixed through rotation with 10% acetic acid, 50% methanol, 40% water. The solution was centrifuged, and the supernatant discarded. 1 mL of ACN was added to dehydrate the gel pieces. A series of washing and drying steps using ACN were then performed to exchange buffers, following the method outlined in Hendy et al.^8^. Finally, the samples were digested overnight at 37 °C in 250 μL of ammonium bicarbonate (0.05 M) and 1 μg trypsin. The next morning, samples were centrifuged for 1 min. 250 μL of ACN was added and the samples were placed on a shaker for 5 min. The supernatant was removed and retained in a new tube, and 250 μL of 5% formic acid was added to the gel pieces for 5 min. The supernatant was removed and added to the first fraction, and 100 μL of ACN was added to the gel pieces, and shaken for 5 min. The supernatant was taken off and combined with the first and second fractions before being desalted using Millipore Zip-Tips prior to MS/MS analysis.

All samples were analysed using a Thermo Fisher Scientific n-LC Q-Exactive tandem mass spectrometer at the Discovery Proteomics Facility, Target Discovery Institute, Oxford according to previously published specifications^35^.

### Ancient milk

We re-analyzed six recently published datasets reporting proteomic evidence of ancient milk, including five dental calculus studies^7–9,11,27^ and one proteomic analysis of ceramic vessels^15^ (Table 2). Although a number of publications have recently reported the recovery of milk proteins from ancient samples^12–14,36–41^, few studies to date have made their raw data available for re-analysis through public databases. These six datasets were selected as they span a wide timescale and geographical area, and most importantly, raw data and sample identifiers were made publically available through ProteomeXchange. Where possible, ages were assigned to samples using published radiocarbon dates. In the absence of direct dating, age was assigned to be in the middle of the reported archaeological period or date ranges (see Supplementary Table S1).

**Table 2:** Archaeological datasets reanalysed in this study.

Dataset (accession)	Substrate	Number of samples	Age (range)	Site/s	Radiocarbon dates available?
Warinner et al.^35^ *(PXD001357, PXD001359, PXD001360, PXD001361, and PXD001362)*	Dental calculus	122	Neolithic—present day	UK, Denmark, Norway, Germany, Hungary, Italy, Switzerland, Armenia, Russia, St Helena, Greenland	No
^26^ *(MSV000081706)*	Dental calculus	12 samples from 3 individuals	Neolithic—Bronze Age	UK	Yes
^9^ *(PXD008217)*	Dental calculus	12 samples from 9 individuals	Bronze Age	Mongolia	Yes
^8^ *(PXD009603)*	Dental calculus	112	Iron Age—present day	UK (USA -modern samples only)	No
^8^ *(PXD008647)*	Ceramic vessels	18 samples from 10 vessels (33 including replicates)	Neolithic	Çatalhöyük, Turkey	Yes—indirectly from context
Charlton et al.^11^ *(PXD012893)*	Dental calculus	10	Neolithic	UK	Yes—indirectly from context

### Bioinformatic analysis

#### MaxQuant

Proteomic data analysis followed the same bioinformatic methods for all datasets. Raw files from all datasets were searched using MaxQuant 1.6.2.6a against a database of caseins (including alpha-S1-, alpha-S2-, beta-, and kappa-casein) and BLG from horse, goat, sheep, and cow, using a semi-tryptic search strategy (Supplementary File S1). The minimum score for modified and unmodified peptides was set to 60. For previously published archaeological datasets extracted using a filter-aided sample preparation method (FASP), the fixed modification was set to carbamidomethyl (C), and the variable modifications hydroxyproline, Glu and Gln to pyro-Glu, deamidation (NQ), acetyl (N-term) and oxidation (M). For the published datasets, and archaeological samples extracted with the GASP method, the fixed modification was propionamide (C), and the variable modifications included all those in the FASP setup, plus propionamide N-term and propionamide (K). As there is considerable overlap between the database used and MaxQuant’s contaminant database, the search for contaminants was turned off. All other parameters were set to MaxQuant’s defaults, including a false discovery rate (FDR) of 1%.

In order to allow for robust comparison between datasets, the experimental datasets (milk powder and the contaminated artefacts) were run using the same settings as the FASP-extracted archaeological data.

#### DeamiDATE

We then ran deamiDATE 1.0^24^ on the MaxQuant output files, which performs intensity-based deamidation calculations at the sample-protein level. Deamidation refers to the process in which asparagine and glutamine lose an amide group, and thereby are converted into aspartic acid and glutamic acid, respectively. Due to differences in the pathways involved, glutamine deamidates at a much slower rate than asparagine (with median half-times of 6100 and 60 days respectively), and as such, it has been proposed as a method for determining relative archaeological age^42–49^, though has also been suggested to be a proxy for preservation, rather than a dating tool^50^. In theory, the higher the extent of glutamine deamidation, the more degraded (i.e., “older”) the sample. DeamiDATE calculates the deamidation of proteins within a sample, including site-specific deamidation—i.e., differences in deamidation rate of glutamine and asparagine based on the presence of specific neighbouring amino acids^25^.

This site-specific approach is more nuanced than reporting the bulk levels of deamidation in a sample, as it can differentiate between rapid deamidation events (e.g., those that can occur within days of deposition, or during the protein extraction process—and therefore have low predictive power as to estimate the age or authenticity of a sample) and high half-time deamidation events (e.g., those that occur more slowly and should therefore only be observed in genuinely ancient proteins). In order to avoid spurious protein identifications, only proteins with a minimum of two supporting peptides were included in downstream analyses, as per Hendy et al.^22^. The evidence and peptide files from MaxQuant were used as input to the program, and peptides missing intensities were discarded. Output from MaxQuant and deamiDATE are included in Supplementary Files S2 and S3 respectively.

## Results

### Modern milk

#### Skim milk powder

**Figure 1:**
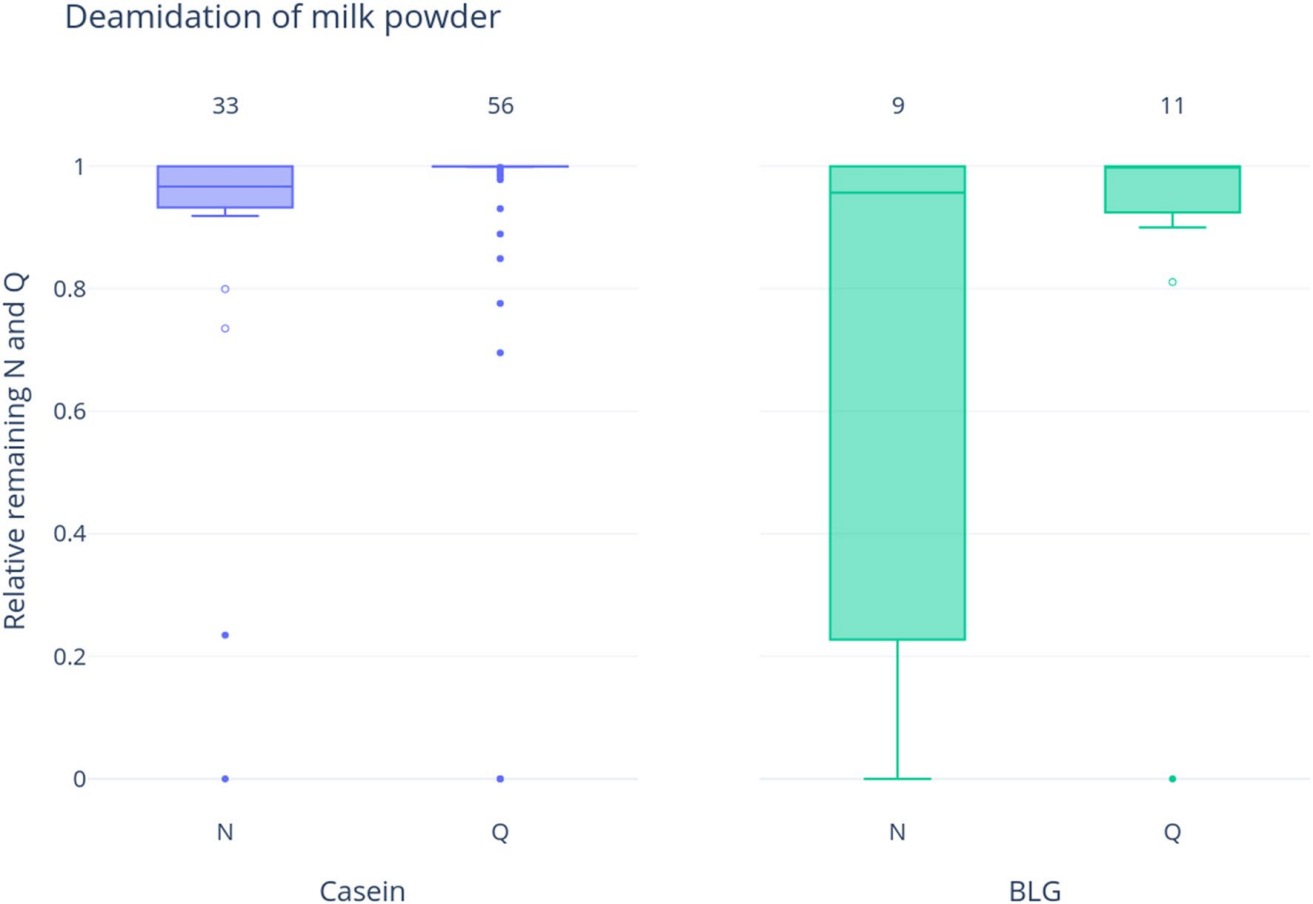
Deamidation of any casein and BLG proteins in milk powder samples. Numbers at the top of the bars show the number of deamidating amino acids (i.e., asparagine and glutamine combined) represented by this result. The y-axis represents the relative remaining amount of the deamidating amino acid—therefore high values represent less deamidation; whereas low values imply higher levels of deamidation. Outliers are shown as points, while suspected outliers are shown as hollow points.

As expected, proteins extracted from skim milk powder resulted in a large number of casein and BLG peptides (n = 295 and n = 72, respectively). As milk is subjected to extreme heat in the dehydration process, and heating has a denaturing effect on protein structure, we expected to find increased levels of deamidation. In contrast to our expectations, for both BLG and caseins, the median values display very low levels of bulk deamidation (above 95% undamaged peptides in all cases, Fig. 1, Supplementary Table S2). For both proteins, there is slightly more asparagine deamidation than glutamine, consistent with expectations of facile asparagine deamidation and with the difference in calculated activation energies (92–100 kJ/mol for Asn and 131 kJ/mol or 134 kJ/mol for either direct hydrolysis of cyclization-mediated glutamine deamidation). There is also greater variability in asparagine deamidation in BLG compared to caseins (excluding outliers).

**Figure 2:**
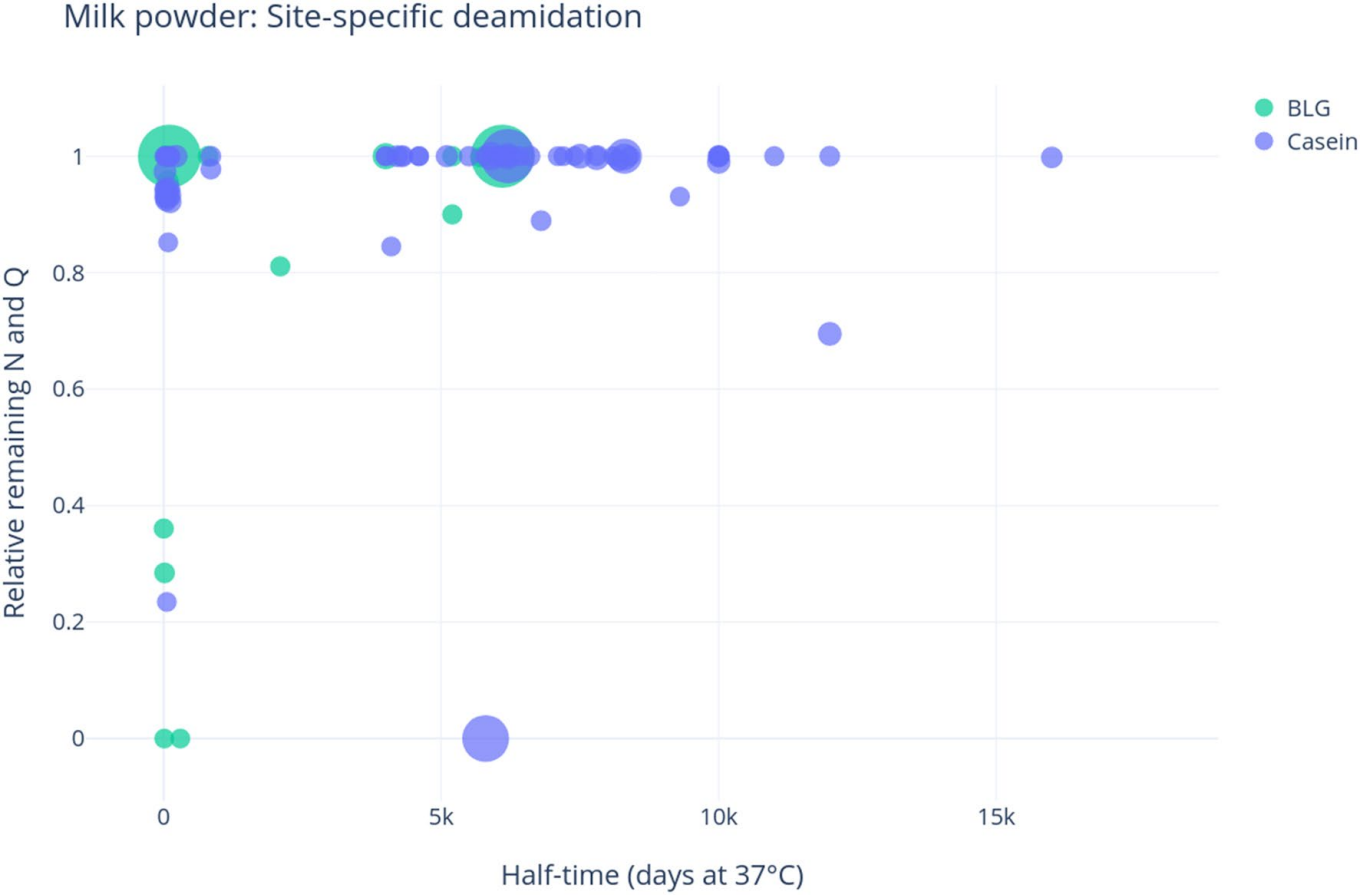
Site-specific deamidation of milk powder proteins. The y-axis shows the relative remaining portion of the deamidating amino acid, while the x-axis represents the half-time of the sites of deamidation. The size of the points is relative to the intensity of the peptide identified. Half-times as estimated by Robinson and Robinson^26^, plots produced through deamiDATE^24^.

We also examined patterns of site-specific deamidation in the skim milk powder (Fig. 2; Supplementary Table S2). BLG displays only high-levels of deamidation in low half-time sites, i.e., sites which take on average a few days (in the standard conditions used for the theoretical rate calculations) to deamidate. The casein results are very similar, although fewer deamidated low half-time sites were detected, and surprisingly shows some sites with very high half-time (above 5000 days) that are deamidated. This implies that caseins and BLG react differently to heat, and therefore also possibly to the diagenetic effects of time, consistent with Schroeter and Cleland’s^50^ suggestion that deamidation is a proxy for preservational quality. It is important to point out that temperature alters the overall order of occurrence of the various diagenetic reactions, so much so that high-temperature laboratory experiments are notoriously unable to mimic the combined effect of time and normal burial temperatures on protein diagenesis. The rapid exposure to extreme heat is therefore likely to produce effects that are significantly different from those observed in archaeological specimens.

### Contamination experiment

**Figure 3:**
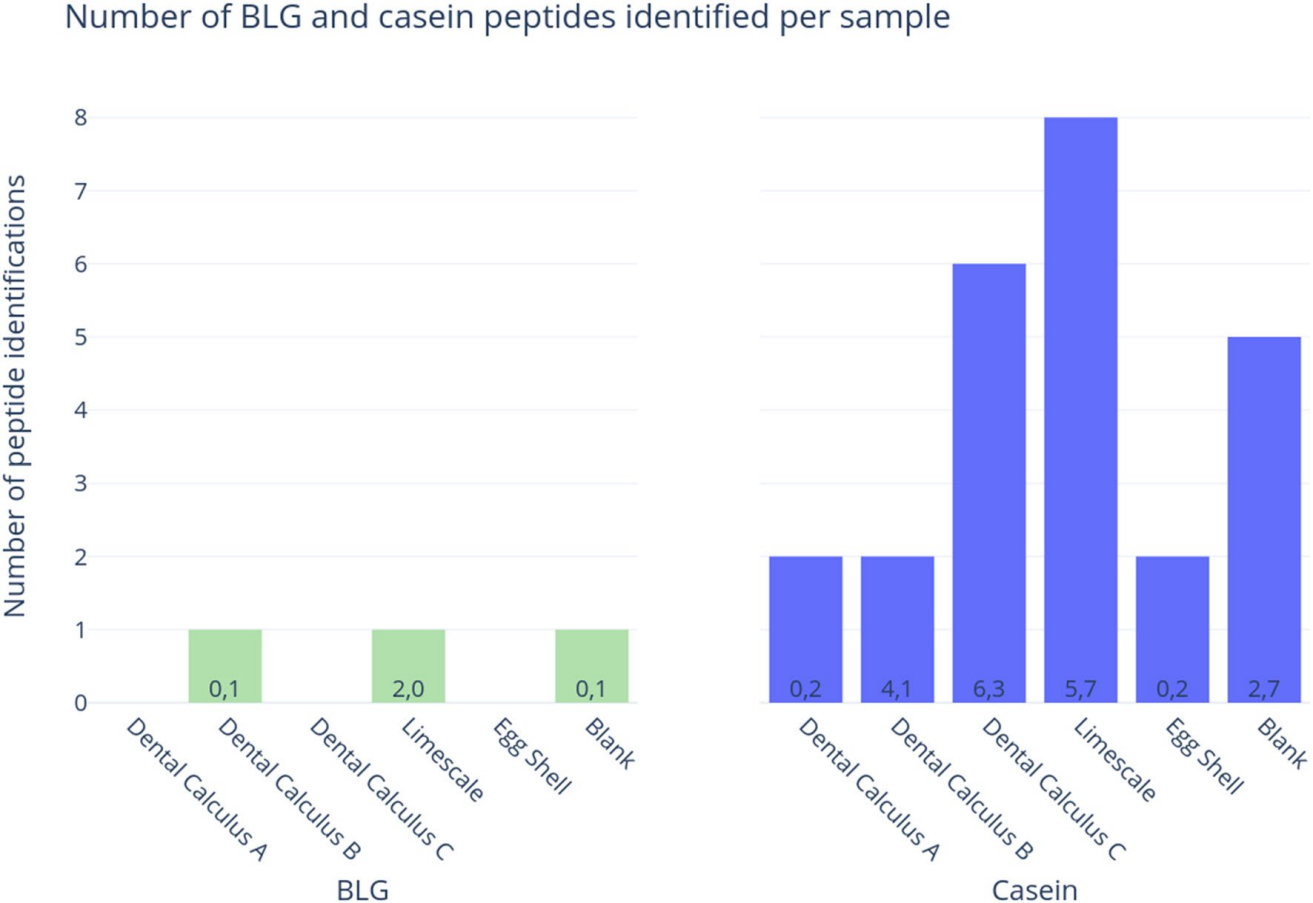
Number of BLG and any casein peptides identified in experimentally contaminated samples (extracted and analyzed in this study) according to MaxQuant’s ‘razor and unique peptides’ output. The number of asparagine and glutamine (respectively) residues in all peptides in each category is shown in the bars.

We extracted proteins from archaeological materials within a modern protein laboratory setting, first to determine whether traces of milk proteins from the laboratory environment could be detected through mass spectrometry, and second, to characterize deamidation in any contaminating milk peptides. Caseins were confidently detected (with two or more unique peptides) in all five samples in the contamination experiment, as well as the extraction blank control (five peptides) (Fig. 3). BLG was detected in two out of the five samples, as well as the extraction blank control, but only a single peptide was identified in each sample (each representing a different amino acid sequence) (Fig. 3).

**Figure 4:**
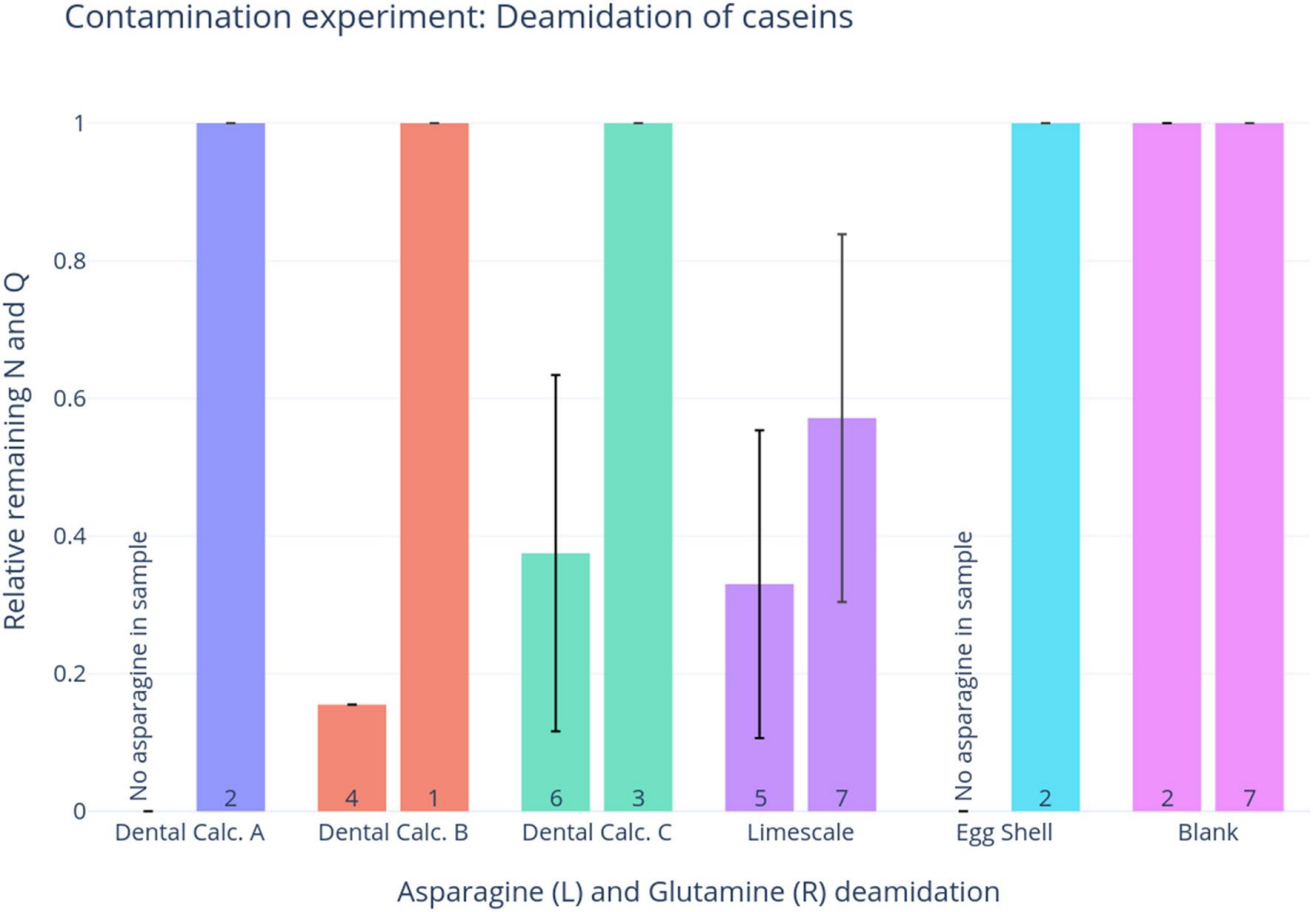
Bar graph of deamidation of asparagine (left) and glutamine (right) in contamination experiment samples. Error bars represent the standard deviation. The number of residues involved in this calculation is shown in each bar; plots produced through deamiDATE^24^.

Most of the experimental samples displayed no deamidation within the casein peptides (Fig. 4), which is consistent with expectations of contamination from modern laboratory reagents, such as skim milk powder (presented above). There is some asparagine deamidation in two of the dental calculus samples, though this could have been a result of laboratory-induced deamidation (Supplementary Table S3, Fig. 4, see discussion below). The limescale sample exhibits the highest levels of deamidation, with an average of 20% remaining asparagine and 57% remaining glutamine, though with a wide range of glutamine deamidation.

**Figure 5:**
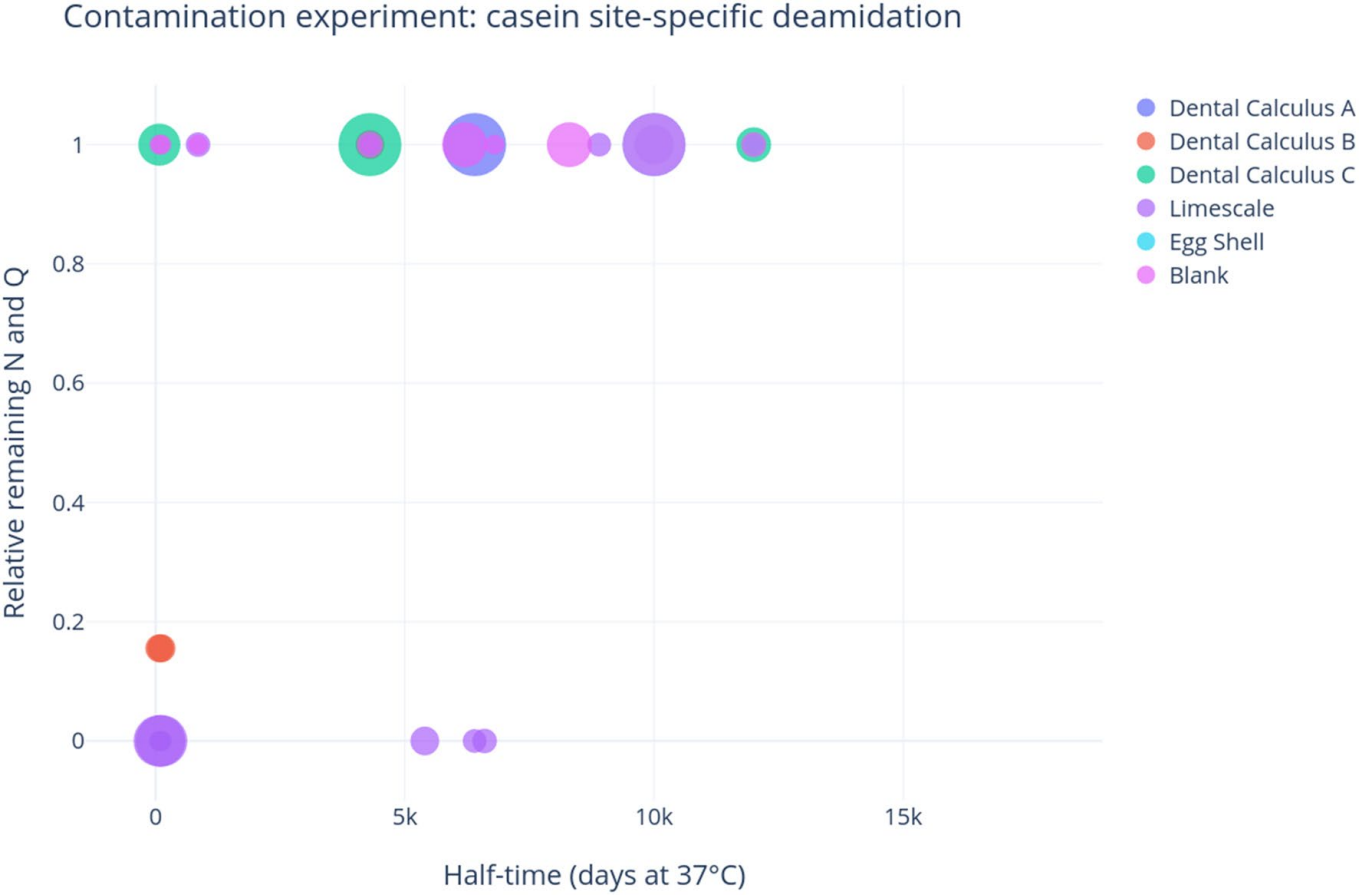
Site-specific deamidation in contamination experiment, where the y-axis represents the proportion of remaining deamidating amino acid, and the x-axis shows the half-time of the sites. The size of the points represents the intensity of the peptide; plots produced through deamiDATE^24^.

Figure 5 displays the site-specific deamidation of caseins within the contamination experiment, and indicates that the only casein deamidation events in the dental calculus samples are at very low half-times, so could likely be a result of sample preparation and processing, and are unlikely to be a signature of ancient milk. The limescale results are more nuanced. It shows some high half-time deamidation, which is theoretically indicative of genuine archaeological proteins. However, these peptides with high half-time deamidation have very low intensity (shown by the size of the scatter points), and there seem to be an equal amount of high half-time potential deamidation events in limescale that do not undergo deamidation, and these points have higher intensity. Lastly, the limescale casein peptides are either totally deamidated or totally intact (e.g., they are at 0 or 1)—there are no cases when the same peptide is found in both states, which would lead to a deamidation value of above 0 and below 1.

### Ancient milk

**Figure 6:**
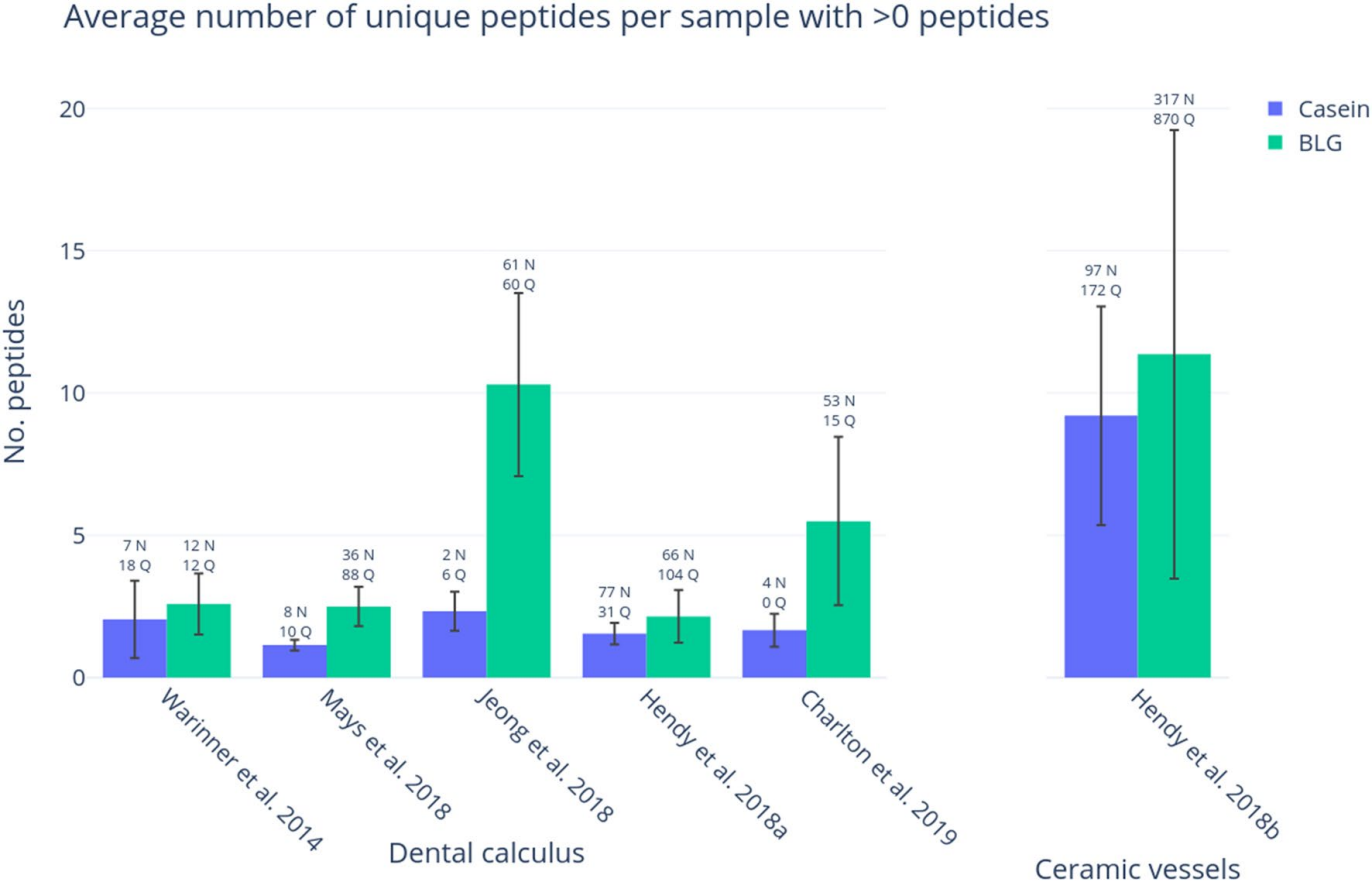
BLG and casein identifications in samples with at least one peptide in reanalysed datasets from previously published works. The average number of unique BLG and casein peptides is shown per sample in each study for samples that have at least one peptide. The error bars represent standard deviation. The summed total number of asparagine and glutamine residues attributed to any milk peptides for all sample in each study is shown above the error bars.

We characterized deamidation patterns in six previously published datasets with putative ancient milk proteins obtained from dental calculus and ceramic vessels. All datasets contained samples that had produced confident identifications of BLG and/or caseins (i.e., two or more unique peptides) in their original publications. As shown in Fig. 6, when data from all samples are grouped, more BLG peptides are detected compared to caseins. In general, caseins are detected relatively infrequently in ancient dental calculus; the only samples to produce abundant casein peptides were modern dental calculus samples (Hendy et al. 2018a^8^) (see Supplementary Table S4) and ancient ceramic pottery residues from Catalhöyük West (Hendy et al. 2018b^15^).

**Figure 7:**
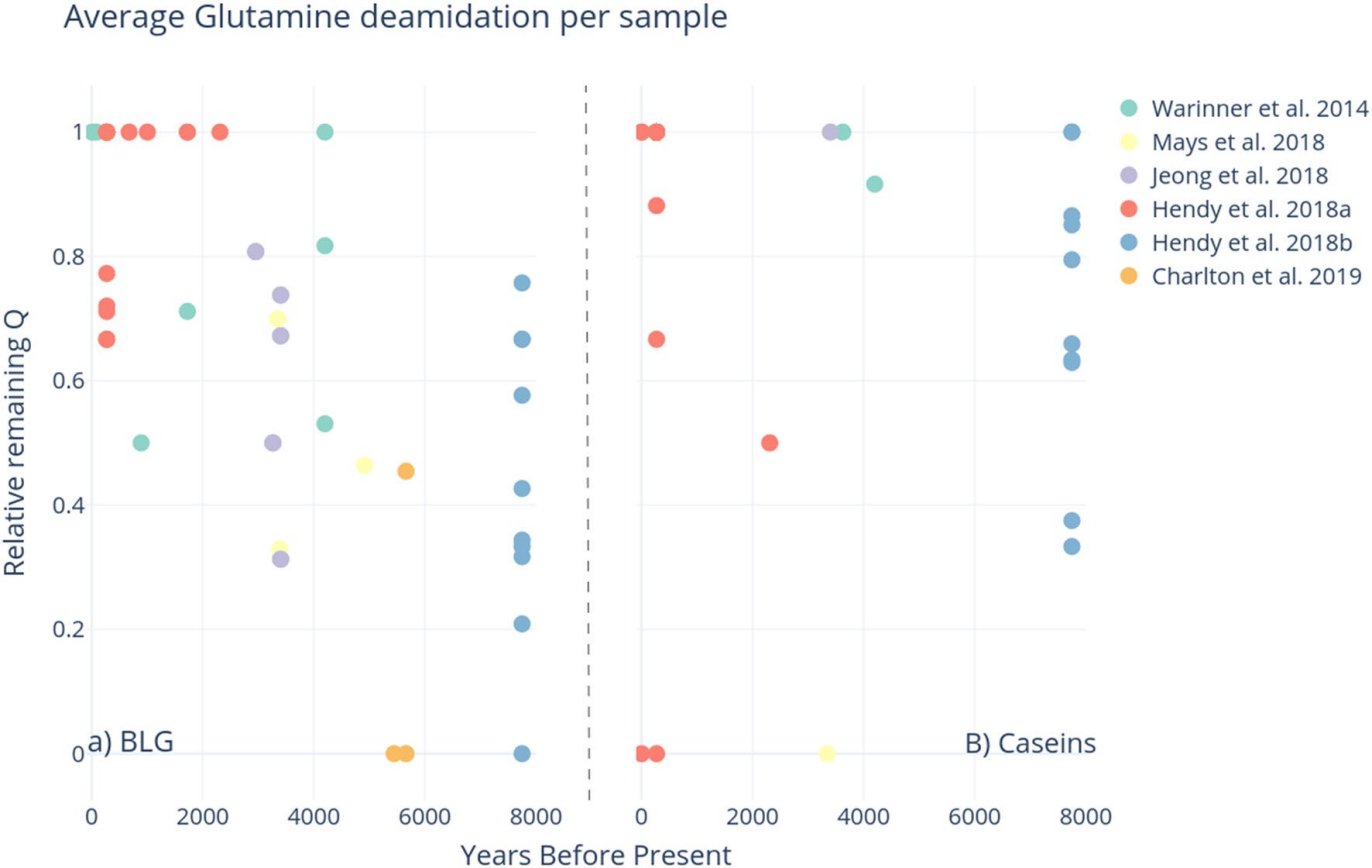
Average glutamine deamidation per sample, (**A**) BLG, (**B**) Caseins. The estimated age of the samples (years before present) is on the x-axis. Plots produced through deamiDATE^24^.

We characterized levels of glutamine deamidation in each sample, as shown in Fig. 7 (average asparagine deamidation is displayed in Supplementary Fig. S1). There is no clear pattern of increasing glutamine deamidation in samples of increasing age for neither BLG nor caseins, and variability is fairly high, consistent with Schroeter and Cleland’s^50^ findings on the variability of collagen deamidation. This variability could indicate a mix of modern and ancient origins of the recovered peptides or simply the difficulty in tracking the behaviour of multiple amino acids in complex protein structures embedded in widely different mineral matrices, undergoing degradation in different environments.

**Figure 8:**
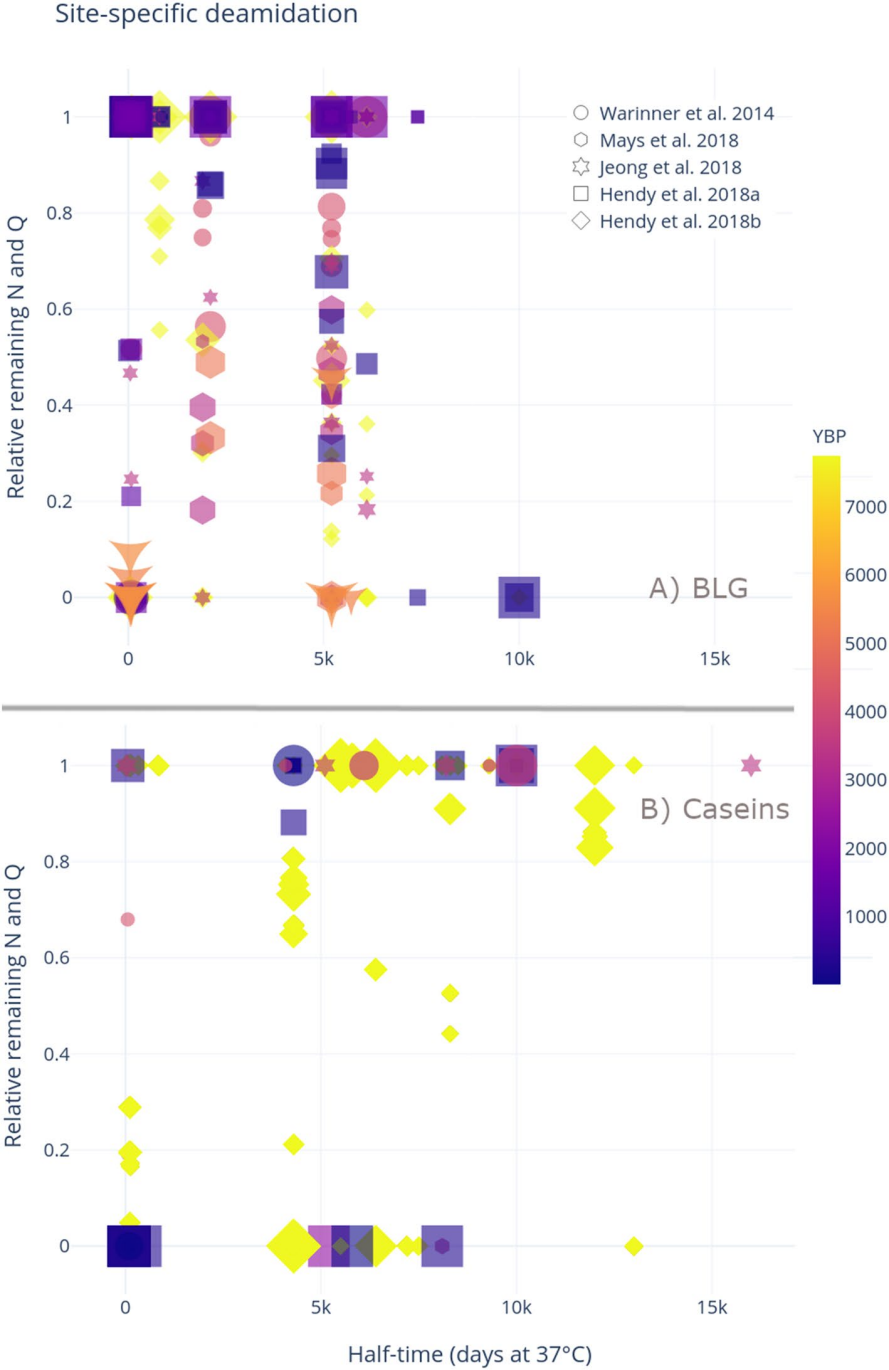
Site-specific deamidation of (**A**) BLG and (**B**) Caseins, where the points are coloured by the samples’ age, and the marker represents the paper from which the sample originated. Plots produced through deamiDATE^24^.

We characterized site-specific deamidation for both BLG and caseins in the ancient samples. We expected that high levels of high half-time deamidation would be detected in the most ancient samples—this is not the case, however. Figure 8a suggests that BLG deamidation varies greatly, and does not seem to correlate with the age of the sample. Indeed, at the highest half-times detected, three deamidating positions show complete deamidation, yet they are all in peptides retrieved from less than 1000 year old samples from Hendy et al. 2018^8^. This highlights how complex post-depositional histories can affect the reliability of this type of analysis if the data are taken at face value.

Deamidation of caseins, shown in Fig. 8b demonstrates even more variable patterns. Identified peptides across all samples seem to either be completely deamidated, or completely intact. The obvious exception to this is the ceramic vessel residues analysed by Hendy et al. 2018b^15^, which are the only samples to consistently display more intermediate levels of deamidation (discussed below).

## Discussion

This study sought to quantify the degradation of milk proteins in archaeological data, reanalysing 274 archaeological samples from six studies; along with two experimental datasets. The main aim was to explore the degree to which deamidation could reliably differentiate between modern and ancient milk proteomes.

### Modern milk displays limited damage compared to ancient milk

Though the mechanisms involved are complex, and as yet poorly understood, it is clear that modern milk sources display less damage than putative ancient milk proteins. As expected, asparagine deamidation occurs relatively rapidly in both caseins and BLG, as is shown in the milk powder analysis (Fig. 1), and the contamination experiment (Fig. 4). Samples in both experiments show higher asparagine deamidation than glutamine, although asparagine deamidation is still not very advanced. Very little glutamine deamidation is observed in the experimental samples—even when the sample has undergone extreme heating, such as in the milk powder. While glutamine deamidation is useful as a crude measure of overall degradation, we stress that the extent of variability observed in this and other studies (e.g., Schroeter and Cleland^50^) —due to the extreme variability of substrates and environments, as well as the intrinsic nature of the molecules—would prevent a systematic use of glutamine deamidation alone for authentication purposes.

Glutamine deamidation of both caseins and BLG seems to increase over time, as shown in Fig. 7, however this is far from a clear pattern, and the picture is even fuzzier in the case of asparagine deamidation. More accurate dating of the archaeological samples may result in clearer trends: currently just 4% of published dataset samples have a radiocarbon date assigned; 10% of samples have a radiocarbon date from the burial context. Additionally, 7% of samples are from modern dental patients, and therefore have a reliable date. The remaining 78% of dates are inferred based on broad archaeological periods. Furthermore, the degradation of biomolecules is also affected by a sample’s thermal history; thus, the integration of thermal-ages could further improve the correlation of degradation and age^51^. Thermal age takes into account the temperature of the depositional environment, and assigns a new “age” for a sample, corrected to a constant 10 °C^32^. Therefore, samples of the same age from cold environments have a “younger” thermal age than those from hot environments, whose diagenesis would have occurred at increased rates due to the temperature. An important caveat is that these calculations are affected by a number of errors, due to both the difficulty in estimating the kinetic parameters of the reactions in archaeological samples and the uncertainty related to palaeotemperature reconstructions. Furthermore, the concept of thermal age does not take into account all the other environmental factors that affect diagenetic rates (e.g., microbial attack, pH, effect of metals and solutes)—factors which will also influence deamidation rates. Nevertheless, future studies of open-access palaeoproteomics datasets from a range of archaeological ages and sites, as well as a better understanding of the pathways of diagenesis and mechanisms of degradation may help to tease apart the relative importance of these other diagenetic forces.

### Milk proteins are difficult to authenticate on the basis of deamidation

A key concern in palaeoproteomic analyses is differentiating endogenous, ancient proteins from those originating from post-excavation and/or laboratory sources. Previous publications have outlined best practices for reducing potential contamination in ancient proteins studies, including but not limited to the use of a dedicated laboratory for ancient protein extractions, sterile equipment and pure reagents, non-proteinaceous protective equipment and clothing (e.g., no latex, silk, leather, wool), extraction blank controls, experimental replicates, and injection blanks between samples during LC–MS/MS^22^. Here, we demonstrate the importance of extracting ancient proteins within a dedicated laboratory, using appropriate controls to limit contamination from reagents and cross-sample handling. We were able to pick up low levels of casein and BLG contamination, merely by exposing archaeological substrates to a laboratory setting in which modern milk products (e.g., skim milk powder, caseins), are routinely used. Both BLG and caseins were detected in the extraction blank, demonstrating the utility of these controls in monitoring for and detecting potential contaminants within the extraction process. It is important to note that the milk contamination detected in this study is unlikely to be the results of instrument carry-over, but instead likely occurred at the extraction stage. Previously, samples from two of the archaeological sites (i.e., dental calculus samples from St. Helena and the ostrich eggshell from Leatoli) were extracted in a dedicated ancient proteins laboratory, and analyzed on the same LC–MS/MS platforms at the Target Discovery Institute; milk proteins were not detected in these samples. The ostrich eggshell sample was also exposed to high concentration bleach (NaOCl, 12% w/v) for more than 100 h—a key step in palaeoproteomics for biomineralised samples, as it ensures the isolation of endogenous (intracrystalline) proteins (see Demarchi et al.^32^). Our experiment further reinforces the importance of authenticating ancient proteins that are commonly present in laboratory reagents, such as milk, blood, and eggs^8^, and the increased burden of proof to demonstrate their authenticity.

Interestingly, caseins were more common in all of the experimental samples compared with BLG, and in all cases were identified with two or more peptides. One of the strategies for reducing the impact of false positive results in mass spectrometry is to only accept protein identifications based on the presence of two or more distinct peptides^52,53^. According to this criterion, none of the BLG identifications within the experimental samples would have been deemed confident, as none of the samples displayed more than one peptide assigned to BLG. In contrast, the casein identifications would have met this ‘two-peptide rule’. Thus, while multiple distinct peptides may act as one measure of authenticity, it is insufficient to rule out all potential contaminating sources and the ‘two peptide rule’ should not be used as a blanket rule to confirm the presence of an endogenous protein. Another strategy may be to compare the ratio of BLG to casein peptides in ancient samples. Previous analyses of archaeological dental calculus have recovered BLG in higher ratios than caseins (Fig. 8), and confident casein identifications were primarily recovered from modern dental calculus samples, or skeletons of relatively recent antiquity (e.g., post-medieval). Therefore, in dental calculus studies, a relatively high ratio of BLG compared to caseins, may be further evidence of an endogenous origin; nevertheless, the reasons for this difference in BLG and casein survival are not currently well understood.

### Database selection significantly influences peptide identifications

It is important to note that differences in peptide identifications may be due to data analysis search strategies. When performing searches using an extremely specific database (for example, of ten different milk proteins), there is an increased chance of milk proteins being identified, as the software attempts to maximise the number of matches. When performing an exploratory search, a common practice performed in previous studies for metaproteomic studies is to perform a search across a wide, yet relevant, database, such as Uniprot^54^ and/or the Human Oral Microbiome database (HOMD)^55^ to reduce the potential for false positive matches. For example, in their analysis of dietary proteins entrapped in dental calculus, Hendy et al.^8^ noted that multiple putative ‘dietary’ proteins were identified when searching dental calculus proteomes against Uniprot alone, but that these were identified as false positives after HOMD was also included within the search strategy. Once the presence of the proteins of interest has been validated, searches using smaller databases may be useful for further analyses.

Furthermore, we would also recommend manual inspection of spectra of peptides that are crucial for the correct interpretation of results. As search algorithms and data analysis strategies differ, and none are currently optimised for ancient datasets, there is not a standard score cutoff at which only reliable identifications are recorded. Lastly, in order to confirm whether recovered peptides that purport to be from a certain protein are indeed unique to a protein of a specific taxonomic group, a BLASTp search^56^ should be carried out and the results reported (see for example the impact of this approach in verifying the taxa of putative dinosaur peptides in Buckley et al.^57^). Due to the conserved nature of proteins, many peptide or protein identifications may not be species-specific. In these cases, the lowest common ancestor taxon (i.e., family, order, genus) should be clearly reported.

### Deamidation—how much is enough?

In this study, we also examine the extent to which site-specific deamidation can act as a measure of authenticity for ancient milk proteins, specifically BLG and caseins. This meta-analysis of 274 samples ranging in age from the Neolithic to the present day demonstrates that a moderate overall trend of deamidation is visible in milk proteins over time, but the real question is whether this can translate to individual studies, which will likely have a smaller number of samples and a limited time-span. Although the rule of thumb that more deamidation events at high half-time sites imply genuinely ancient proteins does seem to hold true, conclusions like this become increasingly frail as the sample size and the number of peptides recovered decreases. Even proteins which meet the ‘two-peptide’ rule may lack sufficient data to assess deamidation. For example, if two unique casein peptides are detected in a sample, only one of them may have a glutamine residue. This glutamine residue is either deamidated, or is not. Is a single deamidated residue sufficient to demonstrate authenticity? Likely not, especially considering the sporadic deamidation that was identified in modern milk proteins. DeamiDATE’s site-specific deamidation calculation may provide some nuanced information, as total deamidation at high half-time sites is more convincing evidence towards authenticity than mere “bulk” deamidation, which varies in rate. This type of sporadic, low-level recovery of milk proteins is particularly common in dental calculus studies; for example, in the meta-analysis conducted here, individuals with any evidence of BLG displayed on average only 3 BLG peptides. However, only an even smaller subset of these peptides will contain glutamine or asparagine. Only by assessing dozens of individual peptides may clear deamidation patterns emerge. These challenges in authenticating individual peptides and proteins demonstrate the necessity for relatively large and robust datasets in order to apply deamidation as a measure of antiquity. Nevertheless, the generation of such a dataset may not be possible in all ancient protein studies due to a lack of surviving peptides. Even when restricting analysis of site-specific deamidation to samples containing more than ten unique BLG peptides, obvious patterns of older samples being more deamidated do not appear (Supplementary Fig. S2).

Hendy et al.’s^15^ study on ancient proteins from ceramic residues at Çatalhöyük demonstrates variation in deamidation levels. There are a number of factors which support the authenticity of recovered proteins in this study. First, the authors followed all the laboratory guidelines for the analysis of ancient proteins^22^ sampled from three different parts of the ceramic vessels; the study made use of extraction replicates, which were analysed at different facilities, and which both produced strong evidence of dairy products. Moreover, these ceramic vessels produced an average of 13 unique casein peptides and 12 unique BLG peptides per vessel, and 5 different ruminant milk proteins, including whey proteins (BLG), caseins (alpha-S1, alpha-S2, beta-, kappa-casein) as well as a protein regulating the secretion of milk fat droplets. In contrast to the dental calculus studies which typically display the sporadic presence of relatively few BLG peptides, the Çatalhöyük vessels display evidence of a milk proteome. As the vessels were excavated from the West Mound of Çatalhöyük, they are assumed to have originated from 7100 to 5700 BCE, and represent the oldest samples in this meta-analysis. Nevertheless, a large variation in deamidation levels is found in the peptides identified in all the ceramic vessel samples, ranging from 0 to 76% remaining glutamine in BLG, and 33% to 100% in caseins. This variation, as highlighted in the bulk deamidation meta-analysis of collagen by Schroeter and Cleland^50^, does not match the expectation that thermally old samples should always be completely deamidated.

Deamidation rates may also vary based on archaeological substrate. Here, we examined deamidation levels in previously published datasets from dental calculus^7–9,27^ and ceramic vessels^15^. Although both substrates display deamidation of milk proteins, they also illuminate clear evidence of differential preservation. In the case of casein deamidation (Fig. 8), the ceramic vessels (shown in yellow diamonds) are the only samples that display nuanced levels of deamidation—all other samples have either complete deamidation or none. Although some caseins detected in archaeological substrates may be spurious, the above evidence points to preservation of genuinely ancient caseins in at least some ceramic vessel residues.

As mineralised residues adhering to ceramic vessels and calculus represent different binding substrates, it would follow that the preservation conditions would be different. Furthermore, ceramic vessels would often have contained whole milk, while dairy proteins found in dental calculus would represent the consumption of either processed or raw milk—that is, that the input proteins for these two substrates could be different. Further open-access analysis into ancient milk products, such as cheese and kefir, could shed light on how processed and raw milk proteins preserve in the absence of a mineralised substrate. This would allow a much larger abundance of data, and a full range of proteins and peptides, as the milk proteins would not be competing for sequencing against the environmental or oral microbiome proteomes. This type of analysis would allow researchers to investigate further how protein structure and peptide position influences preservation and diagenesis—including deamidation, other post-translational modifications, and hydrolysis.

Finally, different protein extraction methods may also influence site-specific deamidation rates^58–60^, and thus further systematic comparisons of protein extraction methods on modern and ancient samples are required to optimize the use of deamidation as a marker of protein diagenesis.

### Multiple lines of evidence are necessary

Using characteristic signatures of taphonomic damage to authenticate ancient biomolecular data is not new, but faces several unique challenges in palaeoproteomics. Many readers will be familiar with MapDamage^61,62^, a commonly-implemented software program designed to quantify damage patterns in ancient DNA data based on the size distribution of recovered sequencing reads and the frequency of cytosine to thymine (C-to-T) misincorporations at 5′ ends of sequences. The fundamental principle behind deamiDATE is the same, namely that we are trying to use molecular damage to authenticate ancient sequences. Nevertheless, there are notable differences between the two approaches as a consequence of the different structures of the two biopolymers, and the methods of analyses. Firstly, given that the extent of diagenetic (natural) hydrolysis is typically unknown in ancient samples, it is common to enzymatically cleave ancient proteins to obtain shorter sequences, while in ancient DNA chain scission is a wholly diagenetic phenomenon. Therefore, tryptic peptide length distributions cannot offer the same insight into preservation as DNA fragment size distributions. Non-tryptic peptides do however offer some insights into the extent of molecular fragmentation^32,63–66^. Moreover, peptides may have been exposed on the surface or buried deep within a structure, yet the dielectric constant is known to impact rates of deamidation. The double helix of DNA is largely a fixed molecular organization. In proteins, there are 20 amino acids residues as opposed to four (five) nucleotide bases and consequently there is a much greater variation in structural organization of proteins. In ancient DNA there is periodicity in fragment length resulting from the wrapping of DNA around histones^67^. It is known that deamidation rates vary as a consequence of entropic effects (e.g., secondary structure^68^) and we would envisage deamidation rates will be more difficult to assess and are impacted by the extent of degradation/denaturation. Finally, position dependent nucleotide misincorporation patterns may be assessed in every DNA sequence mapping to the genome of interest, and thus produce taphonomic data at millions or billions of distinct nucleotide sites. In contrast, deamidation rates can only be assessed at two of the 20 amino acid sites, limiting the number of datapoints available for analysis.

To increase the quantity of peptides available for site-specific analysis, we propose that the deamidation levels of proteins of interest, such as, in this case, dietary proteins, are compared to proteins that are likely to be endogenous to the substrate. For example, dietary proteins originating from dental calculus should be compared to endogenous proteins from the host. The similarity, or lack thereof, of the diagenetic status between the endogenous host proteins and the protein of interest could be used as further evidence towards genuinity. This approach has been informally applied in past analyses^51,69^, but should be adopted by future ancient protein studies.

## Conclusions

Milk proteins discovered in archaeological artefacts show deamidation signatures distinct from modern milk samples, though this damage is highly variable, and a large sample size is required to elucidate such patterns. The extent of variability in deamidation found in this study speaks against a systematic use of deamidation levels alone for the authentication of ancient proteins—multiple lines of evidence required to reliably authenticate palaeoproteomic studies^22^. This study reinforces the importance of the use of dedicated facilities for the extraction of ancient proteins, free from modern contaminants that could obfuscate results of archaeological importance—such as milk powder. Finally, site-specific deamidation represents an additional method to assess the preservation of ancient milk proteins, but damage patterns may only be robustly assessed through the careful analysis of large-scale data. Thus, it is increasingly important that raw data resulting from palaeoproteomics experiments is made open access, so that more meta-analyses may shed more light on the patterns of degradation in ancient proteomes and across various archaeological substrates.

## Supplementary Information

Supplementary Information 1.

Supplementary Information 2.

Supplementary Information 3.

Supplementary Information 4.

Supplementary Information 5.

Supplementary Information 6.

Supplementary Information 7.

Supplementary Information 8.

Supplementary Information 9.

## Data Availability

The mass spectral datasets generated during the current study are available via the PRIDE database (https://www.ebi.ac.uk/pride/) with the following credentials: Projet PXD022368.
