# Author Correction: Assessing the degradation of ancient milk proteins through site-specific deamidation patterns

**DOI:** 10.1038/s41598-022-06151-5

**Published:** 2022-02-01

**Authors:** Abigail Ramsøe, Mia Crispin, Meaghan Mackie, Krista McGrath, Roman Fischer, Beatrice Demarchi, Matthew J. Collins, Jessica Hendy, Camilla Speller

**Affiliations:** 1 BioArCh, Department of Archaeology, University of York, York, UK; 2 Department of Earth Sciences, Natural History Museum, London, UK; 3 The GLOBE Institute, University of Copenhagen, Copenhagen, Denmark; 4 The Novo Nordisk Foundation Center for Protein Research, University of Copenhagen, Copenhagen, Denmark; 5 Department of Prehistory and Institute of Environmental Science and Technology (ICTA), Universitat Autònoma de Barcelona, Bellaterra, Spain; 6 Target Discovery Institute, Nuffield Department of Medicine, University of Oxford, Oxford, UK; 7 Department of Life Sciences and Systems Biology, University of Turin, Turin, Italy; 8 McDonald Institute for Archaeological Research, University of Cambridge, Cambridge, UK; 9 Department of Anthropology, University of British Columbia, Vancouver, Canada

Correction to: *Scientific Reports* 10.1038/s41598-021-87125-x, published online 08 April 2021

The Funding section in the original version of this Article was omitted. The Funding section now reads:

“This work was supported by Danish National Research Foundation DNRF128.”

The original Article has been corrected.

