# Supplementary material for: Assessing the degradation of ancient milk proteins through site-specific deamidation patterns
Source: Sci Rep. 2021 Apr 8;11:7795. doi: 10.1038/s41598-021-87125-x (PMC8032661; doi:10.1038/s41598-021-87125-x)
Supplement: Supplementary file 1 — Supplementary Information 1. [file 41598_2021_87125_MOESM1_ESM.pdf]

>sp|P04653|CASA1_SHEEP Alpha-S1-casein OS=Ovis aries OX=9940 GN=CSN1S1 PE=1 SV=3
MKLLILTCLVAVALARPKHPIKHQGLSSEVLNENLLRFVVAPFPEVFRKENINELSKDIG
SESIEDQAMEDAKQMKAGSSSSSEEIVPNSAEQKYIQKEDVPSERYLGYLEQLLRLKKYN
VPQLEIVPKSAEEQLHSMKEGNPAHQKQPMIAVNQELAYFYPQLFRQFYQLDAYPSGAWY
YLPLGTQYTDAPSFSDIPNPIGSENSGKITMPLW
>sp|P02669|CASK_SHEEP Kappa-casein OS=Ovis aries OX=9940 GN=CSN3 PE=1 SV=2
MMKSFFLVVTILALTLPFLGAQEQNQEQRICCEKDERFFDDKIAKYIPIQYVLSRYPSYG
LNYYQQRPVALINNQFLPYPYYAKPVAVRSPAQTLQWQVLPNAVPAKSCQDQPTAMARHP
HPHLSFMAIPPKKDQDKTEIPAINTIASAEPTVHSTPTTEAVVNAVDNPEASSESIASAP
ETNTAQVTSTEV
>sp|P11839|CASB_SHEEP Beta-casein OS=Ovis aries OX=9940 GN=CSN2 PE=1 SV=3
MKVLILACLVALALAREQEELNVVGETVESLSSSEESITHINKKIEKFQSEEQQQTEDEL
QDKIHPFAQAQSLVYPFTGPIPNSLPQNILPLTQTPVVVPPFLQPEIMGVPKVKETMVPK
HKEMPFPKYPVEPFTESQSLTLTDVEKLHLPLPLVQSWMHQPPQPLPPTVMFPPQSVLSL
SQPKVLPVPQKAVPQRDMPIQAFLLYQEPVLGPVRGPFPILV
>sp|P04654|CASA2_SHEEP Alpha-S2-casein OS=Ovis aries OX=9940 GN=CSN1S2 PE=2 SV=1
MKFFIFTCLLAVALAKHKMEHVSSSEEPINISQEIYKQEKNMAIHPRKEKLCTTSCEEVV
RNADEEEYSIRSSSEESAEVAPEEVKITVDDKHYQKALNEINQFYQKFPQYLQYLYQGPI
VLNPWDQVKRNAGPFTPTVNREQLSTSEENSKKTIDMESTEVFTKKTKLTEEEKNRLNFL
KKISQYYQKFAWPQYLKTVDQHQKAMKPWTQPKTNAIPYVRYL
>sp|P67976|LACB_SHEEP Beta-lactoglobulin-1/B OS=Ovis aries OX=9940 PE=1 SV=1
MKCLLLALGLALACGVQAIIVTQTMKGLDIQKVAGTWHSLAMAASDISLLDAQSAPLRVY
VEELKPTPEGNLEILLQKWENGECAQKKIIAEKTKIPAVFKIDALNENKVLVLDTDYKKY
LLFCMENSAEPEQSLACQCLVRTPEVDNEALEKFDKALKALPMHIRLAFNPTQLEGQCHV
>sp|P02758|LACB1_HORSE Beta-lactoglobulin-1 OS=Equus caballus OX=9796 GN=LGB1 PE=1 SV=3
MKCLLLALGLALMCGIQATNIPQTMQDLDLQEVAGKWHSVAMAASDISLLDSESAPLRVY
IEKLRPTPEDNLEIILREGENKGCAEKKIFAEKTESPAEFKINYLDEDTVFALDTDYKNY
LFLCMKNAATPGQSLVCQYLARTQMVDEEIMEKFRRALQPLPGRVQIVPDLTRMAERCRI
>sp|P07380|LACB2_HORSE Beta-lactoglobulin-2 OS=Equus caballus OX=9796 GN=LGB2 PE=1 SV=3
MKCLLLALGLSLMCGNQATDIPQTMQDLDLQEVAGRWHSVAMVASDISLLDSESVPLRVY
VEELRPTPEGNLEIILREGANHACVERNIVAQKTEDPAVFTVNYQGERKISVLDTDYAHY
MFFCVGPPLPSAEHGMVCQYLARTQKVDEEVMEKFSRALQPLPGRVQIVQDPSGGQERCG
F
>sp|Q9GKK3|CASB_HORSE Beta-casein OS=Equus caballus OX=9796 GN=CSN2 PE=1 SV=3
MKILILACLVALALAREKEELNVSSETVESLSSNEPDSSSEESITHINKEKLQKFKHEGQ
QQREVERQDKISRFVQPQPVVYPYAEPVPYAVVPQSILPLAQPPILPFLQPEIMEVSQAK
ETILPKRKVMPFLKSPIVPFSERQILNPTNGENLRLPVHLIQPFMHQVPQSLLQTLMLPS
QPVLSPPQSKVAPFPQPVVPYPQRDTPVQAFLLYQDPRLGPTGELDPATQPIVAVHNPVI
V
>sp|P82187|CASK_HORSE Kappa-casein OS=Equus caballus OX=9796 GN=CSN3 PE=1 SV=2
MKSFFLVVNILALTLPFLGAEVQNQEQPTCHKNDERFFDLKTVKYIPIYYVLNSSPRYEP

IYYQHRLALLINNQHMPYQYYARPAAVRPHVQIPQWQVLPNIYPSTVVRHPCPHPSFIAI
PPKKLQEITVIPKINTIATVEPTPIPTPEPTVNNAVIPDASSEFIIASTPETTTVPVTSP
VVQKL
>tr|Q8SPR1|Q8SPR1_HORSE Alpha s1 casein OS=Equus caballus OX=9796 PE=2
SV=1
MKLLILTCLVAVALARPKLPHRQPEIIQNEQDSREKVLKERKFPSFALEYINELNRQREL
LKEKQKDEHKEYLIEDPEQQESSSTSSSEEVVPINTEQKRIPREDMLYQHTLEQLRRLSK
YNQLQLQAIHAQEQLIRMKENSQRKPMRVVNQEQAYFYLEPFQPSYQLDVYPYAAWFHPA
QIMQHVAYSPFHDTAKLIASENSEKTDIIPEW
>tr|D2KAS0|D2KAS0_HORSE Alpha-S2-casein variant B OS=Equus caballus
OX=9796 GN=CSN1S2 PE=2 SV=1
MKFFIFTCLLAVALAKHNMEHRSSSEDSVNISQEKFKQEKYVVIPTSKESICSTSCEEAT
RNINEMESAKFPTEREEKEVEEKHHLKQLNKINQFYEKLNFLQYLQALRQPRIVLTPWDQ
TKTGDSPFIPIVNTEQLFTSEEIPKKTVDMESTEVVTEKTELTEEEKNYLKLLYYEKFTL
PQYFKIVRQHQTTMDPRSHRKTNSYQIIPVLRYF
>tr|A0A0C5DH76|A0A0C5DH76_HORSE Alpha-S2-casein variant A OS=Equus
caballus OX=9796 GN=CSN1S2 PE=2 SV=1
MKFFIFTCLLAVALAKHNMEHRSSSEDSVNISQEKFKQEKYVVIPTSKESICSTSCEEAT
RNINEMESAKFPTEVYSSSSSSEESAKFPTEREEKEVEEKHHLKQLNKINQFYEKLNFLQ
YLQALRQPRIVLTPWDQTKTGDSPFIPIVNTEQLFTSEEIPKKTVDMESTEVVTEKTELT
EEEKNYLKLLYYEKFTLPQYFKIVRQHQTTMDPRSHRKTNSYQIIPVLRYF
>sp|P02670|CASK_CAPHI Kappa-casein OS=Capra hircus OX=9925 GN=CSN3
PE=1 SV=2
MMKSFFLVVTILALTLPFLGAQEQNQEQPICCEKDERFFDDKIAKYIPIQYVLSRYPSYG
LNYYQQRPVALINNQFLPYPYYAKPVAVRSPAQTLQWQVLPNTVPAKSCQDQPTTLARHP
HPHLSFMAIPPKKDQDKTEVPAINTIASAEPTVHSTPTTEAIVNTVDNPEASSESIASAS
ETNTAQVTSTEV
>sp|P18626|CASA1_CAPHI Alpha-S1-casein OS=Capra hircus OX=9925
GN=CSN1S1 PE=1 SV=2
MKLLILTCLVAVALARPKHPINHRGLSPEVPNENLLRFVVAPFPEVFRKENINELSKDIG
SESTEDQAMEDAKQMKAGSSSSSEEIVPNSAEQKYIQKEDVPSERYLGYLEQLLRLKKYN
VPQLEIVPKSAEEQLHSMKEGNPAHQKQPMIAVNQELAYFYPQLFRQFYQLDAYPSGAWY
YLPLGTQYTDAPSFSDIPNPIGSENSGKTTMPLW
>sp|P33049|CASA2_CAPHI Alpha-S2-casein OS=Capra hircus OX=9925
GN=CSN1S2 PE=2 SV=1
MKFFIFTCLLAVALAKHKMEHVSSSEEPINIFQEIYKQEKNMAIHPRKEKLCTTSCEEVV
RNANEEEYSIRSSSEESAEVAPEEIKITVDDKHYQKALNEINQFYQKFPQYLQYPYQGPI
VLNPWDQVKRNAGPFTPTVNREQLSTSEENSKKTIDMESTEVFTKKTKLTEEEKNRLNFL
KKISQYYQKFAWPQYLKTVDQHQKAMKPWTQPKTNAIPYVRYL
>sp|P33048|CASB_CAPHI Beta-casein OS=Capra hircus OX=9925 GN=CSN2 PE=2
SV=1
MKVLILACLVALAIAREQEELNVVGETVESLSSSEESITHINKKIEKFQSEEQQQTEDEL
QDKIHPFAQAQSLVYPFTGPIPNSLPQNILPLTQTPVVVPPFLQPEIMGVPKVKETMVPK
HKEMPFPKYPVEPFTESQSLTLTDVEKLHLPLPLVQSWMHQPPQPLSPTVMFPPQSVLSL
SQPKVLPVPQKAVPQRDMPIQAFLLYQEPVLGPVRGPFPILV
>sp|P02754|LACB_BOVIN Beta-lactoglobulin OS=Bos taurus OX=9913 GN=LGB
PE=1 SV=3
MKCLLLALALTCGAQALIVTQTMKGLDIQKVAGTWYSLAMAASDISLLDAQSAPLRVYVE
ELKPTPEGDLEILLQKWENGECAQKKIIAEKTKIPAVFKIDALNENKVLVLDTDYKKYLL
FCMENSAEPEQSLACQCLVRTPEVDDEALEKFDKALKALPMHIRLSFNPTQLEEQCHI

>tr|B2YKY6|B2YKY6_CAPHI Beta-lactoglobulin (Fragment) OS=Capra hircus
OX=9925 GN=BLG PE=3 SV=1
LNENKVLVLDTDYKKYLLFCMENSAEPEQSLACQCLVRTPEVDKEALEKFDKALKALPMH
IRLAFNPTQLE
>sp|P02662|CASA1_BOVIN Alpha-S1-casein OS=Bos taurus OX=9913 GN=CSN1S1
PE=1 SV=2
MKLLILTCLVAVALARPKHPIKHQGLPQEVLNENLLRFFVAPFPEVFGKEKVNELSKDIG
SESTEDQAMEDIKQMEAESISSSEEIVPNSVEQKHIQKEDVPSERYLGYLEQLLRLKKYK
VPQLEIVPNSAEERLHSMKEGIHAQQKEPMIGVNQELAYFYPELFRQFYQLDAYPSGAWY
YVPLGTQYTDAPSFSDIPNPIGSENSEKTTMPLW
>sp|P02666|CASB_BOVIN Beta-casein OS=Bos taurus OX=9913 GN=CSN2 PE=1
SV=2
MKVLILACLVALALARELEELNVPGEIVESLSSSEESITRINKKIEKFQSEEQQQTEDEL
QDKIHPFAQTQSLVYPFPGPIPNSLPQNIPPLTQTPVVVPPFLQPEVMGVSKVKEAMAPK
HKEMPFPKYPVEPFTESQSLTLTDVENLHLPLPLLQSWMHQPHQPLPPTVMFPPQSVLSL
SQSKVLPVPQKAVPYPQRDMPIQAFLLYQEPVLGPVRGPFPIIV
>sp|P02668|CASK_BOVIN Kappa-casein OS=Bos taurus OX=9913 GN=CSN3 PE=1
SV=1
MMKSFFLVVTILALTLPFLGAQEQNQEQPIRCEKDERFFSDKIAKYIPIQYVLSRYPSYG
LNYYQQKPVALINNQFLPYPYYAKPAAVRSPAQILQWQVLSNTVPAKSCQAQPTTMARHP
HPHLSFMAIPPKKNQDKTEIPTINTIASGEPTSTPTTEAVESTVATLEDSPEVIESPPEI
NTVQVTSTAV
>sp|P02663|CASA2_BOVIN Alpha-S2-casein OS=Bos taurus OX=9913 GN=CSN1S2
PE=1 SV=2
MKFFIFTCLLAVALAKNTMEHVSSSEESIISQETYKQEKNMAINPSKENLCSTFCKEVVR
NANEEEYSIGSSSEESAEVATEEVKITVDDKHYQKALNEINQFYQKFPQYLQYLYQGPIV
LNPWDQVKRNAVPITPTLNREQLSTSEENSKKTVDMESTEVFTKKTKLTEEEKNRLNFLK
KISQRYQKFALPQYLKTVYQHQKAMKPWIQPKTKVIPYVRYL
