# Supplementary figures and images for: Assessing the degradation of ancient milk proteins through site-specific deamidation patterns
Source: Sci Rep. 2021 Apr 8;11:7795. doi: 10.1038/s41598-021-87125-x (PMC8032661; doi:10.1038/s41598-021-87125-x)

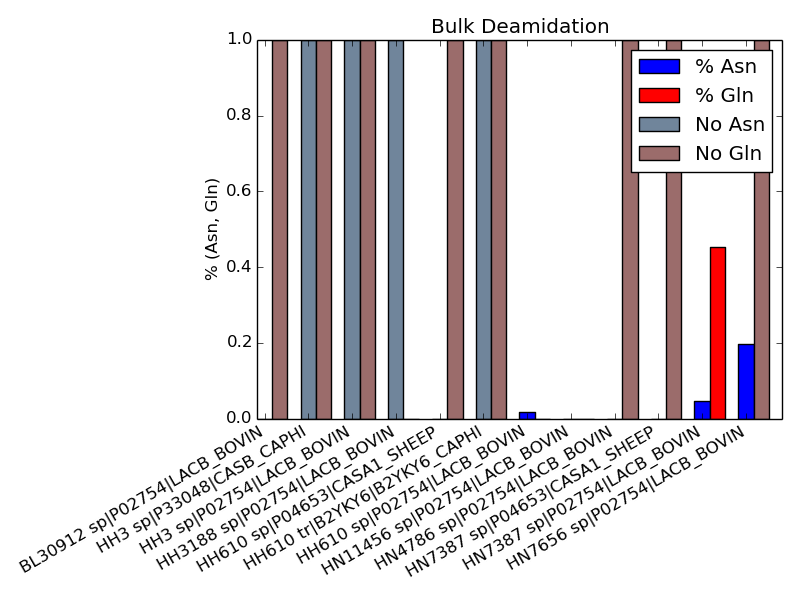

Supplement: Supplementary file 3 — Supplementary Information 3. [file 41598_2021_87125_MOESM3_ESM.zip › Charlton2019-deamResults/Bulk_plot.png]
