# Supplementary material for: Assessing the degradation of ancient milk proteins through site-specific deamidation patterns
Source: Sci Rep. 2021 Apr 8;11:7795. doi: 10.1038/s41598-021-87125-x (PMC8032661; doi:10.1038/s41598-021-87125-x)
Supplement: Supplementary file 4 — Supplementary Information 4. [file 41598_2021_87125_MOESM4_ESM.docx]

Supplementary Information for:

Assessing the degradation of ancient milk proteins through site-specific deamidation patterns

**Authors:**

Abigail Ramsøe^1,2*^*, Mia Crispin^1^, Meaghan Mackie^3,4^, Krista McGrath^1,5^, Roman Fischer^6^, Beatrice Demarchi^7^, Matthew J. Collins^3,8^, Jessica Hendy^1^, Camilla Speller*^1,9^

**Affiliations:**

1 BioArCh, Department of Archaeology, University of York, York, UK

2 Department of Earth Sciences, Natural History Museum, London, UK

3 The GLOBE Institute, University of Copenhagen, Copenhagen, Denmark

4 The Novo Nordisk Foundation Center for Protein Research, University of Copenhagen, Denmark

5 Department of Prehistory and Institute of Environmental Science and Technology (ICTA), Universitat Autònoma de Barcelona, Bellaterra, Spain

6 Target Discovery Institute, Nuffield Department of Medicine, University of Oxford, UK

7 Department of Life Sciences and Systems Biology, University of Turin, Italy

8 McDonald Institute for Archaeological Research, University of Cambridge, Cambridge, UK

9 Department of Anthropology, University of British Columbia, Vancouver, Canada

## **Supplementary Information**

##
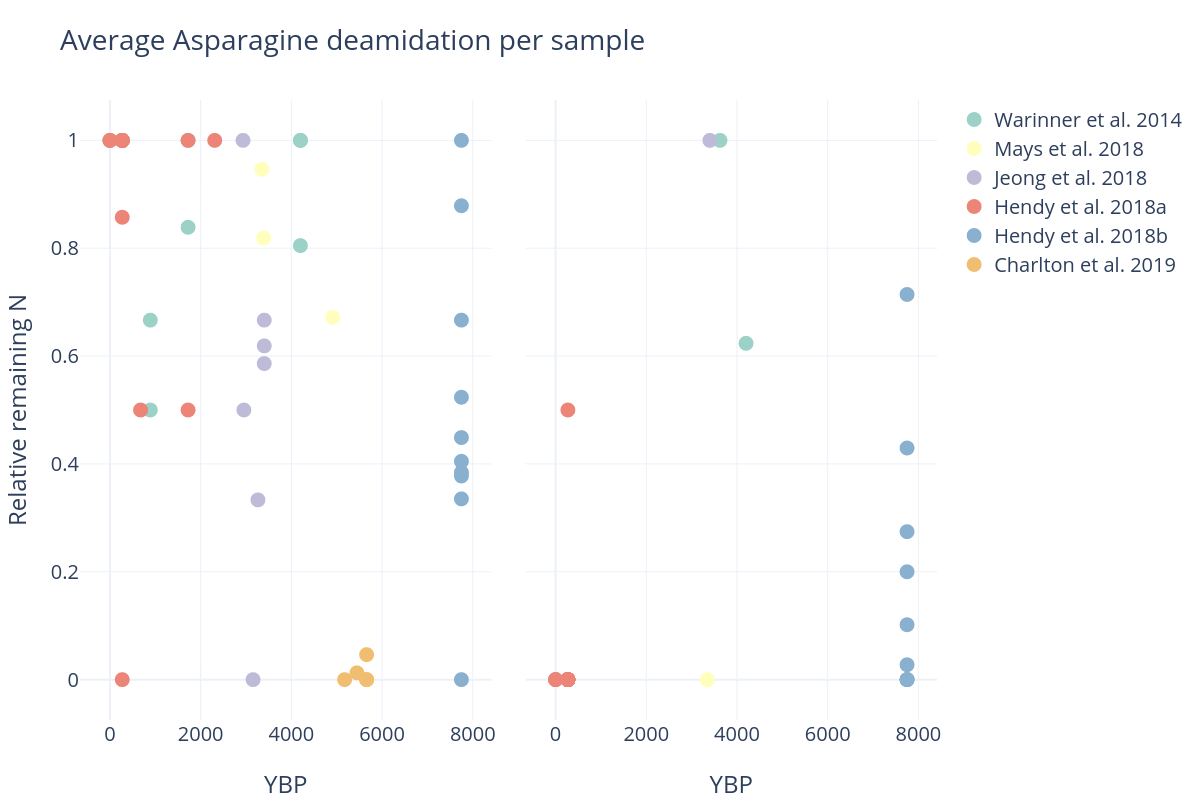


## Figure S1: Average asparagine deamidation per sample within previously published archaeological datasets (left panel BLG; right panel caseins).


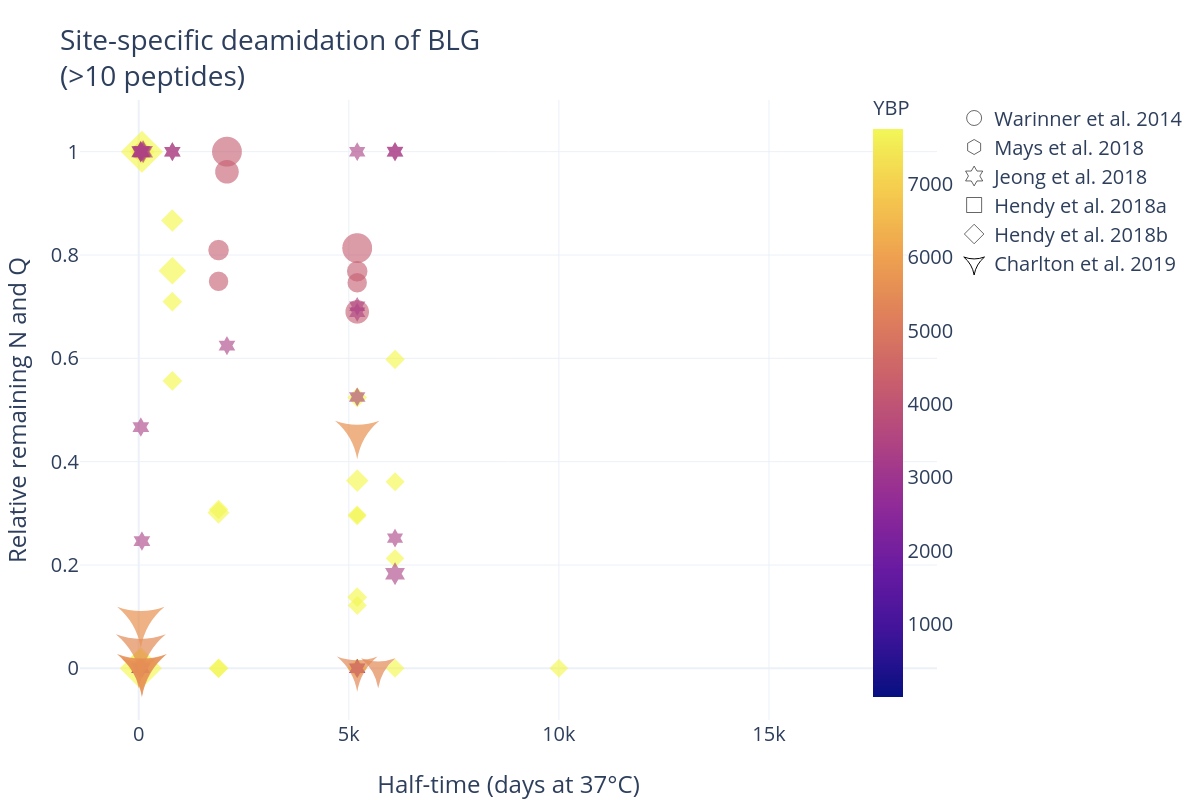


Figure S2: Site-specific deamidation in previously published archaeological samples containing more than ten unique BLG peptides.
