# Supplementary material for: Assessing the degradation of ancient milk proteins through site-specific deamidation patterns
Source: Sci Rep. 2021 Apr 8;11:7795. doi: 10.1038/s41598-021-87125-x (PMC8032661; doi:10.1038/s41598-021-87125-x)
Supplement: Supplementary file 5 — Supplementary Information 5. [file 41598_2021_87125_MOESM5_ESM.docx]

Figure 5 shows the presence of three outlier positions in the limescale sample - with a high half-time and a complete deamidation.

| **Sample** | **Peptide** | **Score** | **Half-time** | **Assigned protein** | **Unique?** |
| --- | --- | --- | --- | --- | --- |
| Limescale | FPQPVVPYPQR | 62 | 6400 | Horse CASB | Equus unique, no paralogues |
| Limescale | IVLTPWDQTK | 65 | 5400 | Horse CASA2 | Equus unique, sequence differences on non-deamidating residues |
| Limescale | YQGPIVLNPWDQVK | 64 | 7600 | Cow CASA2 | Unique to CASA2, non unique to species |

Although all these peptides are unique to milk proteins, it is unlikely that horse casein was present in the laboratory environment that contaminated these samples. This, combined with the low score (just above the cutoff of 60), implies that these identifications are likely false positives.

Though not depicted in Fig. 5, there are also BLG-origin outliers in this dataset.

| **Sample** | **Peptide** | **Score** | **Half-time** | **Assigned protein** | **Unique?** |
| --- | --- | --- | --- | --- | --- |
| Blank | DLQEVAGR | 63 | 7400 | Horse BLG | Not unique, shared by bacteria |
| Dental calculus B | RALQPLPGR | 61 | 10000 | Horse BLG | Equus unique, no paralogues |

Although the peptide found in the blank could plausibly have originated from a multitude of sources, as above, it is unlikely that horse BLG contaminated dental calculus in the laboratory environment.
