# Supplementary material for: Assessing the degradation of ancient milk proteins through site-specific deamidation patterns
Source: Sci Rep. 2021 Apr 8;11:7795. doi: 10.1038/s41598-021-87125-x (PMC8032661; doi:10.1038/s41598-021-87125-x)
Supplement: Supplementary file 2 — Supplementary Information 2. [file 41598_2021_87125_MOESM2_ESM.zip › zip/Warinner2014.zip!/Warinner2014/St_Helena/tables.pdf]

Summary

The summary file contains summary information for all the raw files processed with a single MaxQuant run. The summary information consists of some MaxQuant parameters, information of the raw file contents, and statistics on the peak detection. Based on this file a quick overview can be gathered on the quality of the data in the raw file.

The last row in this file contains the summary information for each column on each of the processed files.

Name	Separator	Description
Raw file		The raw file processed.
Experiment		Experiment name assigned to this LC-MS run in the experimental design.
Enzyme		The protease used to digest the protein sample.
Enzyme mode		The protease used to digest the protein sample.
Enzyme first search		The protease used for the first search.
Enzyme mode first search		The protease used for the first search.
Use enzyme first search		Marked with '+' when a different protease setup was used for the first search.
Variable modifications		The variable modification(s) used during the identification of peptides.
Fixed modifications		The fixed modification(s) used during the identification of peptides.
Multi modifications		The multi modification(s) used during the identification of peptides.
Variable modifications first search		The variable modification(s) used during the first search.
Use variable modifications first search		Marked with '+' when different variable modifications were used for the first search.
Requantify		The number of labels used.
Multiplicity		The number of labels used.
Max. missed cleavages		The maximum allowed number of missed cleavages.
Labels0		The labels used in the labeling experiment. Allowed values for X: 0=light; 1=medium; 2=heavy label partner.
LC-MS run type		The type of LC-MS run. Usually it will be 'Standard' which refers to a conventional shotgun proteomics run with data-dependent MS/MS.
Time-dependent recalibration		When marked with '+', time-dependent recalibration was applied to improve the data quality.
MS		The number of MS spectra recorded in this raw file.
MS/MS		The number of MS/MS spectra recorded in this raw file.
MS3		The number of MS3 spectra recorded in this raw file.
MS/MS Submitted		The number of tandem MS spectra submitted for analysis.
MS/MS Submitted (SIL)		The number of tandem MS spectra submitted for analysis, where the precursor ion was detected as part of a labeling cluster.
MS/MS Submitted (ISO)		The number of tandem MS spectra submitted for analysis, where the precursor ion was detected as an isotopic pattern.
MS/MS Submitted (PEAK)		The number of tandem MS spectra submitted for analysis, where the precursor ion was detected as a single peak.
MS/MS Identified		The total number of identified tandem MS spectra.
MS/MS Identified (SIL)		The total number of identified tandem MS spectra, where the precursor ion was detected as part of a labeling cluster.
MS/MS Identified (ISO)		The total number of identified tandem MS spectra, where the precursor ion was detected as an isotopic pattern.
MS/MS Identified (PEAK)		The total number of identified tandem MS spectra, where the precursor ion was detected as a single peak.
MS/MS Identified [%]		The percentage of identified tandem MS spectra.
MS/MS Identified (SIL) [%]		The percentage of identified tandem MS spectra, where the precursor ion was detected as part of a labeling cluster.
MS/MS Identified (ISO) [%]		The percentage of identified tandem MS spectra, where the precursor ion was detected as an isotopic pattern.
MS/MS Identified (PEAK) [%]		The percentage of identified tandem MS spectra, where the precursor ion was detected as a single peak.
Peptide Sequences Identified		The total number of unique peptide amino acid sequences identified from the recorded tandem mass spectra.
Peaks		The total number of peaks detected in the full scans.
Peaks Sequenced		The total number of peaks sequenced by tandem MS.

Peaks Sequenced [%]		The percentage of peaks sequenced by tandem MS.
Peaks Repeatedly Sequenced		The total number of peaks repeatedly sequenced (i.e. 1 or more times) by tandem MS.
Peaks Repeatedly Sequenced [%]		The percentage of peaks repeatedly sequenced (i.e. 1 or more times) by tandem MS.
Isotope Patterns		The total number of detected isotope patterns.
Isotope Patterns Sequenced		The total number of isotope patterns sequenced by tandem MS.
Isotope Patterns Sequenced (z>1)		The total number of isotope patterns sequenced by tandem MS with a charge state of 2 or more.
Isotope Patterns Sequenced [%]		The percentage of isotope patterns sequenced by tandem MS.
Isotope Patterns Sequenced (z>1) [%]		The percentage of isotope patterns sequenced by tandem MS with a charge state of 2 or more.
Isotope Patterns Repeatedly Sequenced		The total number of isotope patterns repeatedly sequenced (i.e. 1 or more times) by tandem MS.
Isotope Patterns Repeatedly Sequenced [%]		The percentage of isotope patterns repeatedly sequenced (i.e. 1 or more times) by tandem MS.
Recalibrated		When marked with '+', the masses taken from the raw file were recalibrated.
Av. Absolute Mass Deviation [ppm]		The average absolute mass deviation found comparing to the identification mass in parts per million.
Mass Standard Deviation [ppm]		The standard deviation of the mass deviation found comparing to the identification mass in parts per million.
Av. Absolute Mass Deviation [mDa]		The average absolute mass deviation found comparing to the identification mass in milli-Dalton.
Mass Standard Deviation [mDa]		The standard deviation of the mass deviation found comparing to the identification mass in milli-Dalton.

Evidence

The evidence file combines all the information about the identified peptides and normally is the only file required for processing the results. Additional information about the peptides, modifications, proteins, etc. can be found in the other files by unique identifier linkage.

**Name**	**Separator**	**Description**
Sequence		The identified AA sequence of the peptide.
Length		The length of the sequence stored in the column 'Sequence'.
Modifications		Post-translational modifications contained within the identified peptide sequence.
Modified sequence		Sequence representation including the post-translational modifications (abbreviation of the modification in brackets before the modified AA). The sequence is always surrounded by underscore characters ('_').
Deamidation (NQ) Probabilities		Sequence representation of the peptide including PTM positioning probabilities ([0..1], where 1 is best match) for 'Deamidation (NQ)'.
Hydroxyproline Probabilities		Sequence representation of the peptide including PTM positioning probabilities ([0..1], where 1 is best match) for 'Hydroxyproline'.
Deamidation (NQ) Score Diffs		Sequence representation for each of the possible PTM positions in each possible configuration, the difference is calculated between the identification score with the PTM added to that position and the best scoring identification where no PTM is added to that position. When this value is negative, it is unlikely that the particular modification is located at this position.
Hydroxyproline Score Diffs		Sequence representation for each of the possible PTM positions in each possible configuration, the difference is calculated between the identification score with the PTM added to that position and the best scoring identification where no PTM is added to that position. When this value is negative, it is unlikely that the particular modification is located at this position.
Acetyl (Protein N-term)		The number of occurrences of the modification 'Acetyl (Protein N-term)'.
Deamidation (NQ)		The number of occurrences of the modification 'Deamidation (NQ)'.
Gln->pyro-Glu		The number of occurrences of the modification 'Gln->pyro-Glu'.
Glu->pyro-Glu		The number of occurrences of the modification 'Glu->pyro-Glu'.
Hydroxyproline		The number of occurrences of the modification 'Hydroxyproline'.
Oxidation (M)		The number of occurrences of the modification 'Oxidation (M)'.
Missed cleavages		Number of missed enzymatic cleavages.
Proteins		The identifiers of the proteins this particular peptide is associated with.
Leading proteins		The identifiers of the proteins in the proteinGroups file, with this protein as best match, this particular peptide is associated with. When multiple matches are found here, the best scoring protein can be found in the 'Leading Razor Protein' column.
Leading razor protein		The identifier of the best scoring protein, from the proteinGroups file this, this peptide is associated to.
Type		The type of the feature. 'MSMS' – for an MS/MS spectrum without an MS1 isotope pattern assigned. 'ISO-MSMS' – MS1 isotope cluster identified by MS/MS. 'MULTI-MSMS' – MS1 labeling cluster identified by MS/MS. 'MULTI-SECPEP' – MS1 labeling cluster identified by MS/MS as second peptide. 'MULTI-MATCH' – MS1 labeling cluster identified by matching between runs. In case of label-free data there is no difference between 'MULTI' and 'ISO'.
Raw file		The name of the RAW-file the mass spectral data was derived from.
Experiment		
MS/MS m/z		The m/z used for fragmentation (not necessarily the mono-isotopic m/z).
Charge		The charge-state of the precursor ion.
m/z		The recalibrated mass-over-charge value of the precursor ion.
Mass		The predicted monoisotopic mass of the identified peptide sequence.
Resolution		The resolution of precursor ion measured in Full Width at Half Maximum (FWHM).

Uncalibrated - Calibrated m/z [ppm]		The difference between the uncalibrated and recalibrated mass-over-charge value of the precursor ion measured in parts-per-million. This gives an indication of the mass drift in the original data, which was automatically corrected by MaxQuant.
Uncalibrated - Calibrated m/z [Da]		The difference between the uncalibrated and recalibrated mass-over-charge value of the precursor ion measured in parts-per-million. This gives an indication of the mass drift in the original data, which was automatically corrected by MaxQuant.
Mass error [ppm]		Mass error of the recalibrated mass-over-charge value of the precursor ion in comparison to the predicted monoisotopic mass of the identified peptide sequence in parts per million.
Mass error [Da]		Mass error of the recalibrated mass-over-charge value of the precursor ion in comparison to the predicted monoisotopic mass of the identified peptide sequence in milli-Dalton.
Uncalibrated mass error [ppm]		Mass error of the uncalibrated mass-over-charge value of the precursor ion in comparison to the predicted monoisotopic mass of the identified peptide sequence.<br><br>Note: This column can contain missing values (denoted as NaN).
Uncalibrated mass error [Da]		Mass error of the uncalibrated mass-over-charge value of the precursor ion in comparison to the predicted monoisotopic mass of the identified peptide sequence.<br><br>Note: This column can contain missing values (denoted as NaN).
Max intensity m/z 0		Summed up eXtracted Ion Current (XIC) of all isotopic clusters associated with the identified AA sequence. In case of a labeled experiment this is the total intensity of all the isotopic patterns in the label cluster.
Retention time		The uncalibrated retention time in minutes in the elution profile of the precursor ion.
Retention length		The total retention time length of the peak (last timepoint – first timepoint).
Calibrated retention time		The recalibrated retention time in minutes in the elution profile of the precursor ion.
Calibrated retention time start		The recalibrated retention start in minutes in the elution profile of the precursor ion.
Calibrated retention time finish		The recalibrated retention finish in minutes in the elution profile of the precursor ion.
Retention time calibration		The difference in minutes between the uncalibrated and recalibrated retention time. This gives an indication of the retention time drift in the original data, which was automatically corrected by MaxQuant.<br><br>Note: This column can contain missing values (NaN).
Match time difference		When the option ‘match between runs’ is used in MaxQuant, this value indicates the time difference between the feature from the raw file it was taken from and the feature from the raw file it was matched to.
Match m/z difference		When the option ‘match between runs’ is used in MaxQuant, this value indicates the m/z difference between the feature from the raw file it was taken from and the feature from the raw file it was matched to.
Match q-value		This is the q-value for features that have been identified by 'matching between runs'.
Match score		The andromeda score of the MS/MS identification that is the source of this identification by 'matching between runs'.
Number of data points		The number of data points (peak centroids) collected for this peptide feature.
Number of scans		The number of MS scans that the 3d peaks of this peptide feature are overlapping with.
Number of isotopic peaks		The number of isotopic peaks contained in this peptide feature.
PIF		Short for Parent Ion Fraction; indicates the fraction the target peak makes up of the total intensity in the inclusion window.
Fraction of total spectrum		The percentage the ion intensity makes up of the total intensity of the whole spectrum.
Base peak fraction		The percentage the parent ion intensity in comparison to the highest peak in the MS spectrum.
PEP		Posterior Error Probability of the identification. This value essentially operates as a p-value, where smaller is more significant.
MS/MS count		The number of sequencing events for this sequence, which matches the number of identifiers stored in the column ‘MS/MS IDs’.
MS/MS scan number		The RAW-file derived scan number of the MS/MS with the highest peptide identification score (the highest score is stored in the column 'Score').
Score		Andromeda score for the best associated MS/MS spectrum.
Delta score		Score difference to the second best identified peptide.

Combinatorics		Number of possible distributions of the modifications over the peptide sequence.
Intensity		Summed up eXtracted Ion Current (XIC) of all isotopic clusters associated with the identified AA sequence. In case of a labeled experiment this is the total intensity of all the isotopic patterns in the label cluster.
Reverse		When marked with '+', this particular peptide was found to be part of a protein derived from the reversed part of the decoy database. These should be removed for further data analysis.
Potential contaminant		When marked with '+', this particular peptide was found to be part of a commonly occurring contaminant. These should be removed for further data analysis.
id		A unique (consecutive) identifier for each row in the evidence table, which is used to cross-link the information in this file with the information stored in the other files.
Protein group IDs		The identifier of the protein-group this redundant peptide sequence is associated with, which can be used to look up the extended protein information in the file 'proteinGroups.txt'. As a single peptide can be linked to multiple proteins (e.g. in the case of razor-proteins), multiple id's can be stored here separated by a semicolon. As a protein can be identified by multiple peptides, the same id can be found in different rows.
Peptide ID		The identifier of the non-redundant peptide sequence.
Mod. peptide ID		Identifier of the associated modification summary stored in the file 'modificationSpecificPeptides.txt'.
MS/MS IDs		Identifier(s) of the associated MS/MS summary(s) stored in the file 'msms.txt'.
Best MS/MS		Identifier(s) of the best MS/MS associated spectrum stored in the file 'msms.txt'.
AIF MS/MS IDs		Identifier(s) of the associated All Ion Fragmentation MS/MS summary(s) stored in the file 'aifMsms.txt'.
Deamidation (NQ) site IDs		Identifier(s) of the modification summary stored in the file 'Deamidation (NQ)Sites.txt'.
Hydroxyproline site IDs		Identifier(s) of the modification summary stored in the file 'HydroxyprolineSites.txt'.

Peptides

The peptides table contains information on the identified peptides in the processed raw-files.

**Name**	**Separator**	**Description**
Sequence		The amino acid sequence of the identified peptide.
N-term cleavage window		Sequence window from -15 to 15 around the N-terminal cleavage site of this peptide.
C-term cleavage window		Sequence window from -15 to 15 around the C-terminal cleavage site of this peptide.
Amino acid before		The amino acid in the protein sequence before the peptide.
First amino acid		The amino acid in the first position of the peptide sequence.
Second amino acid		The amino acid in the first position of the peptide sequence.
Second last amino acid		The amino acid in the last position of the peptide sequence.
Last amino acid		The amino acid in the last position of the peptide sequence.
Amino acid after		The amino acid in the protein sequence after the peptide.
A Count		The number of instances of the 'A' amino acid contained within the sequence.
R Count		The number of instances of the 'R' amino acid contained within the sequence.
N Count		The number of instances of the 'N' amino acid contained within the sequence.
D Count		The number of instances of the 'D' amino acid contained within the sequence.
C Count		The number of instances of the 'C' amino acid contained within the sequence.
Q Count		The number of instances of the 'Q' amino acid contained within the sequence.
E Count		The number of instances of the 'E' amino acid contained within the sequence.
G Count		The number of instances of the 'G' amino acid contained within the sequence.
H Count		The number of instances of the 'H' amino acid contained within the sequence.
I Count		The number of instances of the 'I' amino acid contained within the sequence.
L Count		The number of instances of the 'L' amino acid contained within the sequence.
K Count		The number of instances of the 'K' amino acid contained within the sequence.
M Count		The number of instances of the 'M' amino acid contained within the sequence.
F Count		The number of instances of the 'F' amino acid contained within the sequence.
P Count		The number of instances of the 'P' amino acid contained within the sequence.
S Count		The number of instances of the 'S' amino acid contained within the sequence.
T Count		The number of instances of the 'T' amino acid contained within the sequence.
W Count		The number of instances of the 'W' amino acid contained within the sequence.
Y Count		The number of instances of the 'Y' amino acid contained within the sequence.
V Count		The number of instances of the 'V' amino acid contained within the sequence.
U Count		The number of instances of the 'U' amino acid contained within the sequence.
O Count		The number of instances of the 'O' amino acid contained within the sequence.
Length		The length of the sequence stored in the column "Sequence".
Missed cleavages		Number of missed enzymatic cleavages.
Mass		Monoisotopic mass of the peptide.
Proteins		Identifiers of proteins this peptide is associated with.
Leading razor protein		Identifiers of the best scoring protein this peptide is associated with.
Start position		Position of the first amino acid of this peptide in the protein sequence. (one-based)
End position		Position of the last amino acid of this peptide in the protein sequence. (one-based)

Unique (Groups)		When marked with '+', this particular peptide is unique to a single protein group in the proteinGroups file.
Unique (Proteins)		When marked with '+', this particular peptide is unique to a single protein sequence in the fasta file(s).
Charges		All charge states that have been observed.
PEP		Posterior Error Probability of the identification. This value essentially operates as a p-value, where smaller is more significant.
Score		Highest Andromeda score for the associated MS/MS spectra.
Experiment OTE0019_York_060813_JH1		Number of evidence entries for this 'Experiment'.
Experiment OTE0019_York_060813_JH10		Number of evidence entries for this 'Experiment'.
Experiment OTE0019_York_060813_JH11		Number of evidence entries for this 'Experiment'.
Experiment OTE0019_York_060813_JH12		Number of evidence entries for this 'Experiment'.
Experiment OTE0019_York_060813_JH13		Number of evidence entries for this 'Experiment'.
Experiment OTE0019_York_060813_JH14		Number of evidence entries for this 'Experiment'.
Experiment OTE0019_York_060813_JH15		Number of evidence entries for this 'Experiment'.
Experiment OTE0019_York_060813_JH16		Number of evidence entries for this 'Experiment'.
Experiment OTE0019_York_060813_JH17		Number of evidence entries for this 'Experiment'.
Experiment OTE0019_York_060813_JH18		Number of evidence entries for this 'Experiment'.
Experiment OTE0019_York_060813_JH19		Number of evidence entries for this 'Experiment'.
Experiment OTE0019_York_060813_JH2		Number of evidence entries for this 'Experiment'.
Experiment OTE0019_York_060813_JH20		Number of evidence entries for this 'Experiment'.
Experiment OTE0019_York_060813_JH3		Number of evidence entries for this 'Experiment'.
Experiment OTE0019_York_060813_JH4		Number of evidence entries for this 'Experiment'.
Experiment OTE0019_York_060813_JH5		Number of evidence entries for this 'Experiment'.
Experiment OTE0019_York_060813_JH6		Number of evidence entries for this 'Experiment'.
Experiment OTE0019_York_060813_JH7		Number of evidence entries for this 'Experiment'.
Experiment OTE0019_York_060813_JH8		Number of evidence entries for this 'Experiment'.
Experiment OTE0019_York_060813_JH9		Number of evidence entries for this 'Experiment'.
Experiment QE0095_JHendy_JH1_130823142140		Number of evidence entries for this 'Experiment'.
Experiment QE0095_JHendy_JH10_130824054009		Number of evidence entries for this 'Experiment'.
Experiment QE0095_JHendy_JH11_130824082301		Number of evidence entries for this 'Experiment'.
Experiment QE0095_JHendy_JH12_130824101054		Number of evidence entries for this 'Experiment'.
Experiment QE0095_JHendy_JH13_130824115852		Number of evidence entries for this 'Experiment'.
Experiment QE0095_JHendy_JH14_130824134650		Number of evidence entries for this 'Experiment'.
Experiment QE0095_JHendy_JH15		Number of evidence entries for this 'Experiment'.
Experiment QE0095_JHendy_JH16		Number of evidence entries for this 'Experiment'.
Experiment QE0095_JHendy_JH17		Number of evidence entries for this 'Experiment'.
Experiment QE0095_JHendy_JH18		Number of evidence entries for this 'Experiment'.
Experiment QE0095_JHendy_JH19		Number of evidence entries for this 'Experiment'.
Experiment QE0095_JHendy_JH2		Number of evidence entries for this 'Experiment'.
Experiment QE0095_JHendy_JH20		Number of evidence entries for this 'Experiment'.

Experiment QE0095_JHendy_JH3_130823160937		Number of evidence entries for this 'Experiment'.
Experiment QE0095_JHendy_JH4_130823175743		Number of evidence entries for this 'Experiment'.
Experiment QE0095_JHendy_JH5_130823194539		Number of evidence entries for this 'Experiment'.
Experiment QE0095_JHendy_JH6_130823222831		Number of evidence entries for this 'Experiment'.
Experiment QE0095_JHendy_JH7_130824001623		Number of evidence entries for this 'Experiment'.
Experiment QE0095_JHendy_JH8_130824020419		Number of evidence entries for this 'Experiment'.
Experiment QE0095_JHendy_JH9		Number of evidence entries for this 'Experiment'.
Experiment QE0095_JHendy_JH9_130824035216		Number of evidence entries for this 'Experiment'.
Intensity		Summed up eXtracted Ion Current (XIC) of all isotopic clusters associated with the identified AA sequence. In case of a labeled experiment this is the total intensity of all the isotopic patterns in the label cluster.
Intensity OTE0019_York_060813_JH1		Summed up eXtracted Ion Current (XIC) of all isotopic clusters associated with the identified AA sequence. In case of a labeled experiment this is the total intensity of all the isotopic patterns in the label cluster.
Intensity OTE0019_York_060813_JH10		Summed up eXtracted Ion Current (XIC) of all isotopic clusters associated with the identified AA sequence. In case of a labeled experiment this is the total intensity of all the isotopic patterns in the label cluster.
Intensity OTE0019_York_060813_JH11		Summed up eXtracted Ion Current (XIC) of all isotopic clusters associated with the identified AA sequence. In case of a labeled experiment this is the total intensity of all the isotopic patterns in the label cluster.
Intensity OTE0019_York_060813_JH12		Summed up eXtracted Ion Current (XIC) of all isotopic clusters associated with the identified AA sequence. In case of a labeled experiment this is the total intensity of all the isotopic patterns in the label cluster.
Intensity OTE0019_York_060813_JH13		Summed up eXtracted Ion Current (XIC) of all isotopic clusters associated with the identified AA sequence. In case of a labeled experiment this is the total intensity of all the isotopic patterns in the label cluster.
Intensity OTE0019_York_060813_JH14		Summed up eXtracted Ion Current (XIC) of all isotopic clusters associated with the identified AA sequence. In case of a labeled experiment this is the total intensity of all the isotopic patterns in the label cluster.
Intensity OTE0019_York_060813_JH15		Summed up eXtracted Ion Current (XIC) of all isotopic clusters associated with the identified AA sequence. In case of a labeled experiment this is the total intensity of all the isotopic patterns in the label cluster.
Intensity OTE0019_York_060813_JH16		Summed up eXtracted Ion Current (XIC) of all isotopic clusters associated with the identified AA sequence. In case of a labeled experiment this is the total intensity of all the isotopic patterns in the label cluster.
Intensity OTE0019_York_060813_JH17		Summed up eXtracted Ion Current (XIC) of all isotopic clusters associated with the identified AA sequence. In case of a labeled experiment this is the total intensity of all the isotopic patterns in the label cluster.
Intensity OTE0019_York_060813_JH18		Summed up eXtracted Ion Current (XIC) of all isotopic clusters associated with the identified AA sequence. In case of a labeled experiment this is the total intensity of all the isotopic patterns in the label cluster.
Intensity OTE0019_York_060813_JH19		Summed up eXtracted Ion Current (XIC) of all isotopic clusters associated with the identified AA sequence. In case of a labeled experiment this is the total intensity of all the isotopic patterns in the label cluster.
Intensity OTE0019_York_060813_JH2		Summed up eXtracted Ion Current (XIC) of all isotopic clusters associated with the identified AA sequence. In case of a labeled experiment this is the total intensity of all the isotopic patterns in the label cluster.
Intensity OTE0019_York_060813_JH20		Summed up eXtracted Ion Current (XIC) of all isotopic clusters associated with the identified AA sequence. In case of a labeled experiment this is the total intensity of all the isotopic patterns in the label cluster.
Intensity OTE0019_York_060813_JH3		Summed up eXtracted Ion Current (XIC) of all isotopic clusters associated with the identified AA sequence. In case of a labeled experiment this is the total intensity of all the isotopic patterns in the label cluster.

Intensity OTE0019_York_060813_JH4		Summed up eXtracted Ion Current (XIC) of all isotopic clusters associated with the identified AA sequence. In case of a labeled experiment this is the total intensity of all the isotopic patterns in the label cluster.
Intensity OTE0019_York_060813_JH5		Summed up eXtracted Ion Current (XIC) of all isotopic clusters associated with the identified AA sequence. In case of a labeled experiment this is the total intensity of all the isotopic patterns in the label cluster.
Intensity OTE0019_York_060813_JH6		Summed up eXtracted Ion Current (XIC) of all isotopic clusters associated with the identified AA sequence. In case of a labeled experiment this is the total intensity of all the isotopic patterns in the label cluster.
Intensity OTE0019_York_060813_JH7		Summed up eXtracted Ion Current (XIC) of all isotopic clusters associated with the identified AA sequence. In case of a labeled experiment this is the total intensity of all the isotopic patterns in the label cluster.
Intensity OTE0019_York_060813_JH8		Summed up eXtracted Ion Current (XIC) of all isotopic clusters associated with the identified AA sequence. In case of a labeled experiment this is the total intensity of all the isotopic patterns in the label cluster.
Intensity OTE0019_York_060813_JH9		Summed up eXtracted Ion Current (XIC) of all isotopic clusters associated with the identified AA sequence. In case of a labeled experiment this is the total intensity of all the isotopic patterns in the label cluster.
Intensity QE0095_JHendy_JH1_130823142140		Summed up eXtracted Ion Current (XIC) of all isotopic clusters associated with the identified AA sequence. In case of a labeled experiment this is the total intensity of all the isotopic patterns in the label cluster.
Intensity QE0095_JHendy_JH10_130824054009		Summed up eXtracted Ion Current (XIC) of all isotopic clusters associated with the identified AA sequence. In case of a labeled experiment this is the total intensity of all the isotopic patterns in the label cluster.
Intensity QE0095_JHendy_JH11_130824082301		Summed up eXtracted Ion Current (XIC) of all isotopic clusters associated with the identified AA sequence. In case of a labeled experiment this is the total intensity of all the isotopic patterns in the label cluster.
Intensity QE0095_JHendy_JH12_130824101054		Summed up eXtracted Ion Current (XIC) of all isotopic clusters associated with the identified AA sequence. In case of a labeled experiment this is the total intensity of all the isotopic patterns in the label cluster.
Intensity QE0095_JHendy_JH13_130824115852		Summed up eXtracted Ion Current (XIC) of all isotopic clusters associated with the identified AA sequence. In case of a labeled experiment this is the total intensity of all the isotopic patterns in the label cluster.
Intensity QE0095_JHendy_JH14_130824134650		Summed up eXtracted Ion Current (XIC) of all isotopic clusters associated with the identified AA sequence. In case of a labeled experiment this is the total intensity of all the isotopic patterns in the label cluster.
Intensity QE0095_JHendy_JH15		Summed up eXtracted Ion Current (XIC) of all isotopic clusters associated with the identified AA sequence. In case of a labeled experiment this is the total intensity of all the isotopic patterns in the label cluster.
Intensity QE0095_JHendy_JH16		Summed up eXtracted Ion Current (XIC) of all isotopic clusters associated with the identified AA sequence. In case of a labeled experiment this is the total intensity of all the isotopic patterns in the label cluster.
Intensity QE0095_JHendy_JH17		Summed up eXtracted Ion Current (XIC) of all isotopic clusters associated with the identified AA sequence. In case of a labeled experiment this is the total intensity of all the isotopic patterns in the label cluster.
Intensity QE0095_JHendy_JH18		Summed up eXtracted Ion Current (XIC) of all isotopic clusters associated with the identified AA sequence. In case of a labeled experiment this is the total intensity of all the isotopic patterns in the label cluster.
Intensity QE0095_JHendy_JH19		Summed up eXtracted Ion Current (XIC) of all isotopic clusters associated with the identified AA sequence. In case of a labeled experiment this is the total intensity of all the isotopic patterns in the label cluster.
Intensity QE0095_JHendy_JH2		Summed up eXtracted Ion Current (XIC) of all isotopic clusters associated with the identified AA sequence. In case of a labeled experiment this is the total intensity of all the isotopic patterns in the label cluster.
Intensity QE0095_JHendy_JH20		Summed up eXtracted Ion Current (XIC) of all isotopic clusters associated with the identified AA sequence. In case of a labeled experiment this is the total intensity of all the isotopic patterns in the label cluster.
Intensity QE0095_JHendy_JH3_130823160937		Summed up eXtracted Ion Current (XIC) of all isotopic clusters associated with the identified AA sequence. In case of a labeled experiment this is the total intensity of all the isotopic patterns in the label cluster.
Intensity QE0095_JHendy_JH4_130823175743		Summed up eXtracted Ion Current (XIC) of all isotopic clusters associated with the identified AA sequence. In case of a labeled experiment this is the total intensity of all the isotopic patterns in the label cluster.

Intensity QE0095_JHendy_JH5_130823194539		Summed up eXtracted Ion Current (XIC) of all isotopic clusters associated with the identified AA sequence. In case of a labeled experiment this is the total intensity of all the isotopic patterns in the label cluster.
Intensity QE0095_JHendy_JH6_130823222831		Summed up eXtracted Ion Current (XIC) of all isotopic clusters associated with the identified AA sequence. In case of a labeled experiment this is the total intensity of all the isotopic patterns in the label cluster.
Intensity QE0095_JHendy_JH7_130824001623		Summed up eXtracted Ion Current (XIC) of all isotopic clusters associated with the identified AA sequence. In case of a labeled experiment this is the total intensity of all the isotopic patterns in the label cluster.
Intensity QE0095_JHendy_JH8_130824020419		Summed up eXtracted Ion Current (XIC) of all isotopic clusters associated with the identified AA sequence. In case of a labeled experiment this is the total intensity of all the isotopic patterns in the label cluster.
Intensity QE0095_JHendy_JH9		Summed up eXtracted Ion Current (XIC) of all isotopic clusters associated with the identified AA sequence. In case of a labeled experiment this is the total intensity of all the isotopic patterns in the label cluster.
Intensity QE0095_JHendy_JH9_130824035216		Summed up eXtracted Ion Current (XIC) of all isotopic clusters associated with the identified AA sequence. In case of a labeled experiment this is the total intensity of all the isotopic patterns in the label cluster.
Reverse		When marked with '+', this particular peptide was found to be part of a protein derived from the reversed part of the decoy database. These should be removed for further data analysis.
Potential contaminant		When marked with '+', this particular peptide was found to be part of a commonly occurring contaminant. These should be removed for further data analysis.
id		A unique (consecutive) identifier for each row in the peptides table, which is used to cross-link the information in this table with the information stored in the other tables.
Protein group IDs		The identifiers of the protein groups this peptide was linked to, referenced against the proteinGroups table.
Mod. peptide IDs		Identifier(s) for peptide sequence(s), associated with the peptide, referenced against the corresponding modified peptides table.
Evidence IDs		Identifier(s) for analyzed peptide evidence associated with the protein group referenced against the evidence table.
MS/MS IDs		The identifiers of the MS/MS scans identifying this peptide, referenced against the msms table.
Best MS/MS		The identifier of the best (in terms of quality) MS/MS scan identifying this peptide, referenced against the msms table.
Deamidation (NQ) site IDs		Identifier(s) for site(s) associated with the protein group, which show(s) evidence of the modification, referenced against the appropriate modification site file.
Hydroxyproline site IDs		Identifier(s) for site(s) associated with the protein group, which show(s) evidence of the modification, referenced against the appropriate modification site file.
MS/MS Count		

Modification-specific peptides

Name	Separator	Description
Sequence		The identified AA sequence of the peptide.
Modifications		Post-translational modifications contained within the sequence. When no modifications exist, this is set to 'unmodified'.
Mass		Charge corrected mass of the precursor ion.
Mass Fractional Part		The values after the decimal point (ie value - floor(value)).
Protein Groups		IDs of the protein groups to which this peptide belongs.
Proteins		The identifiers of the proteins this particular peptide is associated with.
Unique (Groups)		When marked with '+', this particular peptide is unique to a single protein group in the proteinGroups file.
Unique (Proteins)		When marked with '+', this particular peptide is unique to a single protein sequence in the fasta file(s).
Acetyl (Protein N-term)		Number of Acetyl (Protein N-term) on this peptide.
Deamidation (NQ)		Number of Deamidation (NQ) on this peptide.
Gln->pyro-Glu		Number of Gln->pyro-Glu on this peptide.
Glu->pyro-Glu		Number of Glu->pyro-Glu on this peptide.
Hydroxyproline		Number of Hydroxyproline on this peptide.
Oxidation (M)		Number of Oxidation (M) on this peptide.
Missed cleavages		Number of missed enzymatic cleavages.
Experiment OTE0019_York_060813_JH1		Number of evidence entries for this 'Experiment'.
Experiment OTE0019_York_060813_JH10		Number of evidence entries for this 'Experiment'.
Experiment OTE0019_York_060813_JH11		Number of evidence entries for this 'Experiment'.
Experiment OTE0019_York_060813_JH12		Number of evidence entries for this 'Experiment'.
Experiment OTE0019_York_060813_JH13		Number of evidence entries for this 'Experiment'.
Experiment OTE0019_York_060813_JH14		Number of evidence entries for this 'Experiment'.
Experiment OTE0019_York_060813_JH15		Number of evidence entries for this 'Experiment'.
Experiment OTE0019_York_060813_JH16		Number of evidence entries for this 'Experiment'.
Experiment OTE0019_York_060813_JH17		Number of evidence entries for this 'Experiment'.
Experiment OTE0019_York_060813_JH18		Number of evidence entries for this 'Experiment'.
Experiment OTE0019_York_060813_JH19		Number of evidence entries for this 'Experiment'.
Experiment OTE0019_York_060813_JH2		Number of evidence entries for this 'Experiment'.
Experiment OTE0019_York_060813_JH20		Number of evidence entries for this 'Experiment'.
Experiment OTE0019_York_060813_JH3		Number of evidence entries for this 'Experiment'.
Experiment OTE0019_York_060813_JH4		Number of evidence entries for this 'Experiment'.
Experiment OTE0019_York_060813_JH5		Number of evidence entries for this 'Experiment'.
Experiment OTE0019_York_060813_JH6		Number of evidence entries for this 'Experiment'.
Experiment OTE0019_York_060813_JH7		Number of evidence entries for this 'Experiment'.
Experiment OTE0019_York_060813_JH8		Number of evidence entries for this 'Experiment'.
Experiment OTE0019_York_060813_JH9		Number of evidence entries for this 'Experiment'.
Experiment QE0095_JHendy_JH1_130823142140		Number of evidence entries for this 'Experiment'.
Experiment QE0095_JHendy_JH10_130824054009		Number of evidence entries for this 'Experiment'.

Experiment QE0095_JHendy_JH11_130824082301		Number of evidence entries for this 'Experiment'.
Experiment QE0095_JHendy_JH12_130824101054		Number of evidence entries for this 'Experiment'.
Experiment QE0095_JHendy_JH13_130824115852		Number of evidence entries for this 'Experiment'.
Experiment QE0095_JHendy_JH14_130824134650		Number of evidence entries for this 'Experiment'.
Experiment QE0095_JHendy_JH15		Number of evidence entries for this 'Experiment'.
Experiment QE0095_JHendy_JH16		Number of evidence entries for this 'Experiment'.
Experiment QE0095_JHendy_JH17		Number of evidence entries for this 'Experiment'.
Experiment QE0095_JHendy_JH18		Number of evidence entries for this 'Experiment'.
Experiment QE0095_JHendy_JH19		Number of evidence entries for this 'Experiment'.
Experiment QE0095_JHendy_JH2		Number of evidence entries for this 'Experiment'.
Experiment QE0095_JHendy_JH20		Number of evidence entries for this 'Experiment'.
Experiment QE0095_JHendy_JH3_130823160937		Number of evidence entries for this 'Experiment'.
Experiment QE0095_JHendy_JH4_130823175743		Number of evidence entries for this 'Experiment'.
Experiment QE0095_JHendy_JH5_130823194539		Number of evidence entries for this 'Experiment'.
Experiment QE0095_JHendy_JH6_130823222831		Number of evidence entries for this 'Experiment'.
Experiment QE0095_JHendy_JH7_130824001623		Number of evidence entries for this 'Experiment'.
Experiment QE0095_JHendy_JH8_130824020419		Number of evidence entries for this 'Experiment'.
Experiment QE0095_JHendy_JH9		Number of evidence entries for this 'Experiment'.
Experiment QE0095_JHendy_JH9_130824035216		Number of evidence entries for this 'Experiment'.
Retention time		Retention time in minutes averaged over the evidence entries belonging to this modification-specific peptide.
Calibrated retention time		Calibrated retention time averaged over the evidence entries belonging to this modification-specific peptide. Obviously this only makes sense if retention time recalibration has been performed which is the case when matching between run is selected.
Charges		All charge states that have been observed.
PEP		Posterior Error Probability of the identification. This value essentially operates as a p-value, where smaller is more significant.
MS/MS scan number		The RAW-file derived scan number of the MS/MS with the highest peptide identification score (the highest score is stored in the column 'Score').
Raw file		The name of the RAW-file the mass spectral data was derived from.
Score		Andromeda score for the best identified among the associated MS/MS spectra.
Delta score		Score difference to the second best identified peptide.
Intensity		Summed up eXtracted Ion Current (XIC) of all isotopic clusters associated with the identified AA sequence. In case of a labeled experiment this is the total intensity of all the isotopic patterns in the label cluster.
Intensity OTE0019_York_060813_JH1		Summed up eXtracted Ion Current (XIC) of all isotopic clusters associated with the identified AA sequence. In case of a labeled experiment this is the total intensity of all the isotopic patterns in the label cluster.
Intensity OTE0019_York_060813_JH10		Summed up eXtracted Ion Current (XIC) of all isotopic clusters associated with the identified AA sequence. In case of a labeled experiment this is the total intensity of all the isotopic patterns in the label cluster.
Intensity OTE0019_York_060813_JH11		Summed up eXtracted Ion Current (XIC) of all isotopic clusters associated with the identified AA sequence. In case of a labeled experiment this is the total intensity of all the isotopic patterns in the label cluster.
Intensity OTE0019_York_060813_JH12		Summed up eXtracted Ion Current (XIC) of all isotopic clusters associated with the identified AA sequence. In case of a labeled experiment this is the total intensity of all the isotopic patterns in the label cluster.

Intensity OTE0019_York_060813_JH13		Summed up eXtracted Ion Current (XIC) of all isotopic clusters associated with the identified AA sequence. In case of a labeled experiment this is the total intensity of all the isotopic patterns in the label cluster.
Intensity OTE0019_York_060813_JH14		Summed up eXtracted Ion Current (XIC) of all isotopic clusters associated with the identified AA sequence. In case of a labeled experiment this is the total intensity of all the isotopic patterns in the label cluster.
Intensity OTE0019_York_060813_JH15		Summed up eXtracted Ion Current (XIC) of all isotopic clusters associated with the identified AA sequence. In case of a labeled experiment this is the total intensity of all the isotopic patterns in the label cluster.
Intensity OTE0019_York_060813_JH16		Summed up eXtracted Ion Current (XIC) of all isotopic clusters associated with the identified AA sequence. In case of a labeled experiment this is the total intensity of all the isotopic patterns in the label cluster.
Intensity OTE0019_York_060813_JH17		Summed up eXtracted Ion Current (XIC) of all isotopic clusters associated with the identified AA sequence. In case of a labeled experiment this is the total intensity of all the isotopic patterns in the label cluster.
Intensity OTE0019_York_060813_JH18		Summed up eXtracted Ion Current (XIC) of all isotopic clusters associated with the identified AA sequence. In case of a labeled experiment this is the total intensity of all the isotopic patterns in the label cluster.
Intensity OTE0019_York_060813_JH19		Summed up eXtracted Ion Current (XIC) of all isotopic clusters associated with the identified AA sequence. In case of a labeled experiment this is the total intensity of all the isotopic patterns in the label cluster.
Intensity OTE0019_York_060813_JH2		Summed up eXtracted Ion Current (XIC) of all isotopic clusters associated with the identified AA sequence. In case of a labeled experiment this is the total intensity of all the isotopic patterns in the label cluster.
Intensity OTE0019_York_060813_JH20		Summed up eXtracted Ion Current (XIC) of all isotopic clusters associated with the identified AA sequence. In case of a labeled experiment this is the total intensity of all the isotopic patterns in the label cluster.
Intensity OTE0019_York_060813_JH3		Summed up eXtracted Ion Current (XIC) of all isotopic clusters associated with the identified AA sequence. In case of a labeled experiment this is the total intensity of all the isotopic patterns in the label cluster.
Intensity OTE0019_York_060813_JH4		Summed up eXtracted Ion Current (XIC) of all isotopic clusters associated with the identified AA sequence. In case of a labeled experiment this is the total intensity of all the isotopic patterns in the label cluster.
Intensity OTE0019_York_060813_JH5		Summed up eXtracted Ion Current (XIC) of all isotopic clusters associated with the identified AA sequence. In case of a labeled experiment this is the total intensity of all the isotopic patterns in the label cluster.
Intensity OTE0019_York_060813_JH6		Summed up eXtracted Ion Current (XIC) of all isotopic clusters associated with the identified AA sequence. In case of a labeled experiment this is the total intensity of all the isotopic patterns in the label cluster.
Intensity OTE0019_York_060813_JH7		Summed up eXtracted Ion Current (XIC) of all isotopic clusters associated with the identified AA sequence. In case of a labeled experiment this is the total intensity of all the isotopic patterns in the label cluster.
Intensity OTE0019_York_060813_JH8		Summed up eXtracted Ion Current (XIC) of all isotopic clusters associated with the identified AA sequence. In case of a labeled experiment this is the total intensity of all the isotopic patterns in the label cluster.
Intensity OTE0019_York_060813_JH9		Summed up eXtracted Ion Current (XIC) of all isotopic clusters associated with the identified AA sequence. In case of a labeled experiment this is the total intensity of all the isotopic patterns in the label cluster.
Intensity QE0095_JHendy_JH1_130823142140		Summed up eXtracted Ion Current (XIC) of all isotopic clusters associated with the identified AA sequence. In case of a labeled experiment this is the total intensity of all the isotopic patterns in the label cluster.
Intensity QE0095_JHendy_JH10_130824054009		Summed up eXtracted Ion Current (XIC) of all isotopic clusters associated with the identified AA sequence. In case of a labeled experiment this is the total intensity of all the isotopic patterns in the label cluster.
Intensity QE0095_JHendy_JH11_130824082301		Summed up eXtracted Ion Current (XIC) of all isotopic clusters associated with the identified AA sequence. In case of a labeled experiment this is the total intensity of all the isotopic patterns in the label cluster.
Intensity QE0095_JHendy_JH12_130824101054		Summed up eXtracted Ion Current (XIC) of all isotopic clusters associated with the identified AA sequence. In case of a labeled experiment this is the total intensity of all the isotopic patterns in the label cluster.
Intensity QE0095_JHendy_JH13_130824115852		Summed up eXtracted Ion Current (XIC) of all isotopic clusters associated with the identified AA sequence. In case of a labeled experiment this is the total intensity of all the isotopic patterns in the label cluster.

Intensity QE0095_JHendy_JH14_130824134650		Summed up eXtracted Ion Current (XIC) of all isotopic clusters associated with the identified AA sequence. In case of a labeled experiment this is the total intensity of all the isotopic patterns in the label cluster.
Intensity QE0095_JHendy_JH15		Summed up eXtracted Ion Current (XIC) of all isotopic clusters associated with the identified AA sequence. In case of a labeled experiment this is the total intensity of all the isotopic patterns in the label cluster.
Intensity QE0095_JHendy_JH16		Summed up eXtracted Ion Current (XIC) of all isotopic clusters associated with the identified AA sequence. In case of a labeled experiment this is the total intensity of all the isotopic patterns in the label cluster.
Intensity QE0095_JHendy_JH17		Summed up eXtracted Ion Current (XIC) of all isotopic clusters associated with the identified AA sequence. In case of a labeled experiment this is the total intensity of all the isotopic patterns in the label cluster.
Intensity QE0095_JHendy_JH18		Summed up eXtracted Ion Current (XIC) of all isotopic clusters associated with the identified AA sequence. In case of a labeled experiment this is the total intensity of all the isotopic patterns in the label cluster.
Intensity QE0095_JHendy_JH19		Summed up eXtracted Ion Current (XIC) of all isotopic clusters associated with the identified AA sequence. In case of a labeled experiment this is the total intensity of all the isotopic patterns in the label cluster.
Intensity QE0095_JHendy_JH2		Summed up eXtracted Ion Current (XIC) of all isotopic clusters associated with the identified AA sequence. In case of a labeled experiment this is the total intensity of all the isotopic patterns in the label cluster.
Intensity QE0095_JHendy_JH20		Summed up eXtracted Ion Current (XIC) of all isotopic clusters associated with the identified AA sequence. In case of a labeled experiment this is the total intensity of all the isotopic patterns in the label cluster.
Intensity QE0095_JHendy_JH3_130823160937		Summed up eXtracted Ion Current (XIC) of all isotopic clusters associated with the identified AA sequence. In case of a labeled experiment this is the total intensity of all the isotopic patterns in the label cluster.
Intensity QE0095_JHendy_JH4_130823175743		Summed up eXtracted Ion Current (XIC) of all isotopic clusters associated with the identified AA sequence. In case of a labeled experiment this is the total intensity of all the isotopic patterns in the label cluster.
Intensity QE0095_JHendy_JH5_130823194539		Summed up eXtracted Ion Current (XIC) of all isotopic clusters associated with the identified AA sequence. In case of a labeled experiment this is the total intensity of all the isotopic patterns in the label cluster.
Intensity QE0095_JHendy_JH6_130823222831		Summed up eXtracted Ion Current (XIC) of all isotopic clusters associated with the identified AA sequence. In case of a labeled experiment this is the total intensity of all the isotopic patterns in the label cluster.
Intensity QE0095_JHendy_JH7_130824001623		Summed up eXtracted Ion Current (XIC) of all isotopic clusters associated with the identified AA sequence. In case of a labeled experiment this is the total intensity of all the isotopic patterns in the label cluster.
Intensity QE0095_JHendy_JH8_130824020419		Summed up eXtracted Ion Current (XIC) of all isotopic clusters associated with the identified AA sequence. In case of a labeled experiment this is the total intensity of all the isotopic patterns in the label cluster.
Intensity QE0095_JHendy_JH9		Summed up eXtracted Ion Current (XIC) of all isotopic clusters associated with the identified AA sequence. In case of a labeled experiment this is the total intensity of all the isotopic patterns in the label cluster.
Intensity QE0095_JHendy_JH9_130824035216		Summed up eXtracted Ion Current (XIC) of all isotopic clusters associated with the identified AA sequence. In case of a labeled experiment this is the total intensity of all the isotopic patterns in the label cluster.
Reverse		When marked with '+', this particular peptide was found to be part of a protein derived from the reversed part of the decoy database. These should be removed for further data analysis.
Potential contaminant		When marked with '+', this particular peptide was found to be part of a commonly occurring contaminant. These should be removed for further data analysis.
id		A unique (consecutive) identifier for each row in the peptides table, which is used to cross-link the information in this table with the information stored in the other tables.
Protein group IDs		The identifiers of the protein groups this peptide was linked to, referenced against the proteinGroups table.
Peptide ID		Identifier of the associated peptide sequence summary, which can be found in the file 'peptides.txt'.
Evidence IDs		Identifier(s) for analyzed peptide evidence associated with the protein group referenced against the evidence table.
MS/MS IDs		The identifiers of the MS/MS scans identifying this peptide, referenced against the msms table.
Best MS/MS		The identifier of the best (in terms of quality) MS/MS scan identifying this peptide, referenced against the msms table.

Deamidation (NQ) site IDs		Identifier(s) for site(s) associated with this peptide, which show(s) evidence of the modification, referenced against the appropriate modification site file.
Hydroxyproline site IDs		Identifier(s) for site(s) associated with this peptide, which show(s) evidence of the modification, referenced against the appropriate modification site file.
MS/MS Count		

Deamidation (NQ)Sites

Name	Separator	Description
Proteins		Identifiers of proteins this site is associated with.
Positions within proteins		For each protein identifier in the 'Proteins' column you find here the position of the site in the respective protein sequence. The index of the first amino acid in the sequence is 1.
Leading proteins		
Protein		Identifier of the protein this peptide is associated with.
Fasta headers		Descriptions of proteins this peptide is associated with.
Localization prob		
Score diff		
PEP		The posterior error probability (PEP) of the best identified modified peptide containing this site.
Score		The Andromeda score of the best identified modified peptide containing this site.
Delta score		The Andromeda delta score of the best identified modified peptide containing this site.
Score for localization		The Andromeda score of the MS/MS spectrum used for calculating the localization score for this site.
Localization prob OTE0019_York_060813_JH1		
Score diff OTE0019_York_060813_JH1		
PEP OTE0019_York_060813_JH1		
Score OTE0019_York_060813_JH1		
Localization prob OTE0019_York_060813_JH10		
Score diff OTE0019_York_060813_JH10		
PEP OTE0019_York_060813_JH10		
Score OTE0019_York_060813_JH10		
Localization prob OTE0019_York_060813_JH11		
Score diff OTE0019_York_060813_JH11		
PEP OTE0019_York_060813_JH11		
Score OTE0019_York_060813_JH11		
Localization prob OTE0019_York_060813_JH12		
Score diff OTE0019_York_060813_JH12		
PEP OTE0019_York_060813_JH12		
Score OTE0019_York_060813_JH12		
Localization prob OTE0019_York_060813_JH13		
Score diff OTE0019_York_060813_JH13		
PEP OTE0019_York_060813_JH13		
Score OTE0019_York_060813_JH13		
Localization prob OTE0019_York_060813_JH14		
Score diff OTE0019_York_060813_JH14		
PEP OTE0019_York_060813_JH14		
Score OTE0019_York_060813_JH14		
Localization prob OTE0019_York_060813_JH15		
Score diff OTE0019_York_060813_JH15		
PEP OTE0019_York_060813_JH15		
Score OTE0019_York_060813_JH15		

Localization prob OTE0019_York_060813_JH16		
Score diff OTE0019_York_060813_JH16		
PEP OTE0019_York_060813_JH16		
Score OTE0019_York_060813_JH16		
Localization prob OTE0019_York_060813_JH17		
Score diff OTE0019_York_060813_JH17		
PEP OTE0019_York_060813_JH17		
Score OTE0019_York_060813_JH17		
Localization prob OTE0019_York_060813_JH18		
Score diff OTE0019_York_060813_JH18		
PEP OTE0019_York_060813_JH18		
Score OTE0019_York_060813_JH18		
Localization prob OTE0019_York_060813_JH19		
Score diff OTE0019_York_060813_JH19		
PEP OTE0019_York_060813_JH19		
Score OTE0019_York_060813_JH19		
Localization prob OTE0019_York_060813_JH2		
Score diff OTE0019_York_060813_JH2		
PEP OTE0019_York_060813_JH2		
Score OTE0019_York_060813_JH2		
Localization prob OTE0019_York_060813_JH20		
Score diff OTE0019_York_060813_JH20		
PEP OTE0019_York_060813_JH20		
Score OTE0019_York_060813_JH20		
Localization prob OTE0019_York_060813_JH3		
Score diff OTE0019_York_060813_JH3		
PEP OTE0019_York_060813_JH3		
Score OTE0019_York_060813_JH3		
Localization prob OTE0019_York_060813_JH4		
Score diff OTE0019_York_060813_JH4		
PEP OTE0019_York_060813_JH4		
Score OTE0019_York_060813_JH4		
Localization prob OTE0019_York_060813_JH5		
Score diff OTE0019_York_060813_JH5		
PEP OTE0019_York_060813_JH5		
Score OTE0019_York_060813_JH5		
Localization prob OTE0019_York_060813_JH6		
Score diff OTE0019_York_060813_JH6		
PEP OTE0019_York_060813_JH6		
Score OTE0019_York_060813_JH6		
Localization prob OTE0019_York_060813_JH7		
Score diff OTE0019_York_060813_JH7		
PEP OTE0019_York_060813_JH7		
Score OTE0019_York_060813_JH7		
Localization prob OTE0019_York_060813_JH8		

Score diff OTE0019_York_060813_JH8		
PEP OTE0019_York_060813_JH8		
Score OTE0019_York_060813_JH8		
Localization prob OTE0019_York_060813_JH9		
Score diff OTE0019_York_060813_JH9		
PEP OTE0019_York_060813_JH9		
Score OTE0019_York_060813_JH9		
Localization prob QE0095_JHendy_JH1_130823142140		
Score diff QE0095_JHendy_JH1_130823142140		
PEP QE0095_JHendy_JH1_130823142140		
Score QE0095_JHendy_JH1_130823142140		
Localization prob QE0095_JHendy_JH10_130824054009		
Score diff QE0095_JHendy_JH10_130824054009		
PEP QE0095_JHendy_JH10_130824054009		
Score QE0095_JHendy_JH10_130824054009		
Localization prob QE0095_JHendy_JH11_130824082301		
Score diff QE0095_JHendy_JH11_130824082301		
PEP QE0095_JHendy_JH11_130824082301		
Score QE0095_JHendy_JH11_130824082301		
Localization prob QE0095_JHendy_JH12_130824101054		
Score diff QE0095_JHendy_JH12_130824101054		
PEP QE0095_JHendy_JH12_130824101054		
Score QE0095_JHendy_JH12_130824101054		
Localization prob QE0095_JHendy_JH13_130824115852		
Score diff QE0095_JHendy_JH13_130824115852		
PEP QE0095_JHendy_JH13_130824115852		
Score QE0095_JHendy_JH13_130824115852		
Localization prob QE0095_JHendy_JH14_130824134650		
Score diff QE0095_JHendy_JH14_130824134650		
PEP QE0095_JHendy_JH14_130824134650		

Score QE0095_JHendy_JH14_130824134650		
Localization prob QE0095_JHendy_JH15		
Score diff QE0095_JHendy_JH15		
PEP QE0095_JHendy_JH15		
Score QE0095_JHendy_JH15		
Localization prob QE0095_JHendy_JH16		
Score diff QE0095_JHendy_JH16		
PEP QE0095_JHendy_JH16		
Score QE0095_JHendy_JH16		
Localization prob QE0095_JHendy_JH17		
Score diff QE0095_JHendy_JH17		
PEP QE0095_JHendy_JH17		
Score QE0095_JHendy_JH17		
Localization prob QE0095_JHendy_JH18		
Score diff QE0095_JHendy_JH18		
PEP QE0095_JHendy_JH18		
Score QE0095_JHendy_JH18		
Localization prob QE0095_JHendy_JH19		
Score diff QE0095_JHendy_JH19		
PEP QE0095_JHendy_JH19		
Score QE0095_JHendy_JH19		
Localization prob QE0095_JHendy_JH2		
Score diff QE0095_JHendy_JH2		
PEP QE0095_JHendy_JH2		
Score QE0095_JHendy_JH2		
Localization prob QE0095_JHendy_JH20		
Score diff QE0095_JHendy_JH20		
PEP QE0095_JHendy_JH20		
Score QE0095_JHendy_JH20		
Localization prob QE0095_JHendy_JH3_130823160937		
Score diff QE0095_JHendy_JH3_130823160937		
PEP QE0095_JHendy_JH3_130823160937		
Score QE0095_JHendy_JH3_130823160937		
Localization prob QE0095_JHendy_JH4_130823175743		
Score diff QE0095_JHendy_JH4_130823175743		
PEP QE0095_JHendy_JH4_130823175743		
Score QE0095_JHendy_JH4_130823175743		
Localization prob QE0095_JHendy_JH5_130823194539		
Score diff QE0095_JHendy_JH5_130823194539		
PEP QE0095_JHendy_JH5_130823194539		
Score QE0095_JHendy_JH5_130823194539		

Localization prob QE0095_JHendy_JH6_1308232228 31		
Score diff QE0095_JHendy_JH6_1308232228 31		
PEP QE0095_JHendy_JH6_1308232228 31		
Score QE0095_JHendy_JH6_1308232228 31		
Localization prob QE0095_JHendy_JH7_1308240016 23		
Score diff QE0095_JHendy_JH7_1308240016 23		
PEP QE0095_JHendy_JH7_1308240016 23		
Score QE0095_JHendy_JH7_1308240016 23		
Localization prob QE0095_JHendy_JH8_1308240204 19		
Score diff QE0095_JHendy_JH8_1308240204 19		
PEP QE0095_JHendy_JH8_1308240204 19		
Score QE0095_JHendy_JH8_1308240204 19		
Localization prob QE0095_JHendy_JH9		
Score diff QE0095_JHendy_JH9		
PEP QE0095_JHendy_JH9		
Score QE0095_JHendy_JH9		
Localization prob QE0095_JHendy_JH9_1308240352 16		
Score diff QE0095_JHendy_JH9_1308240352 16		
PEP QE0095_JHendy_JH9_1308240352 16		
Score QE0095_JHendy_JH9_1308240352 16		
Diagnostic peak		
Number of Deamidation (NQ)		Different numbers of Deamidation (NQ) on peptides that this site is involved in.
Amino acid		
Sequence window		
Modification window		
Peptide window coverage		
Deamidation (NQ) Probabilities		
Deamidation (NQ) Score diffs		
Position in peptide		
Charge		Charge state of the precursor ion.
Mass error [ppm]		Mass error of the recalibrated mass-over-charge value of the precursor ion in comparison to the predicted monoisotopic mass of the identified peptide sequence.
Intensity		Summed up eXtracted Ion Current (XIC) of all isotopic clusters associated with the identified AA sequence. In case of a labeled experiment this is the total intensity of all the isotopic patterns in the label cluster.
Intensity___1		Summed up eXtracted Ion Current (XIC) of all isotopic clusters associated with the identified AA sequence. In case of a labeled experiment this is the total intensity of all the isotopic patterns in the label cluster.
Intensity___2		Summed up eXtracted Ion Current (XIC) of all isotopic clusters associated with the identified AA sequence. In case of a labeled experiment this is the total intensity of all the isotopic patterns in the label cluster.

Intensity___3		Summed up eXtracted Ion Current (XIC) of all isotopic clusters associated with the identified AA sequence. In case of a labeled experiment this is the total intensity of all the isotopic patterns in the label cluster.
Ratio mod/base		
Intensity OTE0019_York_060813_JH1		Summed up eXtracted Ion Current (XIC) of all isotopic clusters associated with the identified AA sequence. In case of a labeled experiment this is the total intensity of all the isotopic patterns in the label cluster.
Intensity OTE0019_York_060813_JH10		Summed up eXtracted Ion Current (XIC) of all isotopic clusters associated with the identified AA sequence. In case of a labeled experiment this is the total intensity of all the isotopic patterns in the label cluster.
Intensity OTE0019_York_060813_JH11		Summed up eXtracted Ion Current (XIC) of all isotopic clusters associated with the identified AA sequence. In case of a labeled experiment this is the total intensity of all the isotopic patterns in the label cluster.
Intensity OTE0019_York_060813_JH12		Summed up eXtracted Ion Current (XIC) of all isotopic clusters associated with the identified AA sequence. In case of a labeled experiment this is the total intensity of all the isotopic patterns in the label cluster.
Intensity OTE0019_York_060813_JH13		Summed up eXtracted Ion Current (XIC) of all isotopic clusters associated with the identified AA sequence. In case of a labeled experiment this is the total intensity of all the isotopic patterns in the label cluster.
Intensity OTE0019_York_060813_JH14		Summed up eXtracted Ion Current (XIC) of all isotopic clusters associated with the identified AA sequence. In case of a labeled experiment this is the total intensity of all the isotopic patterns in the label cluster.
Intensity OTE0019_York_060813_JH15		Summed up eXtracted Ion Current (XIC) of all isotopic clusters associated with the identified AA sequence. In case of a labeled experiment this is the total intensity of all the isotopic patterns in the label cluster.
Intensity OTE0019_York_060813_JH16		Summed up eXtracted Ion Current (XIC) of all isotopic clusters associated with the identified AA sequence. In case of a labeled experiment this is the total intensity of all the isotopic patterns in the label cluster.
Intensity OTE0019_York_060813_JH17		Summed up eXtracted Ion Current (XIC) of all isotopic clusters associated with the identified AA sequence. In case of a labeled experiment this is the total intensity of all the isotopic patterns in the label cluster.
Intensity OTE0019_York_060813_JH18		Summed up eXtracted Ion Current (XIC) of all isotopic clusters associated with the identified AA sequence. In case of a labeled experiment this is the total intensity of all the isotopic patterns in the label cluster.
Intensity OTE0019_York_060813_JH19		Summed up eXtracted Ion Current (XIC) of all isotopic clusters associated with the identified AA sequence. In case of a labeled experiment this is the total intensity of all the isotopic patterns in the label cluster.
Intensity OTE0019_York_060813_JH2		Summed up eXtracted Ion Current (XIC) of all isotopic clusters associated with the identified AA sequence. In case of a labeled experiment this is the total intensity of all the isotopic patterns in the label cluster.
Intensity OTE0019_York_060813_JH20		Summed up eXtracted Ion Current (XIC) of all isotopic clusters associated with the identified AA sequence. In case of a labeled experiment this is the total intensity of all the isotopic patterns in the label cluster.
Intensity OTE0019_York_060813_JH3		Summed up eXtracted Ion Current (XIC) of all isotopic clusters associated with the identified AA sequence. In case of a labeled experiment this is the total intensity of all the isotopic patterns in the label cluster.
Intensity OTE0019_York_060813_JH4		Summed up eXtracted Ion Current (XIC) of all isotopic clusters associated with the identified AA sequence. In case of a labeled experiment this is the total intensity of all the isotopic patterns in the label cluster.
Intensity OTE0019_York_060813_JH5		Summed up eXtracted Ion Current (XIC) of all isotopic clusters associated with the identified AA sequence. In case of a labeled experiment this is the total intensity of all the isotopic patterns in the label cluster.
Intensity OTE0019_York_060813_JH6		Summed up eXtracted Ion Current (XIC) of all isotopic clusters associated with the identified AA sequence. In case of a labeled experiment this is the total intensity of all the isotopic patterns in the label cluster.
Intensity OTE0019_York_060813_JH7		Summed up eXtracted Ion Current (XIC) of all isotopic clusters associated with the identified AA sequence. In case of a labeled experiment this is the total intensity of all the isotopic patterns in the label cluster.
Intensity OTE0019_York_060813_JH8		Summed up eXtracted Ion Current (XIC) of all isotopic clusters associated with the identified AA sequence. In case of a labeled experiment this is the total intensity of all the isotopic patterns in the label cluster.
Intensity OTE0019_York_060813_JH9		Summed up eXtracted Ion Current (XIC) of all isotopic clusters associated with the identified AA sequence. In case of a labeled experiment this is the total intensity of all the isotopic patterns in the label cluster.

Intensity QE0095_JHendy_JH1_130823142140		Summed up eXtracted Ion Current (XIC) of all isotopic clusters associated with the identified AA sequence. In case of a labeled experiment this is the total intensity of all the isotopic patterns in the label cluster.
Intensity QE0095_JHendy_JH10_130824054009		Summed up eXtracted Ion Current (XIC) of all isotopic clusters associated with the identified AA sequence. In case of a labeled experiment this is the total intensity of all the isotopic patterns in the label cluster.
Intensity QE0095_JHendy_JH11_130824082301		Summed up eXtracted Ion Current (XIC) of all isotopic clusters associated with the identified AA sequence. In case of a labeled experiment this is the total intensity of all the isotopic patterns in the label cluster.
Intensity QE0095_JHendy_JH12_130824101054		Summed up eXtracted Ion Current (XIC) of all isotopic clusters associated with the identified AA sequence. In case of a labeled experiment this is the total intensity of all the isotopic patterns in the label cluster.
Intensity QE0095_JHendy_JH13_130824115852		Summed up eXtracted Ion Current (XIC) of all isotopic clusters associated with the identified AA sequence. In case of a labeled experiment this is the total intensity of all the isotopic patterns in the label cluster.
Intensity QE0095_JHendy_JH14_130824134650		Summed up eXtracted Ion Current (XIC) of all isotopic clusters associated with the identified AA sequence. In case of a labeled experiment this is the total intensity of all the isotopic patterns in the label cluster.
Intensity QE0095_JHendy_JH15		Summed up eXtracted Ion Current (XIC) of all isotopic clusters associated with the identified AA sequence. In case of a labeled experiment this is the total intensity of all the isotopic patterns in the label cluster.
Intensity QE0095_JHendy_JH16		Summed up eXtracted Ion Current (XIC) of all isotopic clusters associated with the identified AA sequence. In case of a labeled experiment this is the total intensity of all the isotopic patterns in the label cluster.
Intensity QE0095_JHendy_JH17		Summed up eXtracted Ion Current (XIC) of all isotopic clusters associated with the identified AA sequence. In case of a labeled experiment this is the total intensity of all the isotopic patterns in the label cluster.
Intensity QE0095_JHendy_JH18		Summed up eXtracted Ion Current (XIC) of all isotopic clusters associated with the identified AA sequence. In case of a labeled experiment this is the total intensity of all the isotopic patterns in the label cluster.
Intensity QE0095_JHendy_JH19		Summed up eXtracted Ion Current (XIC) of all isotopic clusters associated with the identified AA sequence. In case of a labeled experiment this is the total intensity of all the isotopic patterns in the label cluster.
Intensity QE0095_JHendy_JH2		Summed up eXtracted Ion Current (XIC) of all isotopic clusters associated with the identified AA sequence. In case of a labeled experiment this is the total intensity of all the isotopic patterns in the label cluster.
Intensity QE0095_JHendy_JH20		Summed up eXtracted Ion Current (XIC) of all isotopic clusters associated with the identified AA sequence. In case of a labeled experiment this is the total intensity of all the isotopic patterns in the label cluster.
Intensity QE0095_JHendy_JH3_130823160937		Summed up eXtracted Ion Current (XIC) of all isotopic clusters associated with the identified AA sequence. In case of a labeled experiment this is the total intensity of all the isotopic patterns in the label cluster.
Intensity QE0095_JHendy_JH4_130823175743		Summed up eXtracted Ion Current (XIC) of all isotopic clusters associated with the identified AA sequence. In case of a labeled experiment this is the total intensity of all the isotopic patterns in the label cluster.
Intensity QE0095_JHendy_JH5_130823194539		Summed up eXtracted Ion Current (XIC) of all isotopic clusters associated with the identified AA sequence. In case of a labeled experiment this is the total intensity of all the isotopic patterns in the label cluster.
Intensity QE0095_JHendy_JH6_130823222831		Summed up eXtracted Ion Current (XIC) of all isotopic clusters associated with the identified AA sequence. In case of a labeled experiment this is the total intensity of all the isotopic patterns in the label cluster.
Intensity QE0095_JHendy_JH7_130824001623		Summed up eXtracted Ion Current (XIC) of all isotopic clusters associated with the identified AA sequence. In case of a labeled experiment this is the total intensity of all the isotopic patterns in the label cluster.
Intensity QE0095_JHendy_JH8_130824020419		Summed up eXtracted Ion Current (XIC) of all isotopic clusters associated with the identified AA sequence. In case of a labeled experiment this is the total intensity of all the isotopic patterns in the label cluster.
Intensity QE0095_JHendy_JH9		Summed up eXtracted Ion Current (XIC) of all isotopic clusters associated with the identified AA sequence. In case of a labeled experiment this is the total intensity of all the isotopic patterns in the label cluster.
Intensity QE0095_JHendy_JH9_130824035216		Summed up eXtracted Ion Current (XIC) of all isotopic clusters associated with the identified AA sequence. In case of a labeled experiment this is the total intensity of all the isotopic patterns in the label cluster.

Ratio mod/base OTE0019_York_060813_JH1		
Ratio mod/base OTE0019_York_060813_JH10		
Ratio mod/base OTE0019_York_060813_JH11		
Ratio mod/base OTE0019_York_060813_JH12		
Ratio mod/base OTE0019_York_060813_JH13		
Ratio mod/base OTE0019_York_060813_JH14		
Ratio mod/base OTE0019_York_060813_JH15		
Ratio mod/base OTE0019_York_060813_JH16		
Ratio mod/base OTE0019_York_060813_JH17		
Ratio mod/base OTE0019_York_060813_JH18		
Ratio mod/base OTE0019_York_060813_JH19		
Ratio mod/base OTE0019_York_060813_JH2		
Ratio mod/base OTE0019_York_060813_JH20		
Ratio mod/base OTE0019_York_060813_JH3		
Ratio mod/base OTE0019_York_060813_JH4		
Ratio mod/base OTE0019_York_060813_JH5		
Ratio mod/base OTE0019_York_060813_JH6		
Ratio mod/base OTE0019_York_060813_JH7		
Ratio mod/base OTE0019_York_060813_JH8		
Ratio mod/base OTE0019_York_060813_JH9		
Ratio mod/base QE0095_JHendy_JH1_130823142140		
Ratio mod/base QE0095_JHendy_JH10_130824054009		
Ratio mod/base QE0095_JHendy_JH11_130824082301		
Ratio mod/base QE0095_JHendy_JH12_130824101054		
Ratio mod/base QE0095_JHendy_JH13_130824115852		
Ratio mod/base QE0095_JHendy_JH14_130824134650		
Ratio mod/base QE0095_JHendy_JH15		
Ratio mod/base QE0095_JHendy_JH16		
Ratio mod/base QE0095_JHendy_JH17		
Ratio mod/base QE0095_JHendy_JH18		
Ratio mod/base QE0095_JHendy_JH19		
Ratio mod/base QE0095_JHendy_JH2		
Ratio mod/base QE0095_JHendy_JH20		
Ratio mod/base QE0095_JHendy_JH3_130823160937		
Ratio mod/base QE0095_JHendy_JH4_130823175743		

Ratio mod/base QE0095_JHendy_JH5_130823194539		
Ratio mod/base QE0095_JHendy_JH6_130823222831		
Ratio mod/base QE0095_JHendy_JH7_130824001623		
Ratio mod/base QE0095_JHendy_JH8_130824020419		
Ratio mod/base QE0095_JHendy_JH9		
Ratio mod/base QE0095_JHendy_JH9_130824035216		
Intensity OTE0019_York_060813_JH1___1		Summed up eXtracted Ion Current (XIC) of all isotopic clusters associated with the identified AA sequence. In case of a labeled experiment this is the total intensity of all the isotopic patterns in the label cluster.
Intensity OTE0019_York_060813_JH1___2		Summed up eXtracted Ion Current (XIC) of all isotopic clusters associated with the identified AA sequence. In case of a labeled experiment this is the total intensity of all the isotopic patterns in the label cluster.
Intensity OTE0019_York_060813_JH1___3		Summed up eXtracted Ion Current (XIC) of all isotopic clusters associated with the identified AA sequence. In case of a labeled experiment this is the total intensity of all the isotopic patterns in the label cluster.
Intensity OTE0019_York_060813_JH10___1		Summed up eXtracted Ion Current (XIC) of all isotopic clusters associated with the identified AA sequence. In case of a labeled experiment this is the total intensity of all the isotopic patterns in the label cluster.
Intensity OTE0019_York_060813_JH10___2		Summed up eXtracted Ion Current (XIC) of all isotopic clusters associated with the identified AA sequence. In case of a labeled experiment this is the total intensity of all the isotopic patterns in the label cluster.
Intensity OTE0019_York_060813_JH10___3		Summed up eXtracted Ion Current (XIC) of all isotopic clusters associated with the identified AA sequence. In case of a labeled experiment this is the total intensity of all the isotopic patterns in the label cluster.
Intensity OTE0019_York_060813_JH11___1		Summed up eXtracted Ion Current (XIC) of all isotopic clusters associated with the identified AA sequence. In case of a labeled experiment this is the total intensity of all the isotopic patterns in the label cluster.
Intensity OTE0019_York_060813_JH11___2		Summed up eXtracted Ion Current (XIC) of all isotopic clusters associated with the identified AA sequence. In case of a labeled experiment this is the total intensity of all the isotopic patterns in the label cluster.
Intensity OTE0019_York_060813_JH11___3		Summed up eXtracted Ion Current (XIC) of all isotopic clusters associated with the identified AA sequence. In case of a labeled experiment this is the total intensity of all the isotopic patterns in the label cluster.
Intensity OTE0019_York_060813_JH12___1		Summed up eXtracted Ion Current (XIC) of all isotopic clusters associated with the identified AA sequence. In case of a labeled experiment this is the total intensity of all the isotopic patterns in the label cluster.
Intensity OTE0019_York_060813_JH12___2		Summed up eXtracted Ion Current (XIC) of all isotopic clusters associated with the identified AA sequence. In case of a labeled experiment this is the total intensity of all the isotopic patterns in the label cluster.
Intensity OTE0019_York_060813_JH12___3		Summed up eXtracted Ion Current (XIC) of all isotopic clusters associated with the identified AA sequence. In case of a labeled experiment this is the total intensity of all the isotopic patterns in the label cluster.
Intensity OTE0019_York_060813_JH13___1		Summed up eXtracted Ion Current (XIC) of all isotopic clusters associated with the identified AA sequence. In case of a labeled experiment this is the total intensity of all the isotopic patterns in the label cluster.
Intensity OTE0019_York_060813_JH13___2		Summed up eXtracted Ion Current (XIC) of all isotopic clusters associated with the identified AA sequence. In case of a labeled experiment this is the total intensity of all the isotopic patterns in the label cluster.
Intensity OTE0019_York_060813_JH13___3		Summed up eXtracted Ion Current (XIC) of all isotopic clusters associated with the identified AA sequence. In case of a labeled experiment this is the total intensity of all the isotopic patterns in the label cluster.
Intensity OTE0019_York_060813_JH14___1		Summed up eXtracted Ion Current (XIC) of all isotopic clusters associated with the identified AA sequence. In case of a labeled experiment this is the total intensity of all the isotopic patterns in the label cluster.

Intensity OTE0019_York_060813_JH14___2		Summed up eXtracted Ion Current (XIC) of all isotopic clusters associated with the identified AA sequence. In case of a labeled experiment this is the total intensity of all the isotopic patterns in the label cluster.
Intensity OTE0019_York_060813_JH14___3		Summed up eXtracted Ion Current (XIC) of all isotopic clusters associated with the identified AA sequence. In case of a labeled experiment this is the total intensity of all the isotopic patterns in the label cluster.
Intensity OTE0019_York_060813_JH15___1		Summed up eXtracted Ion Current (XIC) of all isotopic clusters associated with the identified AA sequence. In case of a labeled experiment this is the total intensity of all the isotopic patterns in the label cluster.
Intensity OTE0019_York_060813_JH15___2		Summed up eXtracted Ion Current (XIC) of all isotopic clusters associated with the identified AA sequence. In case of a labeled experiment this is the total intensity of all the isotopic patterns in the label cluster.
Intensity OTE0019_York_060813_JH15___3		Summed up eXtracted Ion Current (XIC) of all isotopic clusters associated with the identified AA sequence. In case of a labeled experiment this is the total intensity of all the isotopic patterns in the label cluster.
Intensity OTE0019_York_060813_JH16___1		Summed up eXtracted Ion Current (XIC) of all isotopic clusters associated with the identified AA sequence. In case of a labeled experiment this is the total intensity of all the isotopic patterns in the label cluster.
Intensity OTE0019_York_060813_JH16___2		Summed up eXtracted Ion Current (XIC) of all isotopic clusters associated with the identified AA sequence. In case of a labeled experiment this is the total intensity of all the isotopic patterns in the label cluster.
Intensity OTE0019_York_060813_JH16___3		Summed up eXtracted Ion Current (XIC) of all isotopic clusters associated with the identified AA sequence. In case of a labeled experiment this is the total intensity of all the isotopic patterns in the label cluster.
Intensity OTE0019_York_060813_JH17___1		Summed up eXtracted Ion Current (XIC) of all isotopic clusters associated with the identified AA sequence. In case of a labeled experiment this is the total intensity of all the isotopic patterns in the label cluster.
Intensity OTE0019_York_060813_JH17___2		Summed up eXtracted Ion Current (XIC) of all isotopic clusters associated with the identified AA sequence. In case of a labeled experiment this is the total intensity of all the isotopic patterns in the label cluster.
Intensity OTE0019_York_060813_JH17___3		Summed up eXtracted Ion Current (XIC) of all isotopic clusters associated with the identified AA sequence. In case of a labeled experiment this is the total intensity of all the isotopic patterns in the label cluster.
Intensity OTE0019_York_060813_JH18___1		Summed up eXtracted Ion Current (XIC) of all isotopic clusters associated with the identified AA sequence. In case of a labeled experiment this is the total intensity of all the isotopic patterns in the label cluster.
Intensity OTE0019_York_060813_JH18___2		Summed up eXtracted Ion Current (XIC) of all isotopic clusters associated with the identified AA sequence. In case of a labeled experiment this is the total intensity of all the isotopic patterns in the label cluster.
Intensity OTE0019_York_060813_JH18___3		Summed up eXtracted Ion Current (XIC) of all isotopic clusters associated with the identified AA sequence. In case of a labeled experiment this is the total intensity of all the isotopic patterns in the label cluster.
Intensity OTE0019_York_060813_JH19___1		Summed up eXtracted Ion Current (XIC) of all isotopic clusters associated with the identified AA sequence. In case of a labeled experiment this is the total intensity of all the isotopic patterns in the label cluster.
Intensity OTE0019_York_060813_JH19___2		Summed up eXtracted Ion Current (XIC) of all isotopic clusters associated with the identified AA sequence. In case of a labeled experiment this is the total intensity of all the isotopic patterns in the label cluster.
Intensity OTE0019_York_060813_JH19___3		Summed up eXtracted Ion Current (XIC) of all isotopic clusters associated with the identified AA sequence. In case of a labeled experiment this is the total intensity of all the isotopic patterns in the label cluster.
Intensity OTE0019_York_060813_JH2___1		Summed up eXtracted Ion Current (XIC) of all isotopic clusters associated with the identified AA sequence. In case of a labeled experiment this is the total intensity of all the isotopic patterns in the label cluster.
Intensity OTE0019_York_060813_JH2___2		Summed up eXtracted Ion Current (XIC) of all isotopic clusters associated with the identified AA sequence. In case of a labeled experiment this is the total intensity of all the isotopic patterns in the label cluster.
Intensity OTE0019_York_060813_JH2___3		Summed up eXtracted Ion Current (XIC) of all isotopic clusters associated with the identified AA sequence. In case of a labeled experiment this is the total intensity of all the isotopic patterns in the label cluster.
Intensity OTE0019_York_060813_JH20___1		Summed up eXtracted Ion Current (XIC) of all isotopic clusters associated with the identified AA sequence. In case of a labeled experiment this is the total intensity of all the isotopic patterns in the label cluster.

Intensity OTE0019_York_060813_JH20___2		Summed up eXtracted Ion Current (XIC) of all isotopic clusters associated with the identified AA sequence. In case of a labeled experiment this is the total intensity of all the isotopic patterns in the label cluster.
Intensity OTE0019_York_060813_JH20___3		Summed up eXtracted Ion Current (XIC) of all isotopic clusters associated with the identified AA sequence. In case of a labeled experiment this is the total intensity of all the isotopic patterns in the label cluster.
Intensity OTE0019_York_060813_JH3___1		Summed up eXtracted Ion Current (XIC) of all isotopic clusters associated with the identified AA sequence. In case of a labeled experiment this is the total intensity of all the isotopic patterns in the label cluster.
Intensity OTE0019_York_060813_JH3___2		Summed up eXtracted Ion Current (XIC) of all isotopic clusters associated with the identified AA sequence. In case of a labeled experiment this is the total intensity of all the isotopic patterns in the label cluster.
Intensity OTE0019_York_060813_JH3___3		Summed up eXtracted Ion Current (XIC) of all isotopic clusters associated with the identified AA sequence. In case of a labeled experiment this is the total intensity of all the isotopic patterns in the label cluster.
Intensity OTE0019_York_060813_JH4___1		Summed up eXtracted Ion Current (XIC) of all isotopic clusters associated with the identified AA sequence. In case of a labeled experiment this is the total intensity of all the isotopic patterns in the label cluster.
Intensity OTE0019_York_060813_JH4___2		Summed up eXtracted Ion Current (XIC) of all isotopic clusters associated with the identified AA sequence. In case of a labeled experiment this is the total intensity of all the isotopic patterns in the label cluster.
Intensity OTE0019_York_060813_JH4___3		Summed up eXtracted Ion Current (XIC) of all isotopic clusters associated with the identified AA sequence. In case of a labeled experiment this is the total intensity of all the isotopic patterns in the label cluster.
Intensity OTE0019_York_060813_JH5___1		Summed up eXtracted Ion Current (XIC) of all isotopic clusters associated with the identified AA sequence. In case of a labeled experiment this is the total intensity of all the isotopic patterns in the label cluster.
Intensity OTE0019_York_060813_JH5___2		Summed up eXtracted Ion Current (XIC) of all isotopic clusters associated with the identified AA sequence. In case of a labeled experiment this is the total intensity of all the isotopic patterns in the label cluster.
Intensity OTE0019_York_060813_JH5___3		Summed up eXtracted Ion Current (XIC) of all isotopic clusters associated with the identified AA sequence. In case of a labeled experiment this is the total intensity of all the isotopic patterns in the label cluster.
Intensity OTE0019_York_060813_JH6___1		Summed up eXtracted Ion Current (XIC) of all isotopic clusters associated with the identified AA sequence. In case of a labeled experiment this is the total intensity of all the isotopic patterns in the label cluster.
Intensity OTE0019_York_060813_JH6___2		Summed up eXtracted Ion Current (XIC) of all isotopic clusters associated with the identified AA sequence. In case of a labeled experiment this is the total intensity of all the isotopic patterns in the label cluster.
Intensity OTE0019_York_060813_JH6___3		Summed up eXtracted Ion Current (XIC) of all isotopic clusters associated with the identified AA sequence. In case of a labeled experiment this is the total intensity of all the isotopic patterns in the label cluster.
Intensity OTE0019_York_060813_JH7___1		Summed up eXtracted Ion Current (XIC) of all isotopic clusters associated with the identified AA sequence. In case of a labeled experiment this is the total intensity of all the isotopic patterns in the label cluster.
Intensity OTE0019_York_060813_JH7___2		Summed up eXtracted Ion Current (XIC) of all isotopic clusters associated with the identified AA sequence. In case of a labeled experiment this is the total intensity of all the isotopic patterns in the label cluster.
Intensity OTE0019_York_060813_JH7___3		Summed up eXtracted Ion Current (XIC) of all isotopic clusters associated with the identified AA sequence. In case of a labeled experiment this is the total intensity of all the isotopic patterns in the label cluster.
Intensity OTE0019_York_060813_JH8___1		Summed up eXtracted Ion Current (XIC) of all isotopic clusters associated with the identified AA sequence. In case of a labeled experiment this is the total intensity of all the isotopic patterns in the label cluster.
Intensity OTE0019_York_060813_JH8___2		Summed up eXtracted Ion Current (XIC) of all isotopic clusters associated with the identified AA sequence. In case of a labeled experiment this is the total intensity of all the isotopic patterns in the label cluster.
Intensity OTE0019_York_060813_JH8___3		Summed up eXtracted Ion Current (XIC) of all isotopic clusters associated with the identified AA sequence. In case of a labeled experiment this is the total intensity of all the isotopic patterns in the label cluster.
Intensity OTE0019_York_060813_JH9___1		Summed up eXtracted Ion Current (XIC) of all isotopic clusters associated with the identified AA sequence. In case of a labeled experiment this is the total intensity of all the isotopic patterns in the label cluster.

Intensity OTE0019_York_060813_JH9___2		Summed up eXtracted Ion Current (XIC) of all isotopic clusters associated with the identified AA sequence. In case of a labeled experiment this is the total intensity of all the isotopic patterns in the label cluster.
Intensity OTE0019_York_060813_JH9___3		Summed up eXtracted Ion Current (XIC) of all isotopic clusters associated with the identified AA sequence. In case of a labeled experiment this is the total intensity of all the isotopic patterns in the label cluster.
Intensity QE0095_JHendy_JH1_130823142140___1		Summed up eXtracted Ion Current (XIC) of all isotopic clusters associated with the identified AA sequence. In case of a labeled experiment this is the total intensity of all the isotopic patterns in the label cluster.
Intensity QE0095_JHendy_JH1_130823142140___2		Summed up eXtracted Ion Current (XIC) of all isotopic clusters associated with the identified AA sequence. In case of a labeled experiment this is the total intensity of all the isotopic patterns in the label cluster.
Intensity QE0095_JHendy_JH1_130823142140___3		Summed up eXtracted Ion Current (XIC) of all isotopic clusters associated with the identified AA sequence. In case of a labeled experiment this is the total intensity of all the isotopic patterns in the label cluster.
Intensity QE0095_JHendy_JH10_130824054009___1		Summed up eXtracted Ion Current (XIC) of all isotopic clusters associated with the identified AA sequence. In case of a labeled experiment this is the total intensity of all the isotopic patterns in the label cluster.
Intensity QE0095_JHendy_JH10_130824054009___2		Summed up eXtracted Ion Current (XIC) of all isotopic clusters associated with the identified AA sequence. In case of a labeled experiment this is the total intensity of all the isotopic patterns in the label cluster.
Intensity QE0095_JHendy_JH10_130824054009___3		Summed up eXtracted Ion Current (XIC) of all isotopic clusters associated with the identified AA sequence. In case of a labeled experiment this is the total intensity of all the isotopic patterns in the label cluster.
Intensity QE0095_JHendy_JH11_130824082301___1		Summed up eXtracted Ion Current (XIC) of all isotopic clusters associated with the identified AA sequence. In case of a labeled experiment this is the total intensity of all the isotopic patterns in the label cluster.
Intensity QE0095_JHendy_JH11_130824082301___2		Summed up eXtracted Ion Current (XIC) of all isotopic clusters associated with the identified AA sequence. In case of a labeled experiment this is the total intensity of all the isotopic patterns in the label cluster.
Intensity QE0095_JHendy_JH11_130824082301___3		Summed up eXtracted Ion Current (XIC) of all isotopic clusters associated with the identified AA sequence. In case of a labeled experiment this is the total intensity of all the isotopic patterns in the label cluster.
Intensity QE0095_JHendy_JH12_130824101054___1		Summed up eXtracted Ion Current (XIC) of all isotopic clusters associated with the identified AA sequence. In case of a labeled experiment this is the total intensity of all the isotopic patterns in the label cluster.
Intensity QE0095_JHendy_JH12_130824101054___2		Summed up eXtracted Ion Current (XIC) of all isotopic clusters associated with the identified AA sequence. In case of a labeled experiment this is the total intensity of all the isotopic patterns in the label cluster.
Intensity QE0095_JHendy_JH12_130824101054___3		Summed up eXtracted Ion Current (XIC) of all isotopic clusters associated with the identified AA sequence. In case of a labeled experiment this is the total intensity of all the isotopic patterns in the label cluster.
Intensity QE0095_JHendy_JH13_130824115852___1		Summed up eXtracted Ion Current (XIC) of all isotopic clusters associated with the identified AA sequence. In case of a labeled experiment this is the total intensity of all the isotopic patterns in the label cluster.
Intensity QE0095_JHendy_JH13_130824115852___2		Summed up eXtracted Ion Current (XIC) of all isotopic clusters associated with the identified AA sequence. In case of a labeled experiment this is the total intensity of all the isotopic patterns in the label cluster.
Intensity QE0095_JHendy_JH13_130824115852___3		Summed up eXtracted Ion Current (XIC) of all isotopic clusters associated with the identified AA sequence. In case of a labeled experiment this is the total intensity of all the isotopic patterns in the label cluster.
Intensity QE0095_JHendy_JH14_130824134650___1		Summed up eXtracted Ion Current (XIC) of all isotopic clusters associated with the identified AA sequence. In case of a labeled experiment this is the total intensity of all the isotopic patterns in the label cluster.
Intensity QE0095_JHendy_JH14_130824134650___2		Summed up eXtracted Ion Current (XIC) of all isotopic clusters associated with the identified AA sequence. In case of a labeled experiment this is the total intensity of all the isotopic patterns in the label cluster.
Intensity QE0095_JHendy_JH14_130824134650___3		Summed up eXtracted Ion Current (XIC) of all isotopic clusters associated with the identified AA sequence. In case of a labeled experiment this is the total intensity of all the isotopic patterns in the label cluster.
Intensity QE0095_JHendy_JH15___1		Summed up eXtracted Ion Current (XIC) of all isotopic clusters associated with the identified AA sequence. In case of a labeled experiment this is the total intensity of all the isotopic patterns in the label cluster.

Intensity QE0095_JHendy_JH15___2		Summed up eXtracted Ion Current (XIC) of all isotopic clusters associated with the identified AA sequence. In case of a labeled experiment this is the total intensity of all the isotopic patterns in the label cluster.
Intensity QE0095_JHendy_JH15___3		Summed up eXtracted Ion Current (XIC) of all isotopic clusters associated with the identified AA sequence. In case of a labeled experiment this is the total intensity of all the isotopic patterns in the label cluster.
Intensity QE0095_JHendy_JH16___1		Summed up eXtracted Ion Current (XIC) of all isotopic clusters associated with the identified AA sequence. In case of a labeled experiment this is the total intensity of all the isotopic patterns in the label cluster.
Intensity QE0095_JHendy_JH16___2		Summed up eXtracted Ion Current (XIC) of all isotopic clusters associated with the identified AA sequence. In case of a labeled experiment this is the total intensity of all the isotopic patterns in the label cluster.
Intensity QE0095_JHendy_JH16___3		Summed up eXtracted Ion Current (XIC) of all isotopic clusters associated with the identified AA sequence. In case of a labeled experiment this is the total intensity of all the isotopic patterns in the label cluster.
Intensity QE0095_JHendy_JH17___1		Summed up eXtracted Ion Current (XIC) of all isotopic clusters associated with the identified AA sequence. In case of a labeled experiment this is the total intensity of all the isotopic patterns in the label cluster.
Intensity QE0095_JHendy_JH17___2		Summed up eXtracted Ion Current (XIC) of all isotopic clusters associated with the identified AA sequence. In case of a labeled experiment this is the total intensity of all the isotopic patterns in the label cluster.
Intensity QE0095_JHendy_JH17___3		Summed up eXtracted Ion Current (XIC) of all isotopic clusters associated with the identified AA sequence. In case of a labeled experiment this is the total intensity of all the isotopic patterns in the label cluster.
Intensity QE0095_JHendy_JH18___1		Summed up eXtracted Ion Current (XIC) of all isotopic clusters associated with the identified AA sequence. In case of a labeled experiment this is the total intensity of all the isotopic patterns in the label cluster.
Intensity QE0095_JHendy_JH18___2		Summed up eXtracted Ion Current (XIC) of all isotopic clusters associated with the identified AA sequence. In case of a labeled experiment this is the total intensity of all the isotopic patterns in the label cluster.
Intensity QE0095_JHendy_JH18___3		Summed up eXtracted Ion Current (XIC) of all isotopic clusters associated with the identified AA sequence. In case of a labeled experiment this is the total intensity of all the isotopic patterns in the label cluster.
Intensity QE0095_JHendy_JH19___1		Summed up eXtracted Ion Current (XIC) of all isotopic clusters associated with the identified AA sequence. In case of a labeled experiment this is the total intensity of all the isotopic patterns in the label cluster.
Intensity QE0095_JHendy_JH19___2		Summed up eXtracted Ion Current (XIC) of all isotopic clusters associated with the identified AA sequence. In case of a labeled experiment this is the total intensity of all the isotopic patterns in the label cluster.
Intensity QE0095_JHendy_JH19___3		Summed up eXtracted Ion Current (XIC) of all isotopic clusters associated with the identified AA sequence. In case of a labeled experiment this is the total intensity of all the isotopic patterns in the label cluster.
Intensity QE0095_JHendy_JH2___1		Summed up eXtracted Ion Current (XIC) of all isotopic clusters associated with the identified AA sequence. In case of a labeled experiment this is the total intensity of all the isotopic patterns in the label cluster.
Intensity QE0095_JHendy_JH2___2		Summed up eXtracted Ion Current (XIC) of all isotopic clusters associated with the identified AA sequence. In case of a labeled experiment this is the total intensity of all the isotopic patterns in the label cluster.
Intensity QE0095_JHendy_JH2___3		Summed up eXtracted Ion Current (XIC) of all isotopic clusters associated with the identified AA sequence. In case of a labeled experiment this is the total intensity of all the isotopic patterns in the label cluster.
Intensity QE0095_JHendy_JH20___1		Summed up eXtracted Ion Current (XIC) of all isotopic clusters associated with the identified AA sequence. In case of a labeled experiment this is the total intensity of all the isotopic patterns in the label cluster.
Intensity QE0095_JHendy_JH20___2		Summed up eXtracted Ion Current (XIC) of all isotopic clusters associated with the identified AA sequence. In case of a labeled experiment this is the total intensity of all the isotopic patterns in the label cluster.
Intensity QE0095_JHendy_JH20___3		Summed up eXtracted Ion Current (XIC) of all isotopic clusters associated with the identified AA sequence. In case of a labeled experiment this is the total intensity of all the isotopic patterns in the label cluster.
Intensity QE0095_JHendy_JH3_130823160937___1		Summed up eXtracted Ion Current (XIC) of all isotopic clusters associated with the identified AA sequence. In case of a labeled experiment this is the total intensity of all the isotopic patterns in the label cluster.

Intensity QE0095_JHendy_JH3_130823160937___2		Summed up eXtracted Ion Current (XIC) of all isotopic clusters associated with the identified AA sequence. In case of a labeled experiment this is the total intensity of all the isotopic patterns in the label cluster.
Intensity QE0095_JHendy_JH3_130823160937___3		Summed up eXtracted Ion Current (XIC) of all isotopic clusters associated with the identified AA sequence. In case of a labeled experiment this is the total intensity of all the isotopic patterns in the label cluster.
Intensity QE0095_JHendy_JH4_130823175743___1		Summed up eXtracted Ion Current (XIC) of all isotopic clusters associated with the identified AA sequence. In case of a labeled experiment this is the total intensity of all the isotopic patterns in the label cluster.
Intensity QE0095_JHendy_JH4_130823175743___2		Summed up eXtracted Ion Current (XIC) of all isotopic clusters associated with the identified AA sequence. In case of a labeled experiment this is the total intensity of all the isotopic patterns in the label cluster.
Intensity QE0095_JHendy_JH4_130823175743___3		Summed up eXtracted Ion Current (XIC) of all isotopic clusters associated with the identified AA sequence. In case of a labeled experiment this is the total intensity of all the isotopic patterns in the label cluster.
Intensity QE0095_JHendy_JH5_130823194539___1		Summed up eXtracted Ion Current (XIC) of all isotopic clusters associated with the identified AA sequence. In case of a labeled experiment this is the total intensity of all the isotopic patterns in the label cluster.
Intensity QE0095_JHendy_JH5_130823194539___2		Summed up eXtracted Ion Current (XIC) of all isotopic clusters associated with the identified AA sequence. In case of a labeled experiment this is the total intensity of all the isotopic patterns in the label cluster.
Intensity QE0095_JHendy_JH5_130823194539___3		Summed up eXtracted Ion Current (XIC) of all isotopic clusters associated with the identified AA sequence. In case of a labeled experiment this is the total intensity of all the isotopic patterns in the label cluster.
Intensity QE0095_JHendy_JH6_130823222831___1		Summed up eXtracted Ion Current (XIC) of all isotopic clusters associated with the identified AA sequence. In case of a labeled experiment this is the total intensity of all the isotopic patterns in the label cluster.
Intensity QE0095_JHendy_JH6_130823222831___2		Summed up eXtracted Ion Current (XIC) of all isotopic clusters associated with the identified AA sequence. In case of a labeled experiment this is the total intensity of all the isotopic patterns in the label cluster.
Intensity QE0095_JHendy_JH6_130823222831___3		Summed up eXtracted Ion Current (XIC) of all isotopic clusters associated with the identified AA sequence. In case of a labeled experiment this is the total intensity of all the isotopic patterns in the label cluster.
Intensity QE0095_JHendy_JH7_130824001623___1		Summed up eXtracted Ion Current (XIC) of all isotopic clusters associated with the identified AA sequence. In case of a labeled experiment this is the total intensity of all the isotopic patterns in the label cluster.
Intensity QE0095_JHendy_JH7_130824001623___2		Summed up eXtracted Ion Current (XIC) of all isotopic clusters associated with the identified AA sequence. In case of a labeled experiment this is the total intensity of all the isotopic patterns in the label cluster.
Intensity QE0095_JHendy_JH7_130824001623___3		Summed up eXtracted Ion Current (XIC) of all isotopic clusters associated with the identified AA sequence. In case of a labeled experiment this is the total intensity of all the isotopic patterns in the label cluster.
Intensity QE0095_JHendy_JH8_130824020419___1		Summed up eXtracted Ion Current (XIC) of all isotopic clusters associated with the identified AA sequence. In case of a labeled experiment this is the total intensity of all the isotopic patterns in the label cluster.
Intensity QE0095_JHendy_JH8_130824020419___2		Summed up eXtracted Ion Current (XIC) of all isotopic clusters associated with the identified AA sequence. In case of a labeled experiment this is the total intensity of all the isotopic patterns in the label cluster.
Intensity QE0095_JHendy_JH8_130824020419___3		Summed up eXtracted Ion Current (XIC) of all isotopic clusters associated with the identified AA sequence. In case of a labeled experiment this is the total intensity of all the isotopic patterns in the label cluster.
Intensity QE0095_JHendy_JH9___1		Summed up eXtracted Ion Current (XIC) of all isotopic clusters associated with the identified AA sequence. In case of a labeled experiment this is the total intensity of all the isotopic patterns in the label cluster.
Intensity QE0095_JHendy_JH9___2		Summed up eXtracted Ion Current (XIC) of all isotopic clusters associated with the identified AA sequence. In case of a labeled experiment this is the total intensity of all the isotopic patterns in the label cluster.
Intensity QE0095_JHendy_JH9___3		Summed up eXtracted Ion Current (XIC) of all isotopic clusters associated with the identified AA sequence. In case of a labeled experiment this is the total intensity of all the isotopic patterns in the label cluster.
Intensity QE0095_JHendy_JH9_130824035216___1		Summed up eXtracted Ion Current (XIC) of all isotopic clusters associated with the identified AA sequence. In case of a labeled experiment this is the total intensity of all the isotopic patterns in the label cluster.

Intensity QE0095_JHendy_JH9_1308240352 16___2		Summed up eXtracted Ion Current (XIC) of all isotopic clusters associated with the identified AA sequence. In case of a labeled experiment this is the total intensity of all the isotopic patterns in the label cluster.
Intensity QE0095_JHendy_JH9_1308240352 16___3		Summed up eXtracted Ion Current (XIC) of all isotopic clusters associated with the identified AA sequence. In case of a labeled experiment this is the total intensity of all the isotopic patterns in the label cluster.
Occupancy OTE0019_York_060813_JH1		
Occupancy ratioOTE0019_York_060813_JH1		
Occupancy error scale OTE0019_York_060813_JH1		
Occupancy OTE0019_York_060813_JH10		
Occupancy ratioOTE0019_York_060813_JH10		
Occupancy error scale OTE0019_York_060813_JH10		
Occupancy OTE0019_York_060813_JH11		
Occupancy ratioOTE0019_York_060813_JH11		
Occupancy error scale OTE0019_York_060813_JH11		
Occupancy OTE0019_York_060813_JH12		
Occupancy ratioOTE0019_York_060813_JH12		
Occupancy error scale OTE0019_York_060813_JH12		
Occupancy OTE0019_York_060813_JH13		
Occupancy ratioOTE0019_York_060813_JH13		
Occupancy error scale OTE0019_York_060813_JH13		
Occupancy OTE0019_York_060813_JH14		
Occupancy ratioOTE0019_York_060813_JH14		
Occupancy error scale OTE0019_York_060813_JH14		
Occupancy OTE0019_York_060813_JH15		
Occupancy ratioOTE0019_York_060813_JH15		
Occupancy error scale OTE0019_York_060813_JH15		
Occupancy OTE0019_York_060813_JH16		
Occupancy ratioOTE0019_York_060813_JH16		
Occupancy error scale OTE0019_York_060813_JH16		
Occupancy OTE0019_York_060813_JH17		
Occupancy ratioOTE0019_York_060813_JH17		
Occupancy error scale OTE0019_York_060813_JH17		
Occupancy OTE0019_York_060813_JH18		
Occupancy ratioOTE0019_York_060813_JH18		
Occupancy error scale OTE0019_York_060813_JH18		
Occupancy OTE0019_York_060813_JH19		
Occupancy ratioOTE0019_York_060813_JH19		
Occupancy error scale OTE0019_York_060813_JH19		
Occupancy OTE0019_York_060813_JH2		

Occupancy ratioOTE0019_York_060813_JH2		
Occupancy error scale OTE0019_York_060813_JH2		
Occupancy OTE0019_York_060813_JH20		
Occupancy ratioOTE0019_York_060813_JH20		
Occupancy error scale OTE0019_York_060813_JH20		
Occupancy OTE0019_York_060813_JH3		
Occupancy ratioOTE0019_York_060813_JH3		
Occupancy error scale OTE0019_York_060813_JH3		
Occupancy OTE0019_York_060813_JH4		
Occupancy ratioOTE0019_York_060813_JH4		
Occupancy error scale OTE0019_York_060813_JH4		
Occupancy OTE0019_York_060813_JH5		
Occupancy ratioOTE0019_York_060813_JH5		
Occupancy error scale OTE0019_York_060813_JH5		
Occupancy OTE0019_York_060813_JH6		
Occupancy ratioOTE0019_York_060813_JH6		
Occupancy error scale OTE0019_York_060813_JH6		
Occupancy OTE0019_York_060813_JH7		
Occupancy ratioOTE0019_York_060813_JH7		
Occupancy error scale OTE0019_York_060813_JH7		
Occupancy OTE0019_York_060813_JH8		
Occupancy ratioOTE0019_York_060813_JH8		
Occupancy error scale OTE0019_York_060813_JH8		
Occupancy OTE0019_York_060813_JH9		
Occupancy ratioOTE0019_York_060813_JH9		
Occupancy error scale OTE0019_York_060813_JH9		
Occupancy QE0095_JHendy_JH1_130823142140		
Occupancy ratioQE0095_JHendy_JH1_130823142140		
Occupancy error scale QE0095_JHendy_JH1_130823142140		
Occupancy QE0095_JHendy_JH10_130824054009		
Occupancy ratioQE0095_JHendy_JH10_130824054009		
Occupancy error scale QE0095_JHendy_JH10_130824054009		
Occupancy QE0095_JHendy_JH11_130824082301		
Occupancy ratioQE0095_JHendy_JH11_130824082301		

Occupancy error scale QE0095_JHendy_JH11_130824082301		
Occupancy QE0095_JHendy_JH12_130824101054		
Occupancy ratioQE0095_JHendy_JH12_130824101054		
Occupancy error scale QE0095_JHendy_JH12_130824101054		
Occupancy QE0095_JHendy_JH13_130824115852		
Occupancy ratioQE0095_JHendy_JH13_130824115852		
Occupancy error scale QE0095_JHendy_JH13_130824115852		
Occupancy QE0095_JHendy_JH14_130824134650		
Occupancy ratioQE0095_JHendy_JH14_130824134650		
Occupancy error scale QE0095_JHendy_JH14_130824134650		
Occupancy QE0095_JHendy_JH15		
Occupancy ratioQE0095_JHendy_JH15		
Occupancy error scale QE0095_JHendy_JH15		
Occupancy QE0095_JHendy_JH16		
Occupancy ratioQE0095_JHendy_JH16		
Occupancy error scale QE0095_JHendy_JH16		
Occupancy QE0095_JHendy_JH17		
Occupancy ratioQE0095_JHendy_JH17		
Occupancy error scale QE0095_JHendy_JH17		
Occupancy QE0095_JHendy_JH18		
Occupancy ratioQE0095_JHendy_JH18		
Occupancy error scale QE0095_JHendy_JH18		
Occupancy QE0095_JHendy_JH19		
Occupancy ratioQE0095_JHendy_JH19		
Occupancy error scale QE0095_JHendy_JH19		
Occupancy QE0095_JHendy_JH2		
Occupancy ratioQE0095_JHendy_JH2		
Occupancy error scale QE0095_JHendy_JH2		
Occupancy QE0095_JHendy_JH20		
Occupancy ratioQE0095_JHendy_JH20		
Occupancy error scale QE0095_JHendy_JH20		
Occupancy QE0095_JHendy_JH3_130823160937		
Occupancy ratioQE0095_JHendy_JH3_130823160937		
Occupancy error scale QE0095_JHendy_JH3_130823160937		
Occupancy QE0095_JHendy_JH4_130823175743		

Occupancy ratioQE0095_JHendy_JH4_130823 175743		
Occupancy error scale QE0095_JHendy_JH4_1308231757 43		
Occupancy QE0095_JHendy_JH5_1308231945 39		
Occupancy ratioQE0095_JHendy_JH5_130823 194539		
Occupancy error scale QE0095_JHendy_JH5_1308231945 39		
Occupancy QE0095_JHendy_JH6_1308232228 31		
Occupancy ratioQE0095_JHendy_JH6_130823 222831		
Occupancy error scale QE0095_JHendy_JH6_1308232228 31		
Occupancy QE0095_JHendy_JH7_1308240016 23		
Occupancy ratioQE0095_JHendy_JH7_130824 001623		
Occupancy error scale QE0095_JHendy_JH7_1308240016 23		
Occupancy QE0095_JHendy_JH8_1308240204 19		
Occupancy ratioQE0095_JHendy_JH8_130824 020419		
Occupancy error scale QE0095_JHendy_JH8_1308240204 19		
Occupancy QE0095_JHendy_JH9		
Occupancy ratioQE0095_JHendy_JH9		
Occupancy error scale QE0095_JHendy_JH9		
Occupancy QE0095_JHendy_JH9_1308240352 16		
Occupancy ratioQE0095_JHendy_JH9_130824 035216		
Occupancy error scale QE0095_JHendy_JH9_1308240352 16		
Reverse		When marked with '+', this particular peptide was found to be part of a protein derived from the reversed part of the protein sequence database. These should be removed for further data analysis.
Potential contaminant		When marked with '+', this particular peptide was found to be part of a commonly occurring contaminant. These should be removed for further data analysis.
id		A unique (consecutive) identifier for each row in the site table, which is used to cross-link the information in this file with the information stored in the other files.
Protein group IDs		The identifier of the protein-group this peptide sequence is associated with, which can be used to look up the extended protein information in the file 'proteinGroups.txt'.<br>As a single peptide can be linked to multiple proteins (e.g. in the case of razor-proteins), multiple id’s can be stored here separated by a semicolon.<br>As a protein can be identified by multiple peptides, the same id can be found in different rows.
Positions		The positions of the modifications in the protein amino acid sequence.
Position		The position of the modification in the protein amino acid sequence.
Peptide IDs		Identifier(s) of the associated peptide sequence(s) summary, which can be found in the file 'peptides.txt'.
Mod. peptide IDs		Identifier(s) of the associated peptide sequence(s) summary, which can be found in the file 'modificationSpecificPeptides.txt'.

Evidence IDs		Identifier(s) for analyzed peptide evidence associated with the protein group referenced against the evidence table.
MS/MS IDs		The identifiers of the MS/MS scans identifying this peptide, referenced against the msms table.
Best localization evidence ID		
Best localization MS/MS ID		
Best localization raw file		
Best localization scan number		
Best score evidence ID		
Best score MS/MS ID		
Best score raw file		
Best score scan number		
Best PEP evidence ID		
Best PEP MS/MS ID		
Best PEP raw file		
Best PEP scan number		

HydroxyprolineSites

Name	Separator	Description
Proteins		Identifiers of proteins this site is associated with.
Positions within proteins		For each protein identifier in the 'Proteins' column you find here the position of the site in the respective protein sequence. The index of the first amino acid in the sequence is 1.
Leading proteins		
Protein		Identifier of the protein this peptide is associated with.
Fasta headers		Descriptions of proteins this peptide is associated with.
Localization prob		
Score diff		
PEP		The posterior error probability (PEP) of the best identified modified peptide containing this site.
Score		The Andromeda score of the best identified modified peptide containing this site.
Delta score		The Andromeda delta score of the best identified modified peptide containing this site.
Score for localization		The Andromeda score of the MS/MS spectrum used for calculating the localization score for this site.
Localization prob OTE0019_York_060813_JH1		
Score diff OTE0019_York_060813_JH1		
PEP OTE0019_York_060813_JH1		
Score OTE0019_York_060813_JH1		
Localization prob OTE0019_York_060813_JH10		
Score diff OTE0019_York_060813_JH10		
PEP OTE0019_York_060813_JH10		
Score OTE0019_York_060813_JH10		
Localization prob OTE0019_York_060813_JH11		
Score diff OTE0019_York_060813_JH11		
PEP OTE0019_York_060813_JH11		
Score OTE0019_York_060813_JH11		
Localization prob OTE0019_York_060813_JH12		
Score diff OTE0019_York_060813_JH12		
PEP OTE0019_York_060813_JH12		
Score OTE0019_York_060813_JH12		
Localization prob OTE0019_York_060813_JH13		
Score diff OTE0019_York_060813_JH13		
PEP OTE0019_York_060813_JH13		
Score OTE0019_York_060813_JH13		
Localization prob OTE0019_York_060813_JH14		
Score diff OTE0019_York_060813_JH14		
PEP OTE0019_York_060813_JH14		
Score OTE0019_York_060813_JH14		
Localization prob OTE0019_York_060813_JH15		
Score diff OTE0019_York_060813_JH15		
PEP OTE0019_York_060813_JH15		
Score OTE0019_York_060813_JH15		

Localization prob OTE0019_York_060813_JH16		
Score diff OTE0019_York_060813_JH16		
PEP OTE0019_York_060813_JH16		
Score OTE0019_York_060813_JH16		
Localization prob OTE0019_York_060813_JH17		
Score diff OTE0019_York_060813_JH17		
PEP OTE0019_York_060813_JH17		
Score OTE0019_York_060813_JH17		
Localization prob OTE0019_York_060813_JH18		
Score diff OTE0019_York_060813_JH18		
PEP OTE0019_York_060813_JH18		
Score OTE0019_York_060813_JH18		
Localization prob OTE0019_York_060813_JH19		
Score diff OTE0019_York_060813_JH19		
PEP OTE0019_York_060813_JH19		
Score OTE0019_York_060813_JH19		
Localization prob OTE0019_York_060813_JH2		
Score diff OTE0019_York_060813_JH2		
PEP OTE0019_York_060813_JH2		
Score OTE0019_York_060813_JH2		
Localization prob OTE0019_York_060813_JH20		
Score diff OTE0019_York_060813_JH20		
PEP OTE0019_York_060813_JH20		
Score OTE0019_York_060813_JH20		
Localization prob OTE0019_York_060813_JH3		
Score diff OTE0019_York_060813_JH3		
PEP OTE0019_York_060813_JH3		
Score OTE0019_York_060813_JH3		
Localization prob OTE0019_York_060813_JH4		
Score diff OTE0019_York_060813_JH4		
PEP OTE0019_York_060813_JH4		
Score OTE0019_York_060813_JH4		
Localization prob OTE0019_York_060813_JH5		
Score diff OTE0019_York_060813_JH5		
PEP OTE0019_York_060813_JH5		
Score OTE0019_York_060813_JH5		
Localization prob OTE0019_York_060813_JH6		
Score diff OTE0019_York_060813_JH6		
PEP OTE0019_York_060813_JH6		
Score OTE0019_York_060813_JH6		
Localization prob OTE0019_York_060813_JH7		
Score diff OTE0019_York_060813_JH7		
PEP OTE0019_York_060813_JH7		
Score OTE0019_York_060813_JH7		
Localization prob OTE0019_York_060813_JH8		

Score diff OTE0019_York_060813_JH8		
PEP OTE0019_York_060813_JH8		
Score OTE0019_York_060813_JH8		
Localization prob OTE0019_York_060813_JH9		
Score diff OTE0019_York_060813_JH9		
PEP OTE0019_York_060813_JH9		
Score OTE0019_York_060813_JH9		
Localization prob QE0095_JHendy_JH1_130823142140		
Score diff QE0095_JHendy_JH1_130823142140		
PEP QE0095_JHendy_JH1_130823142140		
Score QE0095_JHendy_JH1_130823142140		
Localization prob QE0095_JHendy_JH10_130824054009		
Score diff QE0095_JHendy_JH10_130824054009		
PEP QE0095_JHendy_JH10_130824054009		
Score QE0095_JHendy_JH10_130824054009		
Localization prob QE0095_JHendy_JH11_130824082301		
Score diff QE0095_JHendy_JH11_130824082301		
PEP QE0095_JHendy_JH11_130824082301		
Score QE0095_JHendy_JH11_130824082301		
Localization prob QE0095_JHendy_JH12_130824101054		
Score diff QE0095_JHendy_JH12_130824101054		
PEP QE0095_JHendy_JH12_130824101054		
Score QE0095_JHendy_JH12_130824101054		
Localization prob QE0095_JHendy_JH13_130824115852		
Score diff QE0095_JHendy_JH13_130824115852		
PEP QE0095_JHendy_JH13_130824115852		
Score QE0095_JHendy_JH13_130824115852		
Localization prob QE0095_JHendy_JH14_130824134650		
Score diff QE0095_JHendy_JH14_130824134650		
PEP QE0095_JHendy_JH14_130824134650		

Score QE0095_JHendy_JH14_130824134650		
Localization prob QE0095_JHendy_JH15		
Score diff QE0095_JHendy_JH15		
PEP QE0095_JHendy_JH15		
Score QE0095_JHendy_JH15		
Localization prob QE0095_JHendy_JH16		
Score diff QE0095_JHendy_JH16		
PEP QE0095_JHendy_JH16		
Score QE0095_JHendy_JH16		
Localization prob QE0095_JHendy_JH17		
Score diff QE0095_JHendy_JH17		
PEP QE0095_JHendy_JH17		
Score QE0095_JHendy_JH17		
Localization prob QE0095_JHendy_JH18		
Score diff QE0095_JHendy_JH18		
PEP QE0095_JHendy_JH18		
Score QE0095_JHendy_JH18		
Localization prob QE0095_JHendy_JH19		
Score diff QE0095_JHendy_JH19		
PEP QE0095_JHendy_JH19		
Score QE0095_JHendy_JH19		
Localization prob QE0095_JHendy_JH2		
Score diff QE0095_JHendy_JH2		
PEP QE0095_JHendy_JH2		
Score QE0095_JHendy_JH2		
Localization prob QE0095_JHendy_JH20		
Score diff QE0095_JHendy_JH20		
PEP QE0095_JHendy_JH20		
Score QE0095_JHendy_JH20		
Localization prob QE0095_JHendy_JH3_130823160937		
Score diff QE0095_JHendy_JH3_130823160937		
PEP QE0095_JHendy_JH3_130823160937		
Score QE0095_JHendy_JH3_130823160937		
Localization prob QE0095_JHendy_JH4_130823175743		
Score diff QE0095_JHendy_JH4_130823175743		
PEP QE0095_JHendy_JH4_130823175743		
Score QE0095_JHendy_JH4_130823175743		
Localization prob QE0095_JHendy_JH5_130823194539		
Score diff QE0095_JHendy_JH5_130823194539		
PEP QE0095_JHendy_JH5_130823194539		
Score QE0095_JHendy_JH5_130823194539		

Localization prob QE0095_JHendy_JH6_130823222831		
Score diff QE0095_JHendy_JH6_130823222831		
PEP QE0095_JHendy_JH6_130823222831		
Score QE0095_JHendy_JH6_130823222831		
Localization prob QE0095_JHendy_JH7_130824001623		
Score diff QE0095_JHendy_JH7_130824001623		
PEP QE0095_JHendy_JH7_130824001623		
Score QE0095_JHendy_JH7_130824001623		
Localization prob QE0095_JHendy_JH8_130824020419		
Score diff QE0095_JHendy_JH8_130824020419		
PEP QE0095_JHendy_JH8_130824020419		
Score QE0095_JHendy_JH8_130824020419		
Localization prob QE0095_JHendy_JH9		
Score diff QE0095_JHendy_JH9		
PEP QE0095_JHendy_JH9		
Score QE0095_JHendy_JH9		
Localization prob QE0095_JHendy_JH9_130824035216		
Score diff QE0095_JHendy_JH9_130824035216		
PEP QE0095_JHendy_JH9_130824035216		
Score QE0095_JHendy_JH9_130824035216		
Diagnostic peak		
Number of Hydroxyproline		Different numbers of Hydroxyproline on peptides that this site is involved in.
Amino acid		
Sequence window		
Modification window		
Peptide window coverage		
Hydroxyproline Probabilities		
Hydroxyproline Score diffs		
Position in peptide		
Charge		Charge state of the precursor ion.
Mass error [ppm]		Mass error of the recalibrated mass-over-charge value of the precursor ion in comparison to the predicted monoisotopic mass of the identified peptide sequence.
Intensity		Summed up eXtracted Ion Current (XIC) of all isotopic clusters associated with the identified AA sequence. In case of a labeled experiment this is the total intensity of all the isotopic patterns in the label cluster.
Intensity___1		Summed up eXtracted Ion Current (XIC) of all isotopic clusters associated with the identified AA sequence. In case of a labeled experiment this is the total intensity of all the isotopic patterns in the label cluster.
Intensity___2		Summed up eXtracted Ion Current (XIC) of all isotopic clusters associated with the identified AA sequence. In case of a labeled experiment this is the total intensity of all the isotopic patterns in the label cluster.

Intensity___3		Summed up eXtracted Ion Current (XIC) of all isotopic clusters associated with the identified AA sequence. In case of a labeled experiment this is the total intensity of all the isotopic patterns in the label cluster.
Ratio mod/base		
Intensity OTE0019_York_060813_JH1		Summed up eXtracted Ion Current (XIC) of all isotopic clusters associated with the identified AA sequence. In case of a labeled experiment this is the total intensity of all the isotopic patterns in the label cluster.
Intensity OTE0019_York_060813_JH10		Summed up eXtracted Ion Current (XIC) of all isotopic clusters associated with the identified AA sequence. In case of a labeled experiment this is the total intensity of all the isotopic patterns in the label cluster.
Intensity OTE0019_York_060813_JH11		Summed up eXtracted Ion Current (XIC) of all isotopic clusters associated with the identified AA sequence. In case of a labeled experiment this is the total intensity of all the isotopic patterns in the label cluster.
Intensity OTE0019_York_060813_JH12		Summed up eXtracted Ion Current (XIC) of all isotopic clusters associated with the identified AA sequence. In case of a labeled experiment this is the total intensity of all the isotopic patterns in the label cluster.
Intensity OTE0019_York_060813_JH13		Summed up eXtracted Ion Current (XIC) of all isotopic clusters associated with the identified AA sequence. In case of a labeled experiment this is the total intensity of all the isotopic patterns in the label cluster.
Intensity OTE0019_York_060813_JH14		Summed up eXtracted Ion Current (XIC) of all isotopic clusters associated with the identified AA sequence. In case of a labeled experiment this is the total intensity of all the isotopic patterns in the label cluster.
Intensity OTE0019_York_060813_JH15		Summed up eXtracted Ion Current (XIC) of all isotopic clusters associated with the identified AA sequence. In case of a labeled experiment this is the total intensity of all the isotopic patterns in the label cluster.
Intensity OTE0019_York_060813_JH16		Summed up eXtracted Ion Current (XIC) of all isotopic clusters associated with the identified AA sequence. In case of a labeled experiment this is the total intensity of all the isotopic patterns in the label cluster.
Intensity OTE0019_York_060813_JH17		Summed up eXtracted Ion Current (XIC) of all isotopic clusters associated with the identified AA sequence. In case of a labeled experiment this is the total intensity of all the isotopic patterns in the label cluster.
Intensity OTE0019_York_060813_JH18		Summed up eXtracted Ion Current (XIC) of all isotopic clusters associated with the identified AA sequence. In case of a labeled experiment this is the total intensity of all the isotopic patterns in the label cluster.
Intensity OTE0019_York_060813_JH19		Summed up eXtracted Ion Current (XIC) of all isotopic clusters associated with the identified AA sequence. In case of a labeled experiment this is the total intensity of all the isotopic patterns in the label cluster.
Intensity OTE0019_York_060813_JH2		Summed up eXtracted Ion Current (XIC) of all isotopic clusters associated with the identified AA sequence. In case of a labeled experiment this is the total intensity of all the isotopic patterns in the label cluster.
Intensity OTE0019_York_060813_JH20		Summed up eXtracted Ion Current (XIC) of all isotopic clusters associated with the identified AA sequence. In case of a labeled experiment this is the total intensity of all the isotopic patterns in the label cluster.
Intensity OTE0019_York_060813_JH3		Summed up eXtracted Ion Current (XIC) of all isotopic clusters associated with the identified AA sequence. In case of a labeled experiment this is the total intensity of all the isotopic patterns in the label cluster.
Intensity OTE0019_York_060813_JH4		Summed up eXtracted Ion Current (XIC) of all isotopic clusters associated with the identified AA sequence. In case of a labeled experiment this is the total intensity of all the isotopic patterns in the label cluster.
Intensity OTE0019_York_060813_JH5		Summed up eXtracted Ion Current (XIC) of all isotopic clusters associated with the identified AA sequence. In case of a labeled experiment this is the total intensity of all the isotopic patterns in the label cluster.
Intensity OTE0019_York_060813_JH6		Summed up eXtracted Ion Current (XIC) of all isotopic clusters associated with the identified AA sequence. In case of a labeled experiment this is the total intensity of all the isotopic patterns in the label cluster.
Intensity OTE0019_York_060813_JH7		Summed up eXtracted Ion Current (XIC) of all isotopic clusters associated with the identified AA sequence. In case of a labeled experiment this is the total intensity of all the isotopic patterns in the label cluster.
Intensity OTE0019_York_060813_JH8		Summed up eXtracted Ion Current (XIC) of all isotopic clusters associated with the identified AA sequence. In case of a labeled experiment this is the total intensity of all the isotopic patterns in the label cluster.
Intensity OTE0019_York_060813_JH9		Summed up eXtracted Ion Current (XIC) of all isotopic clusters associated with the identified AA sequence. In case of a labeled experiment this is the total intensity of all the isotopic patterns in the label cluster.

Intensity QE0095_JHendy_JH1_130823142140		Summed up eXtracted Ion Current (XIC) of all isotopic clusters associated with the identified AA sequence. In case of a labeled experiment this is the total intensity of all the isotopic patterns in the label cluster.
Intensity QE0095_JHendy_JH10_130824054009		Summed up eXtracted Ion Current (XIC) of all isotopic clusters associated with the identified AA sequence. In case of a labeled experiment this is the total intensity of all the isotopic patterns in the label cluster.
Intensity QE0095_JHendy_JH11_130824082301		Summed up eXtracted Ion Current (XIC) of all isotopic clusters associated with the identified AA sequence. In case of a labeled experiment this is the total intensity of all the isotopic patterns in the label cluster.
Intensity QE0095_JHendy_JH12_130824101054		Summed up eXtracted Ion Current (XIC) of all isotopic clusters associated with the identified AA sequence. In case of a labeled experiment this is the total intensity of all the isotopic patterns in the label cluster.
Intensity QE0095_JHendy_JH13_130824115852		Summed up eXtracted Ion Current (XIC) of all isotopic clusters associated with the identified AA sequence. In case of a labeled experiment this is the total intensity of all the isotopic patterns in the label cluster.
Intensity QE0095_JHendy_JH14_130824134650		Summed up eXtracted Ion Current (XIC) of all isotopic clusters associated with the identified AA sequence. In case of a labeled experiment this is the total intensity of all the isotopic patterns in the label cluster.
Intensity QE0095_JHendy_JH15		Summed up eXtracted Ion Current (XIC) of all isotopic clusters associated with the identified AA sequence. In case of a labeled experiment this is the total intensity of all the isotopic patterns in the label cluster.
Intensity QE0095_JHendy_JH16		Summed up eXtracted Ion Current (XIC) of all isotopic clusters associated with the identified AA sequence. In case of a labeled experiment this is the total intensity of all the isotopic patterns in the label cluster.
Intensity QE0095_JHendy_JH17		Summed up eXtracted Ion Current (XIC) of all isotopic clusters associated with the identified AA sequence. In case of a labeled experiment this is the total intensity of all the isotopic patterns in the label cluster.
Intensity QE0095_JHendy_JH18		Summed up eXtracted Ion Current (XIC) of all isotopic clusters associated with the identified AA sequence. In case of a labeled experiment this is the total intensity of all the isotopic patterns in the label cluster.
Intensity QE0095_JHendy_JH19		Summed up eXtracted Ion Current (XIC) of all isotopic clusters associated with the identified AA sequence. In case of a labeled experiment this is the total intensity of all the isotopic patterns in the label cluster.
Intensity QE0095_JHendy_JH2		Summed up eXtracted Ion Current (XIC) of all isotopic clusters associated with the identified AA sequence. In case of a labeled experiment this is the total intensity of all the isotopic patterns in the label cluster.
Intensity QE0095_JHendy_JH20		Summed up eXtracted Ion Current (XIC) of all isotopic clusters associated with the identified AA sequence. In case of a labeled experiment this is the total intensity of all the isotopic patterns in the label cluster.
Intensity QE0095_JHendy_JH3_130823160937		Summed up eXtracted Ion Current (XIC) of all isotopic clusters associated with the identified AA sequence. In case of a labeled experiment this is the total intensity of all the isotopic patterns in the label cluster.
Intensity QE0095_JHendy_JH4_130823175743		Summed up eXtracted Ion Current (XIC) of all isotopic clusters associated with the identified AA sequence. In case of a labeled experiment this is the total intensity of all the isotopic patterns in the label cluster.
Intensity QE0095_JHendy_JH5_130823194539		Summed up eXtracted Ion Current (XIC) of all isotopic clusters associated with the identified AA sequence. In case of a labeled experiment this is the total intensity of all the isotopic patterns in the label cluster.
Intensity QE0095_JHendy_JH6_130823222831		Summed up eXtracted Ion Current (XIC) of all isotopic clusters associated with the identified AA sequence. In case of a labeled experiment this is the total intensity of all the isotopic patterns in the label cluster.
Intensity QE0095_JHendy_JH7_130824001623		Summed up eXtracted Ion Current (XIC) of all isotopic clusters associated with the identified AA sequence. In case of a labeled experiment this is the total intensity of all the isotopic patterns in the label cluster.
Intensity QE0095_JHendy_JH8_130824020419		Summed up eXtracted Ion Current (XIC) of all isotopic clusters associated with the identified AA sequence. In case of a labeled experiment this is the total intensity of all the isotopic patterns in the label cluster.
Intensity QE0095_JHendy_JH9		Summed up eXtracted Ion Current (XIC) of all isotopic clusters associated with the identified AA sequence. In case of a labeled experiment this is the total intensity of all the isotopic patterns in the label cluster.
Intensity QE0095_JHendy_JH9_130824035216		Summed up eXtracted Ion Current (XIC) of all isotopic clusters associated with the identified AA sequence. In case of a labeled experiment this is the total intensity of all the isotopic patterns in the label cluster.

Ratio mod/base OTE0019_York_060813_JH1		
Ratio mod/base OTE0019_York_060813_JH10		
Ratio mod/base OTE0019_York_060813_JH11		
Ratio mod/base OTE0019_York_060813_JH12		
Ratio mod/base OTE0019_York_060813_JH13		
Ratio mod/base OTE0019_York_060813_JH14		
Ratio mod/base OTE0019_York_060813_JH15		
Ratio mod/base OTE0019_York_060813_JH16		
Ratio mod/base OTE0019_York_060813_JH17		
Ratio mod/base OTE0019_York_060813_JH18		
Ratio mod/base OTE0019_York_060813_JH19		
Ratio mod/base OTE0019_York_060813_JH2		
Ratio mod/base OTE0019_York_060813_JH20		
Ratio mod/base OTE0019_York_060813_JH3		
Ratio mod/base OTE0019_York_060813_JH4		
Ratio mod/base OTE0019_York_060813_JH5		
Ratio mod/base OTE0019_York_060813_JH6		
Ratio mod/base OTE0019_York_060813_JH7		
Ratio mod/base OTE0019_York_060813_JH8		
Ratio mod/base OTE0019_York_060813_JH9		
Ratio mod/base QE0095_JHendy_JH1_130823142140		
Ratio mod/base QE0095_JHendy_JH10_130824054009		
Ratio mod/base QE0095_JHendy_JH11_130824082301		
Ratio mod/base QE0095_JHendy_JH12_130824101054		
Ratio mod/base QE0095_JHendy_JH13_130824115852		
Ratio mod/base QE0095_JHendy_JH14_130824134650		
Ratio mod/base QE0095_JHendy_JH15		
Ratio mod/base QE0095_JHendy_JH16		
Ratio mod/base QE0095_JHendy_JH17		
Ratio mod/base QE0095_JHendy_JH18		
Ratio mod/base QE0095_JHendy_JH19		
Ratio mod/base QE0095_JHendy_JH2		
Ratio mod/base QE0095_JHendy_JH20		
Ratio mod/base QE0095_JHendy_JH3_130823160937		
Ratio mod/base QE0095_JHendy_JH4_130823175743		

Ratio mod/base QE0095_JHendy_JH5_130823194539		
Ratio mod/base QE0095_JHendy_JH6_130823222831		
Ratio mod/base QE0095_JHendy_JH7_130824001623		
Ratio mod/base QE0095_JHendy_JH8_130824020419		
Ratio mod/base QE0095_JHendy_JH9		
Ratio mod/base QE0095_JHendy_JH9_130824035216		
Intensity OTE0019_York_060813_JH1___1		Summed up eXtracted Ion Current (XIC) of all isotopic clusters associated with the identified AA sequence. In case of a labeled experiment this is the total intensity of all the isotopic patterns in the label cluster.
Intensity OTE0019_York_060813_JH1___2		Summed up eXtracted Ion Current (XIC) of all isotopic clusters associated with the identified AA sequence. In case of a labeled experiment this is the total intensity of all the isotopic patterns in the label cluster.
Intensity OTE0019_York_060813_JH1___3		Summed up eXtracted Ion Current (XIC) of all isotopic clusters associated with the identified AA sequence. In case of a labeled experiment this is the total intensity of all the isotopic patterns in the label cluster.
Intensity OTE0019_York_060813_JH10___1		Summed up eXtracted Ion Current (XIC) of all isotopic clusters associated with the identified AA sequence. In case of a labeled experiment this is the total intensity of all the isotopic patterns in the label cluster.
Intensity OTE0019_York_060813_JH10___2		Summed up eXtracted Ion Current (XIC) of all isotopic clusters associated with the identified AA sequence. In case of a labeled experiment this is the total intensity of all the isotopic patterns in the label cluster.
Intensity OTE0019_York_060813_JH10___3		Summed up eXtracted Ion Current (XIC) of all isotopic clusters associated with the identified AA sequence. In case of a labeled experiment this is the total intensity of all the isotopic patterns in the label cluster.
Intensity OTE0019_York_060813_JH11___1		Summed up eXtracted Ion Current (XIC) of all isotopic clusters associated with the identified AA sequence. In case of a labeled experiment this is the total intensity of all the isotopic patterns in the label cluster.
Intensity OTE0019_York_060813_JH11___2		Summed up eXtracted Ion Current (XIC) of all isotopic clusters associated with the identified AA sequence. In case of a labeled experiment this is the total intensity of all the isotopic patterns in the label cluster.
Intensity OTE0019_York_060813_JH11___3		Summed up eXtracted Ion Current (XIC) of all isotopic clusters associated with the identified AA sequence. In case of a labeled experiment this is the total intensity of all the isotopic patterns in the label cluster.
Intensity OTE0019_York_060813_JH12___1		Summed up eXtracted Ion Current (XIC) of all isotopic clusters associated with the identified AA sequence. In case of a labeled experiment this is the total intensity of all the isotopic patterns in the label cluster.
Intensity OTE0019_York_060813_JH12___2		Summed up eXtracted Ion Current (XIC) of all isotopic clusters associated with the identified AA sequence. In case of a labeled experiment this is the total intensity of all the isotopic patterns in the label cluster.
Intensity OTE0019_York_060813_JH12___3		Summed up eXtracted Ion Current (XIC) of all isotopic clusters associated with the identified AA sequence. In case of a labeled experiment this is the total intensity of all the isotopic patterns in the label cluster.
Intensity OTE0019_York_060813_JH13___1		Summed up eXtracted Ion Current (XIC) of all isotopic clusters associated with the identified AA sequence. In case of a labeled experiment this is the total intensity of all the isotopic patterns in the label cluster.
Intensity OTE0019_York_060813_JH13___2		Summed up eXtracted Ion Current (XIC) of all isotopic clusters associated with the identified AA sequence. In case of a labeled experiment this is the total intensity of all the isotopic patterns in the label cluster.
Intensity OTE0019_York_060813_JH13___3		Summed up eXtracted Ion Current (XIC) of all isotopic clusters associated with the identified AA sequence. In case of a labeled experiment this is the total intensity of all the isotopic patterns in the label cluster.
Intensity OTE0019_York_060813_JH14___1		Summed up eXtracted Ion Current (XIC) of all isotopic clusters associated with the identified AA sequence. In case of a labeled experiment this is the total intensity of all the isotopic patterns in the label cluster.

Intensity OTE0019_York_060813_JH14___2		Summed up eXtracted Ion Current (XIC) of all isotopic clusters associated with the identified AA sequence. In case of a labeled experiment this is the total intensity of all the isotopic patterns in the label cluster.
Intensity OTE0019_York_060813_JH14___3		Summed up eXtracted Ion Current (XIC) of all isotopic clusters associated with the identified AA sequence. In case of a labeled experiment this is the total intensity of all the isotopic patterns in the label cluster.
Intensity OTE0019_York_060813_JH15___1		Summed up eXtracted Ion Current (XIC) of all isotopic clusters associated with the identified AA sequence. In case of a labeled experiment this is the total intensity of all the isotopic patterns in the label cluster.
Intensity OTE0019_York_060813_JH15___2		Summed up eXtracted Ion Current (XIC) of all isotopic clusters associated with the identified AA sequence. In case of a labeled experiment this is the total intensity of all the isotopic patterns in the label cluster.
Intensity OTE0019_York_060813_JH15___3		Summed up eXtracted Ion Current (XIC) of all isotopic clusters associated with the identified AA sequence. In case of a labeled experiment this is the total intensity of all the isotopic patterns in the label cluster.
Intensity OTE0019_York_060813_JH16___1		Summed up eXtracted Ion Current (XIC) of all isotopic clusters associated with the identified AA sequence. In case of a labeled experiment this is the total intensity of all the isotopic patterns in the label cluster.
Intensity OTE0019_York_060813_JH16___2		Summed up eXtracted Ion Current (XIC) of all isotopic clusters associated with the identified AA sequence. In case of a labeled experiment this is the total intensity of all the isotopic patterns in the label cluster.
Intensity OTE0019_York_060813_JH16___3		Summed up eXtracted Ion Current (XIC) of all isotopic clusters associated with the identified AA sequence. In case of a labeled experiment this is the total intensity of all the isotopic patterns in the label cluster.
Intensity OTE0019_York_060813_JH17___1		Summed up eXtracted Ion Current (XIC) of all isotopic clusters associated with the identified AA sequence. In case of a labeled experiment this is the total intensity of all the isotopic patterns in the label cluster.
Intensity OTE0019_York_060813_JH17___2		Summed up eXtracted Ion Current (XIC) of all isotopic clusters associated with the identified AA sequence. In case of a labeled experiment this is the total intensity of all the isotopic patterns in the label cluster.
Intensity OTE0019_York_060813_JH17___3		Summed up eXtracted Ion Current (XIC) of all isotopic clusters associated with the identified AA sequence. In case of a labeled experiment this is the total intensity of all the isotopic patterns in the label cluster.
Intensity OTE0019_York_060813_JH18___1		Summed up eXtracted Ion Current (XIC) of all isotopic clusters associated with the identified AA sequence. In case of a labeled experiment this is the total intensity of all the isotopic patterns in the label cluster.
Intensity OTE0019_York_060813_JH18___2		Summed up eXtracted Ion Current (XIC) of all isotopic clusters associated with the identified AA sequence. In case of a labeled experiment this is the total intensity of all the isotopic patterns in the label cluster.
Intensity OTE0019_York_060813_JH18___3		Summed up eXtracted Ion Current (XIC) of all isotopic clusters associated with the identified AA sequence. In case of a labeled experiment this is the total intensity of all the isotopic patterns in the label cluster.
Intensity OTE0019_York_060813_JH19___1		Summed up eXtracted Ion Current (XIC) of all isotopic clusters associated with the identified AA sequence. In case of a labeled experiment this is the total intensity of all the isotopic patterns in the label cluster.
Intensity OTE0019_York_060813_JH19___2		Summed up eXtracted Ion Current (XIC) of all isotopic clusters associated with the identified AA sequence. In case of a labeled experiment this is the total intensity of all the isotopic patterns in the label cluster.
Intensity OTE0019_York_060813_JH19___3		Summed up eXtracted Ion Current (XIC) of all isotopic clusters associated with the identified AA sequence. In case of a labeled experiment this is the total intensity of all the isotopic patterns in the label cluster.
Intensity OTE0019_York_060813_JH2___1		Summed up eXtracted Ion Current (XIC) of all isotopic clusters associated with the identified AA sequence. In case of a labeled experiment this is the total intensity of all the isotopic patterns in the label cluster.
Intensity OTE0019_York_060813_JH2___2		Summed up eXtracted Ion Current (XIC) of all isotopic clusters associated with the identified AA sequence. In case of a labeled experiment this is the total intensity of all the isotopic patterns in the label cluster.
Intensity OTE0019_York_060813_JH2___3		Summed up eXtracted Ion Current (XIC) of all isotopic clusters associated with the identified AA sequence. In case of a labeled experiment this is the total intensity of all the isotopic patterns in the label cluster.
Intensity OTE0019_York_060813_JH20___1		Summed up eXtracted Ion Current (XIC) of all isotopic clusters associated with the identified AA sequence. In case of a labeled experiment this is the total intensity of all the isotopic patterns in the label cluster.

Intensity OTE0019_York_060813_JH20___2		Summed up eXtracted Ion Current (XIC) of all isotopic clusters associated with the identified AA sequence. In case of a labeled experiment this is the total intensity of all the isotopic patterns in the label cluster.
Intensity OTE0019_York_060813_JH20___3		Summed up eXtracted Ion Current (XIC) of all isotopic clusters associated with the identified AA sequence. In case of a labeled experiment this is the total intensity of all the isotopic patterns in the label cluster.
Intensity OTE0019_York_060813_JH3___1		Summed up eXtracted Ion Current (XIC) of all isotopic clusters associated with the identified AA sequence. In case of a labeled experiment this is the total intensity of all the isotopic patterns in the label cluster.
Intensity OTE0019_York_060813_JH3___2		Summed up eXtracted Ion Current (XIC) of all isotopic clusters associated with the identified AA sequence. In case of a labeled experiment this is the total intensity of all the isotopic patterns in the label cluster.
Intensity OTE0019_York_060813_JH3___3		Summed up eXtracted Ion Current (XIC) of all isotopic clusters associated with the identified AA sequence. In case of a labeled experiment this is the total intensity of all the isotopic patterns in the label cluster.
Intensity OTE0019_York_060813_JH4___1		Summed up eXtracted Ion Current (XIC) of all isotopic clusters associated with the identified AA sequence. In case of a labeled experiment this is the total intensity of all the isotopic patterns in the label cluster.
Intensity OTE0019_York_060813_JH4___2		Summed up eXtracted Ion Current (XIC) of all isotopic clusters associated with the identified AA sequence. In case of a labeled experiment this is the total intensity of all the isotopic patterns in the label cluster.
Intensity OTE0019_York_060813_JH4___3		Summed up eXtracted Ion Current (XIC) of all isotopic clusters associated with the identified AA sequence. In case of a labeled experiment this is the total intensity of all the isotopic patterns in the label cluster.
Intensity OTE0019_York_060813_JH5___1		Summed up eXtracted Ion Current (XIC) of all isotopic clusters associated with the identified AA sequence. In case of a labeled experiment this is the total intensity of all the isotopic patterns in the label cluster.
Intensity OTE0019_York_060813_JH5___2		Summed up eXtracted Ion Current (XIC) of all isotopic clusters associated with the identified AA sequence. In case of a labeled experiment this is the total intensity of all the isotopic patterns in the label cluster.
Intensity OTE0019_York_060813_JH5___3		Summed up eXtracted Ion Current (XIC) of all isotopic clusters associated with the identified AA sequence. In case of a labeled experiment this is the total intensity of all the isotopic patterns in the label cluster.
Intensity OTE0019_York_060813_JH6___1		Summed up eXtracted Ion Current (XIC) of all isotopic clusters associated with the identified AA sequence. In case of a labeled experiment this is the total intensity of all the isotopic patterns in the label cluster.
Intensity OTE0019_York_060813_JH6___2		Summed up eXtracted Ion Current (XIC) of all isotopic clusters associated with the identified AA sequence. In case of a labeled experiment this is the total intensity of all the isotopic patterns in the label cluster.
Intensity OTE0019_York_060813_JH6___3		Summed up eXtracted Ion Current (XIC) of all isotopic clusters associated with the identified AA sequence. In case of a labeled experiment this is the total intensity of all the isotopic patterns in the label cluster.
Intensity OTE0019_York_060813_JH7___1		Summed up eXtracted Ion Current (XIC) of all isotopic clusters associated with the identified AA sequence. In case of a labeled experiment this is the total intensity of all the isotopic patterns in the label cluster.
Intensity OTE0019_York_060813_JH7___2		Summed up eXtracted Ion Current (XIC) of all isotopic clusters associated with the identified AA sequence. In case of a labeled experiment this is the total intensity of all the isotopic patterns in the label cluster.
Intensity OTE0019_York_060813_JH7___3		Summed up eXtracted Ion Current (XIC) of all isotopic clusters associated with the identified AA sequence. In case of a labeled experiment this is the total intensity of all the isotopic patterns in the label cluster.
Intensity OTE0019_York_060813_JH8___1		Summed up eXtracted Ion Current (XIC) of all isotopic clusters associated with the identified AA sequence. In case of a labeled experiment this is the total intensity of all the isotopic patterns in the label cluster.
Intensity OTE0019_York_060813_JH8___2		Summed up eXtracted Ion Current (XIC) of all isotopic clusters associated with the identified AA sequence. In case of a labeled experiment this is the total intensity of all the isotopic patterns in the label cluster.
Intensity OTE0019_York_060813_JH8___3		Summed up eXtracted Ion Current (XIC) of all isotopic clusters associated with the identified AA sequence. In case of a labeled experiment this is the total intensity of all the isotopic patterns in the label cluster.
Intensity OTE0019_York_060813_JH9___1		Summed up eXtracted Ion Current (XIC) of all isotopic clusters associated with the identified AA sequence. In case of a labeled experiment this is the total intensity of all the isotopic patterns in the label cluster.

Intensity OTE0019_York_060813_JH9___2		Summed up eXtracted Ion Current (XIC) of all isotopic clusters associated with the identified AA sequence. In case of a labeled experiment this is the total intensity of all the isotopic patterns in the label cluster.
Intensity OTE0019_York_060813_JH9___3		Summed up eXtracted Ion Current (XIC) of all isotopic clusters associated with the identified AA sequence. In case of a labeled experiment this is the total intensity of all the isotopic patterns in the label cluster.
Intensity QE0095_JHendy_JH1_130823142140___1		Summed up eXtracted Ion Current (XIC) of all isotopic clusters associated with the identified AA sequence. In case of a labeled experiment this is the total intensity of all the isotopic patterns in the label cluster.
Intensity QE0095_JHendy_JH1_130823142140___2		Summed up eXtracted Ion Current (XIC) of all isotopic clusters associated with the identified AA sequence. In case of a labeled experiment this is the total intensity of all the isotopic patterns in the label cluster.
Intensity QE0095_JHendy_JH1_130823142140___3		Summed up eXtracted Ion Current (XIC) of all isotopic clusters associated with the identified AA sequence. In case of a labeled experiment this is the total intensity of all the isotopic patterns in the label cluster.
Intensity QE0095_JHendy_JH10_130824054009___1		Summed up eXtracted Ion Current (XIC) of all isotopic clusters associated with the identified AA sequence. In case of a labeled experiment this is the total intensity of all the isotopic patterns in the label cluster.
Intensity QE0095_JHendy_JH10_130824054009___2		Summed up eXtracted Ion Current (XIC) of all isotopic clusters associated with the identified AA sequence. In case of a labeled experiment this is the total intensity of all the isotopic patterns in the label cluster.
Intensity QE0095_JHendy_JH10_130824054009___3		Summed up eXtracted Ion Current (XIC) of all isotopic clusters associated with the identified AA sequence. In case of a labeled experiment this is the total intensity of all the isotopic patterns in the label cluster.
Intensity QE0095_JHendy_JH11_130824082301___1		Summed up eXtracted Ion Current (XIC) of all isotopic clusters associated with the identified AA sequence. In case of a labeled experiment this is the total intensity of all the isotopic patterns in the label cluster.
Intensity QE0095_JHendy_JH11_130824082301___2		Summed up eXtracted Ion Current (XIC) of all isotopic clusters associated with the identified AA sequence. In case of a labeled experiment this is the total intensity of all the isotopic patterns in the label cluster.
Intensity QE0095_JHendy_JH11_130824082301___3		Summed up eXtracted Ion Current (XIC) of all isotopic clusters associated with the identified AA sequence. In case of a labeled experiment this is the total intensity of all the isotopic patterns in the label cluster.
Intensity QE0095_JHendy_JH12_130824101054___1		Summed up eXtracted Ion Current (XIC) of all isotopic clusters associated with the identified AA sequence. In case of a labeled experiment this is the total intensity of all the isotopic patterns in the label cluster.
Intensity QE0095_JHendy_JH12_130824101054___2		Summed up eXtracted Ion Current (XIC) of all isotopic clusters associated with the identified AA sequence. In case of a labeled experiment this is the total intensity of all the isotopic patterns in the label cluster.
Intensity QE0095_JHendy_JH12_130824101054___3		Summed up eXtracted Ion Current (XIC) of all isotopic clusters associated with the identified AA sequence. In case of a labeled experiment this is the total intensity of all the isotopic patterns in the label cluster.
Intensity QE0095_JHendy_JH13_130824115852___1		Summed up eXtracted Ion Current (XIC) of all isotopic clusters associated with the identified AA sequence. In case of a labeled experiment this is the total intensity of all the isotopic patterns in the label cluster.
Intensity QE0095_JHendy_JH13_130824115852___2		Summed up eXtracted Ion Current (XIC) of all isotopic clusters associated with the identified AA sequence. In case of a labeled experiment this is the total intensity of all the isotopic patterns in the label cluster.
Intensity QE0095_JHendy_JH13_130824115852___3		Summed up eXtracted Ion Current (XIC) of all isotopic clusters associated with the identified AA sequence. In case of a labeled experiment this is the total intensity of all the isotopic patterns in the label cluster.
Intensity QE0095_JHendy_JH14_130824134650___1		Summed up eXtracted Ion Current (XIC) of all isotopic clusters associated with the identified AA sequence. In case of a labeled experiment this is the total intensity of all the isotopic patterns in the label cluster.
Intensity QE0095_JHendy_JH14_130824134650___2		Summed up eXtracted Ion Current (XIC) of all isotopic clusters associated with the identified AA sequence. In case of a labeled experiment this is the total intensity of all the isotopic patterns in the label cluster.
Intensity QE0095_JHendy_JH14_130824134650___3		Summed up eXtracted Ion Current (XIC) of all isotopic clusters associated with the identified AA sequence. In case of a labeled experiment this is the total intensity of all the isotopic patterns in the label cluster.
Intensity QE0095_JHendy_JH15___1		Summed up eXtracted Ion Current (XIC) of all isotopic clusters associated with the identified AA sequence. In case of a labeled experiment this is the total intensity of all the isotopic patterns in the label cluster.

Intensity QE0095_JHendy_JH15___2		Summed up eXtracted Ion Current (XIC) of all isotopic clusters associated with the identified AA sequence. In case of a labeled experiment this is the total intensity of all the isotopic patterns in the label cluster.
Intensity QE0095_JHendy_JH15___3		Summed up eXtracted Ion Current (XIC) of all isotopic clusters associated with the identified AA sequence. In case of a labeled experiment this is the total intensity of all the isotopic patterns in the label cluster.
Intensity QE0095_JHendy_JH16___1		Summed up eXtracted Ion Current (XIC) of all isotopic clusters associated with the identified AA sequence. In case of a labeled experiment this is the total intensity of all the isotopic patterns in the label cluster.
Intensity QE0095_JHendy_JH16___2		Summed up eXtracted Ion Current (XIC) of all isotopic clusters associated with the identified AA sequence. In case of a labeled experiment this is the total intensity of all the isotopic patterns in the label cluster.
Intensity QE0095_JHendy_JH16___3		Summed up eXtracted Ion Current (XIC) of all isotopic clusters associated with the identified AA sequence. In case of a labeled experiment this is the total intensity of all the isotopic patterns in the label cluster.
Intensity QE0095_JHendy_JH17___1		Summed up eXtracted Ion Current (XIC) of all isotopic clusters associated with the identified AA sequence. In case of a labeled experiment this is the total intensity of all the isotopic patterns in the label cluster.
Intensity QE0095_JHendy_JH17___2		Summed up eXtracted Ion Current (XIC) of all isotopic clusters associated with the identified AA sequence. In case of a labeled experiment this is the total intensity of all the isotopic patterns in the label cluster.
Intensity QE0095_JHendy_JH17___3		Summed up eXtracted Ion Current (XIC) of all isotopic clusters associated with the identified AA sequence. In case of a labeled experiment this is the total intensity of all the isotopic patterns in the label cluster.
Intensity QE0095_JHendy_JH18___1		Summed up eXtracted Ion Current (XIC) of all isotopic clusters associated with the identified AA sequence. In case of a labeled experiment this is the total intensity of all the isotopic patterns in the label cluster.
Intensity QE0095_JHendy_JH18___2		Summed up eXtracted Ion Current (XIC) of all isotopic clusters associated with the identified AA sequence. In case of a labeled experiment this is the total intensity of all the isotopic patterns in the label cluster.
Intensity QE0095_JHendy_JH18___3		Summed up eXtracted Ion Current (XIC) of all isotopic clusters associated with the identified AA sequence. In case of a labeled experiment this is the total intensity of all the isotopic patterns in the label cluster.
Intensity QE0095_JHendy_JH19___1		Summed up eXtracted Ion Current (XIC) of all isotopic clusters associated with the identified AA sequence. In case of a labeled experiment this is the total intensity of all the isotopic patterns in the label cluster.
Intensity QE0095_JHendy_JH19___2		Summed up eXtracted Ion Current (XIC) of all isotopic clusters associated with the identified AA sequence. In case of a labeled experiment this is the total intensity of all the isotopic patterns in the label cluster.
Intensity QE0095_JHendy_JH19___3		Summed up eXtracted Ion Current (XIC) of all isotopic clusters associated with the identified AA sequence. In case of a labeled experiment this is the total intensity of all the isotopic patterns in the label cluster.
Intensity QE0095_JHendy_JH2___1		Summed up eXtracted Ion Current (XIC) of all isotopic clusters associated with the identified AA sequence. In case of a labeled experiment this is the total intensity of all the isotopic patterns in the label cluster.
Intensity QE0095_JHendy_JH2___2		Summed up eXtracted Ion Current (XIC) of all isotopic clusters associated with the identified AA sequence. In case of a labeled experiment this is the total intensity of all the isotopic patterns in the label cluster.
Intensity QE0095_JHendy_JH2___3		Summed up eXtracted Ion Current (XIC) of all isotopic clusters associated with the identified AA sequence. In case of a labeled experiment this is the total intensity of all the isotopic patterns in the label cluster.
Intensity QE0095_JHendy_JH20___1		Summed up eXtracted Ion Current (XIC) of all isotopic clusters associated with the identified AA sequence. In case of a labeled experiment this is the total intensity of all the isotopic patterns in the label cluster.
Intensity QE0095_JHendy_JH20___2		Summed up eXtracted Ion Current (XIC) of all isotopic clusters associated with the identified AA sequence. In case of a labeled experiment this is the total intensity of all the isotopic patterns in the label cluster.
Intensity QE0095_JHendy_JH20___3		Summed up eXtracted Ion Current (XIC) of all isotopic clusters associated with the identified AA sequence. In case of a labeled experiment this is the total intensity of all the isotopic patterns in the label cluster.
Intensity QE0095_JHendy_JH3_130823160937___1		Summed up eXtracted Ion Current (XIC) of all isotopic clusters associated with the identified AA sequence. In case of a labeled experiment this is the total intensity of all the isotopic patterns in the label cluster.

Intensity QE0095_JHendy_JH3_130823160937___2		Summed up eXtracted Ion Current (XIC) of all isotopic clusters associated with the identified AA sequence. In case of a labeled experiment this is the total intensity of all the isotopic patterns in the label cluster.
Intensity QE0095_JHendy_JH3_130823160937___3		Summed up eXtracted Ion Current (XIC) of all isotopic clusters associated with the identified AA sequence. In case of a labeled experiment this is the total intensity of all the isotopic patterns in the label cluster.
Intensity QE0095_JHendy_JH4_130823175743___1		Summed up eXtracted Ion Current (XIC) of all isotopic clusters associated with the identified AA sequence. In case of a labeled experiment this is the total intensity of all the isotopic patterns in the label cluster.
Intensity QE0095_JHendy_JH4_130823175743___2		Summed up eXtracted Ion Current (XIC) of all isotopic clusters associated with the identified AA sequence. In case of a labeled experiment this is the total intensity of all the isotopic patterns in the label cluster.
Intensity QE0095_JHendy_JH4_130823175743___3		Summed up eXtracted Ion Current (XIC) of all isotopic clusters associated with the identified AA sequence. In case of a labeled experiment this is the total intensity of all the isotopic patterns in the label cluster.
Intensity QE0095_JHendy_JH5_130823194539___1		Summed up eXtracted Ion Current (XIC) of all isotopic clusters associated with the identified AA sequence. In case of a labeled experiment this is the total intensity of all the isotopic patterns in the label cluster.
Intensity QE0095_JHendy_JH5_130823194539___2		Summed up eXtracted Ion Current (XIC) of all isotopic clusters associated with the identified AA sequence. In case of a labeled experiment this is the total intensity of all the isotopic patterns in the label cluster.
Intensity QE0095_JHendy_JH5_130823194539___3		Summed up eXtracted Ion Current (XIC) of all isotopic clusters associated with the identified AA sequence. In case of a labeled experiment this is the total intensity of all the isotopic patterns in the label cluster.
Intensity QE0095_JHendy_JH6_130823222831___1		Summed up eXtracted Ion Current (XIC) of all isotopic clusters associated with the identified AA sequence. In case of a labeled experiment this is the total intensity of all the isotopic patterns in the label cluster.
Intensity QE0095_JHendy_JH6_130823222831___2		Summed up eXtracted Ion Current (XIC) of all isotopic clusters associated with the identified AA sequence. In case of a labeled experiment this is the total intensity of all the isotopic patterns in the label cluster.
Intensity QE0095_JHendy_JH6_130823222831___3		Summed up eXtracted Ion Current (XIC) of all isotopic clusters associated with the identified AA sequence. In case of a labeled experiment this is the total intensity of all the isotopic patterns in the label cluster.
Intensity QE0095_JHendy_JH7_130824001623___1		Summed up eXtracted Ion Current (XIC) of all isotopic clusters associated with the identified AA sequence. In case of a labeled experiment this is the total intensity of all the isotopic patterns in the label cluster.
Intensity QE0095_JHendy_JH7_130824001623___2		Summed up eXtracted Ion Current (XIC) of all isotopic clusters associated with the identified AA sequence. In case of a labeled experiment this is the total intensity of all the isotopic patterns in the label cluster.
Intensity QE0095_JHendy_JH7_130824001623___3		Summed up eXtracted Ion Current (XIC) of all isotopic clusters associated with the identified AA sequence. In case of a labeled experiment this is the total intensity of all the isotopic patterns in the label cluster.
Intensity QE0095_JHendy_JH8_130824020419___1		Summed up eXtracted Ion Current (XIC) of all isotopic clusters associated with the identified AA sequence. In case of a labeled experiment this is the total intensity of all the isotopic patterns in the label cluster.
Intensity QE0095_JHendy_JH8_130824020419___2		Summed up eXtracted Ion Current (XIC) of all isotopic clusters associated with the identified AA sequence. In case of a labeled experiment this is the total intensity of all the isotopic patterns in the label cluster.
Intensity QE0095_JHendy_JH8_130824020419___3		Summed up eXtracted Ion Current (XIC) of all isotopic clusters associated with the identified AA sequence. In case of a labeled experiment this is the total intensity of all the isotopic patterns in the label cluster.
Intensity QE0095_JHendy_JH9___1		Summed up eXtracted Ion Current (XIC) of all isotopic clusters associated with the identified AA sequence. In case of a labeled experiment this is the total intensity of all the isotopic patterns in the label cluster.
Intensity QE0095_JHendy_JH9___2		Summed up eXtracted Ion Current (XIC) of all isotopic clusters associated with the identified AA sequence. In case of a labeled experiment this is the total intensity of all the isotopic patterns in the label cluster.
Intensity QE0095_JHendy_JH9___3		Summed up eXtracted Ion Current (XIC) of all isotopic clusters associated with the identified AA sequence. In case of a labeled experiment this is the total intensity of all the isotopic patterns in the label cluster.
Intensity QE0095_JHendy_JH9_130824035216___1		Summed up eXtracted Ion Current (XIC) of all isotopic clusters associated with the identified AA sequence. In case of a labeled experiment this is the total intensity of all the isotopic patterns in the label cluster.

Intensity QE0095_JHendy_JH9_130824035216___2		Summed up eXtracted Ion Current (XIC) of all isotopic clusters associated with the identified AA sequence. In case of a labeled experiment this is the total intensity of all the isotopic patterns in the label cluster.
Intensity QE0095_JHendy_JH9_130824035216___3		Summed up eXtracted Ion Current (XIC) of all isotopic clusters associated with the identified AA sequence. In case of a labeled experiment this is the total intensity of all the isotopic patterns in the label cluster.
Occupancy OTE0019_York_060813_JH1		
Occupancy ratioOTE0019_York_060813_JH1		
Occupancy error scale OTE0019_York_060813_JH1		
Occupancy OTE0019_York_060813_JH10		
Occupancy ratioOTE0019_York_060813_JH10		
Occupancy error scale OTE0019_York_060813_JH10		
Occupancy OTE0019_York_060813_JH11		
Occupancy ratioOTE0019_York_060813_JH11		
Occupancy error scale OTE0019_York_060813_JH11		
Occupancy OTE0019_York_060813_JH12		
Occupancy ratioOTE0019_York_060813_JH12		
Occupancy error scale OTE0019_York_060813_JH12		
Occupancy OTE0019_York_060813_JH13		
Occupancy ratioOTE0019_York_060813_JH13		
Occupancy error scale OTE0019_York_060813_JH13		
Occupancy OTE0019_York_060813_JH14		
Occupancy ratioOTE0019_York_060813_JH14		
Occupancy error scale OTE0019_York_060813_JH14		
Occupancy OTE0019_York_060813_JH15		
Occupancy ratioOTE0019_York_060813_JH15		
Occupancy error scale OTE0019_York_060813_JH15		
Occupancy OTE0019_York_060813_JH16		
Occupancy ratioOTE0019_York_060813_JH16		
Occupancy error scale OTE0019_York_060813_JH16		
Occupancy OTE0019_York_060813_JH17		
Occupancy ratioOTE0019_York_060813_JH17		
Occupancy error scale OTE0019_York_060813_JH17		
Occupancy OTE0019_York_060813_JH18		
Occupancy ratioOTE0019_York_060813_JH18		
Occupancy error scale OTE0019_York_060813_JH18		
Occupancy OTE0019_York_060813_JH19		
Occupancy ratioOTE0019_York_060813_JH19		
Occupancy error scale OTE0019_York_060813_JH19		
Occupancy OTE0019_York_060813_JH2		

Occupancy ratioOTE0019_York_060813_JH2		
Occupancy error scale OTE0019_York_060813_JH2		
Occupancy OTE0019_York_060813_JH20		
Occupancy ratioOTE0019_York_060813_JH20		
Occupancy error scale OTE0019_York_060813_JH20		
Occupancy OTE0019_York_060813_JH3		
Occupancy ratioOTE0019_York_060813_JH3		
Occupancy error scale OTE0019_York_060813_JH3		
Occupancy OTE0019_York_060813_JH4		
Occupancy ratioOTE0019_York_060813_JH4		
Occupancy error scale OTE0019_York_060813_JH4		
Occupancy OTE0019_York_060813_JH5		
Occupancy ratioOTE0019_York_060813_JH5		
Occupancy error scale OTE0019_York_060813_JH5		
Occupancy OTE0019_York_060813_JH6		
Occupancy ratioOTE0019_York_060813_JH6		
Occupancy error scale OTE0019_York_060813_JH6		
Occupancy OTE0019_York_060813_JH7		
Occupancy ratioOTE0019_York_060813_JH7		
Occupancy error scale OTE0019_York_060813_JH7		
Occupancy OTE0019_York_060813_JH8		
Occupancy ratioOTE0019_York_060813_JH8		
Occupancy error scale OTE0019_York_060813_JH8		
Occupancy OTE0019_York_060813_JH9		
Occupancy ratioOTE0019_York_060813_JH9		
Occupancy error scale OTE0019_York_060813_JH9		
Occupancy QE0095_JHendy_JH1_130823142140		
Occupancy ratioQE0095_JHendy_JH1_130823142140		
Occupancy error scale QE0095_JHendy_JH1_130823142140		
Occupancy QE0095_JHendy_JH10_130824054009		
Occupancy ratioQE0095_JHendy_JH10_130824054009		
Occupancy error scale QE0095_JHendy_JH10_130824054009		
Occupancy QE0095_JHendy_JH11_130824082301		
Occupancy ratioQE0095_JHendy_JH11_130824082301		

Occupancy error scale QE0095_JHendy_JH11_130824082301		
Occupancy QE0095_JHendy_JH12_130824101054		
Occupancy ratioQE0095_JHendy_JH12_130824101054		
Occupancy error scale QE0095_JHendy_JH12_130824101054		
Occupancy QE0095_JHendy_JH13_130824115852		
Occupancy ratioQE0095_JHendy_JH13_130824115852		
Occupancy error scale QE0095_JHendy_JH13_130824115852		
Occupancy QE0095_JHendy_JH14_130824134650		
Occupancy ratioQE0095_JHendy_JH14_130824134650		
Occupancy error scale QE0095_JHendy_JH14_130824134650		
Occupancy QE0095_JHendy_JH15		
Occupancy ratioQE0095_JHendy_JH15		
Occupancy error scale QE0095_JHendy_JH15		
Occupancy QE0095_JHendy_JH16		
Occupancy ratioQE0095_JHendy_JH16		
Occupancy error scale QE0095_JHendy_JH16		
Occupancy QE0095_JHendy_JH17		
Occupancy ratioQE0095_JHendy_JH17		
Occupancy error scale QE0095_JHendy_JH17		
Occupancy QE0095_JHendy_JH18		
Occupancy ratioQE0095_JHendy_JH18		
Occupancy error scale QE0095_JHendy_JH18		
Occupancy QE0095_JHendy_JH19		
Occupancy ratioQE0095_JHendy_JH19		
Occupancy error scale QE0095_JHendy_JH19		
Occupancy QE0095_JHendy_JH2		
Occupancy ratioQE0095_JHendy_JH2		
Occupancy error scale QE0095_JHendy_JH2		
Occupancy QE0095_JHendy_JH20		
Occupancy ratioQE0095_JHendy_JH20		
Occupancy error scale QE0095_JHendy_JH20		
Occupancy QE0095_JHendy_JH3_130823160937		
Occupancy ratioQE0095_JHendy_JH3_130823160937		
Occupancy error scale QE0095_JHendy_JH3_130823160937		
Occupancy QE0095_JHendy_JH4_130823175743		

Occupancy ratioQE0095_JHendy_JH4_130823175743		
Occupancy error scale QE0095_JHendy_JH4_130823175743		
Occupancy QE0095_JHendy_JH5_130823194539		
Occupancy ratioQE0095_JHendy_JH5_130823194539		
Occupancy error scale QE0095_JHendy_JH5_130823194539		
Occupancy QE0095_JHendy_JH6_130823222831		
Occupancy ratioQE0095_JHendy_JH6_130823222831		
Occupancy error scale QE0095_JHendy_JH6_130823222831		
Occupancy QE0095_JHendy_JH7_130824001623		
Occupancy ratioQE0095_JHendy_JH7_130824001623		
Occupancy error scale QE0095_JHendy_JH7_130824001623		
Occupancy QE0095_JHendy_JH8_130824020419		
Occupancy ratioQE0095_JHendy_JH8_130824020419		
Occupancy error scale QE0095_JHendy_JH8_130824020419		
Occupancy QE0095_JHendy_JH9		
Occupancy ratioQE0095_JHendy_JH9		
Occupancy error scale QE0095_JHendy_JH9		
Occupancy QE0095_JHendy_JH9_130824035216		
Occupancy ratioQE0095_JHendy_JH9_130824035216		
Occupancy error scale QE0095_JHendy_JH9_130824035216		
Reverse		When marked with '+', this particular peptide was found to be part of a protein derived from the reversed part of the protein sequence database. These should be removed for further data analysis.
Potential contaminant		When marked with '+', this particular peptide was found to be part of a commonly occurring contaminant. These should be removed for further data analysis.
id		A unique (consecutive) identifier for each row in the site table, which is used to cross-link the information in this file with the information stored in the other files.
Protein group IDs		The identifier of the protein-group this peptide sequence is associated with, which can be used to look up the extended protein information in the file 'proteinGroups.txt'.<br>As a single peptide can be linked to multiple proteins (e.g. in the case of razor-proteins), multiple id's can be stored here separated by a semicolon.<br>As a protein can be identified by multiple peptides, the same id can be found in different rows.
Positions		The positions of the modifications in the protein amino acid sequence.
Position		The position of the modification in the protein amino acid sequence.
Peptide IDs		Identifier(s) of the associated peptide sequence(s) summary, which can be found in the file 'peptides.txt'.
Mod. peptide IDs		Identifier(s) of the associated peptide sequence(s) summary, which can be found in the file 'modificationSpecificPeptides.txt'.

Evidence IDs		Identifier(s) for analyzed peptide evidence associated with the protein group referenced against the evidence table.
MS/MS IDs		The identifiers of the MS/MS scans identifying this peptide, referenced against the msms table.
Best localization evidence ID		
Best localization MS/MS ID		
Best localization raw file		
Best localization scan number		
Best score evidence ID		
Best score MS/MS ID		
Best score raw file		
Best score scan number		
Best PEP evidence ID		
Best PEP MS/MS ID		
Best PEP raw file		
Best PEP scan number		

Protein groups

The Protein Groups table contains information on the identified proteins in the processed raw-files. Each single row contains the group of proteins that could be reconstructed from a set of peptides.

Name	Separator	Description
Protein IDs		Identifier(s) of protein(s) contained in the protein group. They are sorted by number of identified peptides in descending order.
Majority protein IDs		These are the IDs of those proteins that have at least half of the peptides that the leading protein has.
Peptide counts (all)		Number of peptides associated with each protein in protein group, occurring in the order as the protein IDs occur in the 'Protein IDs' column. Here distinct peptide sequences are counted. Modified forms or different charges are counted as one peptide.
Peptide counts (razor+unique)		Number of peptides associated with each protein in protein group, occurring in the order as the protein IDs occur in the 'Protein IDs' column. Here distinct peptide sequences are counted. Modified forms or different charges are counted as one peptide.
Peptide counts (unique)		Number of peptides associated with each protein in protein group, occurring in the order as the protein IDs occur in the 'Protein IDs' column. Here distinct peptide sequences are counted. Modified forms or different charges are counted as one peptide.
Fasta headers		Fasta headers(s) of protein(s) contained within the group.
Number of proteins		Number of proteins contained within the group. This corresponds to the number of entries in the column 'Protein IDs'.
Peptides		The total number of peptide sequences associated with the protein group (i.e. for all the proteins in the group).
Razor + unique peptides		The total number of razor + unique peptides associated with the protein group (i.e. these peptides are shared with another protein group).
Unique peptides		The total number of unique peptides associated with the protein group (i.e. these peptides are not shared with another protein group).
Peptides OTE0019_York_060813_JH1		Number of peptides (distinct peptide sequences) in experiment OTE0019_York_060813_JH1
Peptides OTE0019_York_060813_JH10		Number of peptides (distinct peptide sequences) in experiment OTE0019_York_060813_JH10
Peptides OTE0019_York_060813_JH11		Number of peptides (distinct peptide sequences) in experiment OTE0019_York_060813_JH11
Peptides OTE0019_York_060813_JH12		Number of peptides (distinct peptide sequences) in experiment OTE0019_York_060813_JH12
Peptides OTE0019_York_060813_JH13		Number of peptides (distinct peptide sequences) in experiment OTE0019_York_060813_JH13
Peptides OTE0019_York_060813_JH14		Number of peptides (distinct peptide sequences) in experiment OTE0019_York_060813_JH14
Peptides OTE0019_York_060813_JH15		Number of peptides (distinct peptide sequences) in experiment OTE0019_York_060813_JH15
Peptides OTE0019_York_060813_JH16		Number of peptides (distinct peptide sequences) in experiment OTE0019_York_060813_JH16
Peptides OTE0019_York_060813_JH17		Number of peptides (distinct peptide sequences) in experiment OTE0019_York_060813_JH17
Peptides OTE0019_York_060813_JH18		Number of peptides (distinct peptide sequences) in experiment OTE0019_York_060813_JH18
Peptides OTE0019_York_060813_JH19		Number of peptides (distinct peptide sequences) in experiment OTE0019_York_060813_JH19
Peptides OTE0019_York_060813_JH2		Number of peptides (distinct peptide sequences) in experiment OTE0019_York_060813_JH2
Peptides OTE0019_York_060813_JH20		Number of peptides (distinct peptide sequences) in experiment OTE0019_York_060813_JH20
Peptides OTE0019_York_060813_JH3		Number of peptides (distinct peptide sequences) in experiment OTE0019_York_060813_JH3
Peptides OTE0019_York_060813_JH4		Number of peptides (distinct peptide sequences) in experiment OTE0019_York_060813_JH4
Peptides OTE0019_York_060813_JH5		Number of peptides (distinct peptide sequences) in experiment OTE0019_York_060813_JH5
Peptides OTE0019_York_060813_JH6		Number of peptides (distinct peptide sequences) in experiment OTE0019_York_060813_JH6
Peptides OTE0019_York_060813_JH7		Number of peptides (distinct peptide sequences) in experiment OTE0019_York_060813_JH7

Peptides OTE0019_York_060813_JH8		Number of peptides (distinct peptide sequences) in experiment OTE0019_York_060813_JH8
Peptides OTE0019_York_060813_JH9		Number of peptides (distinct peptide sequences) in experiment OTE0019_York_060813_JH9
Peptides QE0095_JHendy_JH1_130823142140		Number of peptides (distinct peptide sequences) in experiment QE0095_JHendy_JH1_130823142140
Peptides QE0095_JHendy_JH10_130824054009		Number of peptides (distinct peptide sequences) in experiment QE0095_JHendy_JH10_130824054009
Peptides QE0095_JHendy_JH11_130824082301		Number of peptides (distinct peptide sequences) in experiment QE0095_JHendy_JH11_130824082301
Peptides QE0095_JHendy_JH12_130824101054		Number of peptides (distinct peptide sequences) in experiment QE0095_JHendy_JH12_130824101054
Peptides QE0095_JHendy_JH13_130824115852		Number of peptides (distinct peptide sequences) in experiment QE0095_JHendy_JH13_130824115852
Peptides QE0095_JHendy_JH14_130824134650		Number of peptides (distinct peptide sequences) in experiment QE0095_JHendy_JH14_130824134650
Peptides QE0095_JHendy_JH15		Number of peptides (distinct peptide sequences) in experiment QE0095_JHendy_JH15
Peptides QE0095_JHendy_JH16		Number of peptides (distinct peptide sequences) in experiment QE0095_JHendy_JH16
Peptides QE0095_JHendy_JH17		Number of peptides (distinct peptide sequences) in experiment QE0095_JHendy_JH17
Peptides QE0095_JHendy_JH18		Number of peptides (distinct peptide sequences) in experiment QE0095_JHendy_JH18
Peptides QE0095_JHendy_JH19		Number of peptides (distinct peptide sequences) in experiment QE0095_JHendy_JH19
Peptides QE0095_JHendy_JH2		Number of peptides (distinct peptide sequences) in experiment QE0095_JHendy_JH2
Peptides QE0095_JHendy_JH20		Number of peptides (distinct peptide sequences) in experiment QE0095_JHendy_JH20
Peptides QE0095_JHendy_JH3_130823160937		Number of peptides (distinct peptide sequences) in experiment QE0095_JHendy_JH3_130823160937
Peptides QE0095_JHendy_JH4_130823175743		Number of peptides (distinct peptide sequences) in experiment QE0095_JHendy_JH4_130823175743
Peptides QE0095_JHendy_JH5_130823194539		Number of peptides (distinct peptide sequences) in experiment QE0095_JHendy_JH5_130823194539
Peptides QE0095_JHendy_JH6_130823222831		Number of peptides (distinct peptide sequences) in experiment QE0095_JHendy_JH6_130823222831
Peptides QE0095_JHendy_JH7_130824001623		Number of peptides (distinct peptide sequences) in experiment QE0095_JHendy_JH7_130824001623
Peptides QE0095_JHendy_JH8_130824020419		Number of peptides (distinct peptide sequences) in experiment QE0095_JHendy_JH8_130824020419
Peptides QE0095_JHendy_JH9		Number of peptides (distinct peptide sequences) in experiment QE0095_JHendy_JH9
Peptides QE0095_JHendy_JH9_130824035216		Number of peptides (distinct peptide sequences) in experiment QE0095_JHendy_JH9_130824035216
Razor + unique peptides OTE0019_York_060813_JH1		Number of razor + unique peptides (distinct peptide sequences) in experiment OTE0019_York_060813_JH1
Razor + unique peptides OTE0019_York_060813_JH10		Number of razor + unique peptides (distinct peptide sequences) in experiment OTE0019_York_060813_JH10
Razor + unique peptides OTE0019_York_060813_JH11		Number of razor + unique peptides (distinct peptide sequences) in experiment OTE0019_York_060813_JH11
Razor + unique peptides OTE0019_York_060813_JH12		Number of razor + unique peptides (distinct peptide sequences) in experiment OTE0019_York_060813_JH12
Razor + unique peptides OTE0019_York_060813_JH13		Number of razor + unique peptides (distinct peptide sequences) in experiment OTE0019_York_060813_JH13
Razor + unique peptides OTE0019_York_060813_JH14		Number of razor + unique peptides (distinct peptide sequences) in experiment OTE0019_York_060813_JH14
Razor + unique peptides OTE0019_York_060813_JH15		Number of razor + unique peptides (distinct peptide sequences) in experiment OTE0019_York_060813_JH15
Razor + unique peptides OTE0019_York_060813_JH16		Number of razor + unique peptides (distinct peptide sequences) in experiment OTE0019_York_060813_JH16
Razor + unique peptides OTE0019_York_060813_JH17		Number of razor + unique peptides (distinct peptide sequences) in experiment OTE0019_York_060813_JH17
Razor + unique peptides OTE0019_York_060813_JH18		Number of razor + unique peptides (distinct peptide sequences) in experiment OTE0019_York_060813_JH18

Razor + unique peptides OTE0019_York_060813_JH19		Number of razor + unique peptides (distinct peptide sequences) in experiment OTE0019_York_060813_JH19
Razor + unique peptides OTE0019_York_060813_JH2		Number of razor + unique peptides (distinct peptide sequences) in experiment OTE0019_York_060813_JH2
Razor + unique peptides OTE0019_York_060813_JH20		Number of razor + unique peptides (distinct peptide sequences) in experiment OTE0019_York_060813_JH20
Razor + unique peptides OTE0019_York_060813_JH3		Number of razor + unique peptides (distinct peptide sequences) in experiment OTE0019_York_060813_JH3
Razor + unique peptides OTE0019_York_060813_JH4		Number of razor + unique peptides (distinct peptide sequences) in experiment OTE0019_York_060813_JH4
Razor + unique peptides OTE0019_York_060813_JH5		Number of razor + unique peptides (distinct peptide sequences) in experiment OTE0019_York_060813_JH5
Razor + unique peptides OTE0019_York_060813_JH6		Number of razor + unique peptides (distinct peptide sequences) in experiment OTE0019_York_060813_JH6
Razor + unique peptides OTE0019_York_060813_JH7		Number of razor + unique peptides (distinct peptide sequences) in experiment OTE0019_York_060813_JH7
Razor + unique peptides OTE0019_York_060813_JH8		Number of razor + unique peptides (distinct peptide sequences) in experiment OTE0019_York_060813_JH8
Razor + unique peptides OTE0019_York_060813_JH9		Number of razor + unique peptides (distinct peptide sequences) in experiment OTE0019_York_060813_JH9
Razor + unique peptides QE0095_JHendy_JH1_130823142140		Number of razor + unique peptides (distinct peptide sequences) in experiment QE0095_JHendy_JH1_130823142140
Razor + unique peptides QE0095_JHendy_JH10_130824054009		Number of razor + unique peptides (distinct peptide sequences) in experiment QE0095_JHendy_JH10_130824054009
Razor + unique peptides QE0095_JHendy_JH11_130824082301		Number of razor + unique peptides (distinct peptide sequences) in experiment QE0095_JHendy_JH11_130824082301
Razor + unique peptides QE0095_JHendy_JH12_130824101054		Number of razor + unique peptides (distinct peptide sequences) in experiment QE0095_JHendy_JH12_130824101054
Razor + unique peptides QE0095_JHendy_JH13_130824115852		Number of razor + unique peptides (distinct peptide sequences) in experiment QE0095_JHendy_JH13_130824115852
Razor + unique peptides QE0095_JHendy_JH14_130824134650		Number of razor + unique peptides (distinct peptide sequences) in experiment QE0095_JHendy_JH14_130824134650
Razor + unique peptides QE0095_JHendy_JH15		Number of razor + unique peptides (distinct peptide sequences) in experiment QE0095_JHendy_JH15
Razor + unique peptides QE0095_JHendy_JH16		Number of razor + unique peptides (distinct peptide sequences) in experiment QE0095_JHendy_JH16
Razor + unique peptides QE0095_JHendy_JH17		Number of razor + unique peptides (distinct peptide sequences) in experiment QE0095_JHendy_JH17
Razor + unique peptides QE0095_JHendy_JH18		Number of razor + unique peptides (distinct peptide sequences) in experiment QE0095_JHendy_JH18
Razor + unique peptides QE0095_JHendy_JH19		Number of razor + unique peptides (distinct peptide sequences) in experiment QE0095_JHendy_JH19
Razor + unique peptides QE0095_JHendy_JH2		Number of razor + unique peptides (distinct peptide sequences) in experiment QE0095_JHendy_JH2
Razor + unique peptides QE0095_JHendy_JH20		Number of razor + unique peptides (distinct peptide sequences) in experiment QE0095_JHendy_JH20
Razor + unique peptides QE0095_JHendy_JH3_130823160937		Number of razor + unique peptides (distinct peptide sequences) in experiment QE0095_JHendy_JH3_130823160937
Razor + unique peptides QE0095_JHendy_JH4_130823175743		Number of razor + unique peptides (distinct peptide sequences) in experiment QE0095_JHendy_JH4_130823175743
Razor + unique peptides QE0095_JHendy_JH5_130823194539		Number of razor + unique peptides (distinct peptide sequences) in experiment QE0095_JHendy_JH5_130823194539
Razor + unique peptides QE0095_JHendy_JH6_130823222831		Number of razor + unique peptides (distinct peptide sequences) in experiment QE0095_JHendy_JH6_130823222831
Razor + unique peptides QE0095_JHendy_JH7_130824001623		Number of razor + unique peptides (distinct peptide sequences) in experiment QE0095_JHendy_JH7_130824001623
Razor + unique peptides QE0095_JHendy_JH8_130824020419		Number of razor + unique peptides (distinct peptide sequences) in experiment QE0095_JHendy_JH8_130824020419
Razor + unique peptides QE0095_JHendy_JH9		Number of razor + unique peptides (distinct peptide sequences) in experiment QE0095_JHendy_JH9
Razor + unique peptides QE0095_JHendy_JH9_130824035216		Number of razor + unique peptides (distinct peptide sequences) in experiment QE0095_JHendy_JH9_130824035216
Unique peptides OTE0019_York_060813_JH1		Number of unique peptides (distinct peptide sequences) in experiment OTE0019_York_060813_JH1
Unique peptides OTE0019_York_060813_JH10		Number of unique peptides (distinct peptide sequences) in experiment OTE0019_York_060813_JH10

Unique peptides OTE0019_York_060813_JH11		Number of unique peptides (distinct peptide sequences) in experiment OTE0019_York_060813_JH11
Unique peptides OTE0019_York_060813_JH12		Number of unique peptides (distinct peptide sequences) in experiment OTE0019_York_060813_JH12
Unique peptides OTE0019_York_060813_JH13		Number of unique peptides (distinct peptide sequences) in experiment OTE0019_York_060813_JH13
Unique peptides OTE0019_York_060813_JH14		Number of unique peptides (distinct peptide sequences) in experiment OTE0019_York_060813_JH14
Unique peptides OTE0019_York_060813_JH15		Number of unique peptides (distinct peptide sequences) in experiment OTE0019_York_060813_JH15
Unique peptides OTE0019_York_060813_JH16		Number of unique peptides (distinct peptide sequences) in experiment OTE0019_York_060813_JH16
Unique peptides OTE0019_York_060813_JH17		Number of unique peptides (distinct peptide sequences) in experiment OTE0019_York_060813_JH17
Unique peptides OTE0019_York_060813_JH18		Number of unique peptides (distinct peptide sequences) in experiment OTE0019_York_060813_JH18
Unique peptides OTE0019_York_060813_JH19		Number of unique peptides (distinct peptide sequences) in experiment OTE0019_York_060813_JH19
Unique peptides OTE0019_York_060813_JH2		Number of unique peptides (distinct peptide sequences) in experiment OTE0019_York_060813_JH2
Unique peptides OTE0019_York_060813_JH20		Number of unique peptides (distinct peptide sequences) in experiment OTE0019_York_060813_JH20
Unique peptides OTE0019_York_060813_JH3		Number of unique peptides (distinct peptide sequences) in experiment OTE0019_York_060813_JH3
Unique peptides OTE0019_York_060813_JH4		Number of unique peptides (distinct peptide sequences) in experiment OTE0019_York_060813_JH4
Unique peptides OTE0019_York_060813_JH5		Number of unique peptides (distinct peptide sequences) in experiment OTE0019_York_060813_JH5
Unique peptides OTE0019_York_060813_JH6		Number of unique peptides (distinct peptide sequences) in experiment OTE0019_York_060813_JH6
Unique peptides OTE0019_York_060813_JH7		Number of unique peptides (distinct peptide sequences) in experiment OTE0019_York_060813_JH7
Unique peptides OTE0019_York_060813_JH8		Number of unique peptides (distinct peptide sequences) in experiment OTE0019_York_060813_JH8
Unique peptides OTE0019_York_060813_JH9		Number of unique peptides (distinct peptide sequences) in experiment OTE0019_York_060813_JH9
Unique peptides QE0095_JHendy_JH1_130823142140		Number of unique peptides (distinct peptide sequences) in experiment QE0095_JHendy_JH1_130823142140
Unique peptides QE0095_JHendy_JH10_130824054009		Number of unique peptides (distinct peptide sequences) in experiment QE0095_JHendy_JH10_130824054009
Unique peptides QE0095_JHendy_JH11_130824082301		Number of unique peptides (distinct peptide sequences) in experiment QE0095_JHendy_JH11_130824082301
Unique peptides QE0095_JHendy_JH12_130824101054		Number of unique peptides (distinct peptide sequences) in experiment QE0095_JHendy_JH12_130824101054
Unique peptides QE0095_JHendy_JH13_130824115852		Number of unique peptides (distinct peptide sequences) in experiment QE0095_JHendy_JH13_130824115852
Unique peptides QE0095_JHendy_JH14_130824134650		Number of unique peptides (distinct peptide sequences) in experiment QE0095_JHendy_JH14_130824134650
Unique peptides QE0095_JHendy_JH15		Number of unique peptides (distinct peptide sequences) in experiment QE0095_JHendy_JH15
Unique peptides QE0095_JHendy_JH16		Number of unique peptides (distinct peptide sequences) in experiment QE0095_JHendy_JH16
Unique peptides QE0095_JHendy_JH17		Number of unique peptides (distinct peptide sequences) in experiment QE0095_JHendy_JH17
Unique peptides QE0095_JHendy_JH18		Number of unique peptides (distinct peptide sequences) in experiment QE0095_JHendy_JH18
Unique peptides QE0095_JHendy_JH19		Number of unique peptides (distinct peptide sequences) in experiment QE0095_JHendy_JH19
Unique peptides QE0095_JHendy_JH2		Number of unique peptides (distinct peptide sequences) in experiment QE0095_JHendy_JH2
Unique peptides QE0095_JHendy_JH20		Number of unique peptides (distinct peptide sequences) in experiment QE0095_JHendy_JH20
Unique peptides QE0095_JHendy_JH3_130823160937		Number of unique peptides (distinct peptide sequences) in experiment QE0095_JHendy_JH3_130823160937
Unique peptides QE0095_JHendy_JH4_130823175743		Number of unique peptides (distinct peptide sequences) in experiment QE0095_JHendy_JH4_130823175743
Unique peptides QE0095_JHendy_JH5_130823194539		Number of unique peptides (distinct peptide sequences) in experiment QE0095_JHendy_JH5_130823194539

Unique peptides QE0095_JHendy_JH6_130823222831		Number of unique peptides (distinct peptide sequences) in experiment QE0095_JHendy_JH6_130823222831
Unique peptides QE0095_JHendy_JH7_130824001623		Number of unique peptides (distinct peptide sequences) in experiment QE0095_JHendy_JH7_130824001623
Unique peptides QE0095_JHendy_JH8_130824020419		Number of unique peptides (distinct peptide sequences) in experiment QE0095_JHendy_JH8_130824020419
Unique peptides QE0095_JHendy_JH9		Number of unique peptides (distinct peptide sequences) in experiment QE0095_JHendy_JH9
Unique peptides QE0095_JHendy_JH9_130824035216		Number of unique peptides (distinct peptide sequences) in experiment QE0095_JHendy_JH9_130824035216
Sequence coverage [%]		Percentage of the sequence that is covered by the identified peptides of the best protein sequence contained in the group.
Unique + razor sequence coverage [%]		Percentage of the sequence that is covered by the identified unique and razor peptides of the best protein sequence contained in the group.
Unique sequence coverage [%]		Percentage of the sequence that is covered by the identified unique peptides of the best protein sequence contained in the group.
Mol. weight [kDa]		Molecular weight of the leading protein sequence contained in the protein group.
Sequence length		The length of the leading protein sequence contained in the group.
Sequence lengths		The length of all sequences of the proteins contained in the group.
Q-value		This is the ratio of reverse to forward protein groups.
Score		Protein score which is derived from peptide posterior error probabilities.
Sequence coverage OTE0019_York_060813_JH1 [%]		Percentage of the sequence that is covered by the identified peptides in this sample of the longest protein sequence contained within the group.
Sequence coverage OTE0019_York_060813_JH10 [%]		Percentage of the sequence that is covered by the identified peptides in this sample of the longest protein sequence contained within the group.
Sequence coverage OTE0019_York_060813_JH11 [%]		Percentage of the sequence that is covered by the identified peptides in this sample of the longest protein sequence contained within the group.
Sequence coverage OTE0019_York_060813_JH12 [%]		Percentage of the sequence that is covered by the identified peptides in this sample of the longest protein sequence contained within the group.
Sequence coverage OTE0019_York_060813_JH13 [%]		Percentage of the sequence that is covered by the identified peptides in this sample of the longest protein sequence contained within the group.
Sequence coverage OTE0019_York_060813_JH14 [%]		Percentage of the sequence that is covered by the identified peptides in this sample of the longest protein sequence contained within the group.
Sequence coverage OTE0019_York_060813_JH15 [%]		Percentage of the sequence that is covered by the identified peptides in this sample of the longest protein sequence contained within the group.
Sequence coverage OTE0019_York_060813_JH16 [%]		Percentage of the sequence that is covered by the identified peptides in this sample of the longest protein sequence contained within the group.
Sequence coverage OTE0019_York_060813_JH17 [%]		Percentage of the sequence that is covered by the identified peptides in this sample of the longest protein sequence contained within the group.
Sequence coverage OTE0019_York_060813_JH18 [%]		Percentage of the sequence that is covered by the identified peptides in this sample of the longest protein sequence contained within the group.
Sequence coverage OTE0019_York_060813_JH19 [%]		Percentage of the sequence that is covered by the identified peptides in this sample of the longest protein sequence contained within the group.
Sequence coverage OTE0019_York_060813_JH2 [%]		Percentage of the sequence that is covered by the identified peptides in this sample of the longest protein sequence contained within the group.
Sequence coverage OTE0019_York_060813_JH20 [%]		Percentage of the sequence that is covered by the identified peptides in this sample of the longest protein sequence contained within the group.
Sequence coverage OTE0019_York_060813_JH3 [%]		Percentage of the sequence that is covered by the identified peptides in this sample of the longest protein sequence contained within the group.
Sequence coverage OTE0019_York_060813_JH4 [%]		Percentage of the sequence that is covered by the identified peptides in this sample of the longest protein sequence contained within the group.
Sequence coverage OTE0019_York_060813_JH5 [%]		Percentage of the sequence that is covered by the identified peptides in this sample of the longest protein sequence contained within the group.

Sequence coverage OTE0019_York_060813_JH6 [%]		Percentage of the sequence that is covered by the identified peptides in this sample of the longest protein sequence contained within the group.
Sequence coverage OTE0019_York_060813_JH7 [%]		Percentage of the sequence that is covered by the identified peptides in this sample of the longest protein sequence contained within the group.
Sequence coverage OTE0019_York_060813_JH8 [%]		Percentage of the sequence that is covered by the identified peptides in this sample of the longest protein sequence contained within the group.
Sequence coverage OTE0019_York_060813_JH9 [%]		Percentage of the sequence that is covered by the identified peptides in this sample of the longest protein sequence contained within the group.
Sequence coverage QE0095_JHendy_JH1_130823142140 [%]		Percentage of the sequence that is covered by the identified peptides in this sample of the longest protein sequence contained within the group.
Sequence coverage QE0095_JHendy_JH10_130824054009 [%]		Percentage of the sequence that is covered by the identified peptides in this sample of the longest protein sequence contained within the group.
Sequence coverage QE0095_JHendy_JH11_130824082301 [%]		Percentage of the sequence that is covered by the identified peptides in this sample of the longest protein sequence contained within the group.
Sequence coverage QE0095_JHendy_JH12_130824101054 [%]		Percentage of the sequence that is covered by the identified peptides in this sample of the longest protein sequence contained within the group.
Sequence coverage QE0095_JHendy_JH13_130824115852 [%]		Percentage of the sequence that is covered by the identified peptides in this sample of the longest protein sequence contained within the group.
Sequence coverage QE0095_JHendy_JH14_130824134650 [%]		Percentage of the sequence that is covered by the identified peptides in this sample of the longest protein sequence contained within the group.
Sequence coverage QE0095_JHendy_JH15 [%]		Percentage of the sequence that is covered by the identified peptides in this sample of the longest protein sequence contained within the group.
Sequence coverage QE0095_JHendy_JH16 [%]		Percentage of the sequence that is covered by the identified peptides in this sample of the longest protein sequence contained within the group.
Sequence coverage QE0095_JHendy_JH17 [%]		Percentage of the sequence that is covered by the identified peptides in this sample of the longest protein sequence contained within the group.
Sequence coverage QE0095_JHendy_JH18 [%]		Percentage of the sequence that is covered by the identified peptides in this sample of the longest protein sequence contained within the group.
Sequence coverage QE0095_JHendy_JH19 [%]		Percentage of the sequence that is covered by the identified peptides in this sample of the longest protein sequence contained within the group.
Sequence coverage QE0095_JHendy_JH2 [%]		Percentage of the sequence that is covered by the identified peptides in this sample of the longest protein sequence contained within the group.
Sequence coverage QE0095_JHendy_JH20 [%]		Percentage of the sequence that is covered by the identified peptides in this sample of the longest protein sequence contained within the group.
Sequence coverage QE0095_JHendy_JH3_130823160937 [%]		Percentage of the sequence that is covered by the identified peptides in this sample of the longest protein sequence contained within the group.
Sequence coverage QE0095_JHendy_JH4_130823175743 [%]		Percentage of the sequence that is covered by the identified peptides in this sample of the longest protein sequence contained within the group.
Sequence coverage QE0095_JHendy_JH5_130823194539 [%]		Percentage of the sequence that is covered by the identified peptides in this sample of the longest protein sequence contained within the group.
Sequence coverage QE0095_JHendy_JH6_130823222831 [%]		Percentage of the sequence that is covered by the identified peptides in this sample of the longest protein sequence contained within the group.
Sequence coverage QE0095_JHendy_JH7_130824001623 [%]		Percentage of the sequence that is covered by the identified peptides in this sample of the longest protein sequence contained within the group.
Sequence coverage QE0095_JHendy_JH8_130824020419 [%]		Percentage of the sequence that is covered by the identified peptides in this sample of the longest protein sequence contained within the group.
Sequence coverage QE0095_JHendy_JH9 [%]		Percentage of the sequence that is covered by the identified peptides in this sample of the longest protein sequence contained within the group.
Sequence coverage QE0095_JHendy_JH9_130824035216 [%]		Percentage of the sequence that is covered by the identified peptides in this sample of the longest protein sequence contained within the group.
Intensity		Summed up eXtracted Ion Current (XIC) of all isotopic clusters associated with the identified AA sequence. In case of a labeled experiment this is the total intensity of all the isotopic patterns in the label cluster.

Intensity OTE0019_York_060813_JH1		Summed up eXtracted Ion Current (XIC) of all isotopic clusters associated with the identified AA sequence. In case of a labeled experiment this is the total intensity of all the isotopic patterns in the label cluster.
Intensity OTE0019_York_060813_JH10		Summed up eXtracted Ion Current (XIC) of all isotopic clusters associated with the identified AA sequence. In case of a labeled experiment this is the total intensity of all the isotopic patterns in the label cluster.
Intensity OTE0019_York_060813_JH11		Summed up eXtracted Ion Current (XIC) of all isotopic clusters associated with the identified AA sequence. In case of a labeled experiment this is the total intensity of all the isotopic patterns in the label cluster.
Intensity OTE0019_York_060813_JH12		Summed up eXtracted Ion Current (XIC) of all isotopic clusters associated with the identified AA sequence. In case of a labeled experiment this is the total intensity of all the isotopic patterns in the label cluster.
Intensity OTE0019_York_060813_JH13		Summed up eXtracted Ion Current (XIC) of all isotopic clusters associated with the identified AA sequence. In case of a labeled experiment this is the total intensity of all the isotopic patterns in the label cluster.
Intensity OTE0019_York_060813_JH14		Summed up eXtracted Ion Current (XIC) of all isotopic clusters associated with the identified AA sequence. In case of a labeled experiment this is the total intensity of all the isotopic patterns in the label cluster.
Intensity OTE0019_York_060813_JH15		Summed up eXtracted Ion Current (XIC) of all isotopic clusters associated with the identified AA sequence. In case of a labeled experiment this is the total intensity of all the isotopic patterns in the label cluster.
Intensity OTE0019_York_060813_JH16		Summed up eXtracted Ion Current (XIC) of all isotopic clusters associated with the identified AA sequence. In case of a labeled experiment this is the total intensity of all the isotopic patterns in the label cluster.
Intensity OTE0019_York_060813_JH17		Summed up eXtracted Ion Current (XIC) of all isotopic clusters associated with the identified AA sequence. In case of a labeled experiment this is the total intensity of all the isotopic patterns in the label cluster.
Intensity OTE0019_York_060813_JH18		Summed up eXtracted Ion Current (XIC) of all isotopic clusters associated with the identified AA sequence. In case of a labeled experiment this is the total intensity of all the isotopic patterns in the label cluster.
Intensity OTE0019_York_060813_JH19		Summed up eXtracted Ion Current (XIC) of all isotopic clusters associated with the identified AA sequence. In case of a labeled experiment this is the total intensity of all the isotopic patterns in the label cluster.
Intensity OTE0019_York_060813_JH2		Summed up eXtracted Ion Current (XIC) of all isotopic clusters associated with the identified AA sequence. In case of a labeled experiment this is the total intensity of all the isotopic patterns in the label cluster.
Intensity OTE0019_York_060813_JH20		Summed up eXtracted Ion Current (XIC) of all isotopic clusters associated with the identified AA sequence. In case of a labeled experiment this is the total intensity of all the isotopic patterns in the label cluster.
Intensity OTE0019_York_060813_JH3		Summed up eXtracted Ion Current (XIC) of all isotopic clusters associated with the identified AA sequence. In case of a labeled experiment this is the total intensity of all the isotopic patterns in the label cluster.
Intensity OTE0019_York_060813_JH4		Summed up eXtracted Ion Current (XIC) of all isotopic clusters associated with the identified AA sequence. In case of a labeled experiment this is the total intensity of all the isotopic patterns in the label cluster.
Intensity OTE0019_York_060813_JH5		Summed up eXtracted Ion Current (XIC) of all isotopic clusters associated with the identified AA sequence. In case of a labeled experiment this is the total intensity of all the isotopic patterns in the label cluster.
Intensity OTE0019_York_060813_JH6		Summed up eXtracted Ion Current (XIC) of all isotopic clusters associated with the identified AA sequence. In case of a labeled experiment this is the total intensity of all the isotopic patterns in the label cluster.
Intensity OTE0019_York_060813_JH7		Summed up eXtracted Ion Current (XIC) of all isotopic clusters associated with the identified AA sequence. In case of a labeled experiment this is the total intensity of all the isotopic patterns in the label cluster.
Intensity OTE0019_York_060813_JH8		Summed up eXtracted Ion Current (XIC) of all isotopic clusters associated with the identified AA sequence. In case of a labeled experiment this is the total intensity of all the isotopic patterns in the label cluster.
Intensity OTE0019_York_060813_JH9		Summed up eXtracted Ion Current (XIC) of all isotopic clusters associated with the identified AA sequence. In case of a labeled experiment this is the total intensity of all the isotopic patterns in the label cluster.
Intensity QE0095_JHendy_JH1_130823142140		Summed up eXtracted Ion Current (XIC) of all isotopic clusters associated with the identified AA sequence. In case of a labeled experiment this is the total intensity of all the isotopic patterns in the label cluster.

Intensity QE0095_JHendy_JH10_130824054009		Summed up eXtracted Ion Current (XIC) of all isotopic clusters associated with the identified AA sequence. In case of a labeled experiment this is the total intensity of all the isotopic patterns in the label cluster.
Intensity QE0095_JHendy_JH11_130824082301		Summed up eXtracted Ion Current (XIC) of all isotopic clusters associated with the identified AA sequence. In case of a labeled experiment this is the total intensity of all the isotopic patterns in the label cluster.
Intensity QE0095_JHendy_JH12_130824101054		Summed up eXtracted Ion Current (XIC) of all isotopic clusters associated with the identified AA sequence. In case of a labeled experiment this is the total intensity of all the isotopic patterns in the label cluster.
Intensity QE0095_JHendy_JH13_130824115852		Summed up eXtracted Ion Current (XIC) of all isotopic clusters associated with the identified AA sequence. In case of a labeled experiment this is the total intensity of all the isotopic patterns in the label cluster.
Intensity QE0095_JHendy_JH14_130824134650		Summed up eXtracted Ion Current (XIC) of all isotopic clusters associated with the identified AA sequence. In case of a labeled experiment this is the total intensity of all the isotopic patterns in the label cluster.
Intensity QE0095_JHendy_JH15		Summed up eXtracted Ion Current (XIC) of all isotopic clusters associated with the identified AA sequence. In case of a labeled experiment this is the total intensity of all the isotopic patterns in the label cluster.
Intensity QE0095_JHendy_JH16		Summed up eXtracted Ion Current (XIC) of all isotopic clusters associated with the identified AA sequence. In case of a labeled experiment this is the total intensity of all the isotopic patterns in the label cluster.
Intensity QE0095_JHendy_JH17		Summed up eXtracted Ion Current (XIC) of all isotopic clusters associated with the identified AA sequence. In case of a labeled experiment this is the total intensity of all the isotopic patterns in the label cluster.
Intensity QE0095_JHendy_JH18		Summed up eXtracted Ion Current (XIC) of all isotopic clusters associated with the identified AA sequence. In case of a labeled experiment this is the total intensity of all the isotopic patterns in the label cluster.
Intensity QE0095_JHendy_JH19		Summed up eXtracted Ion Current (XIC) of all isotopic clusters associated with the identified AA sequence. In case of a labeled experiment this is the total intensity of all the isotopic patterns in the label cluster.
Intensity QE0095_JHendy_JH2		Summed up eXtracted Ion Current (XIC) of all isotopic clusters associated with the identified AA sequence. In case of a labeled experiment this is the total intensity of all the isotopic patterns in the label cluster.
Intensity QE0095_JHendy_JH20		Summed up eXtracted Ion Current (XIC) of all isotopic clusters associated with the identified AA sequence. In case of a labeled experiment this is the total intensity of all the isotopic patterns in the label cluster.
Intensity QE0095_JHendy_JH3_130823160937		Summed up eXtracted Ion Current (XIC) of all isotopic clusters associated with the identified AA sequence. In case of a labeled experiment this is the total intensity of all the isotopic patterns in the label cluster.
Intensity QE0095_JHendy_JH4_130823175743		Summed up eXtracted Ion Current (XIC) of all isotopic clusters associated with the identified AA sequence. In case of a labeled experiment this is the total intensity of all the isotopic patterns in the label cluster.
Intensity QE0095_JHendy_JH5_130823194539		Summed up eXtracted Ion Current (XIC) of all isotopic clusters associated with the identified AA sequence. In case of a labeled experiment this is the total intensity of all the isotopic patterns in the label cluster.
Intensity QE0095_JHendy_JH6_130823222831		Summed up eXtracted Ion Current (XIC) of all isotopic clusters associated with the identified AA sequence. In case of a labeled experiment this is the total intensity of all the isotopic patterns in the label cluster.
Intensity QE0095_JHendy_JH7_130824001623		Summed up eXtracted Ion Current (XIC) of all isotopic clusters associated with the identified AA sequence. In case of a labeled experiment this is the total intensity of all the isotopic patterns in the label cluster.
Intensity QE0095_JHendy_JH8_130824020419		Summed up eXtracted Ion Current (XIC) of all isotopic clusters associated with the identified AA sequence. In case of a labeled experiment this is the total intensity of all the isotopic patterns in the label cluster.
Intensity QE0095_JHendy_JH9		Summed up eXtracted Ion Current (XIC) of all isotopic clusters associated with the identified AA sequence. In case of a labeled experiment this is the total intensity of all the isotopic patterns in the label cluster.
Intensity QE0095_JHendy_JH9_130824035216		Summed up eXtracted Ion Current (XIC) of all isotopic clusters associated with the identified AA sequence. In case of a labeled experiment this is the total intensity of all the isotopic patterns in the label cluster.
MS/MS count		
Only identified by site		When marked with '+', this particular protein group was identified only by a modification site.

Reverse		When marked with '+', this particular protein group contains no protein, made up of at least 50% of the peptides of the leading protein, with a peptide derived from the reversed part of the decoy database. These should be removed for further data analysis. The 50% rule is in place to prevent spurious protein hits to erroneously flag the protein group as reverse.
Potential contaminant		When marked with '+', this particular protein group was found to be a commonly occurring contaminant. These should be removed for further data analysis.
id		A unique (consecutive) identifier for each row in the proteinGroups table, which is used to cross-link the information in this file with the information stored in the other files.
Peptide IDs		Identifier(s) of the associated peptide sequence(s) summary, which can be found in the file 'peptides.txt'.
Peptide is razor		Indicates for each peptide ID if it is a razor or group unique peptide (true) or a non unique non razor peptide (false).
Mod. peptide IDs		
Evidence IDs		
MS/MS IDs		
Best MS/MS		The identifier of the best (in terms of quality) MS/MS scans identifying the peptides of this protein, referenced against the msms table.
Deamidation (NQ) site IDs		Identifier(s) for site(s) associated with the protein group, which show(s) evidence of the modification, referenced against the appropriate modification site file.
Hydroxyproline site IDs		Identifier(s) for site(s) associated with the protein group, which show(s) evidence of the modification, referenced against the appropriate modification site file.
Deamidation (NQ) site positions		Positions of the sites in the leading protein of this group.
Hydroxyproline site positions		Positions of the sites in the leading protein of this group.

All peptides

Name	Separator	Description
Raw file		Name of the raw file the spectral data was extracted from.
Type		The type of detection for the peptide. MULTI – A labeling multiplet was detected.<br>ISO – An isotope pattern was detected.
Charge		The charge state of the peptide.
m/z		The mass divided by the charge of the charged peptide.
Mass		The mass of the neutral peptide ((m/z-proton) * charge).
Uncalibrated m/z		m/z before recalibrations have been applied.
Resolution		The resolution of the peak detected for the peptide measured in Full Width at Half Maximum (FWHM).
Number of data points		The number of data points (peak centroids) collected for this peptide feature.
Number of scans		The number of MS scans that the 3d peaks of this peptide feature are overlapping with.
Number of isotopic peaks		The number of isotopic peaks contained in this peptide feature.
PIF		Short for Parent Ion Fraction; indicates the fraction the target peak makes up of the total intensity in the inclusion window.
Mass fractional part		The values after the radix point (ie value - floor(value)).
Mass deficit		Empirically derived deviation measure to the next nearest integer scaled to center around 0. Can be used to visually detect contaminants in a plot setting Mass against this value.<br><br>m*a+b – round(m*a+b)<br>m: the peptide mass<br>a: 0.999555<br>b: -0.10
Mass precision [ppm]		The precision of the mass detection of the peptide in parts-per-million.
Max intensity m/z 0		Summed up eXtracted Ion Current (XIC) of all isotopic clusters associated with the identified AA sequence. In case of a labeled experiment this is the total intensity of all the isotopic patterns in the label cluster.
Retention time		The retention time of the peak detected for the peptide measured in minutes.
Retention length		The total retention time width of the peak (last timepoint – first timepoint) in seconds.
Retention length (FWHM)		The full width at half maximum value retention time width of the peak in seconds.
Min scan number		The first scan number at which the peak was encountered.
Max scan number		The last scan number at which the peak was encountered.
Identified		When marked with '+' this particular MS/MS scan was identified as a peptide; when marked with '-' no identification was made.
MS/MS IDs		Unique identifier linking this identification to the MS/MS scans.
Sequence		The identified AA sequence of the peptide.
Length		The length of the sequence stored in the column "Sequence".
Modifications		Post-translational modifications contained within the sequence. When no modifications exist, this is set to 'unmodified'.<br><br>Note: This column only set when this MS/MS spectrum has been identified.
Modified sequence		Sequence representation of the peptide including location(s) of modified AAs.<br><br>Note: This column only set when this MS/MS spectrum has been identified.
Proteins		Identifiers of proteins this peptide is associated with.<br><br>Note: This column only set when this MS/MS spectrum has been identified.
Score		The score of the identification (higher is better).<br><br>Note: This column only set when this MS/MS spectrum has been identified.
Intensity		Summed up eXtracted Ion Current (XIC) of all isotopic clusters associated with the identified AA sequence. In case of a labeled experiment this is the total intensity of all the isotopic patterns in the label cluster.
Intensities		Elution profile.
Isotope pattern		Isotope pattern.

MS/MS Count		The number of MS/MS spectra recorded for the peptide.
MSMS Scan Numbers		The scan numbers where the MS/MS spectra were recorded.
MSMS Isotope Indices		Indices of the isotopic peaks that the MS/MS spectra reside on. A value of 0 corresponds to the monoisotopic peak.

MS scans

The msScans table contains information about the full scans, which can be used to verify data quality and generated useful statistics about the interaction between the samples and LC.

Name	Separator	Description
Raw file		The name of the RAW-file the mass spectral data originates from.
Scan number		The scan number (defined in the raw-file) at which the full scan was made.
Scan index		The consecutive index of this full scan.
Retention time		The retention time at which the full scan was made.
Cycle time		The total time (full scan including the tandem MS scans) this full scan has taken up.
Ion injection time		The total injection time that was required to capture the specified amount of ions. This value is limited by a maximum, which can be used to determine whether the time has maxed out (indicative of a bad acquisition).
Base peak intensity		The intensity of the most intense ion in the spectrum.
Total ion current		The total intensity acquired in the full scan.
MS/MS count		The number of tandem MS scans that were made based on this full scan (e.g. a top 10 method selects the top 10 most intense ions in the scan and fragments those).
Mass calibration		The applied mass correction in Th to the full scan.
Experiment		
Peak length		The average time between the start and the end of the peaks detected in the full scan.
Isotope pattern length		The average time between the start and the end of the isotope patterns detected in the full scan.
Multiplet length		The average time between the start and the end of the isotope patterns of the labeling multiplets detected in the full scan.
Peaks / s		The average number of peaks detected per second of chromatography.
Single peaks / s		The average number of single peaks detected per second of chromatography.
Isotope patterns / s		The average number of isotope patterns detected per second of chromatography.
Single isotope patterns / s		The average number of single isotope patterns detected per of second chromatography.
Multiplets / s		The average number of labeling multiplets detected per of second chromatography.
Identified multiplets / s		The percentage of labeling multiplets actually identified.
Multiplet identification rate [%]		The percentage of the detected labeling multiplets that were identified.
MS/MS / s		The average number of MS/MS events per second of chromatography.
Identified MS/MS / s		The average number of identified MS/MS events per second of chromatography.
MS/MS identification rate [%]		The percentage of tandem MS scans that were identified.
Intens Comp Factor		Taken from the Thermo RAW file.
CTCD Comp		Taken from the Thermo RAW file.
RawOvFtT		For Thermo Fisher only. TIC estimation done with the orbitrap cell.
AGC Fill		Taken from the Thermo RAW file.

MZ range

Name	Separator	Description
Raw file		The name of the RAW-file the mass spectral data was derived from.
m/z		The mass-over-charge value.
Peaks / Da		The average number of peaks detected per Dalton.
Single peaks / Da		The average number of single peaks detected per Dalton.
Isotope patterns / Da		The average number of isotope patterns detected per Dalton.
Single isotope patterns / Da		The average number of single isotope patterns detected per Dalton.
SILAC pairs / Da		The average number of SILAC pairs detected per Dalton.
Identified SILAC pairs / Da		The percentage of SILAC pairs actually identified.
SILAC identification rate [%]		The percentage of the detected SILAC pairs that were identified.
MS/MS / Da		The average number of MS/MS events per Dalton.
Identified MS/MS / Da		The average number of identified MS/MS events per Dalton.
Identification rate [%]		The percentage of tandem MS scans that were identified.

MS/MS scans

Name	Separator	Description
Raw file		Name of the RAW file the spectral MS/MS data was extracted from.
Scan number		RAW file derived scan number for the MS/MS spectrum.
Retention time		Time point along the elution profile at which the MS/MS data was recorded.
Ion injection time		The ion inject time for the MS/MS scan. This can be used to determine if this time equals to the maximum ion inject time, general indicative of a lower quality spectrum.
Total ion current		The total ion current of the MS/MS scan. For Thermo data this value is calculated by summing all the intensity values found in the mass spectral data, which is different from the Xcalibur reported TIC (Xcalibur TIC is about 25% of the value reported here).
Collision energy		The collision energy used for the fragmentation that resulted in this MS/MS scan.
Summations		For time of flight instruments only.
Base peak intensity		The intensity of the most intense ion in the spectrum.
Elapsed time		The time the MS/MS scan took to complete.
Identified		When marked with '+' this particular MS/MS scan was identified as a peptide; when marked with '-' no identification was made.
MS/MS IDs		Unique identifier linking this identification to the MS/MS scans.
Sequence		The identified AA sequence of the peptide.
Length		The length of the sequence stored in the column “Sequence”.
Filtered peaks		Number of peaks after the 'top X per 100 Da' filtering.
m/z		Recalibrated m/z of the precursor ion.
Mass		Charge corrected mass of the precursor ion.
Charge		Charge state of the precursor ion.
Type		The type of precursor ion as identified by MaxQuant. ISO – isotopic cluster.<br>PEAK – single peak.<br>MULTI – labeling cluster.
Fragmentation		The type of fragmentation used to create the MS/MS spectrum.<br>CID – Collision Induced Dissociation.<br>HCD – High energy Collision induced Dissociation.<br>ETD – Electron Transfer Dissociation.
Mass analyzer		The mass analyzer used to record the MS/MS spectrum. ITMS – Ion trap.<br>FTMS – Fourier transform ICR or orbitrap cell.<br>TOF – Time of flight.
Parent intensity fraction		The percentage the parent ion intensity makes up of the total intensity in the selection window.
Fraction of total spectrum		The percentage the parent ion intensity makes up of the total intensity of the whole MS spectrum.
Base peak fraction		The percentage the parent ion intensity in comparison to the highest peak in he MS spectrum.
Precursor full scan number		The full scan number where the precursor ion was selected for fragmentation.
Precursor intensity		The intensity of the precursor ion at the scannumber it was selected.
Precursor apex fraction		The fraction the intensity of the precursor ion makes up of the peak (apex) intensity.
Precursor apex offset		How many full scans the precursor ion is offset from the peak (apex) position.
Precursor apex offset time		How much time the precursor ion is offset from the peak (apex) position.
Scan event number		This number indicates which MS/MS scan this one is in the consecutive order of the MS/MS scans that are acquired after an MS scan.
Modifications		Post-translational modifications contained within the sequence. When no modifications exist, this is set to ‘unmodified’.<br><br>Note: This column only set when this MS/MS spectrum has been identified.
Modified sequence		Sequence representation of the peptide including location(s) of modified AAs.<br><br>Note: This column only set when this MS/MS spectrum has been identified.

Proteins		Identifiers of proteins this peptide is associated with.<br><br>Note: This column only set when this MS/MS spectrum has been identified.
Score		The score of the identification (higher is better).<br><br>Note: This column only set when this MS/MS spectrum has been identified.
Experiment		
Intens Comp Factor		Taken from the Thermo RAW file.
CTCD Comp		Taken from the Thermo RAW file.
RawOvFtT		For Thermo Fisher only. TIC estimation done with the orbitrap cell.
AGC Fill		Taken from the Thermo RAW file.
Scan index		Consecutive index of the MS/MS spectrum.
MS scan index		Consecutive index of the MS spectrum prior to this MS/MS spectrum.
MS scan number		Scan number of the MS spectrum prior to this MS/MS spectrum.

MS/MS

Name	Separator	Description
Raw file		The name of the RAW file the mass spectral data was read from.
Scan number		The RAW-file derived scan number of the MS/MS spectrum.
Scan index		The consecutive index of the MS/MS spectrum.
Sequence		The identified AA sequence of the peptide.
Length		The length of the sequence stored in the column "Sequence".
Missed cleavages		Number of missed enzymatic cleavages.
Modifications		Post-translational modifications contained within the identified peptide sequence.
Modified sequence		Sequence representation including the post-translational modifications (abbreviation of the modification in brackets before the modified AA). The sequence is always surrounded by underscore characters ('_').
Deamidation (NQ) Probabilities		Sequence representation of the peptide including PTM positioning probabilities ([0..1], where 1 is best match) for 'Deamidation (NQ)'.
Hydroxyproline Probabilities		Sequence representation of the peptide including PTM positioning probabilities ([0..1], where 1 is best match) for 'Hydroxyproline'.
Deamidation (NQ) Score Diffs		
Hydroxyproline Score Diffs		
Acetyl (Protein N-term)		
Deamidation (NQ)		
Gln->pyro-Glu		
Glu->pyro-Glu		
Hydroxyproline		
Oxidation (M)		
Proteins		The identifiers of the proteins the identified peptide is associated with.
Charge		The charge state of the precursor ion.
Fragmentation		The type of fragmentation used to create the MS/MS spectrum.<br>CID – Collision Induced Dissociation.<br>HCD – High energy Collision induced Dissociation.<br>ETD – Electron Transfer Dissociation.
Mass analyzer		The mass analyzer used to record the MS/MS spectrum. ITMS – Ion trap.<br>FTMS – Fourier transform ICR or orbitrap cell.<br>TOF – Time of flight.
Type		The type of precursor ion as identified by MaxQuant. ISO – isotopic cluster.<br>PEAK – single peak.<br>MULTI – labeling cluster.
Scan event number		
Isotope index		
m/z		The mass-over-charge of the precursor ion.
Mass		The charge corrected mass of the precursor ion.
Mass error [ppm]		Mass error of the recalibrated mass-over-charge value of the precursor ion in comparison to the predicted monoisotopic mass of the identified peptide sequence expressed in parts per million.
Mass error [Da]		Mass error of the recalibrated mass-over-charge value of the precursor ion in comparison to the predicted monoisotopic mass of the identified peptide sequence expressed in atomic mass units.
Simple mass error [ppm]		
Retention time		The uncalibrated retention time in minutes where the MS/MS spectrum has been acquired.
PEP		Posterior Error Probability of the identification. This value essentially operates as a p-value, where smaller is more significant.
Score		Andromeda score for the best associated MS/MS spectrum.
Delta score		Score difference to the second best identified peptide with a different amino acid sequence.
Score diff		Score difference to the second best positioning of modifications identified peptide with the same amino acid sequence.
Localization prob		
Combinatorics		Number of possible distributions of the modifications over the peptide sequence.

PIF		Short for Parent Ion Fraction; indicates the fraction the target peak makes up of the total intensity in the inclusion window.
Fraction of total spectrum		The percentage the parent ion intensity makes up of the total intensity of the whole spectrum.
Base peak fraction		The percentage the parent ion intensity in comparison to the highest peak in he MS spectrum.
Precursor Full ScanNumber		The full scannumber where the precursor ion was selected for fragmentation.
Precursor Intensity		The intensity of the precursor ion at the scannumber it was selected.
Precursor Apex Fraction		The fraction the intensity of the precursor ion makes up of the peak (apex) intensity.
Precursor Apex Offset		How many full scans the precursor ion is offset from the peak (apex) position.
Precursor Apex Offset Time		How much time the precursor ion is offset from the peak (apex) position.
Matches		The species of the peaks in the fragmentation spectrum after TopN filtering.
Intensities		The intensities of the peaks in the fragmentation spectrum after TopN filtering.
Mass Deviations [Da]		The mass deviation of each peak in the fragmentation spectrum in absolute mass units.
Mass Deviations [ppm]		The mass deviation of each peak in the fragmentation spectrum in parts per million.
Masses		The masses-over-charge of the peaks in the fragmentation spectrum.
Number of Matches		The number of peaks matching to the predicted fragmentation spectrum.
Intensity coverage		The fraction of intensity in the MS/MS spectrum that is annotated.
Peak coverage		The fraction of peaks in the MS/MS spectrum that are annotated.
Neutral loss level		How many neutral losses were applied to each fragment in the Andromeda scoring.
ETD identification type		For ETD spectra several different combinations of ion series are scored. Here the highest scoring combination is indicated
Reverse		When marked with '+', this particular peptide was found to be part of a protein derived from the reversed part of the decoy database. These should be removed for further data analysis.
All scores		
All sequences		
All modified sequences		
id		A unique (consecutive) identifier for each row in the msms table, which is used to cross-link the information in this file with the information stored in the other files.
Protein group IDs		The identifier of the protein-group this redundant peptide sequence is associated with, which can be used to look up the extended protein information in the file 'proteinGroups.txt'.<br>As a single peptide can be linked to multiple proteins (e.g. in the case of razor-proteins), multiple id's can be stored here separated by a semicolon.<br>As a protein can be identified by multiple peptides, the same id can be found in different rows.
Peptide ID		The identifier of the non-redundant peptide sequence.
Mod. peptide ID		Identifier of the associated modification summary stored in the file 'modificationSpecificPeptides.txt'.
Evidence ID		Identifier of the associated evidence stored in the file 'evidence.txt'.
Deamidation (NQ) site IDs		Identifier of the oxidation summary stored in the file 'Deamidation (NQ)Sites.txt'.
Hydroxyproline site IDs		Identifier of the oxidation summary stored in the file 'HydroxyprolineSites.txt'.
