# Supplementary material for: Assessing the degradation of ancient milk proteins through site-specific deamidation patterns
Source: Sci Rep. 2021 Apr 8;11:7795. doi: 10.1038/s41598-021-87125-x (PMC8032661; doi:10.1038/s41598-021-87125-x)
Supplement: Supplementary file 2 — Supplementary Information 2. [file 41598_2021_87125_MOESM2_ESM.zip › zip/Hendy2018b.zip!/Hendy2018b/tables.pdf]

Summary

The summary file contains summary information for all the raw files processed with a single MaxQuant run. The summary information consists of some MaxQuant parameters, information of the raw file contents, and statistics on the peak detection. Based on this file a quick overview can be gathered on the quality of the data in the raw file.

The last row in this file contains the summary information for each column on each of the processed files.

Name	Separator	Description
Raw file		The raw file processed.
Experiment		Experiment name assigned to this LC-MS run in the experimental design.
Enzyme		The protease used to digest the protein sample.
Enzyme mode		The protease used to digest the protein sample.
Enzyme first search		The protease used for the first search.
Enzyme mode first search		The protease used for the first search.
Use enzyme first search		Marked with '+' when a different protease setup was used for the first search.
Variable modifications		The variable modification(s) used during the identification of peptides.
Fixed modifications		The fixed modification(s) used during the identification of peptides.
Multi modifications		The multi modification(s) used during the identification of peptides.
Variable modifications first search		The variable modification(s) used during the first search.
Use variable modifications first search		Marked with '+' when different variable modifications were used for the first search.
Requantify		The number of labels used.
Multiplicity		The number of labels used.
Max. missed cleavages		The maximum allowed number of missed cleavages.
Labels0		The labels used in the labeling experiment. Allowed values for X: 0=light; 1=medium; 2=heavy label partner.
LC-MS run type		The type of LC-MS run. Usually it will be 'Standard' which refers to a conventional shotgun proteomics run with data-dependent MS/MS.
Time-dependent recalibration		When marked with '+', time-dependent recalibration was applied to improve the data quality.
MS		The number of MS spectra recorded in this raw file.
MS/MS		The number of MS/MS spectra recorded in this raw file.
MS3		The number of MS3 spectra recorded in this raw file.
MS/MS Submitted		The number of tandem MS spectra submitted for analysis.
MS/MS Submitted (SIL)		The number of tandem MS spectra submitted for analysis, where the precursor ion was detected as part of a labeling cluster.
MS/MS Submitted (ISO)		The number of tandem MS spectra submitted for analysis, where the precursor ion was detected as an isotopic pattern.
MS/MS Submitted (PEAK)		The number of tandem MS spectra submitted for analysis, where the precursor ion was detected as a single peak.
MS/MS Identified		The total number of identified tandem MS spectra.
MS/MS Identified (SIL)		The total number of identified tandem MS spectra, where the precursor ion was detected as part of a labeling cluster.
MS/MS Identified (ISO)		The total number of identified tandem MS spectra, where the precursor ion was detected as an isotopic pattern.
MS/MS Identified (PEAK)		The total number of identified tandem MS spectra, where the precursor ion was detected as a single peak.
MS/MS Identified [%]		The percentage of identified tandem MS spectra.
MS/MS Identified (SIL) [%]		The percentage of identified tandem MS spectra, where the precursor ion was detected as part of a labeling cluster.
MS/MS Identified (ISO) [%]		The percentage of identified tandem MS spectra, where the precursor ion was detected as an isotopic pattern.
MS/MS Identified (PEAK) [%]		The percentage of identified tandem MS spectra, where the precursor ion was detected as a single peak.
Peptide Sequences Identified		The total number of unique peptide amino acid sequences identified from the recorded tandem mass spectra.
Peaks		The total number of peaks detected in the full scans.
Peaks Sequenced		The total number of peaks sequenced by tandem MS.

Peaks Sequenced [%]		The percentage of peaks sequenced by tandem MS.
Peaks Repeatedly Sequenced		The total number of peaks repeatedly sequenced (i.e. 1 or more times) by tandem MS.
Peaks Repeatedly Sequenced [%]		The percentage of peaks repeatedly sequenced (i.e. 1 or more times) by tandem MS.
Isotope Patterns		The total number of detected isotope patterns.
Isotope Patterns Sequenced		The total number of isotope patterns sequenced by tandem MS.
Isotope Patterns Sequenced (z>1)		The total number of isotope patterns sequenced by tandem MS with a charge state of 2 or more.
Isotope Patterns Sequenced [%]		The percentage of isotope patterns sequenced by tandem MS.
Isotope Patterns Sequenced (z>1) [%]		The percentage of isotope patterns sequenced by tandem MS with a charge state of 2 or more.
Isotope Patterns Repeatedly Sequenced		The total number of isotope patterns repeatedly sequenced (i.e. 1 or more times) by tandem MS.
Isotope Patterns Repeatedly Sequenced [%]		The percentage of isotope patterns repeatedly sequenced (i.e. 1 or more times) by tandem MS.
Recalibrated		When marked with '+', the masses taken from the raw file were recalibrated.
Av. Absolute Mass Deviation [ppm]		The average absolute mass deviation found comparing to the identification mass in parts per million.
Mass Standard Deviation [ppm]		The standard deviation of the mass deviation found comparing to the identification mass in parts per million.
Av. Absolute Mass Deviation [mDa]		The average absolute mass deviation found comparing to the identification mass in milli-Dalton.
Mass Standard Deviation [mDa]		The standard deviation of the mass deviation found comparing to the identification mass in milli-Dalton.

Evidence

The evidence file combines all the information about the identified peptides and normally is the only file required for processing the results. Additional information about the peptides, modifications, proteins, etc. can be found in the other files by unique identifier linkage.

**Name**	**Separator**	**Description**
Sequence		The identified AA sequence of the peptide.
Length		The length of the sequence stored in the column 'Sequence'.
Modifications		Post-translational modifications contained within the identified peptide sequence.
Modified sequence		Sequence representation including the post-translational modifications (abbreviation of the modification in brackets before the modified AA). The sequence is always surrounded by underscore characters ('_').
Deamidation (NQ) Probabilities		Sequence representation of the peptide including PTM positioning probabilities ([0..1], where 1 is best match) for 'Deamidation (NQ)'.
Hydroxyproline Probabilities		Sequence representation of the peptide including PTM positioning probabilities ([0..1], where 1 is best match) for 'Hydroxyproline'.
Oxidation (M) Probabilities		Sequence representation of the peptide including PTM positioning probabilities ([0..1], where 1 is best match) for 'Oxidation (M)'.
Propionamide K Probabilities		Sequence representation of the peptide including PTM positioning probabilities ([0..1], where 1 is best match) for 'Propionamide K'.
Deamidation (NQ) Score Diffs		Sequence representation for each of the possible PTM positions in each possible configuration, the difference is calculated between the identification score with the PTM added to that position and the best scoring identification where no PTM is added to that position. When this value is negative, it is unlikely that the particular modification is located at this position.
Hydroxyproline Score Diffs		Sequence representation for each of the possible PTM positions in each possible configuration, the difference is calculated between the identification score with the PTM added to that position and the best scoring identification where no PTM is added to that position. When this value is negative, it is unlikely that the particular modification is located at this position.
Oxidation (M) Score Diffs		Sequence representation for each of the possible PTM positions in each possible configuration, the difference is calculated between the identification score with the PTM added to that position and the best scoring identification where no PTM is added to that position. When this value is negative, it is unlikely that the particular modification is located at this position.
Propionamide K Score Diffs		Sequence representation for each of the possible PTM positions in each possible configuration, the difference is calculated between the identification score with the PTM added to that position and the best scoring identification where no PTM is added to that position. When this value is negative, it is unlikely that the particular modification is located at this position.
Acetyl (Protein N-term)		The number of occurrences of the modification 'Acetyl (Protein N-term)'.
Deamidation (NQ)		The number of occurrences of the modification 'Deamidation (NQ)'.
Gln->pyro-Glu		The number of occurrences of the modification 'Gln->pyro-Glu'.
Glu->pyro-Glu		The number of occurrences of the modification 'Glu->pyro-Glu'.
Hydroxyproline		The number of occurrences of the modification 'Hydroxyproline'.
Oxidation (M)		The number of occurrences of the modification 'Oxidation (M)'.
Propionamide K		The number of occurrences of the modification 'Propionamide K'.
Propionamide N-term		The number of occurrences of the modification 'Propionamide N-term'.
Missed cleavages		Number of missed enzymatic cleavages.
Proteins		The identifiers of the proteins this particular peptide is associated with.
Leading proteins		The identifiers of the proteins in the proteinGroups file, with this protein as best match, this particular peptide is associated with. When multiple matches are found here, the best scoring protein can be found in the 'Leading Razor Protein' column.

Leading razor protein		The identifier of the best scoring protein, from the proteinGroups file this, this peptide is associated to.
Type		The type of the feature. 'MSMS' – for an MS/MS spectrum without an MS1 isotope pattern assigned. 'ISO-MSMS' – MS1 isotope cluster identified by MS/MS. 'MULTI-MSMS' – MS1 labeling cluster identified by MS/MS. 'MULTI-SECPEP' – MS1 labeling cluster identified by MS/MS as second peptide. 'MULTI-MATCH' – MS1 labeling cluster identified by matching between runs. In case of label-free data there is no difference between 'MULTI' and 'ISO'.
Raw file		The name of the RAW-file the mass spectral data was derived from.
Experiment		
MS/MS m/z		The m/z used for fragmentation (not necessarily the mono-isotopic m/z).
Charge		The charge-state of the precursor ion.
m/z		The recalibrated mass-over-charge value of the precursor ion.
Mass		The predicted monoisotopic mass of the identified peptide sequence.
Resolution		The resolution of precursor ion measured in Full Width at Half Maximum (FWHM).
Uncalibrated - Calibrated m/z [ppm]		The difference between the uncalibrated and recalibrated mass-over-charge value of the precursor ion measured in parts-per-million. This gives an indication of the mass drift in the original data, which was automatically corrected by MaxQuant.
Uncalibrated - Calibrated m/z [Da]		The difference between the uncalibrated and recalibrated mass-over-charge value of the precursor ion measured in parts-per-million. This gives an indication of the mass drift in the original data, which was automatically corrected by MaxQuant.
Mass error [ppm]		Mass error of the recalibrated mass-over-charge value of the precursor ion in comparison to the predicted monoisotopic mass of the identified peptide sequence in parts per million.
Mass error [Da]		Mass error of the recalibrated mass-over-charge value of the precursor ion in comparison to the predicted monoisotopic mass of the identified peptide sequence in milli-Dalton.
Uncalibrated mass error [ppm]		Mass error of the uncalibrated mass-over-charge value of the precursor ion in comparison to the predicted monoisotopic mass of the identified peptide sequence.<br><br>Note: This column can contain missing values (denoted as NaN).
Uncalibrated mass error [Da]		Mass error of the uncalibrated mass-over-charge value of the precursor ion in comparison to the predicted monoisotopic mass of the identified peptide sequence.<br><br>Note: This column can contain missing values (denoted as NaN).
Max intensity m/z 0		Summed up eXtracted Ion Current (XIC) of all isotopic clusters associated with the identified AA sequence. In case of a labeled experiment this is the total intensity of all the isotopic patterns in the label cluster.
Retention time		The uncalibrated retention time in minutes in the elution profile of the precursor ion.
Retention length		The total retention time length of the peak (last timepoint – first timepoint).
Calibrated retention time		The recalibrated retention time in minutes in the elution profile of the precursor ion.
Calibrated retention time start		The recalibrated retention start in minutes in the elution profile of the precursor ion.
Calibrated retention time finish		The recalibrated retention finish in minutes in the elution profile of the precursor ion.
Retention time calibration		The difference in minutes between the uncalibrated and recalibrated retention time. This gives an indication of the retention time drift in the original data, which was automatically corrected by MaxQuant.<br><br>Note: This column can contain missing values (NaN).
Match time difference		When the option ‘match between runs’ is used in MaxQuant, this value indicates the time difference between the feature from the raw file it was taken from and the feature from the raw file it was matched to.
Match m/z difference		When the option ‘match between runs’ is used in MaxQuant, this value indicates the m/z difference between the feature from the raw file it was taken from and the feature from the raw file it was matched to.
Match q-value		This is the q-value for features that have been identified by 'matching between runs'.
Match score		The andromeda score of the MS/MS identification that is the source of this identification by 'matching between runs'.

Number of data points		The number of data points (peak centroids) collected for this peptide feature.
Number of scans		The number of MS scans that the 3d peaks of this peptide feature are overlapping with.
Number of isotopic peaks		The number of isotopic peaks contained in this peptide feature.
PIF		Short for Parent Ion Fraction; indicates the fraction the target peak makes up of the total intensity in the inclusion window.
Fraction of total spectrum		The percentage the ion intensity makes up of the total intensity of the whole spectrum.
Base peak fraction		The percentage the parent ion intensity in comparison to the highest peak in the MS spectrum.
PEP		Posterior Error Probability of the identification. This value essentially operates as a p-value, where smaller is more significant.
MS/MS count		The number of sequencing events for this sequence, which matches the number of identifiers stored in the column ‘MS/MS IDs’.
MS/MS scan number		The RAW-file derived scan number of the MS/MS with the highest peptide identification score (the highest score is stored in the column 'Score').
Score		Andromeda score for the best associated MS/MS spectrum.
Delta score		Score difference to the second best identified peptide.
Combinatorics		Number of possible distributions of the modifications over the peptide sequence.
Intensity		Summed up eXtracted Ion Current (XIC) of all isotopic clusters associated with the identified AA sequence. In case of a labeled experiment this is the total intensity of all the isotopic patterns in the label cluster.
Reverse		When marked with '+', this particular peptide was found to be part of a protein derived from the reversed part of the decoy database. These should be removed for further data analysis.
Potential contaminant		When marked with '+', this particular peptide was found to be part of a commonly occurring contaminant. These should be removed for further data analysis.
id		A unique (consecutive) identifier for each row in the evidence table, which is used to cross-link the information in this file with the information stored in the other files.
Protein group IDs		The identifier of the protein-group this redundant peptide sequence is associated with, which can be used to look up the extended protein information in the file 'proteinGroups.txt'. As a single peptide can be linked to multiple proteins (e.g. in the case of razor-proteins), multiple id’s can be stored here separated by a semicolon. As a protein can be identified by multiple peptides, the same id can be found in different rows.
Peptide ID		The identifier of the non-redundant peptide sequence.
Mod. peptide ID		Identifier of the associated modification summary stored in the file 'modificationSpecificPeptides.txt'.
MS/MS IDs		Identifier(s) of the associated MS/MS summary(s) stored in the file 'msms.txt'.
Best MS/MS		Identifier(s) of the best MS/MS associated spectrum stored in the file 'msms.txt'.
AIF MS/MS IDs		Identifier(s) of the associated All Ion Fragmentation MS/MS summary(s) stored in the file 'aifMsms.txt'.
Deamidation (NQ) site IDs		Identifier(s) of the modification summary stored in the file 'Deamidation (NQ)Sites.txt'.
Hydroxyproline site IDs		Identifier(s) of the modification summary stored in the file 'HydroxyprolineSites.txt'.
Oxidation (M) site IDs		Identifier(s) of the modification summary stored in the file 'Oxidation (M)Sites.txt'.
Propionamide K site IDs		Identifier(s) of the modification summary stored in the file 'Propionamide KSites.txt'.

Peptides

The peptides table contains information on the identified peptides in the processed raw-files.

Name	Separator	Description
Sequence		The amino acid sequence of the identified peptide.
N-term cleavage window		Sequence window from -15 to 15 around the N-terminal cleavage site of this peptide.
C-term cleavage window		Sequence window from -15 to 15 around the C-terminal cleavage site of this peptide.
Amino acid before		The amino acid in the protein sequence before the peptide.
First amino acid		The amino acid in the first position of the peptide sequence.
Second amino acid		The amino acid in the first position of the peptide sequence.
Second last amino acid		The amino acid in the last position of the peptide sequence.
Last amino acid		The amino acid in the last position of the peptide sequence.
Amino acid after		The amino acid in the protein sequence after the peptide.
A Count		The number of instances of the 'A' amino acid contained within the sequence.
R Count		The number of instances of the 'R' amino acid contained within the sequence.
N Count		The number of instances of the 'N' amino acid contained within the sequence.
D Count		The number of instances of the 'D' amino acid contained within the sequence.
C Count		The number of instances of the 'C' amino acid contained within the sequence.
Q Count		The number of instances of the 'Q' amino acid contained within the sequence.
E Count		The number of instances of the 'E' amino acid contained within the sequence.
G Count		The number of instances of the 'G' amino acid contained within the sequence.
H Count		The number of instances of the 'H' amino acid contained within the sequence.
I Count		The number of instances of the 'I' amino acid contained within the sequence.
L Count		The number of instances of the 'L' amino acid contained within the sequence.
K Count		The number of instances of the 'K' amino acid contained within the sequence.
M Count		The number of instances of the 'M' amino acid contained within the sequence.
F Count		The number of instances of the 'F' amino acid contained within the sequence.
P Count		The number of instances of the 'P' amino acid contained within the sequence.
S Count		The number of instances of the 'S' amino acid contained within the sequence.
T Count		The number of instances of the 'T' amino acid contained within the sequence.
W Count		The number of instances of the 'W' amino acid contained within the sequence.
Y Count		The number of instances of the 'Y' amino acid contained within the sequence.
V Count		The number of instances of the 'V' amino acid contained within the sequence.
U Count		The number of instances of the 'U' amino acid contained within the sequence.
O Count		The number of instances of the 'O' amino acid contained within the sequence.
Length		The length of the sequence stored in the column "Sequence".
Missed cleavages		Number of missed enzymatic cleavages.
Mass		Monoisotopic mass of the peptide.
Proteins		Identifiers of proteins this peptide is associated with.
Leading razor protein		Identifiers of the best scoring protein this peptide is associated with.
Start position		Position of the first amino acid of this peptide in the protein sequence. (one-based)
End position		Position of the last amino acid of this peptide in the protein sequence. (one-based)

Unique (Groups)		When marked with '+', this particular peptide is unique to a single protein group in the proteinGroups file.
Unique (Proteins)		When marked with '+', this particular peptide is unique to a single protein sequence in the fasta file(s).
Charges		All charge states that have been observed.
PEP		Posterior Error Probability of the identification. This value essentially operates as a p-value, where smaller is more significant.
Score		Highest Andromeda score for the associated MS/MS spectra.
Experiment 20160805_QE5_nLC7_MEM_COLLAB_JH_A1_01		Number of evidence entries for this 'Experiment'.
Experiment 20160805_QE5_nLC7_MEM_COLLAB_JH_A2_01		Number of evidence entries for this 'Experiment'.
Experiment 20160805_QE5_nLC7_MEM_COLLAB_JH_A3_01		Number of evidence entries for this 'Experiment'.
Experiment 20160805_QE5_nLC7_MEM_COLLAB_JH_B1_01		Number of evidence entries for this 'Experiment'.
Experiment 20160805_QE5_nLC7_MEM_COLLAB_JH_B2_01		Number of evidence entries for this 'Experiment'.
Experiment 20160805_QE5_nLC7_MEM_COLLAB_JH_B3_01		Number of evidence entries for this 'Experiment'.
Experiment 20160805_QE5_nLC7_MEM_COLLAB_JH_B4_01		Number of evidence entries for this 'Experiment'.
Experiment 20160805_QE5_nLC7_MEM_COLLAB_JH_B5_01		Number of evidence entries for this 'Experiment'.
Experiment 20160805_QE5_nLC7_MEM_COLLAB_JH_C1_01		Number of evidence entries for this 'Experiment'.
Experiment 20160805_QE5_nLC7_MEM_COLLAB_JH_C2_01		Number of evidence entries for this 'Experiment'.
Experiment 20160805_QE5_nLC7_MEM_COLLAB_JH_C3_01		Number of evidence entries for this 'Experiment'.
Experiment 20160805_QE5_nLC7_MEM_COLLAB_JH_C4_01		Number of evidence entries for this 'Experiment'.
Experiment 20160805_QE5_nLC7_MEM_COLLAB_JH_C5_01		Number of evidence entries for this 'Experiment'.
Experiment 20160805_QE5_nLC7_MEM_COLLAB_JH_C6_01		Number of evidence entries for this 'Experiment'.
Experiment 20160805_QE5_nLC7_MEM_COLLAB_JH_C7_01		Number of evidence entries for this 'Experiment'.
Experiment 20160805_QE5_nLC7_MEM_COLLAB_JH_C8_01		Number of evidence entries for this 'Experiment'.
Experiment FL0145_JHendy_130416_MSQ525_CW10		Number of evidence entries for this 'Experiment'.
Experiment FL0145_JHendy_130416_MSQ525_CW11		Number of evidence entries for this 'Experiment'.
Experiment FL0145_JHendy_130416_MSQ525_CW18		Number of evidence entries for this 'Experiment'.
Experiment FL0145_JHendy_130416_MSQ525_CW20		Number of evidence entries for this 'Experiment'.
Experiment FL0145_JHendy_130416_MSQ525_CW21		Number of evidence entries for this 'Experiment'.
Experiment FL0145_JHendy_130416_MSQ525_CW23		Number of evidence entries for this 'Experiment'.
Experiment FL0145_JHendy_130416_MSQ525_CW8_160425165036		Number of evidence entries for this 'Experiment'.
Experiment FL0168_JHendy_MSQ525_170516_BK		Number of evidence entries for this 'Experiment'.

Experiment FL0168_JHendy_MSQ525_170516_CW1		Number of evidence entries for this 'Experiment'.
Experiment FL0168_JHendy_MSQ525_170516_CW10		Number of evidence entries for this 'Experiment'.
Experiment FL0168_JHendy_MSQ525_170516_CW2		Number of evidence entries for this 'Experiment'.
Experiment FL0168_JHendy_MSQ525_170516_CW3		Number of evidence entries for this 'Experiment'.
Experiment FL0168_JHendy_MSQ525_170516_CW4		Number of evidence entries for this 'Experiment'.
Experiment FL0168_JHendy_MSQ525_170516_CW5		Number of evidence entries for this 'Experiment'.
Experiment FL0168_JHendy_MSQ525_170516_CW6		Number of evidence entries for this 'Experiment'.
Experiment FL0168_JHendy_MSQ525_170516_CW7		Number of evidence entries for this 'Experiment'.
Experiment FL0168_JHendy_MSQ525_170516_CW8		Number of evidence entries for this 'Experiment'.
Experiment FL0168_JHendy_MSQ525_170516_CW9		Number of evidence entries for this 'Experiment'.
Intensity		Summed up eXtracted Ion Current (XIC) of all isotopic clusters associated with the identified AA sequence. In case of a labeled experiment this is the total intensity of all the isotopic patterns in the label cluster.
Intensity 20160805_QE5_nLC7_MEM_COLLAB_JH_A1_01		Summed up eXtracted Ion Current (XIC) of all isotopic clusters associated with the identified AA sequence. In case of a labeled experiment this is the total intensity of all the isotopic patterns in the label cluster.
Intensity 20160805_QE5_nLC7_MEM_COLLAB_JH_A2_01		Summed up eXtracted Ion Current (XIC) of all isotopic clusters associated with the identified AA sequence. In case of a labeled experiment this is the total intensity of all the isotopic patterns in the label cluster.
Intensity 20160805_QE5_nLC7_MEM_COLLAB_JH_A3_01		Summed up eXtracted Ion Current (XIC) of all isotopic clusters associated with the identified AA sequence. In case of a labeled experiment this is the total intensity of all the isotopic patterns in the label cluster.
Intensity 20160805_QE5_nLC7_MEM_COLLAB_JH_B1_01		Summed up eXtracted Ion Current (XIC) of all isotopic clusters associated with the identified AA sequence. In case of a labeled experiment this is the total intensity of all the isotopic patterns in the label cluster.
Intensity 20160805_QE5_nLC7_MEM_COLLAB_JH_B2_01		Summed up eXtracted Ion Current (XIC) of all isotopic clusters associated with the identified AA sequence. In case of a labeled experiment this is the total intensity of all the isotopic patterns in the label cluster.
Intensity 20160805_QE5_nLC7_MEM_COLLAB_JH_B3_01		Summed up eXtracted Ion Current (XIC) of all isotopic clusters associated with the identified AA sequence. In case of a labeled experiment this is the total intensity of all the isotopic patterns in the label cluster.
Intensity 20160805_QE5_nLC7_MEM_COLLAB_JH_B4_01		Summed up eXtracted Ion Current (XIC) of all isotopic clusters associated with the identified AA sequence. In case of a labeled experiment this is the total intensity of all the isotopic patterns in the label cluster.
Intensity 20160805_QE5_nLC7_MEM_COLLAB_JH_B5_01		Summed up eXtracted Ion Current (XIC) of all isotopic clusters associated with the identified AA sequence. In case of a labeled experiment this is the total intensity of all the isotopic patterns in the label cluster.
Intensity 20160805_QE5_nLC7_MEM_COLLAB_JH_C1_01		Summed up eXtracted Ion Current (XIC) of all isotopic clusters associated with the identified AA sequence. In case of a labeled experiment this is the total intensity of all the isotopic patterns in the label cluster.
Intensity 20160805_QE5_nLC7_MEM_COLLAB_JH_C2_01		Summed up eXtracted Ion Current (XIC) of all isotopic clusters associated with the identified AA sequence. In case of a labeled experiment this is the total intensity of all the isotopic patterns in the label cluster.
Intensity 20160805_QE5_nLC7_MEM_COLLAB_JH_C3_01		Summed up eXtracted Ion Current (XIC) of all isotopic clusters associated with the identified AA sequence. In case of a labeled experiment this is the total intensity of all the isotopic patterns in the label cluster.
Intensity 20160805_QE5_nLC7_MEM_COLLAB_JH_C4_01		Summed up eXtracted Ion Current (XIC) of all isotopic clusters associated with the identified AA sequence. In case of a labeled experiment this is the total intensity of all the isotopic patterns in the label cluster.

Intensity 20160805_QE5_nLC7_MEM_COLLAB_JH_C5_01		Summed up eXtracted Ion Current (XIC) of all isotopic clusters associated with the identified AA sequence. In case of a labeled experiment this is the total intensity of all the isotopic patterns in the label cluster.
Intensity 20160805_QE5_nLC7_MEM_COLLAB_JH_C6_01		Summed up eXtracted Ion Current (XIC) of all isotopic clusters associated with the identified AA sequence. In case of a labeled experiment this is the total intensity of all the isotopic patterns in the label cluster.
Intensity 20160805_QE5_nLC7_MEM_COLLAB_JH_C7_01		Summed up eXtracted Ion Current (XIC) of all isotopic clusters associated with the identified AA sequence. In case of a labeled experiment this is the total intensity of all the isotopic patterns in the label cluster.
Intensity 20160805_QE5_nLC7_MEM_COLLAB_JH_C8_01		Summed up eXtracted Ion Current (XIC) of all isotopic clusters associated with the identified AA sequence. In case of a labeled experiment this is the total intensity of all the isotopic patterns in the label cluster.
Intensity FL0145_JHendy_130416_MSQ525_CW10		Summed up eXtracted Ion Current (XIC) of all isotopic clusters associated with the identified AA sequence. In case of a labeled experiment this is the total intensity of all the isotopic patterns in the label cluster.
Intensity FL0145_JHendy_130416_MSQ525_CW11		Summed up eXtracted Ion Current (XIC) of all isotopic clusters associated with the identified AA sequence. In case of a labeled experiment this is the total intensity of all the isotopic patterns in the label cluster.
Intensity FL0145_JHendy_130416_MSQ525_CW18		Summed up eXtracted Ion Current (XIC) of all isotopic clusters associated with the identified AA sequence. In case of a labeled experiment this is the total intensity of all the isotopic patterns in the label cluster.
Intensity FL0145_JHendy_130416_MSQ525_CW20		Summed up eXtracted Ion Current (XIC) of all isotopic clusters associated with the identified AA sequence. In case of a labeled experiment this is the total intensity of all the isotopic patterns in the label cluster.
Intensity FL0145_JHendy_130416_MSQ525_CW21		Summed up eXtracted Ion Current (XIC) of all isotopic clusters associated with the identified AA sequence. In case of a labeled experiment this is the total intensity of all the isotopic patterns in the label cluster.
Intensity FL0145_JHendy_130416_MSQ525_CW23		Summed up eXtracted Ion Current (XIC) of all isotopic clusters associated with the identified AA sequence. In case of a labeled experiment this is the total intensity of all the isotopic patterns in the label cluster.
Intensity FL0145_JHendy_130416_MSQ525_CW8_160425165036		Summed up eXtracted Ion Current (XIC) of all isotopic clusters associated with the identified AA sequence. In case of a labeled experiment this is the total intensity of all the isotopic patterns in the label cluster.
Intensity FL0168_JHendy_MSQ525_170516_BK		Summed up eXtracted Ion Current (XIC) of all isotopic clusters associated with the identified AA sequence. In case of a labeled experiment this is the total intensity of all the isotopic patterns in the label cluster.
Intensity FL0168_JHendy_MSQ525_170516_CW1		Summed up eXtracted Ion Current (XIC) of all isotopic clusters associated with the identified AA sequence. In case of a labeled experiment this is the total intensity of all the isotopic patterns in the label cluster.
Intensity FL0168_JHendy_MSQ525_170516_CW10		Summed up eXtracted Ion Current (XIC) of all isotopic clusters associated with the identified AA sequence. In case of a labeled experiment this is the total intensity of all the isotopic patterns in the label cluster.
Intensity FL0168_JHendy_MSQ525_170516_CW2		Summed up eXtracted Ion Current (XIC) of all isotopic clusters associated with the identified AA sequence. In case of a labeled experiment this is the total intensity of all the isotopic patterns in the label cluster.
Intensity FL0168_JHendy_MSQ525_170516_CW3		Summed up eXtracted Ion Current (XIC) of all isotopic clusters associated with the identified AA sequence. In case of a labeled experiment this is the total intensity of all the isotopic patterns in the label cluster.
Intensity FL0168_JHendy_MSQ525_170516_CW4		Summed up eXtracted Ion Current (XIC) of all isotopic clusters associated with the identified AA sequence. In case of a labeled experiment this is the total intensity of all the isotopic patterns in the label cluster.
Intensity FL0168_JHendy_MSQ525_170516_CW5		Summed up eXtracted Ion Current (XIC) of all isotopic clusters associated with the identified AA sequence. In case of a labeled experiment this is the total intensity of all the isotopic patterns in the label cluster.
Intensity FL0168_JHendy_MSQ525_170516_CW6		Summed up eXtracted Ion Current (XIC) of all isotopic clusters associated with the identified AA sequence. In case of a labeled experiment this is the total intensity of all the isotopic patterns in the label cluster.
Intensity FL0168_JHendy_MSQ525_170516_CW7		Summed up eXtracted Ion Current (XIC) of all isotopic clusters associated with the identified AA sequence. In case of a labeled experiment this is the total intensity of all the isotopic patterns in the label cluster.
Intensity FL0168_JHendy_MSQ525_170516_CW8		Summed up eXtracted Ion Current (XIC) of all isotopic clusters associated with the identified AA sequence. In case of a labeled experiment this is the total intensity of all the isotopic patterns in the label cluster.

Intensity FL0168_JHendy_MSQ525_170516_CW9		Summed up eXtracted Ion Current (XIC) of all isotopic clusters associated with the identified AA sequence. In case of a labeled experiment this is the total intensity of all the isotopic patterns in the label cluster.
Reverse		When marked with '+', this particular peptide was found to be part of a protein derived from the reversed part of the decoy database. These should be removed for further data analysis.
Potential contaminant		When marked with '+', this particular peptide was found to be part of a commonly occurring contaminant. These should be removed for further data analysis.
id		A unique (consecutive) identifier for each row in the peptides table, which is used to cross-link the information in this table with the information stored in the other tables.
Protein group IDs		The identifiers of the protein groups this peptide was linked to, referenced against the proteinGroups table.
Mod. peptide IDs		Identifier(s) for peptide sequence(s), associated with the peptide, referenced against the corresponding modified peptides table.
Evidence IDs		Identifier(s) for analyzed peptide evidence associated with the protein group referenced against the evidence table.
MS/MS IDs		The identifiers of the MS/MS scans identifying this peptide, referenced against the msms table.
Best MS/MS		The identifier of the best (in terms of quality) MS/MS scan identifying this peptide, referenced against the msms table.
Deamidation (NQ) site IDs		Identifier(s) for site(s) associated with the protein group, which show(s) evidence of the modification, referenced against the appropriate modification site file.
Hydroxyproline site IDs		Identifier(s) for site(s) associated with the protein group, which show(s) evidence of the modification, referenced against the appropriate modification site file.
Oxidation (M) site IDs		Identifier(s) for site(s) associated with the protein group, which show(s) evidence of the modification, referenced against the appropriate modification site file.
Propionamide K site IDs		Identifier(s) for site(s) associated with the protein group, which show(s) evidence of the modification, referenced against the appropriate modification site file.
MS/MS Count		

Modification-specific peptides

Name	Separator	Description
Sequence		The identified AA sequence of the peptide.
Modifications		Post-translational modifications contained within the sequence. When no modifications exist, this is set to 'unmodified'.
Mass		Charge corrected mass of the precursor ion.
Mass Fractional Part		The values after the decimal point (ie value - floor(value)).
Protein Groups		IDs of the protein groups to which this peptide belongs.
Proteins		The identifiers of the proteins this particular peptide is associated with.
Unique (Groups)		When marked with '+', this particular peptide is unique to a single protein group in the proteinGroups file.
Unique (Proteins)		When marked with '+', this particular peptide is unique to a single protein sequence in the fasta file(s).
Acetyl (Protein N-term)		Number of Acetyl (Protein N-term) on this peptide.
Deamidation (NQ)		Number of Deamidation (NQ) on this peptide.
Gln->pyro-Glu		Number of Gln->pyro-Glu on this peptide.
Glu->pyro-Glu		Number of Glu->pyro-Glu on this peptide.
Hydroxyproline		Number of Hydroxyproline on this peptide.
Oxidation (M)		Number of Oxidation (M) on this peptide.
Propionamide K		Number of Propionamide K on this peptide.
Propionamide N-term		Number of Propionamide N-term on this peptide.
Missed cleavages		Number of missed enzymatic cleavages.
Experiment 20160805_QE5_nLC7_MEM_COLLAB_JH_A1_01		Number of evidence entries for this 'Experiment'.
Experiment 20160805_QE5_nLC7_MEM_COLLAB_JH_A2_01		Number of evidence entries for this 'Experiment'.
Experiment 20160805_QE5_nLC7_MEM_COLLAB_JH_A3_01		Number of evidence entries for this 'Experiment'.
Experiment 20160805_QE5_nLC7_MEM_COLLAB_JH_B1_01		Number of evidence entries for this 'Experiment'.
Experiment 20160805_QE5_nLC7_MEM_COLLAB_JH_B2_01		Number of evidence entries for this 'Experiment'.
Experiment 20160805_QE5_nLC7_MEM_COLLAB_JH_B3_01		Number of evidence entries for this 'Experiment'.
Experiment 20160805_QE5_nLC7_MEM_COLLAB_JH_B4_01		Number of evidence entries for this 'Experiment'.
Experiment 20160805_QE5_nLC7_MEM_COLLAB_JH_B5_01		Number of evidence entries for this 'Experiment'.
Experiment 20160805_QE5_nLC7_MEM_COLLAB_JH_C1_01		Number of evidence entries for this 'Experiment'.
Experiment 20160805_QE5_nLC7_MEM_COLLAB_JH_C2_01		Number of evidence entries for this 'Experiment'.
Experiment 20160805_QE5_nLC7_MEM_COLLAB_JH_C3_01		Number of evidence entries for this 'Experiment'.
Experiment 20160805_QE5_nLC7_MEM_COLLAB_JH_C4_01		Number of evidence entries for this 'Experiment'.
Experiment 20160805_QE5_nLC7_MEM_COLLAB_JH_C5_01		Number of evidence entries for this 'Experiment'.
Experiment 20160805_QE5_nLC7_MEM_COLLAB_JH_C6_01		Number of evidence entries for this 'Experiment'.
Experiment 20160805_QE5_nLC7_MEM_COLLAB_JH_C7_01		Number of evidence entries for this 'Experiment'.
Experiment 20160805_QE5_nLC7_MEM_COLLAB_JH_C8_01		Number of evidence entries for this 'Experiment'.

Experiment FL0145_JHendy_130416_MSQ525_CW10		Number of evidence entries for this 'Experiment'.
Experiment FL0145_JHendy_130416_MSQ525_CW11		Number of evidence entries for this 'Experiment'.
Experiment FL0145_JHendy_130416_MSQ525_CW18		Number of evidence entries for this 'Experiment'.
Experiment FL0145_JHendy_130416_MSQ525_CW20		Number of evidence entries for this 'Experiment'.
Experiment FL0145_JHendy_130416_MSQ525_CW21		Number of evidence entries for this 'Experiment'.
Experiment FL0145_JHendy_130416_MSQ525_CW23		Number of evidence entries for this 'Experiment'.
Experiment FL0145_JHendy_130416_MSQ525_CW8_160425165036		Number of evidence entries for this 'Experiment'.
Experiment FL0168_JHendy_MSQ525_170516_BK		Number of evidence entries for this 'Experiment'.
Experiment FL0168_JHendy_MSQ525_170516_CW1		Number of evidence entries for this 'Experiment'.
Experiment FL0168_JHendy_MSQ525_170516_CW10		Number of evidence entries for this 'Experiment'.
Experiment FL0168_JHendy_MSQ525_170516_CW2		Number of evidence entries for this 'Experiment'.
Experiment FL0168_JHendy_MSQ525_170516_CW3		Number of evidence entries for this 'Experiment'.
Experiment FL0168_JHendy_MSQ525_170516_CW4		Number of evidence entries for this 'Experiment'.
Experiment FL0168_JHendy_MSQ525_170516_CW5		Number of evidence entries for this 'Experiment'.
Experiment FL0168_JHendy_MSQ525_170516_CW6		Number of evidence entries for this 'Experiment'.
Experiment FL0168_JHendy_MSQ525_170516_CW7		Number of evidence entries for this 'Experiment'.
Experiment FL0168_JHendy_MSQ525_170516_CW8		Number of evidence entries for this 'Experiment'.
Experiment FL0168_JHendy_MSQ525_170516_CW9		Number of evidence entries for this 'Experiment'.
Retention time		Retention time in minutes averaged over the evidence entries belonging to this modification-specific peptide.
Calibrated retention time		Calibrated retention time averaged over the evidence entries belonging to this modification-specific peptide. Obviously this only makes sense if retention time recalibration has been performed which is the case when matching between run is selected.
Charges		All charge states that have been observed.
PEP		Posterior Error Probability of the identification. This value essentially operates as a p-value, where smaller is more significant.
MS/MS scan number		The RAW-file derived scan number of the MS/MS with the highest peptide identification score (the highest score is stored in the column 'Score').
Raw file		The name of the RAW-file the mass spectral data was derived from.
Score		Andromeda score for the best identified among the associated MS/MS spectra.
Delta score		Score difference to the second best identified peptide.
Intensity		Summed up eXtracted Ion Current (XIC) of all isotopic clusters associated with the identified AA sequence. In case of a labeled experiment this is the total intensity of all the isotopic patterns in the label cluster.
Intensity 20160805_QE5_nLC7_MEM_COLLAB_JH_A1_01		Summed up eXtracted Ion Current (XIC) of all isotopic clusters associated with the identified AA sequence. In case of a labeled experiment this is the total intensity of all the isotopic patterns in the label cluster.

Intensity 20160805_QE5_nLC7_MEM_COLLAB_JH_A2_01		Summed up eXtracted Ion Current (XIC) of all isotopic clusters associated with the identified AA sequence. In case of a labeled experiment this is the total intensity of all the isotopic patterns in the label cluster.
Intensity 20160805_QE5_nLC7_MEM_COLLAB_JH_A3_01		Summed up eXtracted Ion Current (XIC) of all isotopic clusters associated with the identified AA sequence. In case of a labeled experiment this is the total intensity of all the isotopic patterns in the label cluster.
Intensity 20160805_QE5_nLC7_MEM_COLLAB_JH_B1_01		Summed up eXtracted Ion Current (XIC) of all isotopic clusters associated with the identified AA sequence. In case of a labeled experiment this is the total intensity of all the isotopic patterns in the label cluster.
Intensity 20160805_QE5_nLC7_MEM_COLLAB_JH_B2_01		Summed up eXtracted Ion Current (XIC) of all isotopic clusters associated with the identified AA sequence. In case of a labeled experiment this is the total intensity of all the isotopic patterns in the label cluster.
Intensity 20160805_QE5_nLC7_MEM_COLLAB_JH_B3_01		Summed up eXtracted Ion Current (XIC) of all isotopic clusters associated with the identified AA sequence. In case of a labeled experiment this is the total intensity of all the isotopic patterns in the label cluster.
Intensity 20160805_QE5_nLC7_MEM_COLLAB_JH_B4_01		Summed up eXtracted Ion Current (XIC) of all isotopic clusters associated with the identified AA sequence. In case of a labeled experiment this is the total intensity of all the isotopic patterns in the label cluster.
Intensity 20160805_QE5_nLC7_MEM_COLLAB_JH_B5_01		Summed up eXtracted Ion Current (XIC) of all isotopic clusters associated with the identified AA sequence. In case of a labeled experiment this is the total intensity of all the isotopic patterns in the label cluster.
Intensity 20160805_QE5_nLC7_MEM_COLLAB_JH_C1_01		Summed up eXtracted Ion Current (XIC) of all isotopic clusters associated with the identified AA sequence. In case of a labeled experiment this is the total intensity of all the isotopic patterns in the label cluster.
Intensity 20160805_QE5_nLC7_MEM_COLLAB_JH_C2_01		Summed up eXtracted Ion Current (XIC) of all isotopic clusters associated with the identified AA sequence. In case of a labeled experiment this is the total intensity of all the isotopic patterns in the label cluster.
Intensity 20160805_QE5_nLC7_MEM_COLLAB_JH_C3_01		Summed up eXtracted Ion Current (XIC) of all isotopic clusters associated with the identified AA sequence. In case of a labeled experiment this is the total intensity of all the isotopic patterns in the label cluster.
Intensity 20160805_QE5_nLC7_MEM_COLLAB_JH_C4_01		Summed up eXtracted Ion Current (XIC) of all isotopic clusters associated with the identified AA sequence. In case of a labeled experiment this is the total intensity of all the isotopic patterns in the label cluster.
Intensity 20160805_QE5_nLC7_MEM_COLLAB_JH_C5_01		Summed up eXtracted Ion Current (XIC) of all isotopic clusters associated with the identified AA sequence. In case of a labeled experiment this is the total intensity of all the isotopic patterns in the label cluster.
Intensity 20160805_QE5_nLC7_MEM_COLLAB_JH_C6_01		Summed up eXtracted Ion Current (XIC) of all isotopic clusters associated with the identified AA sequence. In case of a labeled experiment this is the total intensity of all the isotopic patterns in the label cluster.
Intensity 20160805_QE5_nLC7_MEM_COLLAB_JH_C7_01		Summed up eXtracted Ion Current (XIC) of all isotopic clusters associated with the identified AA sequence. In case of a labeled experiment this is the total intensity of all the isotopic patterns in the label cluster.
Intensity 20160805_QE5_nLC7_MEM_COLLAB_JH_C8_01		Summed up eXtracted Ion Current (XIC) of all isotopic clusters associated with the identified AA sequence. In case of a labeled experiment this is the total intensity of all the isotopic patterns in the label cluster.
Intensity FL0145_JHendy_130416_MSQ525_CW10		Summed up eXtracted Ion Current (XIC) of all isotopic clusters associated with the identified AA sequence. In case of a labeled experiment this is the total intensity of all the isotopic patterns in the label cluster.
Intensity FL0145_JHendy_130416_MSQ525_CW11		Summed up eXtracted Ion Current (XIC) of all isotopic clusters associated with the identified AA sequence. In case of a labeled experiment this is the total intensity of all the isotopic patterns in the label cluster.
Intensity FL0145_JHendy_130416_MSQ525_CW18		Summed up eXtracted Ion Current (XIC) of all isotopic clusters associated with the identified AA sequence. In case of a labeled experiment this is the total intensity of all the isotopic patterns in the label cluster.
Intensity FL0145_JHendy_130416_MSQ525_CW20		Summed up eXtracted Ion Current (XIC) of all isotopic clusters associated with the identified AA sequence. In case of a labeled experiment this is the total intensity of all the isotopic patterns in the label cluster.
Intensity FL0145_JHendy_130416_MSQ525_CW21		Summed up eXtracted Ion Current (XIC) of all isotopic clusters associated with the identified AA sequence. In case of a labeled experiment this is the total intensity of all the isotopic patterns in the label cluster.
Intensity FL0145_JHendy_130416_MSQ525_CW23		Summed up eXtracted Ion Current (XIC) of all isotopic clusters associated with the identified AA sequence. In case of a labeled experiment this is the total intensity of all the isotopic patterns in the label cluster.

Intensity FL0145_JHendy_130416_MSQ525_CW8_160425165036		Summed up eXtracted Ion Current (XIC) of all isotopic clusters associated with the identified AA sequence. In case of a labeled experiment this is the total intensity of all the isotopic patterns in the label cluster.
Intensity FL0168_JHendy_MSQ525_170516_BK		Summed up eXtracted Ion Current (XIC) of all isotopic clusters associated with the identified AA sequence. In case of a labeled experiment this is the total intensity of all the isotopic patterns in the label cluster.
Intensity FL0168_JHendy_MSQ525_170516_CW1		Summed up eXtracted Ion Current (XIC) of all isotopic clusters associated with the identified AA sequence. In case of a labeled experiment this is the total intensity of all the isotopic patterns in the label cluster.
Intensity FL0168_JHendy_MSQ525_170516_CW10		Summed up eXtracted Ion Current (XIC) of all isotopic clusters associated with the identified AA sequence. In case of a labeled experiment this is the total intensity of all the isotopic patterns in the label cluster.
Intensity FL0168_JHendy_MSQ525_170516_CW2		Summed up eXtracted Ion Current (XIC) of all isotopic clusters associated with the identified AA sequence. In case of a labeled experiment this is the total intensity of all the isotopic patterns in the label cluster.
Intensity FL0168_JHendy_MSQ525_170516_CW3		Summed up eXtracted Ion Current (XIC) of all isotopic clusters associated with the identified AA sequence. In case of a labeled experiment this is the total intensity of all the isotopic patterns in the label cluster.
Intensity FL0168_JHendy_MSQ525_170516_CW4		Summed up eXtracted Ion Current (XIC) of all isotopic clusters associated with the identified AA sequence. In case of a labeled experiment this is the total intensity of all the isotopic patterns in the label cluster.
Intensity FL0168_JHendy_MSQ525_170516_CW5		Summed up eXtracted Ion Current (XIC) of all isotopic clusters associated with the identified AA sequence. In case of a labeled experiment this is the total intensity of all the isotopic patterns in the label cluster.
Intensity FL0168_JHendy_MSQ525_170516_CW6		Summed up eXtracted Ion Current (XIC) of all isotopic clusters associated with the identified AA sequence. In case of a labeled experiment this is the total intensity of all the isotopic patterns in the label cluster.
Intensity FL0168_JHendy_MSQ525_170516_CW7		Summed up eXtracted Ion Current (XIC) of all isotopic clusters associated with the identified AA sequence. In case of a labeled experiment this is the total intensity of all the isotopic patterns in the label cluster.
Intensity FL0168_JHendy_MSQ525_170516_CW8		Summed up eXtracted Ion Current (XIC) of all isotopic clusters associated with the identified AA sequence. In case of a labeled experiment this is the total intensity of all the isotopic patterns in the label cluster.
Intensity FL0168_JHendy_MSQ525_170516_CW9		Summed up eXtracted Ion Current (XIC) of all isotopic clusters associated with the identified AA sequence. In case of a labeled experiment this is the total intensity of all the isotopic patterns in the label cluster.
Reverse		When marked with '+', this particular peptide was found to be part of a protein derived from the reversed part of the decoy database. These should be removed for further data analysis.
Potential contaminant		When marked with '+', this particular peptide was found to be part of a commonly occurring contaminant. These should be removed for further data analysis.
id		A unique (consecutive) identifier for each row in the peptides table, which is used to cross-link the information in this table with the information stored in the other tables.
Protein group IDs		The identifiers of the protein groups this peptide was linked to, referenced against the proteinGroups table.
Peptide ID		Identifier of the associated peptide sequence summary, which can be found in the file 'peptides.txt'.
Evidence IDs		Identifier(s) for analyzed peptide evidence associated with the protein group referenced against the evidence table.
MS/MS IDs		The identifiers of the MS/MS scans identifying this peptide, referenced against the msms table.
Best MS/MS		The identifier of the best (in terms of quality) MS/MS scan identifying this peptide, referenced against the msms table.
Deamidation (NQ) site IDs		Identifier(s) for site(s) associated with this peptide, which show(s) evidence of the modification, referenced against the appropriate modification site file.
Hydroxyproline site IDs		Identifier(s) for site(s) associated with this peptide, which show(s) evidence of the modification, referenced against the appropriate modification site file.
Oxidation (M) site IDs		Identifier(s) for site(s) associated with this peptide, which show(s) evidence of the modification, referenced against the appropriate modification site file.
Propionamide K site IDs		Identifier(s) for site(s) associated with this peptide, which show(s) evidence of the modification, referenced against the appropriate modification site file.
MS/MS Count		

Deamidation (NQ)Sites

Name	Separator	Description
Proteins		Identifiers of proteins this site is associated with.
Positions within proteins		For each protein identifier in the 'Proteins' column you find here the position of the site in the respective protein sequence. The index of the first amino acid in the sequence is 1.
Leading proteins		
Protein		Identifier of the protein this peptide is associated with.
Fasta headers		Descriptions of proteins this peptide is associated with.
Localization prob		
Score diff		
PEP		The posterior error probability (PEP) of the best identified modified peptide containing this site.
Score		The Andromeda score of the best identified modified peptide containing this site.
Delta score		The Andromeda delta score of the best identified modified peptide containing this site.
Score for localization		The Andromeda score of the MS/MS spectrum used for calculating the localization score for this site.
Localization prob 20160805_QE5_nLC7_MEM_COLLAB_JH_A1_01		
Score diff 20160805_QE5_nLC7_MEM_COLLAB_JH_A1_01		
PEP 20160805_QE5_nLC7_MEM_COLLAB_JH_A1_01		
Score 20160805_QE5_nLC7_MEM_COLLAB_JH_A1_01		
Localization prob 20160805_QE5_nLC7_MEM_COLLAB_JH_A2_01		
Score diff 20160805_QE5_nLC7_MEM_COLLAB_JH_A2_01		
PEP 20160805_QE5_nLC7_MEM_COLLAB_JH_A2_01		
Score 20160805_QE5_nLC7_MEM_COLLAB_JH_A2_01		
Localization prob 20160805_QE5_nLC7_MEM_COLLAB_JH_A3_01		
Score diff 20160805_QE5_nLC7_MEM_COLLAB_JH_A3_01		
PEP 20160805_QE5_nLC7_MEM_COLLAB_JH_A3_01		
Score 20160805_QE5_nLC7_MEM_COLLAB_JH_A3_01		
Localization prob 20160805_QE5_nLC7_MEM_COLLAB_JH_B1_01		
Score diff 20160805_QE5_nLC7_MEM_COLLAB_JH_B1_01		
PEP 20160805_QE5_nLC7_MEM_COLLAB_JH_B1_01		
Score 20160805_QE5_nLC7_MEM_COLLAB_JH_B1_01		
Localization prob 20160805_QE5_nLC7_MEM_COLLAB_JH_B2_01		
Score diff 20160805_QE5_nLC7_MEM_COLLAB_JH_B2_01		

PEP 20160805_QE5_nLC7_MEM_COLLAB_JH_B2_01		
Score 20160805_QE5_nLC7_MEM_COLLAB_JH_B2_01		
Localization prob 20160805_QE5_nLC7_MEM_COLLAB_JH_B3_01		
Score diff 20160805_QE5_nLC7_MEM_COLLAB_JH_B3_01		
PEP 20160805_QE5_nLC7_MEM_COLLAB_JH_B3_01		
Score 20160805_QE5_nLC7_MEM_COLLAB_JH_B3_01		
Localization prob 20160805_QE5_nLC7_MEM_COLLAB_JH_B4_01		
Score diff 20160805_QE5_nLC7_MEM_COLLAB_JH_B4_01		
PEP 20160805_QE5_nLC7_MEM_COLLAB_JH_B4_01		
Score 20160805_QE5_nLC7_MEM_COLLAB_JH_B4_01		
Localization prob 20160805_QE5_nLC7_MEM_COLLAB_JH_B5_01		
Score diff 20160805_QE5_nLC7_MEM_COLLAB_JH_B5_01		
PEP 20160805_QE5_nLC7_MEM_COLLAB_JH_B5_01		
Score 20160805_QE5_nLC7_MEM_COLLAB_JH_B5_01		
Localization prob 20160805_QE5_nLC7_MEM_COLLAB_JH_C1_01		
Score diff 20160805_QE5_nLC7_MEM_COLLAB_JH_C1_01		
PEP 20160805_QE5_nLC7_MEM_COLLAB_JH_C1_01		
Score 20160805_QE5_nLC7_MEM_COLLAB_JH_C1_01		
Localization prob 20160805_QE5_nLC7_MEM_COLLAB_JH_C2_01		
Score diff 20160805_QE5_nLC7_MEM_COLLAB_JH_C2_01		
PEP 20160805_QE5_nLC7_MEM_COLLAB_JH_C2_01		
Score 20160805_QE5_nLC7_MEM_COLLAB_JH_C2_01		
Localization prob 20160805_QE5_nLC7_MEM_COLLAB_JH_C3_01		
Score diff 20160805_QE5_nLC7_MEM_COLLAB_JH_C3_01		
PEP 20160805_QE5_nLC7_MEM_COLLAB_JH_C3_01		
Score 20160805_QE5_nLC7_MEM_COLLAB_JH_C3_01		
Localization prob 20160805_QE5_nLC7_MEM_COLLAB_JH_C4_01		

Score diff 20160805_QE5_nLC7_MEM_COLLAB_JH_C4_01		
PEP 20160805_QE5_nLC7_MEM_COLLAB_JH_C4_01		
Score 20160805_QE5_nLC7_MEM_COLLAB_JH_C4_01		
Localization prob 20160805_QE5_nLC7_MEM_COLLAB_JH_C5_01		
Score diff 20160805_QE5_nLC7_MEM_COLLAB_JH_C5_01		
PEP 20160805_QE5_nLC7_MEM_COLLAB_JH_C5_01		
Score 20160805_QE5_nLC7_MEM_COLLAB_JH_C5_01		
Localization prob 20160805_QE5_nLC7_MEM_COLLAB_JH_C6_01		
Score diff 20160805_QE5_nLC7_MEM_COLLAB_JH_C6_01		
PEP 20160805_QE5_nLC7_MEM_COLLAB_JH_C6_01		
Score 20160805_QE5_nLC7_MEM_COLLAB_JH_C6_01		
Localization prob 20160805_QE5_nLC7_MEM_COLLAB_JH_C7_01		
Score diff 20160805_QE5_nLC7_MEM_COLLAB_JH_C7_01		
PEP 20160805_QE5_nLC7_MEM_COLLAB_JH_C7_01		
Score 20160805_QE5_nLC7_MEM_COLLAB_JH_C7_01		
Localization prob 20160805_QE5_nLC7_MEM_COLLAB_JH_C8_01		
Score diff 20160805_QE5_nLC7_MEM_COLLAB_JH_C8_01		
PEP 20160805_QE5_nLC7_MEM_COLLAB_JH_C8_01		
Score 20160805_QE5_nLC7_MEM_COLLAB_JH_C8_01		
Localization prob FL0145_JHendy_130416_MSQ525_CW10		
Score diff FL0145_JHendy_130416_MSQ525_CW10		
PEP FL0145_JHendy_130416_MSQ525_CW10		
Score FL0145_JHendy_130416_MSQ525_CW10		
Localization prob FL0145_JHendy_130416_MSQ525_CW11		
Score diff FL0145_JHendy_130416_MSQ525_CW11		
PEP FL0145_JHendy_130416_MSQ525_CW11		
Score FL0145_JHendy_130416_MSQ525_CW11		

Localization prob FL0145_JHendy_130416_MSQ525_CW18		
Score diff FL0145_JHendy_130416_MSQ525_CW18		
PEP FL0145_JHendy_130416_MSQ525_CW18		
Score FL0145_JHendy_130416_MSQ525_CW18		
Localization prob FL0145_JHendy_130416_MSQ525_CW20		
Score diff FL0145_JHendy_130416_MSQ525_CW20		
PEP FL0145_JHendy_130416_MSQ525_CW20		
Score FL0145_JHendy_130416_MSQ525_CW20		
Localization prob FL0145_JHendy_130416_MSQ525_CW21		
Score diff FL0145_JHendy_130416_MSQ525_CW21		
PEP FL0145_JHendy_130416_MSQ525_CW21		
Score FL0145_JHendy_130416_MSQ525_CW21		
Localization prob FL0145_JHendy_130416_MSQ525_CW23		
Score diff FL0145_JHendy_130416_MSQ525_CW23		
PEP FL0145_JHendy_130416_MSQ525_CW23		
Score FL0145_JHendy_130416_MSQ525_CW23		
Localization prob FL0145_JHendy_130416_MSQ525_CW8_160425165036		
Score diff FL0145_JHendy_130416_MSQ525_CW8_160425165036		
PEP FL0145_JHendy_130416_MSQ525_CW8_160425165036		
Score FL0145_JHendy_130416_MSQ525_CW8_160425165036		
Localization prob FL0168_JHendy_MSQ525_170516_BK		
Score diff FL0168_JHendy_MSQ525_170516_BK		
PEP FL0168_JHendy_MSQ525_170516_BK		
Score FL0168_JHendy_MSQ525_170516_BK		
Localization prob FL0168_JHendy_MSQ525_170516_CW1		
Score diff FL0168_JHendy_MSQ525_170516_CW1		
PEP FL0168_JHendy_MSQ525_170516_CW1		

Score FL0168_JHendy_MSQ525_170516_CW1		
Localization prob FL0168_JHendy_MSQ525_170516_CW10		
Score diff FL0168_JHendy_MSQ525_170516_CW10		
PEP FL0168_JHendy_MSQ525_170516_CW10		
Score FL0168_JHendy_MSQ525_170516_CW10		
Localization prob FL0168_JHendy_MSQ525_170516_CW2		
Score diff FL0168_JHendy_MSQ525_170516_CW2		
PEP FL0168_JHendy_MSQ525_170516_CW2		
Score FL0168_JHendy_MSQ525_170516_CW2		
Localization prob FL0168_JHendy_MSQ525_170516_CW3		
Score diff FL0168_JHendy_MSQ525_170516_CW3		
PEP FL0168_JHendy_MSQ525_170516_CW3		
Score FL0168_JHendy_MSQ525_170516_CW3		
Localization prob FL0168_JHendy_MSQ525_170516_CW4		
Score diff FL0168_JHendy_MSQ525_170516_CW4		
PEP FL0168_JHendy_MSQ525_170516_CW4		
Score FL0168_JHendy_MSQ525_170516_CW4		
Localization prob FL0168_JHendy_MSQ525_170516_CW5		
Score diff FL0168_JHendy_MSQ525_170516_CW5		
PEP FL0168_JHendy_MSQ525_170516_CW5		
Score FL0168_JHendy_MSQ525_170516_CW5		
Localization prob FL0168_JHendy_MSQ525_170516_CW6		
Score diff FL0168_JHendy_MSQ525_170516_CW6		
PEP FL0168_JHendy_MSQ525_170516_CW6		
Score FL0168_JHendy_MSQ525_170516_CW6		
Localization prob FL0168_JHendy_MSQ525_170516_CW7		
Score diff FL0168_JHendy_MSQ525_170516_CW7		

PEP FL0168_JHendy_MSQ525_170516_CW7		
Score FL0168_JHendy_MSQ525_170516_CW7		
Localization prob FL0168_JHendy_MSQ525_170516_CW8		
Score diff FL0168_JHendy_MSQ525_170516_CW8		
PEP FL0168_JHendy_MSQ525_170516_CW8		
Score FL0168_JHendy_MSQ525_170516_CW8		
Localization prob FL0168_JHendy_MSQ525_170516_CW9		
Score diff FL0168_JHendy_MSQ525_170516_CW9		
PEP FL0168_JHendy_MSQ525_170516_CW9		
Score FL0168_JHendy_MSQ525_170516_CW9		
Diagnostic peak		
Number of Deamidation (NQ)		Different numbers of Deamidation (NQ) on peptides that this site is involved in.
Amino acid		
Sequence window		
Modification window		
Peptide window coverage		
Deamidation (NQ) Probabilities		
Deamidation (NQ) Score diffs		
Position in peptide		
Charge		Charge state of the precursor ion.
Mass error [ppm]		Mass error of the recalibrated mass-over-charge value of the precursor ion in comparison to the predicted monoisotopic mass of the identified peptide sequence.
Intensity		Summed up eXtracted Ion Current (XIC) of all isotopic clusters associated with the identified AA sequence. In case of a labeled experiment this is the total intensity of all the isotopic patterns in the label cluster.
Intensity___1		Summed up eXtracted Ion Current (XIC) of all isotopic clusters associated with the identified AA sequence. In case of a labeled experiment this is the total intensity of all the isotopic patterns in the label cluster.
Intensity___2		Summed up eXtracted Ion Current (XIC) of all isotopic clusters associated with the identified AA sequence. In case of a labeled experiment this is the total intensity of all the isotopic patterns in the label cluster.
Intensity___3		Summed up eXtracted Ion Current (XIC) of all isotopic clusters associated with the identified AA sequence. In case of a labeled experiment this is the total intensity of all the isotopic patterns in the label cluster.
Ratio mod/base		
Intensity 20160805_QE5_nLC7_MEM_COLLAB_JH_A1_01		Summed up eXtracted Ion Current (XIC) of all isotopic clusters associated with the identified AA sequence. In case of a labeled experiment this is the total intensity of all the isotopic patterns in the label cluster.
Intensity 20160805_QE5_nLC7_MEM_COLLAB_JH_A2_01		Summed up eXtracted Ion Current (XIC) of all isotopic clusters associated with the identified AA sequence. In case of a labeled experiment this is the total intensity of all the isotopic patterns in the label cluster.
Intensity 20160805_QE5_nLC7_MEM_COLLAB_JH_A3_01		Summed up eXtracted Ion Current (XIC) of all isotopic clusters associated with the identified AA sequence. In case of a labeled experiment this is the total intensity of all the isotopic patterns in the label cluster.
Intensity 20160805_QE5_nLC7_MEM_COLLAB_JH_B1_01		Summed up eXtracted Ion Current (XIC) of all isotopic clusters associated with the identified AA sequence. In case of a labeled experiment this is the total intensity of all the isotopic patterns in the label cluster.

Intensity 20160805_QE5_nLC7_MEM_COLLAB_JH_B2_01		Summed up eXtracted Ion Current (XIC) of all isotopic clusters associated with the identified AA sequence. In case of a labeled experiment this is the total intensity of all the isotopic patterns in the label cluster.
Intensity 20160805_QE5_nLC7_MEM_COLLAB_JH_B3_01		Summed up eXtracted Ion Current (XIC) of all isotopic clusters associated with the identified AA sequence. In case of a labeled experiment this is the total intensity of all the isotopic patterns in the label cluster.
Intensity 20160805_QE5_nLC7_MEM_COLLAB_JH_B4_01		Summed up eXtracted Ion Current (XIC) of all isotopic clusters associated with the identified AA sequence. In case of a labeled experiment this is the total intensity of all the isotopic patterns in the label cluster.
Intensity 20160805_QE5_nLC7_MEM_COLLAB_JH_B5_01		Summed up eXtracted Ion Current (XIC) of all isotopic clusters associated with the identified AA sequence. In case of a labeled experiment this is the total intensity of all the isotopic patterns in the label cluster.
Intensity 20160805_QE5_nLC7_MEM_COLLAB_JH_C1_01		Summed up eXtracted Ion Current (XIC) of all isotopic clusters associated with the identified AA sequence. In case of a labeled experiment this is the total intensity of all the isotopic patterns in the label cluster.
Intensity 20160805_QE5_nLC7_MEM_COLLAB_JH_C2_01		Summed up eXtracted Ion Current (XIC) of all isotopic clusters associated with the identified AA sequence. In case of a labeled experiment this is the total intensity of all the isotopic patterns in the label cluster.
Intensity 20160805_QE5_nLC7_MEM_COLLAB_JH_C3_01		Summed up eXtracted Ion Current (XIC) of all isotopic clusters associated with the identified AA sequence. In case of a labeled experiment this is the total intensity of all the isotopic patterns in the label cluster.
Intensity 20160805_QE5_nLC7_MEM_COLLAB_JH_C4_01		Summed up eXtracted Ion Current (XIC) of all isotopic clusters associated with the identified AA sequence. In case of a labeled experiment this is the total intensity of all the isotopic patterns in the label cluster.
Intensity 20160805_QE5_nLC7_MEM_COLLAB_JH_C5_01		Summed up eXtracted Ion Current (XIC) of all isotopic clusters associated with the identified AA sequence. In case of a labeled experiment this is the total intensity of all the isotopic patterns in the label cluster.
Intensity 20160805_QE5_nLC7_MEM_COLLAB_JH_C6_01		Summed up eXtracted Ion Current (XIC) of all isotopic clusters associated with the identified AA sequence. In case of a labeled experiment this is the total intensity of all the isotopic patterns in the label cluster.
Intensity 20160805_QE5_nLC7_MEM_COLLAB_JH_C7_01		Summed up eXtracted Ion Current (XIC) of all isotopic clusters associated with the identified AA sequence. In case of a labeled experiment this is the total intensity of all the isotopic patterns in the label cluster.
Intensity 20160805_QE5_nLC7_MEM_COLLAB_JH_C8_01		Summed up eXtracted Ion Current (XIC) of all isotopic clusters associated with the identified AA sequence. In case of a labeled experiment this is the total intensity of all the isotopic patterns in the label cluster.
Intensity FL0145_JHendy_130416_MSQ525_CW10		Summed up eXtracted Ion Current (XIC) of all isotopic clusters associated with the identified AA sequence. In case of a labeled experiment this is the total intensity of all the isotopic patterns in the label cluster.
Intensity FL0145_JHendy_130416_MSQ525_CW11		Summed up eXtracted Ion Current (XIC) of all isotopic clusters associated with the identified AA sequence. In case of a labeled experiment this is the total intensity of all the isotopic patterns in the label cluster.
Intensity FL0145_JHendy_130416_MSQ525_CW18		Summed up eXtracted Ion Current (XIC) of all isotopic clusters associated with the identified AA sequence. In case of a labeled experiment this is the total intensity of all the isotopic patterns in the label cluster.
Intensity FL0145_JHendy_130416_MSQ525_CW20		Summed up eXtracted Ion Current (XIC) of all isotopic clusters associated with the identified AA sequence. In case of a labeled experiment this is the total intensity of all the isotopic patterns in the label cluster.
Intensity FL0145_JHendy_130416_MSQ525_CW21		Summed up eXtracted Ion Current (XIC) of all isotopic clusters associated with the identified AA sequence. In case of a labeled experiment this is the total intensity of all the isotopic patterns in the label cluster.
Intensity FL0145_JHendy_130416_MSQ525_CW23		Summed up eXtracted Ion Current (XIC) of all isotopic clusters associated with the identified AA sequence. In case of a labeled experiment this is the total intensity of all the isotopic patterns in the label cluster.
Intensity FL0145_JHendy_130416_MSQ525_CW8_160425165036		Summed up eXtracted Ion Current (XIC) of all isotopic clusters associated with the identified AA sequence. In case of a labeled experiment this is the total intensity of all the isotopic patterns in the label cluster.
Intensity FL0168_JHendy_MSQ525_170516_BK		Summed up eXtracted Ion Current (XIC) of all isotopic clusters associated with the identified AA sequence. In case of a labeled experiment this is the total intensity of all the isotopic patterns in the label cluster.
Intensity FL0168_JHendy_MSQ525_170516_CW1		Summed up eXtracted Ion Current (XIC) of all isotopic clusters associated with the identified AA sequence. In case of a labeled experiment this is the total intensity of all the isotopic patterns in the label cluster.

Intensity FL0168_JHendy_MSQ525_170516_CW10		Summed up eXtracted Ion Current (XIC) of all isotopic clusters associated with the identified AA sequence. In case of a labeled experiment this is the total intensity of all the isotopic patterns in the label cluster.
Intensity FL0168_JHendy_MSQ525_170516_CW2		Summed up eXtracted Ion Current (XIC) of all isotopic clusters associated with the identified AA sequence. In case of a labeled experiment this is the total intensity of all the isotopic patterns in the label cluster.
Intensity FL0168_JHendy_MSQ525_170516_CW3		Summed up eXtracted Ion Current (XIC) of all isotopic clusters associated with the identified AA sequence. In case of a labeled experiment this is the total intensity of all the isotopic patterns in the label cluster.
Intensity FL0168_JHendy_MSQ525_170516_CW4		Summed up eXtracted Ion Current (XIC) of all isotopic clusters associated with the identified AA sequence. In case of a labeled experiment this is the total intensity of all the isotopic patterns in the label cluster.
Intensity FL0168_JHendy_MSQ525_170516_CW5		Summed up eXtracted Ion Current (XIC) of all isotopic clusters associated with the identified AA sequence. In case of a labeled experiment this is the total intensity of all the isotopic patterns in the label cluster.
Intensity FL0168_JHendy_MSQ525_170516_CW6		Summed up eXtracted Ion Current (XIC) of all isotopic clusters associated with the identified AA sequence. In case of a labeled experiment this is the total intensity of all the isotopic patterns in the label cluster.
Intensity FL0168_JHendy_MSQ525_170516_CW7		Summed up eXtracted Ion Current (XIC) of all isotopic clusters associated with the identified AA sequence. In case of a labeled experiment this is the total intensity of all the isotopic patterns in the label cluster.
Intensity FL0168_JHendy_MSQ525_170516_CW8		Summed up eXtracted Ion Current (XIC) of all isotopic clusters associated with the identified AA sequence. In case of a labeled experiment this is the total intensity of all the isotopic patterns in the label cluster.
Intensity FL0168_JHendy_MSQ525_170516_CW9		Summed up eXtracted Ion Current (XIC) of all isotopic clusters associated with the identified AA sequence. In case of a labeled experiment this is the total intensity of all the isotopic patterns in the label cluster.
Ratio mod/base 20160805_QE5_nLC7_MEM_COLLAB_JH_A1_01		
Ratio mod/base 20160805_QE5_nLC7_MEM_COLLAB_JH_A2_01		
Ratio mod/base 20160805_QE5_nLC7_MEM_COLLAB_JH_A3_01		
Ratio mod/base 20160805_QE5_nLC7_MEM_COLLAB_JH_B1_01		
Ratio mod/base 20160805_QE5_nLC7_MEM_COLLAB_JH_B2_01		
Ratio mod/base 20160805_QE5_nLC7_MEM_COLLAB_JH_B3_01		
Ratio mod/base 20160805_QE5_nLC7_MEM_COLLAB_JH_B4_01		
Ratio mod/base 20160805_QE5_nLC7_MEM_COLLAB_JH_B5_01		
Ratio mod/base 20160805_QE5_nLC7_MEM_COLLAB_JH_C1_01		
Ratio mod/base 20160805_QE5_nLC7_MEM_COLLAB_JH_C2_01		
Ratio mod/base 20160805_QE5_nLC7_MEM_COLLAB_JH_C3_01		
Ratio mod/base 20160805_QE5_nLC7_MEM_COLLAB_JH_C4_01		
Ratio mod/base 20160805_QE5_nLC7_MEM_COLLAB_JH_C5_01		
Ratio mod/base 20160805_QE5_nLC7_MEM_COLLAB_JH_C6_01		
Ratio mod/base 20160805_QE5_nLC7_MEM_COLLAB_JH_C7_01		

Ratio mod/base 20160805_QE5_nLC7_MEM_COLLAB_JH_C8_01		
Ratio mod/base FL0145_JHendy_130416_MSQ525_CW10		
Ratio mod/base FL0145_JHendy_130416_MSQ525_CW11		
Ratio mod/base FL0145_JHendy_130416_MSQ525_CW18		
Ratio mod/base FL0145_JHendy_130416_MSQ525_CW20		
Ratio mod/base FL0145_JHendy_130416_MSQ525_CW21		
Ratio mod/base FL0145_JHendy_130416_MSQ525_CW23		
Ratio mod/base FL0145_JHendy_130416_MSQ525_CW8_160425165036		
Ratio mod/base FL0168_JHendy_MSQ525_170516_BK		
Ratio mod/base FL0168_JHendy_MSQ525_170516_CW1		
Ratio mod/base FL0168_JHendy_MSQ525_170516_CW10		
Ratio mod/base FL0168_JHendy_MSQ525_170516_CW2		
Ratio mod/base FL0168_JHendy_MSQ525_170516_CW3		
Ratio mod/base FL0168_JHendy_MSQ525_170516_CW4		
Ratio mod/base FL0168_JHendy_MSQ525_170516_CW5		
Ratio mod/base FL0168_JHendy_MSQ525_170516_CW6		
Ratio mod/base FL0168_JHendy_MSQ525_170516_CW7		
Ratio mod/base FL0168_JHendy_MSQ525_170516_CW8		
Ratio mod/base FL0168_JHendy_MSQ525_170516_CW9		
Intensity 20160805_QE5_nLC7_MEM_COLLAB_JH_A1_01___1		Summed up eXtracted Ion Current (XIC) of all isotopic clusters associated with the identified AA sequence. In case of a labeled experiment this is the total intensity of all the isotopic patterns in the label cluster.
Intensity 20160805_QE5_nLC7_MEM_COLLAB_JH_A1_01___2		Summed up eXtracted Ion Current (XIC) of all isotopic clusters associated with the identified AA sequence. In case of a labeled experiment this is the total intensity of all the isotopic patterns in the label cluster.
Intensity 20160805_QE5_nLC7_MEM_COLLAB_JH_A1_01___3		Summed up eXtracted Ion Current (XIC) of all isotopic clusters associated with the identified AA sequence. In case of a labeled experiment this is the total intensity of all the isotopic patterns in the label cluster.
Intensity 20160805_QE5_nLC7_MEM_COLLAB_JH_A2_01___1		Summed up eXtracted Ion Current (XIC) of all isotopic clusters associated with the identified AA sequence. In case of a labeled experiment this is the total intensity of all the isotopic patterns in the label cluster.
Intensity 20160805_QE5_nLC7_MEM_COLLAB_JH_A2_01___2		Summed up eXtracted Ion Current (XIC) of all isotopic clusters associated with the identified AA sequence. In case of a labeled experiment this is the total intensity of all the isotopic patterns in the label cluster.
Intensity 20160805_QE5_nLC7_MEM_COLLAB_JH_A2_01___3		Summed up eXtracted Ion Current (XIC) of all isotopic clusters associated with the identified AA sequence. In case of a labeled experiment this is the total intensity of all the isotopic patterns in the label cluster.

Intensity 20160805_QE5_nLC7_MEM_COLLAB_JH_A3_01___1		Summed up eXtracted Ion Current (XIC) of all isotopic clusters associated with the identified AA sequence. In case of a labeled experiment this is the total intensity of all the isotopic patterns in the label cluster.
Intensity 20160805_QE5_nLC7_MEM_COLLAB_JH_A3_01___2		Summed up eXtracted Ion Current (XIC) of all isotopic clusters associated with the identified AA sequence. In case of a labeled experiment this is the total intensity of all the isotopic patterns in the label cluster.
Intensity 20160805_QE5_nLC7_MEM_COLLAB_JH_A3_01___3		Summed up eXtracted Ion Current (XIC) of all isotopic clusters associated with the identified AA sequence. In case of a labeled experiment this is the total intensity of all the isotopic patterns in the label cluster.
Intensity 20160805_QE5_nLC7_MEM_COLLAB_JH_B1_01___1		Summed up eXtracted Ion Current (XIC) of all isotopic clusters associated with the identified AA sequence. In case of a labeled experiment this is the total intensity of all the isotopic patterns in the label cluster.
Intensity 20160805_QE5_nLC7_MEM_COLLAB_JH_B1_01___2		Summed up eXtracted Ion Current (XIC) of all isotopic clusters associated with the identified AA sequence. In case of a labeled experiment this is the total intensity of all the isotopic patterns in the label cluster.
Intensity 20160805_QE5_nLC7_MEM_COLLAB_JH_B1_01___3		Summed up eXtracted Ion Current (XIC) of all isotopic clusters associated with the identified AA sequence. In case of a labeled experiment this is the total intensity of all the isotopic patterns in the label cluster.
Intensity 20160805_QE5_nLC7_MEM_COLLAB_JH_B2_01___1		Summed up eXtracted Ion Current (XIC) of all isotopic clusters associated with the identified AA sequence. In case of a labeled experiment this is the total intensity of all the isotopic patterns in the label cluster.
Intensity 20160805_QE5_nLC7_MEM_COLLAB_JH_B2_01___2		Summed up eXtracted Ion Current (XIC) of all isotopic clusters associated with the identified AA sequence. In case of a labeled experiment this is the total intensity of all the isotopic patterns in the label cluster.
Intensity 20160805_QE5_nLC7_MEM_COLLAB_JH_B2_01___3		Summed up eXtracted Ion Current (XIC) of all isotopic clusters associated with the identified AA sequence. In case of a labeled experiment this is the total intensity of all the isotopic patterns in the label cluster.
Intensity 20160805_QE5_nLC7_MEM_COLLAB_JH_B3_01___1		Summed up eXtracted Ion Current (XIC) of all isotopic clusters associated with the identified AA sequence. In case of a labeled experiment this is the total intensity of all the isotopic patterns in the label cluster.
Intensity 20160805_QE5_nLC7_MEM_COLLAB_JH_B3_01___2		Summed up eXtracted Ion Current (XIC) of all isotopic clusters associated with the identified AA sequence. In case of a labeled experiment this is the total intensity of all the isotopic patterns in the label cluster.
Intensity 20160805_QE5_nLC7_MEM_COLLAB_JH_B3_01___3		Summed up eXtracted Ion Current (XIC) of all isotopic clusters associated with the identified AA sequence. In case of a labeled experiment this is the total intensity of all the isotopic patterns in the label cluster.
Intensity 20160805_QE5_nLC7_MEM_COLLAB_JH_B4_01___1		Summed up eXtracted Ion Current (XIC) of all isotopic clusters associated with the identified AA sequence. In case of a labeled experiment this is the total intensity of all the isotopic patterns in the label cluster.
Intensity 20160805_QE5_nLC7_MEM_COLLAB_JH_B4_01___2		Summed up eXtracted Ion Current (XIC) of all isotopic clusters associated with the identified AA sequence. In case of a labeled experiment this is the total intensity of all the isotopic patterns in the label cluster.
Intensity 20160805_QE5_nLC7_MEM_COLLAB_JH_B4_01___3		Summed up eXtracted Ion Current (XIC) of all isotopic clusters associated with the identified AA sequence. In case of a labeled experiment this is the total intensity of all the isotopic patterns in the label cluster.
Intensity 20160805_QE5_nLC7_MEM_COLLAB_JH_B5_01___1		Summed up eXtracted Ion Current (XIC) of all isotopic clusters associated with the identified AA sequence. In case of a labeled experiment this is the total intensity of all the isotopic patterns in the label cluster.
Intensity 20160805_QE5_nLC7_MEM_COLLAB_JH_B5_01___2		Summed up eXtracted Ion Current (XIC) of all isotopic clusters associated with the identified AA sequence. In case of a labeled experiment this is the total intensity of all the isotopic patterns in the label cluster.
Intensity 20160805_QE5_nLC7_MEM_COLLAB_JH_B5_01___3		Summed up eXtracted Ion Current (XIC) of all isotopic clusters associated with the identified AA sequence. In case of a labeled experiment this is the total intensity of all the isotopic patterns in the label cluster.
Intensity 20160805_QE5_nLC7_MEM_COLLAB_JH_C1_01___1		Summed up eXtracted Ion Current (XIC) of all isotopic clusters associated with the identified AA sequence. In case of a labeled experiment this is the total intensity of all the isotopic patterns in the label cluster.
Intensity 20160805_QE5_nLC7_MEM_COLLAB_JH_C1_01___2		Summed up eXtracted Ion Current (XIC) of all isotopic clusters associated with the identified AA sequence. In case of a labeled experiment this is the total intensity of all the isotopic patterns in the label cluster.
Intensity 20160805_QE5_nLC7_MEM_COLLAB_JH_C1_01___3		Summed up eXtracted Ion Current (XIC) of all isotopic clusters associated with the identified AA sequence. In case of a labeled experiment this is the total intensity of all the isotopic patterns in the label cluster.

Intensity 20160805_QE5_nLC7_MEM_COLLAB_JH_C2_01___1		Summed up eXtracted Ion Current (XIC) of all isotopic clusters associated with the identified AA sequence. In case of a labeled experiment this is the total intensity of all the isotopic patterns in the label cluster.
Intensity 20160805_QE5_nLC7_MEM_COLLAB_JH_C2_01___2		Summed up eXtracted Ion Current (XIC) of all isotopic clusters associated with the identified AA sequence. In case of a labeled experiment this is the total intensity of all the isotopic patterns in the label cluster.
Intensity 20160805_QE5_nLC7_MEM_COLLAB_JH_C2_01___3		Summed up eXtracted Ion Current (XIC) of all isotopic clusters associated with the identified AA sequence. In case of a labeled experiment this is the total intensity of all the isotopic patterns in the label cluster.
Intensity 20160805_QE5_nLC7_MEM_COLLAB_JH_C3_01___1		Summed up eXtracted Ion Current (XIC) of all isotopic clusters associated with the identified AA sequence. In case of a labeled experiment this is the total intensity of all the isotopic patterns in the label cluster.
Intensity 20160805_QE5_nLC7_MEM_COLLAB_JH_C3_01___2		Summed up eXtracted Ion Current (XIC) of all isotopic clusters associated with the identified AA sequence. In case of a labeled experiment this is the total intensity of all the isotopic patterns in the label cluster.
Intensity 20160805_QE5_nLC7_MEM_COLLAB_JH_C3_01___3		Summed up eXtracted Ion Current (XIC) of all isotopic clusters associated with the identified AA sequence. In case of a labeled experiment this is the total intensity of all the isotopic patterns in the label cluster.
Intensity 20160805_QE5_nLC7_MEM_COLLAB_JH_C4_01___1		Summed up eXtracted Ion Current (XIC) of all isotopic clusters associated with the identified AA sequence. In case of a labeled experiment this is the total intensity of all the isotopic patterns in the label cluster.
Intensity 20160805_QE5_nLC7_MEM_COLLAB_JH_C4_01___2		Summed up eXtracted Ion Current (XIC) of all isotopic clusters associated with the identified AA sequence. In case of a labeled experiment this is the total intensity of all the isotopic patterns in the label cluster.
Intensity 20160805_QE5_nLC7_MEM_COLLAB_JH_C4_01___3		Summed up eXtracted Ion Current (XIC) of all isotopic clusters associated with the identified AA sequence. In case of a labeled experiment this is the total intensity of all the isotopic patterns in the label cluster.
Intensity 20160805_QE5_nLC7_MEM_COLLAB_JH_C5_01___1		Summed up eXtracted Ion Current (XIC) of all isotopic clusters associated with the identified AA sequence. In case of a labeled experiment this is the total intensity of all the isotopic patterns in the label cluster.
Intensity 20160805_QE5_nLC7_MEM_COLLAB_JH_C5_01___2		Summed up eXtracted Ion Current (XIC) of all isotopic clusters associated with the identified AA sequence. In case of a labeled experiment this is the total intensity of all the isotopic patterns in the label cluster.
Intensity 20160805_QE5_nLC7_MEM_COLLAB_JH_C5_01___3		Summed up eXtracted Ion Current (XIC) of all isotopic clusters associated with the identified AA sequence. In case of a labeled experiment this is the total intensity of all the isotopic patterns in the label cluster.
Intensity 20160805_QE5_nLC7_MEM_COLLAB_JH_C6_01___1		Summed up eXtracted Ion Current (XIC) of all isotopic clusters associated with the identified AA sequence. In case of a labeled experiment this is the total intensity of all the isotopic patterns in the label cluster.
Intensity 20160805_QE5_nLC7_MEM_COLLAB_JH_C6_01___2		Summed up eXtracted Ion Current (XIC) of all isotopic clusters associated with the identified AA sequence. In case of a labeled experiment this is the total intensity of all the isotopic patterns in the label cluster.
Intensity 20160805_QE5_nLC7_MEM_COLLAB_JH_C6_01___3		Summed up eXtracted Ion Current (XIC) of all isotopic clusters associated with the identified AA sequence. In case of a labeled experiment this is the total intensity of all the isotopic patterns in the label cluster.
Intensity 20160805_QE5_nLC7_MEM_COLLAB_JH_C7_01___1		Summed up eXtracted Ion Current (XIC) of all isotopic clusters associated with the identified AA sequence. In case of a labeled experiment this is the total intensity of all the isotopic patterns in the label cluster.
Intensity 20160805_QE5_nLC7_MEM_COLLAB_JH_C7_01___2		Summed up eXtracted Ion Current (XIC) of all isotopic clusters associated with the identified AA sequence. In case of a labeled experiment this is the total intensity of all the isotopic patterns in the label cluster.
Intensity 20160805_QE5_nLC7_MEM_COLLAB_JH_C7_01___3		Summed up eXtracted Ion Current (XIC) of all isotopic clusters associated with the identified AA sequence. In case of a labeled experiment this is the total intensity of all the isotopic patterns in the label cluster.
Intensity 20160805_QE5_nLC7_MEM_COLLAB_JH_C8_01___1		Summed up eXtracted Ion Current (XIC) of all isotopic clusters associated with the identified AA sequence. In case of a labeled experiment this is the total intensity of all the isotopic patterns in the label cluster.
Intensity 20160805_QE5_nLC7_MEM_COLLAB_JH_C8_01___2		Summed up eXtracted Ion Current (XIC) of all isotopic clusters associated with the identified AA sequence. In case of a labeled experiment this is the total intensity of all the isotopic patterns in the label cluster.
Intensity 20160805_QE5_nLC7_MEM_COLLAB_JH_C8_01___3		Summed up eXtracted Ion Current (XIC) of all isotopic clusters associated with the identified AA sequence. In case of a labeled experiment this is the total intensity of all the isotopic patterns in the label cluster.

Intensity FL0145_JHendy_130416_MSQ525_CW10___1		Summed up eXtracted Ion Current (XIC) of all isotopic clusters associated with the identified AA sequence. In case of a labeled experiment this is the total intensity of all the isotopic patterns in the label cluster.
Intensity FL0145_JHendy_130416_MSQ525_CW10___2		Summed up eXtracted Ion Current (XIC) of all isotopic clusters associated with the identified AA sequence. In case of a labeled experiment this is the total intensity of all the isotopic patterns in the label cluster.
Intensity FL0145_JHendy_130416_MSQ525_CW10___3		Summed up eXtracted Ion Current (XIC) of all isotopic clusters associated with the identified AA sequence. In case of a labeled experiment this is the total intensity of all the isotopic patterns in the label cluster.
Intensity FL0145_JHendy_130416_MSQ525_CW11___1		Summed up eXtracted Ion Current (XIC) of all isotopic clusters associated with the identified AA sequence. In case of a labeled experiment this is the total intensity of all the isotopic patterns in the label cluster.
Intensity FL0145_JHendy_130416_MSQ525_CW11___2		Summed up eXtracted Ion Current (XIC) of all isotopic clusters associated with the identified AA sequence. In case of a labeled experiment this is the total intensity of all the isotopic patterns in the label cluster.
Intensity FL0145_JHendy_130416_MSQ525_CW11___3		Summed up eXtracted Ion Current (XIC) of all isotopic clusters associated with the identified AA sequence. In case of a labeled experiment this is the total intensity of all the isotopic patterns in the label cluster.
Intensity FL0145_JHendy_130416_MSQ525_CW18___1		Summed up eXtracted Ion Current (XIC) of all isotopic clusters associated with the identified AA sequence. In case of a labeled experiment this is the total intensity of all the isotopic patterns in the label cluster.
Intensity FL0145_JHendy_130416_MSQ525_CW18___2		Summed up eXtracted Ion Current (XIC) of all isotopic clusters associated with the identified AA sequence. In case of a labeled experiment this is the total intensity of all the isotopic patterns in the label cluster.
Intensity FL0145_JHendy_130416_MSQ525_CW18___3		Summed up eXtracted Ion Current (XIC) of all isotopic clusters associated with the identified AA sequence. In case of a labeled experiment this is the total intensity of all the isotopic patterns in the label cluster.
Intensity FL0145_JHendy_130416_MSQ525_CW20___1		Summed up eXtracted Ion Current (XIC) of all isotopic clusters associated with the identified AA sequence. In case of a labeled experiment this is the total intensity of all the isotopic patterns in the label cluster.
Intensity FL0145_JHendy_130416_MSQ525_CW20___2		Summed up eXtracted Ion Current (XIC) of all isotopic clusters associated with the identified AA sequence. In case of a labeled experiment this is the total intensity of all the isotopic patterns in the label cluster.
Intensity FL0145_JHendy_130416_MSQ525_CW20___3		Summed up eXtracted Ion Current (XIC) of all isotopic clusters associated with the identified AA sequence. In case of a labeled experiment this is the total intensity of all the isotopic patterns in the label cluster.
Intensity FL0145_JHendy_130416_MSQ525_CW21___1		Summed up eXtracted Ion Current (XIC) of all isotopic clusters associated with the identified AA sequence. In case of a labeled experiment this is the total intensity of all the isotopic patterns in the label cluster.
Intensity FL0145_JHendy_130416_MSQ525_CW21___2		Summed up eXtracted Ion Current (XIC) of all isotopic clusters associated with the identified AA sequence. In case of a labeled experiment this is the total intensity of all the isotopic patterns in the label cluster.
Intensity FL0145_JHendy_130416_MSQ525_CW21___3		Summed up eXtracted Ion Current (XIC) of all isotopic clusters associated with the identified AA sequence. In case of a labeled experiment this is the total intensity of all the isotopic patterns in the label cluster.
Intensity FL0145_JHendy_130416_MSQ525_CW23___1		Summed up eXtracted Ion Current (XIC) of all isotopic clusters associated with the identified AA sequence. In case of a labeled experiment this is the total intensity of all the isotopic patterns in the label cluster.
Intensity FL0145_JHendy_130416_MSQ525_CW23___2		Summed up eXtracted Ion Current (XIC) of all isotopic clusters associated with the identified AA sequence. In case of a labeled experiment this is the total intensity of all the isotopic patterns in the label cluster.
Intensity FL0145_JHendy_130416_MSQ525_CW23___3		Summed up eXtracted Ion Current (XIC) of all isotopic clusters associated with the identified AA sequence. In case of a labeled experiment this is the total intensity of all the isotopic patterns in the label cluster.
Intensity FL0145_JHendy_130416_MSQ525_CW8_160425165036___1		Summed up eXtracted Ion Current (XIC) of all isotopic clusters associated with the identified AA sequence. In case of a labeled experiment this is the total intensity of all the isotopic patterns in the label cluster.
Intensity FL0145_JHendy_130416_MSQ525_CW8_160425165036___2		Summed up eXtracted Ion Current (XIC) of all isotopic clusters associated with the identified AA sequence. In case of a labeled experiment this is the total intensity of all the isotopic patterns in the label cluster.
Intensity FL0145_JHendy_130416_MSQ525_CW8_160425165036___3		Summed up eXtracted Ion Current (XIC) of all isotopic clusters associated with the identified AA sequence. In case of a labeled experiment this is the total intensity of all the isotopic patterns in the label cluster.

Intensity FL0168_JHendy_MSQ525_170516_BK___1		Summed up eXtracted Ion Current (XIC) of all isotopic clusters associated with the identified AA sequence. In case of a labeled experiment this is the total intensity of all the isotopic patterns in the label cluster.
Intensity FL0168_JHendy_MSQ525_170516_BK___2		Summed up eXtracted Ion Current (XIC) of all isotopic clusters associated with the identified AA sequence. In case of a labeled experiment this is the total intensity of all the isotopic patterns in the label cluster.
Intensity FL0168_JHendy_MSQ525_170516_BK___3		Summed up eXtracted Ion Current (XIC) of all isotopic clusters associated with the identified AA sequence. In case of a labeled experiment this is the total intensity of all the isotopic patterns in the label cluster.
Intensity FL0168_JHendy_MSQ525_170516_CW1___1		Summed up eXtracted Ion Current (XIC) of all isotopic clusters associated with the identified AA sequence. In case of a labeled experiment this is the total intensity of all the isotopic patterns in the label cluster.
Intensity FL0168_JHendy_MSQ525_170516_CW1___2		Summed up eXtracted Ion Current (XIC) of all isotopic clusters associated with the identified AA sequence. In case of a labeled experiment this is the total intensity of all the isotopic patterns in the label cluster.
Intensity FL0168_JHendy_MSQ525_170516_CW1___3		Summed up eXtracted Ion Current (XIC) of all isotopic clusters associated with the identified AA sequence. In case of a labeled experiment this is the total intensity of all the isotopic patterns in the label cluster.
Intensity FL0168_JHendy_MSQ525_170516_CW10___1		Summed up eXtracted Ion Current (XIC) of all isotopic clusters associated with the identified AA sequence. In case of a labeled experiment this is the total intensity of all the isotopic patterns in the label cluster.
Intensity FL0168_JHendy_MSQ525_170516_CW10___2		Summed up eXtracted Ion Current (XIC) of all isotopic clusters associated with the identified AA sequence. In case of a labeled experiment this is the total intensity of all the isotopic patterns in the label cluster.
Intensity FL0168_JHendy_MSQ525_170516_CW10___3		Summed up eXtracted Ion Current (XIC) of all isotopic clusters associated with the identified AA sequence. In case of a labeled experiment this is the total intensity of all the isotopic patterns in the label cluster.
Intensity FL0168_JHendy_MSQ525_170516_CW2___1		Summed up eXtracted Ion Current (XIC) of all isotopic clusters associated with the identified AA sequence. In case of a labeled experiment this is the total intensity of all the isotopic patterns in the label cluster.
Intensity FL0168_JHendy_MSQ525_170516_CW2___2		Summed up eXtracted Ion Current (XIC) of all isotopic clusters associated with the identified AA sequence. In case of a labeled experiment this is the total intensity of all the isotopic patterns in the label cluster.
Intensity FL0168_JHendy_MSQ525_170516_CW2___3		Summed up eXtracted Ion Current (XIC) of all isotopic clusters associated with the identified AA sequence. In case of a labeled experiment this is the total intensity of all the isotopic patterns in the label cluster.
Intensity FL0168_JHendy_MSQ525_170516_CW3___1		Summed up eXtracted Ion Current (XIC) of all isotopic clusters associated with the identified AA sequence. In case of a labeled experiment this is the total intensity of all the isotopic patterns in the label cluster.
Intensity FL0168_JHendy_MSQ525_170516_CW3___2		Summed up eXtracted Ion Current (XIC) of all isotopic clusters associated with the identified AA sequence. In case of a labeled experiment this is the total intensity of all the isotopic patterns in the label cluster.
Intensity FL0168_JHendy_MSQ525_170516_CW3___3		Summed up eXtracted Ion Current (XIC) of all isotopic clusters associated with the identified AA sequence. In case of a labeled experiment this is the total intensity of all the isotopic patterns in the label cluster.
Intensity FL0168_JHendy_MSQ525_170516_CW4___1		Summed up eXtracted Ion Current (XIC) of all isotopic clusters associated with the identified AA sequence. In case of a labeled experiment this is the total intensity of all the isotopic patterns in the label cluster.
Intensity FL0168_JHendy_MSQ525_170516_CW4___2		Summed up eXtracted Ion Current (XIC) of all isotopic clusters associated with the identified AA sequence. In case of a labeled experiment this is the total intensity of all the isotopic patterns in the label cluster.
Intensity FL0168_JHendy_MSQ525_170516_CW4___3		Summed up eXtracted Ion Current (XIC) of all isotopic clusters associated with the identified AA sequence. In case of a labeled experiment this is the total intensity of all the isotopic patterns in the label cluster.
Intensity FL0168_JHendy_MSQ525_170516_CW5___1		Summed up eXtracted Ion Current (XIC) of all isotopic clusters associated with the identified AA sequence. In case of a labeled experiment this is the total intensity of all the isotopic patterns in the label cluster.
Intensity FL0168_JHendy_MSQ525_170516_CW5___2		Summed up eXtracted Ion Current (XIC) of all isotopic clusters associated with the identified AA sequence. In case of a labeled experiment this is the total intensity of all the isotopic patterns in the label cluster.
Intensity FL0168_JHendy_MSQ525_170516_CW5___3		Summed up eXtracted Ion Current (XIC) of all isotopic clusters associated with the identified AA sequence. In case of a labeled experiment this is the total intensity of all the isotopic patterns in the label cluster.

Intensity FL0168_JHendy_MSQ525_170516_CW6___1		Summed up eXtracted Ion Current (XIC) of all isotopic clusters associated with the identified AA sequence. In case of a labeled experiment this is the total intensity of all the isotopic patterns in the label cluster.
Intensity FL0168_JHendy_MSQ525_170516_CW6___2		Summed up eXtracted Ion Current (XIC) of all isotopic clusters associated with the identified AA sequence. In case of a labeled experiment this is the total intensity of all the isotopic patterns in the label cluster.
Intensity FL0168_JHendy_MSQ525_170516_CW6___3		Summed up eXtracted Ion Current (XIC) of all isotopic clusters associated with the identified AA sequence. In case of a labeled experiment this is the total intensity of all the isotopic patterns in the label cluster.
Intensity FL0168_JHendy_MSQ525_170516_CW7___1		Summed up eXtracted Ion Current (XIC) of all isotopic clusters associated with the identified AA sequence. In case of a labeled experiment this is the total intensity of all the isotopic patterns in the label cluster.
Intensity FL0168_JHendy_MSQ525_170516_CW7___2		Summed up eXtracted Ion Current (XIC) of all isotopic clusters associated with the identified AA sequence. In case of a labeled experiment this is the total intensity of all the isotopic patterns in the label cluster.
Intensity FL0168_JHendy_MSQ525_170516_CW7___3		Summed up eXtracted Ion Current (XIC) of all isotopic clusters associated with the identified AA sequence. In case of a labeled experiment this is the total intensity of all the isotopic patterns in the label cluster.
Intensity FL0168_JHendy_MSQ525_170516_CW8___1		Summed up eXtracted Ion Current (XIC) of all isotopic clusters associated with the identified AA sequence. In case of a labeled experiment this is the total intensity of all the isotopic patterns in the label cluster.
Intensity FL0168_JHendy_MSQ525_170516_CW8___2		Summed up eXtracted Ion Current (XIC) of all isotopic clusters associated with the identified AA sequence. In case of a labeled experiment this is the total intensity of all the isotopic patterns in the label cluster.
Intensity FL0168_JHendy_MSQ525_170516_CW8___3		Summed up eXtracted Ion Current (XIC) of all isotopic clusters associated with the identified AA sequence. In case of a labeled experiment this is the total intensity of all the isotopic patterns in the label cluster.
Intensity FL0168_JHendy_MSQ525_170516_CW9___1		Summed up eXtracted Ion Current (XIC) of all isotopic clusters associated with the identified AA sequence. In case of a labeled experiment this is the total intensity of all the isotopic patterns in the label cluster.
Intensity FL0168_JHendy_MSQ525_170516_CW9___2		Summed up eXtracted Ion Current (XIC) of all isotopic clusters associated with the identified AA sequence. In case of a labeled experiment this is the total intensity of all the isotopic patterns in the label cluster.
Intensity FL0168_JHendy_MSQ525_170516_CW9___3		Summed up eXtracted Ion Current (XIC) of all isotopic clusters associated with the identified AA sequence. In case of a labeled experiment this is the total intensity of all the isotopic patterns in the label cluster.
Occupancy 20160805_QE5_nLC7_MEM_COLLAB_JH_A1_01		
Occupancy ratio20160805_QE5_nLC7_MEM_COLLAB_JH_A1_01		
Occupancy error scale 20160805_QE5_nLC7_MEM_COLLAB_JH_A1_01		
Occupancy 20160805_QE5_nLC7_MEM_COLLAB_JH_A2_01		
Occupancy ratio20160805_QE5_nLC7_MEM_COLLAB_JH_A2_01		
Occupancy error scale 20160805_QE5_nLC7_MEM_COLLAB_JH_A2_01		
Occupancy 20160805_QE5_nLC7_MEM_COLLAB_JH_A3_01		
Occupancy ratio20160805_QE5_nLC7_MEM_COLLAB_JH_A3_01		
Occupancy error scale 20160805_QE5_nLC7_MEM_COLLAB_JH_A3_01		
Occupancy 20160805_QE5_nLC7_MEM_COLLAB_JH_B1_01		
Occupancy ratio20160805_QE5_nLC7_MEM_COLLAB_JH_B1_01		
Occupancy error scale 20160805_QE5_nLC7_MEM_COLLAB_JH_B1_01		

Occupancy 20160805_QE5_nLC7_MEM_COLLAB_JH_B2_01		
Occupancy ratio20160805_QE5_nLC7_MEM_COLLAB_JH_B2_01		
Occupancy error scale 20160805_QE5_nLC7_MEM_COLLAB_JH_B2_01		
Occupancy 20160805_QE5_nLC7_MEM_COLLAB_JH_B3_01		
Occupancy ratio20160805_QE5_nLC7_MEM_COLLAB_JH_B3_01		
Occupancy error scale 20160805_QE5_nLC7_MEM_COLLAB_JH_B3_01		
Occupancy 20160805_QE5_nLC7_MEM_COLLAB_JH_B4_01		
Occupancy ratio20160805_QE5_nLC7_MEM_COLLAB_JH_B4_01		
Occupancy error scale 20160805_QE5_nLC7_MEM_COLLAB_JH_B4_01		
Occupancy 20160805_QE5_nLC7_MEM_COLLAB_JH_B5_01		
Occupancy ratio20160805_QE5_nLC7_MEM_COLLAB_JH_B5_01		
Occupancy error scale 20160805_QE5_nLC7_MEM_COLLAB_JH_B5_01		
Occupancy 20160805_QE5_nLC7_MEM_COLLAB_JH_C1_01		
Occupancy ratio20160805_QE5_nLC7_MEM_COLLAB_JH_C1_01		
Occupancy error scale 20160805_QE5_nLC7_MEM_COLLAB_JH_C1_01		
Occupancy 20160805_QE5_nLC7_MEM_COLLAB_JH_C2_01		
Occupancy ratio20160805_QE5_nLC7_MEM_COLLAB_JH_C2_01		
Occupancy error scale 20160805_QE5_nLC7_MEM_COLLAB_JH_C2_01		
Occupancy 20160805_QE5_nLC7_MEM_COLLAB_JH_C3_01		
Occupancy ratio20160805_QE5_nLC7_MEM_COLLAB_JH_C3_01		
Occupancy error scale 20160805_QE5_nLC7_MEM_COLLAB_JH_C3_01		
Occupancy 20160805_QE5_nLC7_MEM_COLLAB_JH_C4_01		
Occupancy ratio20160805_QE5_nLC7_MEM_COLLAB_JH_C4_01		
Occupancy error scale 20160805_QE5_nLC7_MEM_COLLAB_JH_C4_01		
Occupancy 20160805_QE5_nLC7_MEM_COLLAB_JH_C5_01		
Occupancy ratio20160805_QE5_nLC7_MEM_COLLAB_JH_C5_01		
Occupancy error scale 20160805_QE5_nLC7_MEM_COLLAB_JH_C5_01		

Occupancy 20160805_QE5_nLC7_MEM_COLLAB_JH_C6_01		
Occupancy ratio20160805_QE5_nLC7_MEM_COLLAB_JH_C6_01		
Occupancy error scale 20160805_QE5_nLC7_MEM_COLLAB_JH_C6_01		
Occupancy 20160805_QE5_nLC7_MEM_COLLAB_JH_C7_01		
Occupancy ratio20160805_QE5_nLC7_MEM_COLLAB_JH_C7_01		
Occupancy error scale 20160805_QE5_nLC7_MEM_COLLAB_JH_C7_01		
Occupancy 20160805_QE5_nLC7_MEM_COLLAB_JH_C8_01		
Occupancy ratio20160805_QE5_nLC7_MEM_COLLAB_JH_C8_01		
Occupancy error scale 20160805_QE5_nLC7_MEM_COLLAB_JH_C8_01		
Occupancy FL0145_JHendy_130416_MSQ525_CW10		
Occupancy ratioFL0145_JHendy_130416_MSQ525_CW10		
Occupancy error scale FL0145_JHendy_130416_MSQ525_CW10		
Occupancy FL0145_JHendy_130416_MSQ525_CW11		
Occupancy ratioFL0145_JHendy_130416_MSQ525_CW11		
Occupancy error scale FL0145_JHendy_130416_MSQ525_CW11		
Occupancy FL0145_JHendy_130416_MSQ525_CW18		
Occupancy ratioFL0145_JHendy_130416_MSQ525_CW18		
Occupancy error scale FL0145_JHendy_130416_MSQ525_CW18		
Occupancy FL0145_JHendy_130416_MSQ525_CW20		
Occupancy ratioFL0145_JHendy_130416_MSQ525_CW20		
Occupancy error scale FL0145_JHendy_130416_MSQ525_CW20		
Occupancy FL0145_JHendy_130416_MSQ525_CW21		
Occupancy ratioFL0145_JHendy_130416_MSQ525_CW21		
Occupancy error scale FL0145_JHendy_130416_MSQ525_CW21		
Occupancy FL0145_JHendy_130416_MSQ525_CW23		
Occupancy ratioFL0145_JHendy_130416_MSQ525_CW23		
Occupancy error scale FL0145_JHendy_130416_MSQ525_CW23		

Occupancy FL0145_JHendy_130416_MSQ525_CW8_160425165036		
Occupancy ratioFL0145_JHendy_130416_MSQ525_CW8_160425165036		
Occupancy error scale FL0145_JHendy_130416_MSQ525_CW8_160425165036		
Occupancy FL0168_JHendy_MSQ525_170516_BK		
Occupancy ratioFL0168_JHendy_MSQ525_170516_BK		
Occupancy error scale FL0168_JHendy_MSQ525_170516_BK		
Occupancy FL0168_JHendy_MSQ525_170516_CW1		
Occupancy ratioFL0168_JHendy_MSQ525_170516_CW1		
Occupancy error scale FL0168_JHendy_MSQ525_170516_CW1		
Occupancy FL0168_JHendy_MSQ525_170516_CW10		
Occupancy ratioFL0168_JHendy_MSQ525_170516_CW10		
Occupancy error scale FL0168_JHendy_MSQ525_170516_CW10		
Occupancy FL0168_JHendy_MSQ525_170516_CW2		
Occupancy ratioFL0168_JHendy_MSQ525_170516_CW2		
Occupancy error scale FL0168_JHendy_MSQ525_170516_CW2		
Occupancy FL0168_JHendy_MSQ525_170516_CW3		
Occupancy ratioFL0168_JHendy_MSQ525_170516_CW3		
Occupancy error scale FL0168_JHendy_MSQ525_170516_CW3		
Occupancy FL0168_JHendy_MSQ525_170516_CW4		
Occupancy ratioFL0168_JHendy_MSQ525_170516_CW4		
Occupancy error scale FL0168_JHendy_MSQ525_170516_CW4		
Occupancy FL0168_JHendy_MSQ525_170516_CW5		
Occupancy ratioFL0168_JHendy_MSQ525_170516_CW5		
Occupancy error scale FL0168_JHendy_MSQ525_170516_CW5		
Occupancy FL0168_JHendy_MSQ525_170516_CW6		
Occupancy ratioFL0168_JHendy_MSQ525_170516_CW6		
Occupancy error scale FL0168_JHendy_MSQ525_170516_CW6		

Occupancy FL0168_JHendy_MSQ525_170516 _CW7		
Occupancy ratioFL0168_JHendy_MSQ525_170 516_CW7		
Occupancy error scale FL0168_JHendy_MSQ525_170516 _CW7		
Occupancy FL0168_JHendy_MSQ525_170516 _CW8		
Occupancy ratioFL0168_JHendy_MSQ525_170 516_CW8		
Occupancy error scale FL0168_JHendy_MSQ525_170516 _CW8		
Occupancy FL0168_JHendy_MSQ525_170516 _CW9		
Occupancy ratioFL0168_JHendy_MSQ525_170 516_CW9		
Occupancy error scale FL0168_JHendy_MSQ525_170516 _CW9		
Reverse		When marked with '+', this particular peptide was found to be part of a protein derived from the reversed part of the protein sequence database. These should be removed for further data analysis.
Potential contaminant		When marked with '+', this particular peptide was found to be part of a commonly occurring contaminant. These should be removed for further data analysis.
id		A unique (consecutive) identifier for each row in the site table, which is used to cross-link the information in this file with the information stored in the other files.
Protein group IDs		The identifier of the protein-group this peptide sequence is associated with, which can be used to look up the extended protein information in the file 'proteinGroups.txt'.<br>As a single peptide can be linked to multiple proteins (e.g. in the case of razor-proteins), multiple id’s can be stored here separated by a semicolon.<br>As a protein can be identified by multiple peptides, the same id can be found in different rows.
Positions		The positions of the modifications in the protein amino acid sequence.
Position		The position of the modification in the protein amino acid sequence.
Peptide IDs		Identifier(s) of the associated peptide sequence(s) summary, which can be found in the file 'peptides.txt'.
Mod. peptide IDs		Identifier(s) of the associated peptide sequence(s) summary, which can be found in the file 'modificationSpecificPeptides.txt'.
Evidence IDs		Identifier(s) for analyzed peptide evidence associated with the protein group referenced against the evidence table.
MS/MS IDs		The identifiers of the MS/MS scans identifying this peptide, referenced against the msms table.
Best localization evidence ID		
Best localization MS/MS ID		
Best localization raw file		
Best localization scan number		
Best score evidence ID		
Best score MS/MS ID		
Best score raw file		
Best score scan number		
Best PEP evidence ID		
Best PEP MS/MS ID		
Best PEP raw file		
Best PEP scan number		

HydroxyprolineSites

Name	Separator	Description
Proteins		Identifiers of proteins this site is associated with.
Positions within proteins		For each protein identifier in the 'Proteins' column you find here the position of the site in the respective protein sequence. The index of the first amino acid in the sequence is 1.
Leading proteins		
Protein		Identifier of the protein this peptide is associated with.
Fasta headers		Descriptions of proteins this peptide is associated with.
Localization prob		
Score diff		
PEP		The posterior error probability (PEP) of the best identified modified peptide containing this site.
Score		The Andromeda score of the best identified modified peptide containing this site.
Delta score		The Andromeda delta score of the best identified modified peptide containing this site.
Score for localization		The Andromeda score of the MS/MS spectrum used for calculating the localization score for this site.
Localization prob 20160805_QE5_nLC7_MEM_COLLAB_JH_A1_01		
Score diff 20160805_QE5_nLC7_MEM_COLLAB_JH_A1_01		
PEP 20160805_QE5_nLC7_MEM_COLLAB_JH_A1_01		
Score 20160805_QE5_nLC7_MEM_COLLAB_JH_A1_01		
Localization prob 20160805_QE5_nLC7_MEM_COLLAB_JH_A2_01		
Score diff 20160805_QE5_nLC7_MEM_COLLAB_JH_A2_01		
PEP 20160805_QE5_nLC7_MEM_COLLAB_JH_A2_01		
Score 20160805_QE5_nLC7_MEM_COLLAB_JH_A2_01		
Localization prob 20160805_QE5_nLC7_MEM_COLLAB_JH_A3_01		
Score diff 20160805_QE5_nLC7_MEM_COLLAB_JH_A3_01		
PEP 20160805_QE5_nLC7_MEM_COLLAB_JH_A3_01		
Score 20160805_QE5_nLC7_MEM_COLLAB_JH_A3_01		
Localization prob 20160805_QE5_nLC7_MEM_COLLAB_JH_B1_01		
Score diff 20160805_QE5_nLC7_MEM_COLLAB_JH_B1_01		
PEP 20160805_QE5_nLC7_MEM_COLLAB_JH_B1_01		
Score 20160805_QE5_nLC7_MEM_COLLAB_JH_B1_01		
Localization prob 20160805_QE5_nLC7_MEM_COLLAB_JH_B2_01		
Score diff 20160805_QE5_nLC7_MEM_COLLAB_JH_B2_01		

PEP 20160805_QE5_nLC7_MEM_COLLAB_JH_B2_01		
Score 20160805_QE5_nLC7_MEM_COLLAB_JH_B2_01		
Localization prob 20160805_QE5_nLC7_MEM_COLLAB_JH_B3_01		
Score diff 20160805_QE5_nLC7_MEM_COLLAB_JH_B3_01		
PEP 20160805_QE5_nLC7_MEM_COLLAB_JH_B3_01		
Score 20160805_QE5_nLC7_MEM_COLLAB_JH_B3_01		
Localization prob 20160805_QE5_nLC7_MEM_COLLAB_JH_B4_01		
Score diff 20160805_QE5_nLC7_MEM_COLLAB_JH_B4_01		
PEP 20160805_QE5_nLC7_MEM_COLLAB_JH_B4_01		
Score 20160805_QE5_nLC7_MEM_COLLAB_JH_B4_01		
Localization prob 20160805_QE5_nLC7_MEM_COLLAB_JH_B5_01		
Score diff 20160805_QE5_nLC7_MEM_COLLAB_JH_B5_01		
PEP 20160805_QE5_nLC7_MEM_COLLAB_JH_B5_01		
Score 20160805_QE5_nLC7_MEM_COLLAB_JH_B5_01		
Localization prob 20160805_QE5_nLC7_MEM_COLLAB_JH_C1_01		
Score diff 20160805_QE5_nLC7_MEM_COLLAB_JH_C1_01		
PEP 20160805_QE5_nLC7_MEM_COLLAB_JH_C1_01		
Score 20160805_QE5_nLC7_MEM_COLLAB_JH_C1_01		
Localization prob 20160805_QE5_nLC7_MEM_COLLAB_JH_C2_01		
Score diff 20160805_QE5_nLC7_MEM_COLLAB_JH_C2_01		
PEP 20160805_QE5_nLC7_MEM_COLLAB_JH_C2_01		
Score 20160805_QE5_nLC7_MEM_COLLAB_JH_C2_01		
Localization prob 20160805_QE5_nLC7_MEM_COLLAB_JH_C3_01		
Score diff 20160805_QE5_nLC7_MEM_COLLAB_JH_C3_01		
PEP 20160805_QE5_nLC7_MEM_COLLAB_JH_C3_01		
Score 20160805_QE5_nLC7_MEM_COLLAB_JH_C3_01		
Localization prob 20160805_QE5_nLC7_MEM_COLLAB_JH_C4_01		

Score diff 20160805_QE5_nLC7_MEM_COLLAB_JH_C4_01		
PEP 20160805_QE5_nLC7_MEM_COLLAB_JH_C4_01		
Score 20160805_QE5_nLC7_MEM_COLLAB_JH_C4_01		
Localization prob 20160805_QE5_nLC7_MEM_COLLAB_JH_C5_01		
Score diff 20160805_QE5_nLC7_MEM_COLLAB_JH_C5_01		
PEP 20160805_QE5_nLC7_MEM_COLLAB_JH_C5_01		
Score 20160805_QE5_nLC7_MEM_COLLAB_JH_C5_01		
Localization prob 20160805_QE5_nLC7_MEM_COLLAB_JH_C6_01		
Score diff 20160805_QE5_nLC7_MEM_COLLAB_JH_C6_01		
PEP 20160805_QE5_nLC7_MEM_COLLAB_JH_C6_01		
Score 20160805_QE5_nLC7_MEM_COLLAB_JH_C6_01		
Localization prob 20160805_QE5_nLC7_MEM_COLLAB_JH_C7_01		
Score diff 20160805_QE5_nLC7_MEM_COLLAB_JH_C7_01		
PEP 20160805_QE5_nLC7_MEM_COLLAB_JH_C7_01		
Score 20160805_QE5_nLC7_MEM_COLLAB_JH_C7_01		
Localization prob 20160805_QE5_nLC7_MEM_COLLAB_JH_C8_01		
Score diff 20160805_QE5_nLC7_MEM_COLLAB_JH_C8_01		
PEP 20160805_QE5_nLC7_MEM_COLLAB_JH_C8_01		
Score 20160805_QE5_nLC7_MEM_COLLAB_JH_C8_01		
Localization prob FL0145_JHendy_130416_MSQ525_CW10		
Score diff FL0145_JHendy_130416_MSQ525_CW10		
PEP FL0145_JHendy_130416_MSQ525_CW10		
Score FL0145_JHendy_130416_MSQ525_CW10		
Localization prob FL0145_JHendy_130416_MSQ525_CW11		
Score diff FL0145_JHendy_130416_MSQ525_CW11		
PEP FL0145_JHendy_130416_MSQ525_CW11		
Score FL0145_JHendy_130416_MSQ525_CW11		

Localization prob FL0145_JHendy_130416_MSQ525_CW18		
Score diff FL0145_JHendy_130416_MSQ525_CW18		
PEP FL0145_JHendy_130416_MSQ525_CW18		
Score FL0145_JHendy_130416_MSQ525_CW18		
Localization prob FL0145_JHendy_130416_MSQ525_CW20		
Score diff FL0145_JHendy_130416_MSQ525_CW20		
PEP FL0145_JHendy_130416_MSQ525_CW20		
Score FL0145_JHendy_130416_MSQ525_CW20		
Localization prob FL0145_JHendy_130416_MSQ525_CW21		
Score diff FL0145_JHendy_130416_MSQ525_CW21		
PEP FL0145_JHendy_130416_MSQ525_CW21		
Score FL0145_JHendy_130416_MSQ525_CW21		
Localization prob FL0145_JHendy_130416_MSQ525_CW23		
Score diff FL0145_JHendy_130416_MSQ525_CW23		
PEP FL0145_JHendy_130416_MSQ525_CW23		
Score FL0145_JHendy_130416_MSQ525_CW23		
Localization prob FL0145_JHendy_130416_MSQ525_CW8_160425165036		
Score diff FL0145_JHendy_130416_MSQ525_CW8_160425165036		
PEP FL0145_JHendy_130416_MSQ525_CW8_160425165036		
Score FL0145_JHendy_130416_MSQ525_CW8_160425165036		
Localization prob FL0168_JHendy_MSQ525_170516_BK		
Score diff FL0168_JHendy_MSQ525_170516_BK		
PEP FL0168_JHendy_MSQ525_170516_BK		
Score FL0168_JHendy_MSQ525_170516_BK		
Localization prob FL0168_JHendy_MSQ525_170516_CW1		
Score diff FL0168_JHendy_MSQ525_170516_CW1		
PEP FL0168_JHendy_MSQ525_170516_CW1		

Score FL0168_JHendy_MSQ525_170516_CW1		
Localization prob FL0168_JHendy_MSQ525_170516_CW10		
Score diff FL0168_JHendy_MSQ525_170516_CW10		
PEP FL0168_JHendy_MSQ525_170516_CW10		
Score FL0168_JHendy_MSQ525_170516_CW10		
Localization prob FL0168_JHendy_MSQ525_170516_CW2		
Score diff FL0168_JHendy_MSQ525_170516_CW2		
PEP FL0168_JHendy_MSQ525_170516_CW2		
Score FL0168_JHendy_MSQ525_170516_CW2		
Localization prob FL0168_JHendy_MSQ525_170516_CW3		
Score diff FL0168_JHendy_MSQ525_170516_CW3		
PEP FL0168_JHendy_MSQ525_170516_CW3		
Score FL0168_JHendy_MSQ525_170516_CW3		
Localization prob FL0168_JHendy_MSQ525_170516_CW4		
Score diff FL0168_JHendy_MSQ525_170516_CW4		
PEP FL0168_JHendy_MSQ525_170516_CW4		
Score FL0168_JHendy_MSQ525_170516_CW4		
Localization prob FL0168_JHendy_MSQ525_170516_CW5		
Score diff FL0168_JHendy_MSQ525_170516_CW5		
PEP FL0168_JHendy_MSQ525_170516_CW5		
Score FL0168_JHendy_MSQ525_170516_CW5		
Localization prob FL0168_JHendy_MSQ525_170516_CW6		
Score diff FL0168_JHendy_MSQ525_170516_CW6		
PEP FL0168_JHendy_MSQ525_170516_CW6		
Score FL0168_JHendy_MSQ525_170516_CW6		
Localization prob FL0168_JHendy_MSQ525_170516_CW7		
Score diff FL0168_JHendy_MSQ525_170516_CW7		

PEP FL0168_JHendy_MSQ525_170516_CW7		
Score FL0168_JHendy_MSQ525_170516_CW7		
Localization prob FL0168_JHendy_MSQ525_170516_CW8		
Score diff FL0168_JHendy_MSQ525_170516_CW8		
PEP FL0168_JHendy_MSQ525_170516_CW8		
Score FL0168_JHendy_MSQ525_170516_CW8		
Localization prob FL0168_JHendy_MSQ525_170516_CW9		
Score diff FL0168_JHendy_MSQ525_170516_CW9		
PEP FL0168_JHendy_MSQ525_170516_CW9		
Score FL0168_JHendy_MSQ525_170516_CW9		
Diagnostic peak		
Number of Hydroxyproline		Different numbers of Hydroxyproline on peptides that this site is involved in.
Amino acid		
Sequence window		
Modification window		
Peptide window coverage		
Hydroxyproline Probabilities		
Hydroxyproline Score diffs		
Position in peptide		
Charge		Charge state of the precursor ion.
Mass error [ppm]		Mass error of the recalibrated mass-over-charge value of the precursor ion in comparison to the predicted monoisotopic mass of the identified peptide sequence.
Intensity		Summed up eXtracted Ion Current (XIC) of all isotopic clusters associated with the identified AA sequence. In case of a labeled experiment this is the total intensity of all the isotopic patterns in the label cluster.
Intensity___1		Summed up eXtracted Ion Current (XIC) of all isotopic clusters associated with the identified AA sequence. In case of a labeled experiment this is the total intensity of all the isotopic patterns in the label cluster.
Intensity___2		Summed up eXtracted Ion Current (XIC) of all isotopic clusters associated with the identified AA sequence. In case of a labeled experiment this is the total intensity of all the isotopic patterns in the label cluster.
Intensity___3		Summed up eXtracted Ion Current (XIC) of all isotopic clusters associated with the identified AA sequence. In case of a labeled experiment this is the total intensity of all the isotopic patterns in the label cluster.
Ratio mod/base		
Intensity 20160805_QE5_nLC7_MEM_COLLAB_JH_A1_01		Summed up eXtracted Ion Current (XIC) of all isotopic clusters associated with the identified AA sequence. In case of a labeled experiment this is the total intensity of all the isotopic patterns in the label cluster.
Intensity 20160805_QE5_nLC7_MEM_COLLAB_JH_A2_01		Summed up eXtracted Ion Current (XIC) of all isotopic clusters associated with the identified AA sequence. In case of a labeled experiment this is the total intensity of all the isotopic patterns in the label cluster.
Intensity 20160805_QE5_nLC7_MEM_COLLAB_JH_A3_01		Summed up eXtracted Ion Current (XIC) of all isotopic clusters associated with the identified AA sequence. In case of a labeled experiment this is the total intensity of all the isotopic patterns in the label cluster.
Intensity 20160805_QE5_nLC7_MEM_COLLAB_JH_B1_01		Summed up eXtracted Ion Current (XIC) of all isotopic clusters associated with the identified AA sequence. In case of a labeled experiment this is the total intensity of all the isotopic patterns in the label cluster.

Intensity 20160805_QE5_nLC7_MEM_COLLAB_JH_B2_01		Summed up eXtracted Ion Current (XIC) of all isotopic clusters associated with the identified AA sequence. In case of a labeled experiment this is the total intensity of all the isotopic patterns in the label cluster.
Intensity 20160805_QE5_nLC7_MEM_COLLAB_JH_B3_01		Summed up eXtracted Ion Current (XIC) of all isotopic clusters associated with the identified AA sequence. In case of a labeled experiment this is the total intensity of all the isotopic patterns in the label cluster.
Intensity 20160805_QE5_nLC7_MEM_COLLAB_JH_B4_01		Summed up eXtracted Ion Current (XIC) of all isotopic clusters associated with the identified AA sequence. In case of a labeled experiment this is the total intensity of all the isotopic patterns in the label cluster.
Intensity 20160805_QE5_nLC7_MEM_COLLAB_JH_B5_01		Summed up eXtracted Ion Current (XIC) of all isotopic clusters associated with the identified AA sequence. In case of a labeled experiment this is the total intensity of all the isotopic patterns in the label cluster.
Intensity 20160805_QE5_nLC7_MEM_COLLAB_JH_C1_01		Summed up eXtracted Ion Current (XIC) of all isotopic clusters associated with the identified AA sequence. In case of a labeled experiment this is the total intensity of all the isotopic patterns in the label cluster.
Intensity 20160805_QE5_nLC7_MEM_COLLAB_JH_C2_01		Summed up eXtracted Ion Current (XIC) of all isotopic clusters associated with the identified AA sequence. In case of a labeled experiment this is the total intensity of all the isotopic patterns in the label cluster.
Intensity 20160805_QE5_nLC7_MEM_COLLAB_JH_C3_01		Summed up eXtracted Ion Current (XIC) of all isotopic clusters associated with the identified AA sequence. In case of a labeled experiment this is the total intensity of all the isotopic patterns in the label cluster.
Intensity 20160805_QE5_nLC7_MEM_COLLAB_JH_C4_01		Summed up eXtracted Ion Current (XIC) of all isotopic clusters associated with the identified AA sequence. In case of a labeled experiment this is the total intensity of all the isotopic patterns in the label cluster.
Intensity 20160805_QE5_nLC7_MEM_COLLAB_JH_C5_01		Summed up eXtracted Ion Current (XIC) of all isotopic clusters associated with the identified AA sequence. In case of a labeled experiment this is the total intensity of all the isotopic patterns in the label cluster.
Intensity 20160805_QE5_nLC7_MEM_COLLAB_JH_C6_01		Summed up eXtracted Ion Current (XIC) of all isotopic clusters associated with the identified AA sequence. In case of a labeled experiment this is the total intensity of all the isotopic patterns in the label cluster.
Intensity 20160805_QE5_nLC7_MEM_COLLAB_JH_C7_01		Summed up eXtracted Ion Current (XIC) of all isotopic clusters associated with the identified AA sequence. In case of a labeled experiment this is the total intensity of all the isotopic patterns in the label cluster.
Intensity 20160805_QE5_nLC7_MEM_COLLAB_JH_C8_01		Summed up eXtracted Ion Current (XIC) of all isotopic clusters associated with the identified AA sequence. In case of a labeled experiment this is the total intensity of all the isotopic patterns in the label cluster.
Intensity FL0145_JHendy_130416_MSQ525_CW10		Summed up eXtracted Ion Current (XIC) of all isotopic clusters associated with the identified AA sequence. In case of a labeled experiment this is the total intensity of all the isotopic patterns in the label cluster.
Intensity FL0145_JHendy_130416_MSQ525_CW11		Summed up eXtracted Ion Current (XIC) of all isotopic clusters associated with the identified AA sequence. In case of a labeled experiment this is the total intensity of all the isotopic patterns in the label cluster.
Intensity FL0145_JHendy_130416_MSQ525_CW18		Summed up eXtracted Ion Current (XIC) of all isotopic clusters associated with the identified AA sequence. In case of a labeled experiment this is the total intensity of all the isotopic patterns in the label cluster.
Intensity FL0145_JHendy_130416_MSQ525_CW20		Summed up eXtracted Ion Current (XIC) of all isotopic clusters associated with the identified AA sequence. In case of a labeled experiment this is the total intensity of all the isotopic patterns in the label cluster.
Intensity FL0145_JHendy_130416_MSQ525_CW21		Summed up eXtracted Ion Current (XIC) of all isotopic clusters associated with the identified AA sequence. In case of a labeled experiment this is the total intensity of all the isotopic patterns in the label cluster.
Intensity FL0145_JHendy_130416_MSQ525_CW23		Summed up eXtracted Ion Current (XIC) of all isotopic clusters associated with the identified AA sequence. In case of a labeled experiment this is the total intensity of all the isotopic patterns in the label cluster.
Intensity FL0145_JHendy_130416_MSQ525_CW8_160425165036		Summed up eXtracted Ion Current (XIC) of all isotopic clusters associated with the identified AA sequence. In case of a labeled experiment this is the total intensity of all the isotopic patterns in the label cluster.
Intensity FL0168_JHendy_MSQ525_170516_BK		Summed up eXtracted Ion Current (XIC) of all isotopic clusters associated with the identified AA sequence. In case of a labeled experiment this is the total intensity of all the isotopic patterns in the label cluster.
Intensity FL0168_JHendy_MSQ525_170516_CW1		Summed up eXtracted Ion Current (XIC) of all isotopic clusters associated with the identified AA sequence. In case of a labeled experiment this is the total intensity of all the isotopic patterns in the label cluster.

Intensity FL0168_JHendy_MSQ525_170516_CW10		Summed up eXtracted Ion Current (XIC) of all isotopic clusters associated with the identified AA sequence. In case of a labeled experiment this is the total intensity of all the isotopic patterns in the label cluster.
Intensity FL0168_JHendy_MSQ525_170516_CW2		Summed up eXtracted Ion Current (XIC) of all isotopic clusters associated with the identified AA sequence. In case of a labeled experiment this is the total intensity of all the isotopic patterns in the label cluster.
Intensity FL0168_JHendy_MSQ525_170516_CW3		Summed up eXtracted Ion Current (XIC) of all isotopic clusters associated with the identified AA sequence. In case of a labeled experiment this is the total intensity of all the isotopic patterns in the label cluster.
Intensity FL0168_JHendy_MSQ525_170516_CW4		Summed up eXtracted Ion Current (XIC) of all isotopic clusters associated with the identified AA sequence. In case of a labeled experiment this is the total intensity of all the isotopic patterns in the label cluster.
Intensity FL0168_JHendy_MSQ525_170516_CW5		Summed up eXtracted Ion Current (XIC) of all isotopic clusters associated with the identified AA sequence. In case of a labeled experiment this is the total intensity of all the isotopic patterns in the label cluster.
Intensity FL0168_JHendy_MSQ525_170516_CW6		Summed up eXtracted Ion Current (XIC) of all isotopic clusters associated with the identified AA sequence. In case of a labeled experiment this is the total intensity of all the isotopic patterns in the label cluster.
Intensity FL0168_JHendy_MSQ525_170516_CW7		Summed up eXtracted Ion Current (XIC) of all isotopic clusters associated with the identified AA sequence. In case of a labeled experiment this is the total intensity of all the isotopic patterns in the label cluster.
Intensity FL0168_JHendy_MSQ525_170516_CW8		Summed up eXtracted Ion Current (XIC) of all isotopic clusters associated with the identified AA sequence. In case of a labeled experiment this is the total intensity of all the isotopic patterns in the label cluster.
Intensity FL0168_JHendy_MSQ525_170516_CW9		Summed up eXtracted Ion Current (XIC) of all isotopic clusters associated with the identified AA sequence. In case of a labeled experiment this is the total intensity of all the isotopic patterns in the label cluster.
Ratio mod/base 20160805_QE5_nLC7_MEM_COLLAB_JH_A1_01		
Ratio mod/base 20160805_QE5_nLC7_MEM_COLLAB_JH_A2_01		
Ratio mod/base 20160805_QE5_nLC7_MEM_COLLAB_JH_A3_01		
Ratio mod/base 20160805_QE5_nLC7_MEM_COLLAB_JH_B1_01		
Ratio mod/base 20160805_QE5_nLC7_MEM_COLLAB_JH_B2_01		
Ratio mod/base 20160805_QE5_nLC7_MEM_COLLAB_JH_B3_01		
Ratio mod/base 20160805_QE5_nLC7_MEM_COLLAB_JH_B4_01		
Ratio mod/base 20160805_QE5_nLC7_MEM_COLLAB_JH_B5_01		
Ratio mod/base 20160805_QE5_nLC7_MEM_COLLAB_JH_C1_01		
Ratio mod/base 20160805_QE5_nLC7_MEM_COLLAB_JH_C2_01		
Ratio mod/base 20160805_QE5_nLC7_MEM_COLLAB_JH_C3_01		
Ratio mod/base 20160805_QE5_nLC7_MEM_COLLAB_JH_C4_01		
Ratio mod/base 20160805_QE5_nLC7_MEM_COLLAB_JH_C5_01		
Ratio mod/base 20160805_QE5_nLC7_MEM_COLLAB_JH_C6_01		
Ratio mod/base 20160805_QE5_nLC7_MEM_COLLAB_JH_C7_01		

Ratio mod/base 20160805_QE5_nLC7_MEM_COLLAB_JH_C8_01		
Ratio mod/base FL0145_JHendy_130416_MSQ525_CW10		
Ratio mod/base FL0145_JHendy_130416_MSQ525_CW11		
Ratio mod/base FL0145_JHendy_130416_MSQ525_CW18		
Ratio mod/base FL0145_JHendy_130416_MSQ525_CW20		
Ratio mod/base FL0145_JHendy_130416_MSQ525_CW21		
Ratio mod/base FL0145_JHendy_130416_MSQ525_CW23		
Ratio mod/base FL0145_JHendy_130416_MSQ525_CW8_160425165036		
Ratio mod/base FL0168_JHendy_MSQ525_170516_BK		
Ratio mod/base FL0168_JHendy_MSQ525_170516_CW1		
Ratio mod/base FL0168_JHendy_MSQ525_170516_CW10		
Ratio mod/base FL0168_JHendy_MSQ525_170516_CW2		
Ratio mod/base FL0168_JHendy_MSQ525_170516_CW3		
Ratio mod/base FL0168_JHendy_MSQ525_170516_CW4		
Ratio mod/base FL0168_JHendy_MSQ525_170516_CW5		
Ratio mod/base FL0168_JHendy_MSQ525_170516_CW6		
Ratio mod/base FL0168_JHendy_MSQ525_170516_CW7		
Ratio mod/base FL0168_JHendy_MSQ525_170516_CW8		
Ratio mod/base FL0168_JHendy_MSQ525_170516_CW9		
Intensity 20160805_QE5_nLC7_MEM_COLLAB_JH_A1_01___1		Summed up eXtracted Ion Current (XIC) of all isotopic clusters associated with the identified AA sequence. In case of a labeled experiment this is the total intensity of all the isotopic patterns in the label cluster.
Intensity 20160805_QE5_nLC7_MEM_COLLAB_JH_A1_01___2		Summed up eXtracted Ion Current (XIC) of all isotopic clusters associated with the identified AA sequence. In case of a labeled experiment this is the total intensity of all the isotopic patterns in the label cluster.
Intensity 20160805_QE5_nLC7_MEM_COLLAB_JH_A1_01___3		Summed up eXtracted Ion Current (XIC) of all isotopic clusters associated with the identified AA sequence. In case of a labeled experiment this is the total intensity of all the isotopic patterns in the label cluster.
Intensity 20160805_QE5_nLC7_MEM_COLLAB_JH_A2_01___1		Summed up eXtracted Ion Current (XIC) of all isotopic clusters associated with the identified AA sequence. In case of a labeled experiment this is the total intensity of all the isotopic patterns in the label cluster.
Intensity 20160805_QE5_nLC7_MEM_COLLAB_JH_A2_01___2		Summed up eXtracted Ion Current (XIC) of all isotopic clusters associated with the identified AA sequence. In case of a labeled experiment this is the total intensity of all the isotopic patterns in the label cluster.
Intensity 20160805_QE5_nLC7_MEM_COLLAB_JH_A2_01___3		Summed up eXtracted Ion Current (XIC) of all isotopic clusters associated with the identified AA sequence. In case of a labeled experiment this is the total intensity of all the isotopic patterns in the label cluster.

Intensity 20160805_QE5_nLC7_MEM_COLLAB_JH_A3_01___1		Summed up eXtracted Ion Current (XIC) of all isotopic clusters associated with the identified AA sequence. In case of a labeled experiment this is the total intensity of all the isotopic patterns in the label cluster.
Intensity 20160805_QE5_nLC7_MEM_COLLAB_JH_A3_01___2		Summed up eXtracted Ion Current (XIC) of all isotopic clusters associated with the identified AA sequence. In case of a labeled experiment this is the total intensity of all the isotopic patterns in the label cluster.
Intensity 20160805_QE5_nLC7_MEM_COLLAB_JH_A3_01___3		Summed up eXtracted Ion Current (XIC) of all isotopic clusters associated with the identified AA sequence. In case of a labeled experiment this is the total intensity of all the isotopic patterns in the label cluster.
Intensity 20160805_QE5_nLC7_MEM_COLLAB_JH_B1_01___1		Summed up eXtracted Ion Current (XIC) of all isotopic clusters associated with the identified AA sequence. In case of a labeled experiment this is the total intensity of all the isotopic patterns in the label cluster.
Intensity 20160805_QE5_nLC7_MEM_COLLAB_JH_B1_01___2		Summed up eXtracted Ion Current (XIC) of all isotopic clusters associated with the identified AA sequence. In case of a labeled experiment this is the total intensity of all the isotopic patterns in the label cluster.
Intensity 20160805_QE5_nLC7_MEM_COLLAB_JH_B1_01___3		Summed up eXtracted Ion Current (XIC) of all isotopic clusters associated with the identified AA sequence. In case of a labeled experiment this is the total intensity of all the isotopic patterns in the label cluster.
Intensity 20160805_QE5_nLC7_MEM_COLLAB_JH_B2_01___1		Summed up eXtracted Ion Current (XIC) of all isotopic clusters associated with the identified AA sequence. In case of a labeled experiment this is the total intensity of all the isotopic patterns in the label cluster.
Intensity 20160805_QE5_nLC7_MEM_COLLAB_JH_B2_01___2		Summed up eXtracted Ion Current (XIC) of all isotopic clusters associated with the identified AA sequence. In case of a labeled experiment this is the total intensity of all the isotopic patterns in the label cluster.
Intensity 20160805_QE5_nLC7_MEM_COLLAB_JH_B2_01___3		Summed up eXtracted Ion Current (XIC) of all isotopic clusters associated with the identified AA sequence. In case of a labeled experiment this is the total intensity of all the isotopic patterns in the label cluster.
Intensity 20160805_QE5_nLC7_MEM_COLLAB_JH_B3_01___1		Summed up eXtracted Ion Current (XIC) of all isotopic clusters associated with the identified AA sequence. In case of a labeled experiment this is the total intensity of all the isotopic patterns in the label cluster.
Intensity 20160805_QE5_nLC7_MEM_COLLAB_JH_B3_01___2		Summed up eXtracted Ion Current (XIC) of all isotopic clusters associated with the identified AA sequence. In case of a labeled experiment this is the total intensity of all the isotopic patterns in the label cluster.
Intensity 20160805_QE5_nLC7_MEM_COLLAB_JH_B3_01___3		Summed up eXtracted Ion Current (XIC) of all isotopic clusters associated with the identified AA sequence. In case of a labeled experiment this is the total intensity of all the isotopic patterns in the label cluster.
Intensity 20160805_QE5_nLC7_MEM_COLLAB_JH_B4_01___1		Summed up eXtracted Ion Current (XIC) of all isotopic clusters associated with the identified AA sequence. In case of a labeled experiment this is the total intensity of all the isotopic patterns in the label cluster.
Intensity 20160805_QE5_nLC7_MEM_COLLAB_JH_B4_01___2		Summed up eXtracted Ion Current (XIC) of all isotopic clusters associated with the identified AA sequence. In case of a labeled experiment this is the total intensity of all the isotopic patterns in the label cluster.
Intensity 20160805_QE5_nLC7_MEM_COLLAB_JH_B4_01___3		Summed up eXtracted Ion Current (XIC) of all isotopic clusters associated with the identified AA sequence. In case of a labeled experiment this is the total intensity of all the isotopic patterns in the label cluster.
Intensity 20160805_QE5_nLC7_MEM_COLLAB_JH_B5_01___1		Summed up eXtracted Ion Current (XIC) of all isotopic clusters associated with the identified AA sequence. In case of a labeled experiment this is the total intensity of all the isotopic patterns in the label cluster.
Intensity 20160805_QE5_nLC7_MEM_COLLAB_JH_B5_01___2		Summed up eXtracted Ion Current (XIC) of all isotopic clusters associated with the identified AA sequence. In case of a labeled experiment this is the total intensity of all the isotopic patterns in the label cluster.
Intensity 20160805_QE5_nLC7_MEM_COLLAB_JH_B5_01___3		Summed up eXtracted Ion Current (XIC) of all isotopic clusters associated with the identified AA sequence. In case of a labeled experiment this is the total intensity of all the isotopic patterns in the label cluster.
Intensity 20160805_QE5_nLC7_MEM_COLLAB_JH_C1_01___1		Summed up eXtracted Ion Current (XIC) of all isotopic clusters associated with the identified AA sequence. In case of a labeled experiment this is the total intensity of all the isotopic patterns in the label cluster.
Intensity 20160805_QE5_nLC7_MEM_COLLAB_JH_C1_01___2		Summed up eXtracted Ion Current (XIC) of all isotopic clusters associated with the identified AA sequence. In case of a labeled experiment this is the total intensity of all the isotopic patterns in the label cluster.
Intensity 20160805_QE5_nLC7_MEM_COLLAB_JH_C1_01___3		Summed up eXtracted Ion Current (XIC) of all isotopic clusters associated with the identified AA sequence. In case of a labeled experiment this is the total intensity of all the isotopic patterns in the label cluster.

Intensity 20160805_QE5_nLC7_MEM_COLLAB_JH_C2_01___1		Summed up eXtracted Ion Current (XIC) of all isotopic clusters associated with the identified AA sequence. In case of a labeled experiment this is the total intensity of all the isotopic patterns in the label cluster.
Intensity 20160805_QE5_nLC7_MEM_COLLAB_JH_C2_01___2		Summed up eXtracted Ion Current (XIC) of all isotopic clusters associated with the identified AA sequence. In case of a labeled experiment this is the total intensity of all the isotopic patterns in the label cluster.
Intensity 20160805_QE5_nLC7_MEM_COLLAB_JH_C2_01___3		Summed up eXtracted Ion Current (XIC) of all isotopic clusters associated with the identified AA sequence. In case of a labeled experiment this is the total intensity of all the isotopic patterns in the label cluster.
Intensity 20160805_QE5_nLC7_MEM_COLLAB_JH_C3_01___1		Summed up eXtracted Ion Current (XIC) of all isotopic clusters associated with the identified AA sequence. In case of a labeled experiment this is the total intensity of all the isotopic patterns in the label cluster.
Intensity 20160805_QE5_nLC7_MEM_COLLAB_JH_C3_01___2		Summed up eXtracted Ion Current (XIC) of all isotopic clusters associated with the identified AA sequence. In case of a labeled experiment this is the total intensity of all the isotopic patterns in the label cluster.
Intensity 20160805_QE5_nLC7_MEM_COLLAB_JH_C3_01___3		Summed up eXtracted Ion Current (XIC) of all isotopic clusters associated with the identified AA sequence. In case of a labeled experiment this is the total intensity of all the isotopic patterns in the label cluster.
Intensity 20160805_QE5_nLC7_MEM_COLLAB_JH_C4_01___1		Summed up eXtracted Ion Current (XIC) of all isotopic clusters associated with the identified AA sequence. In case of a labeled experiment this is the total intensity of all the isotopic patterns in the label cluster.
Intensity 20160805_QE5_nLC7_MEM_COLLAB_JH_C4_01___2		Summed up eXtracted Ion Current (XIC) of all isotopic clusters associated with the identified AA sequence. In case of a labeled experiment this is the total intensity of all the isotopic patterns in the label cluster.
Intensity 20160805_QE5_nLC7_MEM_COLLAB_JH_C4_01___3		Summed up eXtracted Ion Current (XIC) of all isotopic clusters associated with the identified AA sequence. In case of a labeled experiment this is the total intensity of all the isotopic patterns in the label cluster.
Intensity 20160805_QE5_nLC7_MEM_COLLAB_JH_C5_01___1		Summed up eXtracted Ion Current (XIC) of all isotopic clusters associated with the identified AA sequence. In case of a labeled experiment this is the total intensity of all the isotopic patterns in the label cluster.
Intensity 20160805_QE5_nLC7_MEM_COLLAB_JH_C5_01___2		Summed up eXtracted Ion Current (XIC) of all isotopic clusters associated with the identified AA sequence. In case of a labeled experiment this is the total intensity of all the isotopic patterns in the label cluster.
Intensity 20160805_QE5_nLC7_MEM_COLLAB_JH_C5_01___3		Summed up eXtracted Ion Current (XIC) of all isotopic clusters associated with the identified AA sequence. In case of a labeled experiment this is the total intensity of all the isotopic patterns in the label cluster.
Intensity 20160805_QE5_nLC7_MEM_COLLAB_JH_C6_01___1		Summed up eXtracted Ion Current (XIC) of all isotopic clusters associated with the identified AA sequence. In case of a labeled experiment this is the total intensity of all the isotopic patterns in the label cluster.
Intensity 20160805_QE5_nLC7_MEM_COLLAB_JH_C6_01___2		Summed up eXtracted Ion Current (XIC) of all isotopic clusters associated with the identified AA sequence. In case of a labeled experiment this is the total intensity of all the isotopic patterns in the label cluster.
Intensity 20160805_QE5_nLC7_MEM_COLLAB_JH_C6_01___3		Summed up eXtracted Ion Current (XIC) of all isotopic clusters associated with the identified AA sequence. In case of a labeled experiment this is the total intensity of all the isotopic patterns in the label cluster.
Intensity 20160805_QE5_nLC7_MEM_COLLAB_JH_C7_01___1		Summed up eXtracted Ion Current (XIC) of all isotopic clusters associated with the identified AA sequence. In case of a labeled experiment this is the total intensity of all the isotopic patterns in the label cluster.
Intensity 20160805_QE5_nLC7_MEM_COLLAB_JH_C7_01___2		Summed up eXtracted Ion Current (XIC) of all isotopic clusters associated with the identified AA sequence. In case of a labeled experiment this is the total intensity of all the isotopic patterns in the label cluster.
Intensity 20160805_QE5_nLC7_MEM_COLLAB_JH_C7_01___3		Summed up eXtracted Ion Current (XIC) of all isotopic clusters associated with the identified AA sequence. In case of a labeled experiment this is the total intensity of all the isotopic patterns in the label cluster.
Intensity 20160805_QE5_nLC7_MEM_COLLAB_JH_C8_01___1		Summed up eXtracted Ion Current (XIC) of all isotopic clusters associated with the identified AA sequence. In case of a labeled experiment this is the total intensity of all the isotopic patterns in the label cluster.
Intensity 20160805_QE5_nLC7_MEM_COLLAB_JH_C8_01___2		Summed up eXtracted Ion Current (XIC) of all isotopic clusters associated with the identified AA sequence. In case of a labeled experiment this is the total intensity of all the isotopic patterns in the label cluster.
Intensity 20160805_QE5_nLC7_MEM_COLLAB_JH_C8_01___3		Summed up eXtracted Ion Current (XIC) of all isotopic clusters associated with the identified AA sequence. In case of a labeled experiment this is the total intensity of all the isotopic patterns in the label cluster.

Intensity FL0145_JHendy_130416_MSQ525_CW10___1		Summed up eXtracted Ion Current (XIC) of all isotopic clusters associated with the identified AA sequence. In case of a labeled experiment this is the total intensity of all the isotopic patterns in the label cluster.
Intensity FL0145_JHendy_130416_MSQ525_CW10___2		Summed up eXtracted Ion Current (XIC) of all isotopic clusters associated with the identified AA sequence. In case of a labeled experiment this is the total intensity of all the isotopic patterns in the label cluster.
Intensity FL0145_JHendy_130416_MSQ525_CW10___3		Summed up eXtracted Ion Current (XIC) of all isotopic clusters associated with the identified AA sequence. In case of a labeled experiment this is the total intensity of all the isotopic patterns in the label cluster.
Intensity FL0145_JHendy_130416_MSQ525_CW11___1		Summed up eXtracted Ion Current (XIC) of all isotopic clusters associated with the identified AA sequence. In case of a labeled experiment this is the total intensity of all the isotopic patterns in the label cluster.
Intensity FL0145_JHendy_130416_MSQ525_CW11___2		Summed up eXtracted Ion Current (XIC) of all isotopic clusters associated with the identified AA sequence. In case of a labeled experiment this is the total intensity of all the isotopic patterns in the label cluster.
Intensity FL0145_JHendy_130416_MSQ525_CW11___3		Summed up eXtracted Ion Current (XIC) of all isotopic clusters associated with the identified AA sequence. In case of a labeled experiment this is the total intensity of all the isotopic patterns in the label cluster.
Intensity FL0145_JHendy_130416_MSQ525_CW18___1		Summed up eXtracted Ion Current (XIC) of all isotopic clusters associated with the identified AA sequence. In case of a labeled experiment this is the total intensity of all the isotopic patterns in the label cluster.
Intensity FL0145_JHendy_130416_MSQ525_CW18___2		Summed up eXtracted Ion Current (XIC) of all isotopic clusters associated with the identified AA sequence. In case of a labeled experiment this is the total intensity of all the isotopic patterns in the label cluster.
Intensity FL0145_JHendy_130416_MSQ525_CW18___3		Summed up eXtracted Ion Current (XIC) of all isotopic clusters associated with the identified AA sequence. In case of a labeled experiment this is the total intensity of all the isotopic patterns in the label cluster.
Intensity FL0145_JHendy_130416_MSQ525_CW20___1		Summed up eXtracted Ion Current (XIC) of all isotopic clusters associated with the identified AA sequence. In case of a labeled experiment this is the total intensity of all the isotopic patterns in the label cluster.
Intensity FL0145_JHendy_130416_MSQ525_CW20___2		Summed up eXtracted Ion Current (XIC) of all isotopic clusters associated with the identified AA sequence. In case of a labeled experiment this is the total intensity of all the isotopic patterns in the label cluster.
Intensity FL0145_JHendy_130416_MSQ525_CW20___3		Summed up eXtracted Ion Current (XIC) of all isotopic clusters associated with the identified AA sequence. In case of a labeled experiment this is the total intensity of all the isotopic patterns in the label cluster.
Intensity FL0145_JHendy_130416_MSQ525_CW21___1		Summed up eXtracted Ion Current (XIC) of all isotopic clusters associated with the identified AA sequence. In case of a labeled experiment this is the total intensity of all the isotopic patterns in the label cluster.
Intensity FL0145_JHendy_130416_MSQ525_CW21___2		Summed up eXtracted Ion Current (XIC) of all isotopic clusters associated with the identified AA sequence. In case of a labeled experiment this is the total intensity of all the isotopic patterns in the label cluster.
Intensity FL0145_JHendy_130416_MSQ525_CW21___3		Summed up eXtracted Ion Current (XIC) of all isotopic clusters associated with the identified AA sequence. In case of a labeled experiment this is the total intensity of all the isotopic patterns in the label cluster.
Intensity FL0145_JHendy_130416_MSQ525_CW23___1		Summed up eXtracted Ion Current (XIC) of all isotopic clusters associated with the identified AA sequence. In case of a labeled experiment this is the total intensity of all the isotopic patterns in the label cluster.
Intensity FL0145_JHendy_130416_MSQ525_CW23___2		Summed up eXtracted Ion Current (XIC) of all isotopic clusters associated with the identified AA sequence. In case of a labeled experiment this is the total intensity of all the isotopic patterns in the label cluster.
Intensity FL0145_JHendy_130416_MSQ525_CW23___3		Summed up eXtracted Ion Current (XIC) of all isotopic clusters associated with the identified AA sequence. In case of a labeled experiment this is the total intensity of all the isotopic patterns in the label cluster.
Intensity FL0145_JHendy_130416_MSQ525_CW8_160425165036___1		Summed up eXtracted Ion Current (XIC) of all isotopic clusters associated with the identified AA sequence. In case of a labeled experiment this is the total intensity of all the isotopic patterns in the label cluster.
Intensity FL0145_JHendy_130416_MSQ525_CW8_160425165036___2		Summed up eXtracted Ion Current (XIC) of all isotopic clusters associated with the identified AA sequence. In case of a labeled experiment this is the total intensity of all the isotopic patterns in the label cluster.
Intensity FL0145_JHendy_130416_MSQ525_CW8_160425165036___3		Summed up eXtracted Ion Current (XIC) of all isotopic clusters associated with the identified AA sequence. In case of a labeled experiment this is the total intensity of all the isotopic patterns in the label cluster.

Intensity FL0168_JHendy_MSQ525_170516_BK___1		Summed up eXtracted Ion Current (XIC) of all isotopic clusters associated with the identified AA sequence. In case of a labeled experiment this is the total intensity of all the isotopic patterns in the label cluster.
Intensity FL0168_JHendy_MSQ525_170516_BK___2		Summed up eXtracted Ion Current (XIC) of all isotopic clusters associated with the identified AA sequence. In case of a labeled experiment this is the total intensity of all the isotopic patterns in the label cluster.
Intensity FL0168_JHendy_MSQ525_170516_BK___3		Summed up eXtracted Ion Current (XIC) of all isotopic clusters associated with the identified AA sequence. In case of a labeled experiment this is the total intensity of all the isotopic patterns in the label cluster.
Intensity FL0168_JHendy_MSQ525_170516_CW1___1		Summed up eXtracted Ion Current (XIC) of all isotopic clusters associated with the identified AA sequence. In case of a labeled experiment this is the total intensity of all the isotopic patterns in the label cluster.
Intensity FL0168_JHendy_MSQ525_170516_CW1___2		Summed up eXtracted Ion Current (XIC) of all isotopic clusters associated with the identified AA sequence. In case of a labeled experiment this is the total intensity of all the isotopic patterns in the label cluster.
Intensity FL0168_JHendy_MSQ525_170516_CW1___3		Summed up eXtracted Ion Current (XIC) of all isotopic clusters associated with the identified AA sequence. In case of a labeled experiment this is the total intensity of all the isotopic patterns in the label cluster.
Intensity FL0168_JHendy_MSQ525_170516_CW10___1		Summed up eXtracted Ion Current (XIC) of all isotopic clusters associated with the identified AA sequence. In case of a labeled experiment this is the total intensity of all the isotopic patterns in the label cluster.
Intensity FL0168_JHendy_MSQ525_170516_CW10___2		Summed up eXtracted Ion Current (XIC) of all isotopic clusters associated with the identified AA sequence. In case of a labeled experiment this is the total intensity of all the isotopic patterns in the label cluster.
Intensity FL0168_JHendy_MSQ525_170516_CW10___3		Summed up eXtracted Ion Current (XIC) of all isotopic clusters associated with the identified AA sequence. In case of a labeled experiment this is the total intensity of all the isotopic patterns in the label cluster.
Intensity FL0168_JHendy_MSQ525_170516_CW2___1		Summed up eXtracted Ion Current (XIC) of all isotopic clusters associated with the identified AA sequence. In case of a labeled experiment this is the total intensity of all the isotopic patterns in the label cluster.
Intensity FL0168_JHendy_MSQ525_170516_CW2___2		Summed up eXtracted Ion Current (XIC) of all isotopic clusters associated with the identified AA sequence. In case of a labeled experiment this is the total intensity of all the isotopic patterns in the label cluster.
Intensity FL0168_JHendy_MSQ525_170516_CW2___3		Summed up eXtracted Ion Current (XIC) of all isotopic clusters associated with the identified AA sequence. In case of a labeled experiment this is the total intensity of all the isotopic patterns in the label cluster.
Intensity FL0168_JHendy_MSQ525_170516_CW3___1		Summed up eXtracted Ion Current (XIC) of all isotopic clusters associated with the identified AA sequence. In case of a labeled experiment this is the total intensity of all the isotopic patterns in the label cluster.
Intensity FL0168_JHendy_MSQ525_170516_CW3___2		Summed up eXtracted Ion Current (XIC) of all isotopic clusters associated with the identified AA sequence. In case of a labeled experiment this is the total intensity of all the isotopic patterns in the label cluster.
Intensity FL0168_JHendy_MSQ525_170516_CW3___3		Summed up eXtracted Ion Current (XIC) of all isotopic clusters associated with the identified AA sequence. In case of a labeled experiment this is the total intensity of all the isotopic patterns in the label cluster.
Intensity FL0168_JHendy_MSQ525_170516_CW4___1		Summed up eXtracted Ion Current (XIC) of all isotopic clusters associated with the identified AA sequence. In case of a labeled experiment this is the total intensity of all the isotopic patterns in the label cluster.
Intensity FL0168_JHendy_MSQ525_170516_CW4___2		Summed up eXtracted Ion Current (XIC) of all isotopic clusters associated with the identified AA sequence. In case of a labeled experiment this is the total intensity of all the isotopic patterns in the label cluster.
Intensity FL0168_JHendy_MSQ525_170516_CW4___3		Summed up eXtracted Ion Current (XIC) of all isotopic clusters associated with the identified AA sequence. In case of a labeled experiment this is the total intensity of all the isotopic patterns in the label cluster.
Intensity FL0168_JHendy_MSQ525_170516_CW5___1		Summed up eXtracted Ion Current (XIC) of all isotopic clusters associated with the identified AA sequence. In case of a labeled experiment this is the total intensity of all the isotopic patterns in the label cluster.
Intensity FL0168_JHendy_MSQ525_170516_CW5___2		Summed up eXtracted Ion Current (XIC) of all isotopic clusters associated with the identified AA sequence. In case of a labeled experiment this is the total intensity of all the isotopic patterns in the label cluster.
Intensity FL0168_JHendy_MSQ525_170516_CW5___3		Summed up eXtracted Ion Current (XIC) of all isotopic clusters associated with the identified AA sequence. In case of a labeled experiment this is the total intensity of all the isotopic patterns in the label cluster.

Intensity FL0168_JHendy_MSQ525_170516_CW6___1		Summed up eXtracted Ion Current (XIC) of all isotopic clusters associated with the identified AA sequence. In case of a labeled experiment this is the total intensity of all the isotopic patterns in the label cluster.
Intensity FL0168_JHendy_MSQ525_170516_CW6___2		Summed up eXtracted Ion Current (XIC) of all isotopic clusters associated with the identified AA sequence. In case of a labeled experiment this is the total intensity of all the isotopic patterns in the label cluster.
Intensity FL0168_JHendy_MSQ525_170516_CW6___3		Summed up eXtracted Ion Current (XIC) of all isotopic clusters associated with the identified AA sequence. In case of a labeled experiment this is the total intensity of all the isotopic patterns in the label cluster.
Intensity FL0168_JHendy_MSQ525_170516_CW7___1		Summed up eXtracted Ion Current (XIC) of all isotopic clusters associated with the identified AA sequence. In case of a labeled experiment this is the total intensity of all the isotopic patterns in the label cluster.
Intensity FL0168_JHendy_MSQ525_170516_CW7___2		Summed up eXtracted Ion Current (XIC) of all isotopic clusters associated with the identified AA sequence. In case of a labeled experiment this is the total intensity of all the isotopic patterns in the label cluster.
Intensity FL0168_JHendy_MSQ525_170516_CW7___3		Summed up eXtracted Ion Current (XIC) of all isotopic clusters associated with the identified AA sequence. In case of a labeled experiment this is the total intensity of all the isotopic patterns in the label cluster.
Intensity FL0168_JHendy_MSQ525_170516_CW8___1		Summed up eXtracted Ion Current (XIC) of all isotopic clusters associated with the identified AA sequence. In case of a labeled experiment this is the total intensity of all the isotopic patterns in the label cluster.
Intensity FL0168_JHendy_MSQ525_170516_CW8___2		Summed up eXtracted Ion Current (XIC) of all isotopic clusters associated with the identified AA sequence. In case of a labeled experiment this is the total intensity of all the isotopic patterns in the label cluster.
Intensity FL0168_JHendy_MSQ525_170516_CW8___3		Summed up eXtracted Ion Current (XIC) of all isotopic clusters associated with the identified AA sequence. In case of a labeled experiment this is the total intensity of all the isotopic patterns in the label cluster.
Intensity FL0168_JHendy_MSQ525_170516_CW9___1		Summed up eXtracted Ion Current (XIC) of all isotopic clusters associated with the identified AA sequence. In case of a labeled experiment this is the total intensity of all the isotopic patterns in the label cluster.
Intensity FL0168_JHendy_MSQ525_170516_CW9___2		Summed up eXtracted Ion Current (XIC) of all isotopic clusters associated with the identified AA sequence. In case of a labeled experiment this is the total intensity of all the isotopic patterns in the label cluster.
Intensity FL0168_JHendy_MSQ525_170516_CW9___3		Summed up eXtracted Ion Current (XIC) of all isotopic clusters associated with the identified AA sequence. In case of a labeled experiment this is the total intensity of all the isotopic patterns in the label cluster.
Occupancy 20160805_QE5_nLC7_MEM_COLLAB_JH_A1_01		
Occupancy ratio20160805_QE5_nLC7_MEM_COLLAB_JH_A1_01		
Occupancy error scale 20160805_QE5_nLC7_MEM_COLLAB_JH_A1_01		
Occupancy 20160805_QE5_nLC7_MEM_COLLAB_JH_A2_01		
Occupancy ratio20160805_QE5_nLC7_MEM_COLLAB_JH_A2_01		
Occupancy error scale 20160805_QE5_nLC7_MEM_COLLAB_JH_A2_01		
Occupancy 20160805_QE5_nLC7_MEM_COLLAB_JH_A3_01		
Occupancy ratio20160805_QE5_nLC7_MEM_COLLAB_JH_A3_01		
Occupancy error scale 20160805_QE5_nLC7_MEM_COLLAB_JH_A3_01		
Occupancy 20160805_QE5_nLC7_MEM_COLLAB_JH_B1_01		
Occupancy ratio20160805_QE5_nLC7_MEM_COLLAB_JH_B1_01		
Occupancy error scale 20160805_QE5_nLC7_MEM_COLLAB_JH_B1_01		

Occupancy 20160805_QE5_nLC7_MEM_COLLAB_JH_B2_01		
Occupancy ratio20160805_QE5_nLC7_MEM_COLLAB_JH_B2_01		
Occupancy error scale 20160805_QE5_nLC7_MEM_COLLAB_JH_B2_01		
Occupancy 20160805_QE5_nLC7_MEM_COLLAB_JH_B3_01		
Occupancy ratio20160805_QE5_nLC7_MEM_COLLAB_JH_B3_01		
Occupancy error scale 20160805_QE5_nLC7_MEM_COLLAB_JH_B3_01		
Occupancy 20160805_QE5_nLC7_MEM_COLLAB_JH_B4_01		
Occupancy ratio20160805_QE5_nLC7_MEM_COLLAB_JH_B4_01		
Occupancy error scale 20160805_QE5_nLC7_MEM_COLLAB_JH_B4_01		
Occupancy 20160805_QE5_nLC7_MEM_COLLAB_JH_B5_01		
Occupancy ratio20160805_QE5_nLC7_MEM_COLLAB_JH_B5_01		
Occupancy error scale 20160805_QE5_nLC7_MEM_COLLAB_JH_B5_01		
Occupancy 20160805_QE5_nLC7_MEM_COLLAB_JH_C1_01		
Occupancy ratio20160805_QE5_nLC7_MEM_COLLAB_JH_C1_01		
Occupancy error scale 20160805_QE5_nLC7_MEM_COLLAB_JH_C1_01		
Occupancy 20160805_QE5_nLC7_MEM_COLLAB_JH_C2_01		
Occupancy ratio20160805_QE5_nLC7_MEM_COLLAB_JH_C2_01		
Occupancy error scale 20160805_QE5_nLC7_MEM_COLLAB_JH_C2_01		
Occupancy 20160805_QE5_nLC7_MEM_COLLAB_JH_C3_01		
Occupancy ratio20160805_QE5_nLC7_MEM_COLLAB_JH_C3_01		
Occupancy error scale 20160805_QE5_nLC7_MEM_COLLAB_JH_C3_01		
Occupancy 20160805_QE5_nLC7_MEM_COLLAB_JH_C4_01		
Occupancy ratio20160805_QE5_nLC7_MEM_COLLAB_JH_C4_01		
Occupancy error scale 20160805_QE5_nLC7_MEM_COLLAB_JH_C4_01		
Occupancy 20160805_QE5_nLC7_MEM_COLLAB_JH_C5_01		
Occupancy ratio20160805_QE5_nLC7_MEM_COLLAB_JH_C5_01		
Occupancy error scale 20160805_QE5_nLC7_MEM_COLLAB_JH_C5_01		

Occupancy 20160805_QE5_nLC7_MEM_COLLAB_JH_C6_01		
Occupancy ratio20160805_QE5_nLC7_MEM_COLLAB_JH_C6_01		
Occupancy error scale 20160805_QE5_nLC7_MEM_COLLAB_JH_C6_01		
Occupancy 20160805_QE5_nLC7_MEM_COLLAB_JH_C7_01		
Occupancy ratio20160805_QE5_nLC7_MEM_COLLAB_JH_C7_01		
Occupancy error scale 20160805_QE5_nLC7_MEM_COLLAB_JH_C7_01		
Occupancy 20160805_QE5_nLC7_MEM_COLLAB_JH_C8_01		
Occupancy ratio20160805_QE5_nLC7_MEM_COLLAB_JH_C8_01		
Occupancy error scale 20160805_QE5_nLC7_MEM_COLLAB_JH_C8_01		
Occupancy FL0145_JHendy_130416_MSQ525_CW10		
Occupancy ratioFL0145_JHendy_130416_MSQ525_CW10		
Occupancy error scale FL0145_JHendy_130416_MSQ525_CW10		
Occupancy FL0145_JHendy_130416_MSQ525_CW11		
Occupancy ratioFL0145_JHendy_130416_MSQ525_CW11		
Occupancy error scale FL0145_JHendy_130416_MSQ525_CW11		
Occupancy FL0145_JHendy_130416_MSQ525_CW18		
Occupancy ratioFL0145_JHendy_130416_MSQ525_CW18		
Occupancy error scale FL0145_JHendy_130416_MSQ525_CW18		
Occupancy FL0145_JHendy_130416_MSQ525_CW20		
Occupancy ratioFL0145_JHendy_130416_MSQ525_CW20		
Occupancy error scale FL0145_JHendy_130416_MSQ525_CW20		
Occupancy FL0145_JHendy_130416_MSQ525_CW21		
Occupancy ratioFL0145_JHendy_130416_MSQ525_CW21		
Occupancy error scale FL0145_JHendy_130416_MSQ525_CW21		
Occupancy FL0145_JHendy_130416_MSQ525_CW23		
Occupancy ratioFL0145_JHendy_130416_MSQ525_CW23		
Occupancy error scale FL0145_JHendy_130416_MSQ525_CW23		

Occupancy FL0145_JHendy_130416_MSQ525_CW8_160425165036		
Occupancy ratioFL0145_JHendy_130416_MSQ525_CW8_160425165036		
Occupancy error scale FL0145_JHendy_130416_MSQ525_CW8_160425165036		
Occupancy FL0168_JHendy_MSQ525_170516_BK		
Occupancy ratioFL0168_JHendy_MSQ525_170516_BK		
Occupancy error scale FL0168_JHendy_MSQ525_170516_BK		
Occupancy FL0168_JHendy_MSQ525_170516_CW1		
Occupancy ratioFL0168_JHendy_MSQ525_170516_CW1		
Occupancy error scale FL0168_JHendy_MSQ525_170516_CW1		
Occupancy FL0168_JHendy_MSQ525_170516_CW10		
Occupancy ratioFL0168_JHendy_MSQ525_170516_CW10		
Occupancy error scale FL0168_JHendy_MSQ525_170516_CW10		
Occupancy FL0168_JHendy_MSQ525_170516_CW2		
Occupancy ratioFL0168_JHendy_MSQ525_170516_CW2		
Occupancy error scale FL0168_JHendy_MSQ525_170516_CW2		
Occupancy FL0168_JHendy_MSQ525_170516_CW3		
Occupancy ratioFL0168_JHendy_MSQ525_170516_CW3		
Occupancy error scale FL0168_JHendy_MSQ525_170516_CW3		
Occupancy FL0168_JHendy_MSQ525_170516_CW4		
Occupancy ratioFL0168_JHendy_MSQ525_170516_CW4		
Occupancy error scale FL0168_JHendy_MSQ525_170516_CW4		
Occupancy FL0168_JHendy_MSQ525_170516_CW5		
Occupancy ratioFL0168_JHendy_MSQ525_170516_CW5		
Occupancy error scale FL0168_JHendy_MSQ525_170516_CW5		
Occupancy FL0168_JHendy_MSQ525_170516_CW6		
Occupancy ratioFL0168_JHendy_MSQ525_170516_CW6		
Occupancy error scale FL0168_JHendy_MSQ525_170516_CW6		

Occupancy FL0168_JHendy_MSQ525_170516_CW7		
Occupancy ratioFL0168_JHendy_MSQ525_170516_CW7		
Occupancy error scale FL0168_JHendy_MSQ525_170516_CW7		
Occupancy FL0168_JHendy_MSQ525_170516_CW8		
Occupancy ratioFL0168_JHendy_MSQ525_170516_CW8		
Occupancy error scale FL0168_JHendy_MSQ525_170516_CW8		
Occupancy FL0168_JHendy_MSQ525_170516_CW9		
Occupancy ratioFL0168_JHendy_MSQ525_170516_CW9		
Occupancy error scale FL0168_JHendy_MSQ525_170516_CW9		
Reverse		When marked with '+', this particular peptide was found to be part of a protein derived from the reversed part of the protein sequence database. These should be removed for further data analysis.
Potential contaminant		When marked with '+', this particular peptide was found to be part of a commonly occurring contaminant. These should be removed for further data analysis.
id		A unique (consecutive) identifier for each row in the site table, which is used to cross-link the information in this file with the information stored in the other files.
Protein group IDs		The identifier of the protein-group this peptide sequence is associated with, which can be used to look up the extended protein information in the file 'proteinGroups.txt'.<br>As a single peptide can be linked to multiple proteins (e.g. in the case of razor-proteins), multiple id's can be stored here separated by a semicolon.<br>As a protein can be identified by multiple peptides, the same id can be found in different rows.
Positions		The positions of the modifications in the protein amino acid sequence.
Position		The position of the modification in the protein amino acid sequence.
Peptide IDs		Identifier(s) of the associated peptide sequence(s) summary, which can be found in the file 'peptides.txt'.
Mod. peptide IDs		Identifier(s) of the associated peptide sequence(s) summary, which can be found in the file 'modificationSpecificPeptides.txt'.
Evidence IDs		Identifier(s) for analyzed peptide evidence associated with the protein group referenced against the evidence table.
MS/MS IDs		The identifiers of the MS/MS scans identifying this peptide, referenced against the msms table.
Best localization evidence ID		
Best localization MS/MS ID		
Best localization raw file		
Best localization scan number		
Best score evidence ID		
Best score MS/MS ID		
Best score raw file		
Best score scan number		
Best PEP evidence ID		
Best PEP MS/MS ID		
Best PEP raw file		
Best PEP scan number		

Oxidation (M)Sites

Name	Separator	Description
Proteins		Identifiers of proteins this site is associated with.
Positions within proteins		For each protein identifier in the 'Proteins' column you find here the position of the site in the respective protein sequence. The index of the first amino acid in the sequence is 1.
Leading proteins		
Protein		Identifier of the protein this peptide is associated with.
Fasta headers		Descriptions of proteins this peptide is associated with.
Localization prob		
Score diff		
PEP		The posterior error probability (PEP) of the best identified modified peptide containing this site.
Score		The Andromeda score of the best identified modified peptide containing this site.
Delta score		The Andromeda delta score of the best identified modified peptide containing this site.
Score for localization		The Andromeda score of the MS/MS spectrum used for calculating the localization score for this site.
Localization prob 20160805_QE5_nLC7_MEM_COLLAB_JH_A1_01		
Score diff 20160805_QE5_nLC7_MEM_COLLAB_JH_A1_01		
PEP 20160805_QE5_nLC7_MEM_COLLAB_JH_A1_01		
Score 20160805_QE5_nLC7_MEM_COLLAB_JH_A1_01		
Localization prob 20160805_QE5_nLC7_MEM_COLLAB_JH_A2_01		
Score diff 20160805_QE5_nLC7_MEM_COLLAB_JH_A2_01		
PEP 20160805_QE5_nLC7_MEM_COLLAB_JH_A2_01		
Score 20160805_QE5_nLC7_MEM_COLLAB_JH_A2_01		
Localization prob 20160805_QE5_nLC7_MEM_COLLAB_JH_A3_01		
Score diff 20160805_QE5_nLC7_MEM_COLLAB_JH_A3_01		
PEP 20160805_QE5_nLC7_MEM_COLLAB_JH_A3_01		
Score 20160805_QE5_nLC7_MEM_COLLAB_JH_A3_01		
Localization prob 20160805_QE5_nLC7_MEM_COLLAB_JH_B1_01		
Score diff 20160805_QE5_nLC7_MEM_COLLAB_JH_B1_01		
PEP 20160805_QE5_nLC7_MEM_COLLAB_JH_B1_01		
Score 20160805_QE5_nLC7_MEM_COLLAB_JH_B1_01		
Localization prob 20160805_QE5_nLC7_MEM_COLLAB_JH_B2_01		
Score diff 20160805_QE5_nLC7_MEM_COLLAB_JH_B2_01		

PEP 20160805_QE5_nLC7_MEM_COLLAB_JH_B2_01		
Score 20160805_QE5_nLC7_MEM_COLLAB_JH_B2_01		
Localization prob 20160805_QE5_nLC7_MEM_COLLAB_JH_B3_01		
Score diff 20160805_QE5_nLC7_MEM_COLLAB_JH_B3_01		
PEP 20160805_QE5_nLC7_MEM_COLLAB_JH_B3_01		
Score 20160805_QE5_nLC7_MEM_COLLAB_JH_B3_01		
Localization prob 20160805_QE5_nLC7_MEM_COLLAB_JH_B4_01		
Score diff 20160805_QE5_nLC7_MEM_COLLAB_JH_B4_01		
PEP 20160805_QE5_nLC7_MEM_COLLAB_JH_B4_01		
Score 20160805_QE5_nLC7_MEM_COLLAB_JH_B4_01		
Localization prob 20160805_QE5_nLC7_MEM_COLLAB_JH_B5_01		
Score diff 20160805_QE5_nLC7_MEM_COLLAB_JH_B5_01		
PEP 20160805_QE5_nLC7_MEM_COLLAB_JH_B5_01		
Score 20160805_QE5_nLC7_MEM_COLLAB_JH_B5_01		
Localization prob 20160805_QE5_nLC7_MEM_COLLAB_JH_C1_01		
Score diff 20160805_QE5_nLC7_MEM_COLLAB_JH_C1_01		
PEP 20160805_QE5_nLC7_MEM_COLLAB_JH_C1_01		
Score 20160805_QE5_nLC7_MEM_COLLAB_JH_C1_01		
Localization prob 20160805_QE5_nLC7_MEM_COLLAB_JH_C2_01		
Score diff 20160805_QE5_nLC7_MEM_COLLAB_JH_C2_01		
PEP 20160805_QE5_nLC7_MEM_COLLAB_JH_C2_01		
Score 20160805_QE5_nLC7_MEM_COLLAB_JH_C2_01		
Localization prob 20160805_QE5_nLC7_MEM_COLLAB_JH_C3_01		
Score diff 20160805_QE5_nLC7_MEM_COLLAB_JH_C3_01		
PEP 20160805_QE5_nLC7_MEM_COLLAB_JH_C3_01		
Score 20160805_QE5_nLC7_MEM_COLLAB_JH_C3_01		
Localization prob 20160805_QE5_nLC7_MEM_COLLAB_JH_C4_01		

Score diff 20160805_QE5_nLC7_MEM_COLLAB_JH_C4_01		
PEP 20160805_QE5_nLC7_MEM_COLLAB_JH_C4_01		
Score 20160805_QE5_nLC7_MEM_COLLAB_JH_C4_01		
Localization prob 20160805_QE5_nLC7_MEM_COLLAB_JH_C5_01		
Score diff 20160805_QE5_nLC7_MEM_COLLAB_JH_C5_01		
PEP 20160805_QE5_nLC7_MEM_COLLAB_JH_C5_01		
Score 20160805_QE5_nLC7_MEM_COLLAB_JH_C5_01		
Localization prob 20160805_QE5_nLC7_MEM_COLLAB_JH_C6_01		
Score diff 20160805_QE5_nLC7_MEM_COLLAB_JH_C6_01		
PEP 20160805_QE5_nLC7_MEM_COLLAB_JH_C6_01		
Score 20160805_QE5_nLC7_MEM_COLLAB_JH_C6_01		
Localization prob 20160805_QE5_nLC7_MEM_COLLAB_JH_C7_01		
Score diff 20160805_QE5_nLC7_MEM_COLLAB_JH_C7_01		
PEP 20160805_QE5_nLC7_MEM_COLLAB_JH_C7_01		
Score 20160805_QE5_nLC7_MEM_COLLAB_JH_C7_01		
Localization prob 20160805_QE5_nLC7_MEM_COLLAB_JH_C8_01		
Score diff 20160805_QE5_nLC7_MEM_COLLAB_JH_C8_01		
PEP 20160805_QE5_nLC7_MEM_COLLAB_JH_C8_01		
Score 20160805_QE5_nLC7_MEM_COLLAB_JH_C8_01		
Localization prob FL0145_JHendy_130416_MSQ525_CW10		
Score diff FL0145_JHendy_130416_MSQ525_CW10		
PEP FL0145_JHendy_130416_MSQ525_CW10		
Score FL0145_JHendy_130416_MSQ525_CW10		
Localization prob FL0145_JHendy_130416_MSQ525_CW11		
Score diff FL0145_JHendy_130416_MSQ525_CW11		
PEP FL0145_JHendy_130416_MSQ525_CW11		
Score FL0145_JHendy_130416_MSQ525_CW11		

Localization prob FL0145_JHendy_130416_MSQ525_CW18		
Score diff FL0145_JHendy_130416_MSQ525_CW18		
PEP FL0145_JHendy_130416_MSQ525_CW18		
Score FL0145_JHendy_130416_MSQ525_CW18		
Localization prob FL0145_JHendy_130416_MSQ525_CW20		
Score diff FL0145_JHendy_130416_MSQ525_CW20		
PEP FL0145_JHendy_130416_MSQ525_CW20		
Score FL0145_JHendy_130416_MSQ525_CW20		
Localization prob FL0145_JHendy_130416_MSQ525_CW21		
Score diff FL0145_JHendy_130416_MSQ525_CW21		
PEP FL0145_JHendy_130416_MSQ525_CW21		
Score FL0145_JHendy_130416_MSQ525_CW21		
Localization prob FL0145_JHendy_130416_MSQ525_CW23		
Score diff FL0145_JHendy_130416_MSQ525_CW23		
PEP FL0145_JHendy_130416_MSQ525_CW23		
Score FL0145_JHendy_130416_MSQ525_CW23		
Localization prob FL0145_JHendy_130416_MSQ525_CW8_160425165036		
Score diff FL0145_JHendy_130416_MSQ525_CW8_160425165036		
PEP FL0145_JHendy_130416_MSQ525_CW8_160425165036		
Score FL0145_JHendy_130416_MSQ525_CW8_160425165036		
Localization prob FL0168_JHendy_MSQ525_170516_BK		
Score diff FL0168_JHendy_MSQ525_170516_BK		
PEP FL0168_JHendy_MSQ525_170516_BK		
Score FL0168_JHendy_MSQ525_170516_BK		
Localization prob FL0168_JHendy_MSQ525_170516_CW1		
Score diff FL0168_JHendy_MSQ525_170516_CW1		
PEP FL0168_JHendy_MSQ525_170516_CW1		

Score FL0168_JHendy_MSQ525_170516_CW1		
Localization prob FL0168_JHendy_MSQ525_170516_CW10		
Score diff FL0168_JHendy_MSQ525_170516_CW10		
PEP FL0168_JHendy_MSQ525_170516_CW10		
Score FL0168_JHendy_MSQ525_170516_CW10		
Localization prob FL0168_JHendy_MSQ525_170516_CW2		
Score diff FL0168_JHendy_MSQ525_170516_CW2		
PEP FL0168_JHendy_MSQ525_170516_CW2		
Score FL0168_JHendy_MSQ525_170516_CW2		
Localization prob FL0168_JHendy_MSQ525_170516_CW3		
Score diff FL0168_JHendy_MSQ525_170516_CW3		
PEP FL0168_JHendy_MSQ525_170516_CW3		
Score FL0168_JHendy_MSQ525_170516_CW3		
Localization prob FL0168_JHendy_MSQ525_170516_CW4		
Score diff FL0168_JHendy_MSQ525_170516_CW4		
PEP FL0168_JHendy_MSQ525_170516_CW4		
Score FL0168_JHendy_MSQ525_170516_CW4		
Localization prob FL0168_JHendy_MSQ525_170516_CW5		
Score diff FL0168_JHendy_MSQ525_170516_CW5		
PEP FL0168_JHendy_MSQ525_170516_CW5		
Score FL0168_JHendy_MSQ525_170516_CW5		
Localization prob FL0168_JHendy_MSQ525_170516_CW6		
Score diff FL0168_JHendy_MSQ525_170516_CW6		
PEP FL0168_JHendy_MSQ525_170516_CW6		
Score FL0168_JHendy_MSQ525_170516_CW6		
Localization prob FL0168_JHendy_MSQ525_170516_CW7		
Score diff FL0168_JHendy_MSQ525_170516_CW7		

PEP FL0168_JHendy_MSQ525_170516_CW7		
Score FL0168_JHendy_MSQ525_170516_CW7		
Localization prob FL0168_JHendy_MSQ525_170516_CW8		
Score diff FL0168_JHendy_MSQ525_170516_CW8		
PEP FL0168_JHendy_MSQ525_170516_CW8		
Score FL0168_JHendy_MSQ525_170516_CW8		
Localization prob FL0168_JHendy_MSQ525_170516_CW9		
Score diff FL0168_JHendy_MSQ525_170516_CW9		
PEP FL0168_JHendy_MSQ525_170516_CW9		
Score FL0168_JHendy_MSQ525_170516_CW9		
Diagnostic peak		
Number of Oxidation (M)		Different numbers of Oxidation (M) on peptides that this site is involved in.
Amino acid		
Sequence window		
Modification window		
Peptide window coverage		
Oxidation (M) Probabilities		
Oxidation (M) Score diffs		
Position in peptide		
Charge		Charge state of the precursor ion.
Mass error [ppm]		Mass error of the recalibrated mass-over-charge value of the precursor ion in comparison to the predicted monoisotopic mass of the identified peptide sequence.
Intensity		Summed up eXtracted Ion Current (XIC) of all isotopic clusters associated with the identified AA sequence. In case of a labeled experiment this is the total intensity of all the isotopic patterns in the label cluster.
Intensity___1		Summed up eXtracted Ion Current (XIC) of all isotopic clusters associated with the identified AA sequence. In case of a labeled experiment this is the total intensity of all the isotopic patterns in the label cluster.
Intensity___2		Summed up eXtracted Ion Current (XIC) of all isotopic clusters associated with the identified AA sequence. In case of a labeled experiment this is the total intensity of all the isotopic patterns in the label cluster.
Intensity___3		Summed up eXtracted Ion Current (XIC) of all isotopic clusters associated with the identified AA sequence. In case of a labeled experiment this is the total intensity of all the isotopic patterns in the label cluster.
Ratio mod/base		
Intensity 20160805_QE5_nLC7_MEM_COLLAB_JH_A1_01		Summed up eXtracted Ion Current (XIC) of all isotopic clusters associated with the identified AA sequence. In case of a labeled experiment this is the total intensity of all the isotopic patterns in the label cluster.
Intensity 20160805_QE5_nLC7_MEM_COLLAB_JH_A2_01		Summed up eXtracted Ion Current (XIC) of all isotopic clusters associated with the identified AA sequence. In case of a labeled experiment this is the total intensity of all the isotopic patterns in the label cluster.
Intensity 20160805_QE5_nLC7_MEM_COLLAB_JH_A3_01		Summed up eXtracted Ion Current (XIC) of all isotopic clusters associated with the identified AA sequence. In case of a labeled experiment this is the total intensity of all the isotopic patterns in the label cluster.
Intensity 20160805_QE5_nLC7_MEM_COLLAB_JH_B1_01		Summed up eXtracted Ion Current (XIC) of all isotopic clusters associated with the identified AA sequence. In case of a labeled experiment this is the total intensity of all the isotopic patterns in the label cluster.

Intensity 20160805_QE5_nLC7_MEM_COLLAB_JH_B2_01		Summed up eXtracted Ion Current (XIC) of all isotopic clusters associated with the identified AA sequence. In case of a labeled experiment this is the total intensity of all the isotopic patterns in the label cluster.
Intensity 20160805_QE5_nLC7_MEM_COLLAB_JH_B3_01		Summed up eXtracted Ion Current (XIC) of all isotopic clusters associated with the identified AA sequence. In case of a labeled experiment this is the total intensity of all the isotopic patterns in the label cluster.
Intensity 20160805_QE5_nLC7_MEM_COLLAB_JH_B4_01		Summed up eXtracted Ion Current (XIC) of all isotopic clusters associated with the identified AA sequence. In case of a labeled experiment this is the total intensity of all the isotopic patterns in the label cluster.
Intensity 20160805_QE5_nLC7_MEM_COLLAB_JH_B5_01		Summed up eXtracted Ion Current (XIC) of all isotopic clusters associated with the identified AA sequence. In case of a labeled experiment this is the total intensity of all the isotopic patterns in the label cluster.
Intensity 20160805_QE5_nLC7_MEM_COLLAB_JH_C1_01		Summed up eXtracted Ion Current (XIC) of all isotopic clusters associated with the identified AA sequence. In case of a labeled experiment this is the total intensity of all the isotopic patterns in the label cluster.
Intensity 20160805_QE5_nLC7_MEM_COLLAB_JH_C2_01		Summed up eXtracted Ion Current (XIC) of all isotopic clusters associated with the identified AA sequence. In case of a labeled experiment this is the total intensity of all the isotopic patterns in the label cluster.
Intensity 20160805_QE5_nLC7_MEM_COLLAB_JH_C3_01		Summed up eXtracted Ion Current (XIC) of all isotopic clusters associated with the identified AA sequence. In case of a labeled experiment this is the total intensity of all the isotopic patterns in the label cluster.
Intensity 20160805_QE5_nLC7_MEM_COLLAB_JH_C4_01		Summed up eXtracted Ion Current (XIC) of all isotopic clusters associated with the identified AA sequence. In case of a labeled experiment this is the total intensity of all the isotopic patterns in the label cluster.
Intensity 20160805_QE5_nLC7_MEM_COLLAB_JH_C5_01		Summed up eXtracted Ion Current (XIC) of all isotopic clusters associated with the identified AA sequence. In case of a labeled experiment this is the total intensity of all the isotopic patterns in the label cluster.
Intensity 20160805_QE5_nLC7_MEM_COLLAB_JH_C6_01		Summed up eXtracted Ion Current (XIC) of all isotopic clusters associated with the identified AA sequence. In case of a labeled experiment this is the total intensity of all the isotopic patterns in the label cluster.
Intensity 20160805_QE5_nLC7_MEM_COLLAB_JH_C7_01		Summed up eXtracted Ion Current (XIC) of all isotopic clusters associated with the identified AA sequence. In case of a labeled experiment this is the total intensity of all the isotopic patterns in the label cluster.
Intensity 20160805_QE5_nLC7_MEM_COLLAB_JH_C8_01		Summed up eXtracted Ion Current (XIC) of all isotopic clusters associated with the identified AA sequence. In case of a labeled experiment this is the total intensity of all the isotopic patterns in the label cluster.
Intensity FL0145_JHendy_130416_MSQ525_CW10		Summed up eXtracted Ion Current (XIC) of all isotopic clusters associated with the identified AA sequence. In case of a labeled experiment this is the total intensity of all the isotopic patterns in the label cluster.
Intensity FL0145_JHendy_130416_MSQ525_CW11		Summed up eXtracted Ion Current (XIC) of all isotopic clusters associated with the identified AA sequence. In case of a labeled experiment this is the total intensity of all the isotopic patterns in the label cluster.
Intensity FL0145_JHendy_130416_MSQ525_CW18		Summed up eXtracted Ion Current (XIC) of all isotopic clusters associated with the identified AA sequence. In case of a labeled experiment this is the total intensity of all the isotopic patterns in the label cluster.
Intensity FL0145_JHendy_130416_MSQ525_CW20		Summed up eXtracted Ion Current (XIC) of all isotopic clusters associated with the identified AA sequence. In case of a labeled experiment this is the total intensity of all the isotopic patterns in the label cluster.
Intensity FL0145_JHendy_130416_MSQ525_CW21		Summed up eXtracted Ion Current (XIC) of all isotopic clusters associated with the identified AA sequence. In case of a labeled experiment this is the total intensity of all the isotopic patterns in the label cluster.
Intensity FL0145_JHendy_130416_MSQ525_CW23		Summed up eXtracted Ion Current (XIC) of all isotopic clusters associated with the identified AA sequence. In case of a labeled experiment this is the total intensity of all the isotopic patterns in the label cluster.
Intensity FL0145_JHendy_130416_MSQ525_CW8_160425165036		Summed up eXtracted Ion Current (XIC) of all isotopic clusters associated with the identified AA sequence. In case of a labeled experiment this is the total intensity of all the isotopic patterns in the label cluster.
Intensity FL0168_JHendy_MSQ525_170516_BK		Summed up eXtracted Ion Current (XIC) of all isotopic clusters associated with the identified AA sequence. In case of a labeled experiment this is the total intensity of all the isotopic patterns in the label cluster.
Intensity FL0168_JHendy_MSQ525_170516_CW1		Summed up eXtracted Ion Current (XIC) of all isotopic clusters associated with the identified AA sequence. In case of a labeled experiment this is the total intensity of all the isotopic patterns in the label cluster.

Intensity FL0168_JHendy_MSQ525_170516_CW10		Summed up eXtracted Ion Current (XIC) of all isotopic clusters associated with the identified AA sequence. In case of a labeled experiment this is the total intensity of all the isotopic patterns in the label cluster.
Intensity FL0168_JHendy_MSQ525_170516_CW2		Summed up eXtracted Ion Current (XIC) of all isotopic clusters associated with the identified AA sequence. In case of a labeled experiment this is the total intensity of all the isotopic patterns in the label cluster.
Intensity FL0168_JHendy_MSQ525_170516_CW3		Summed up eXtracted Ion Current (XIC) of all isotopic clusters associated with the identified AA sequence. In case of a labeled experiment this is the total intensity of all the isotopic patterns in the label cluster.
Intensity FL0168_JHendy_MSQ525_170516_CW4		Summed up eXtracted Ion Current (XIC) of all isotopic clusters associated with the identified AA sequence. In case of a labeled experiment this is the total intensity of all the isotopic patterns in the label cluster.
Intensity FL0168_JHendy_MSQ525_170516_CW5		Summed up eXtracted Ion Current (XIC) of all isotopic clusters associated with the identified AA sequence. In case of a labeled experiment this is the total intensity of all the isotopic patterns in the label cluster.
Intensity FL0168_JHendy_MSQ525_170516_CW6		Summed up eXtracted Ion Current (XIC) of all isotopic clusters associated with the identified AA sequence. In case of a labeled experiment this is the total intensity of all the isotopic patterns in the label cluster.
Intensity FL0168_JHendy_MSQ525_170516_CW7		Summed up eXtracted Ion Current (XIC) of all isotopic clusters associated with the identified AA sequence. In case of a labeled experiment this is the total intensity of all the isotopic patterns in the label cluster.
Intensity FL0168_JHendy_MSQ525_170516_CW8		Summed up eXtracted Ion Current (XIC) of all isotopic clusters associated with the identified AA sequence. In case of a labeled experiment this is the total intensity of all the isotopic patterns in the label cluster.
Intensity FL0168_JHendy_MSQ525_170516_CW9		Summed up eXtracted Ion Current (XIC) of all isotopic clusters associated with the identified AA sequence. In case of a labeled experiment this is the total intensity of all the isotopic patterns in the label cluster.
Ratio mod/base 20160805_QE5_nLC7_MEM_COLLAB_JH_A1_01		
Ratio mod/base 20160805_QE5_nLC7_MEM_COLLAB_JH_A2_01		
Ratio mod/base 20160805_QE5_nLC7_MEM_COLLAB_JH_A3_01		
Ratio mod/base 20160805_QE5_nLC7_MEM_COLLAB_JH_B1_01		
Ratio mod/base 20160805_QE5_nLC7_MEM_COLLAB_JH_B2_01		
Ratio mod/base 20160805_QE5_nLC7_MEM_COLLAB_JH_B3_01		
Ratio mod/base 20160805_QE5_nLC7_MEM_COLLAB_JH_B4_01		
Ratio mod/base 20160805_QE5_nLC7_MEM_COLLAB_JH_B5_01		
Ratio mod/base 20160805_QE5_nLC7_MEM_COLLAB_JH_C1_01		
Ratio mod/base 20160805_QE5_nLC7_MEM_COLLAB_JH_C2_01		
Ratio mod/base 20160805_QE5_nLC7_MEM_COLLAB_JH_C3_01		
Ratio mod/base 20160805_QE5_nLC7_MEM_COLLAB_JH_C4_01		
Ratio mod/base 20160805_QE5_nLC7_MEM_COLLAB_JH_C5_01		
Ratio mod/base 20160805_QE5_nLC7_MEM_COLLAB_JH_C6_01		
Ratio mod/base 20160805_QE5_nLC7_MEM_COLLAB_JH_C7_01		

Ratio mod/base 20160805_QE5_nLC7_MEM_COLLAB_JH_C8_01		
Ratio mod/base FL0145_JHendy_130416_MSQ525_CW10		
Ratio mod/base FL0145_JHendy_130416_MSQ525_CW11		
Ratio mod/base FL0145_JHendy_130416_MSQ525_CW18		
Ratio mod/base FL0145_JHendy_130416_MSQ525_CW20		
Ratio mod/base FL0145_JHendy_130416_MSQ525_CW21		
Ratio mod/base FL0145_JHendy_130416_MSQ525_CW23		
Ratio mod/base FL0145_JHendy_130416_MSQ525_CW8_160425165036		
Ratio mod/base FL0168_JHendy_MSQ525_170516_BK		
Ratio mod/base FL0168_JHendy_MSQ525_170516_CW1		
Ratio mod/base FL0168_JHendy_MSQ525_170516_CW10		
Ratio mod/base FL0168_JHendy_MSQ525_170516_CW2		
Ratio mod/base FL0168_JHendy_MSQ525_170516_CW3		
Ratio mod/base FL0168_JHendy_MSQ525_170516_CW4		
Ratio mod/base FL0168_JHendy_MSQ525_170516_CW5		
Ratio mod/base FL0168_JHendy_MSQ525_170516_CW6		
Ratio mod/base FL0168_JHendy_MSQ525_170516_CW7		
Ratio mod/base FL0168_JHendy_MSQ525_170516_CW8		
Ratio mod/base FL0168_JHendy_MSQ525_170516_CW9		
Intensity 20160805_QE5_nLC7_MEM_COLLAB_JH_A1_01___1		Summed up eXtracted Ion Current (XIC) of all isotopic clusters associated with the identified AA sequence. In case of a labeled experiment this is the total intensity of all the isotopic patterns in the label cluster.
Intensity 20160805_QE5_nLC7_MEM_COLLAB_JH_A1_01___2		Summed up eXtracted Ion Current (XIC) of all isotopic clusters associated with the identified AA sequence. In case of a labeled experiment this is the total intensity of all the isotopic patterns in the label cluster.
Intensity 20160805_QE5_nLC7_MEM_COLLAB_JH_A1_01___3		Summed up eXtracted Ion Current (XIC) of all isotopic clusters associated with the identified AA sequence. In case of a labeled experiment this is the total intensity of all the isotopic patterns in the label cluster.
Intensity 20160805_QE5_nLC7_MEM_COLLAB_JH_A2_01___1		Summed up eXtracted Ion Current (XIC) of all isotopic clusters associated with the identified AA sequence. In case of a labeled experiment this is the total intensity of all the isotopic patterns in the label cluster.
Intensity 20160805_QE5_nLC7_MEM_COLLAB_JH_A2_01___2		Summed up eXtracted Ion Current (XIC) of all isotopic clusters associated with the identified AA sequence. In case of a labeled experiment this is the total intensity of all the isotopic patterns in the label cluster.
Intensity 20160805_QE5_nLC7_MEM_COLLAB_JH_A2_01___3		Summed up eXtracted Ion Current (XIC) of all isotopic clusters associated with the identified AA sequence. In case of a labeled experiment this is the total intensity of all the isotopic patterns in the label cluster.

Intensity 20160805_QE5_nLC7_MEM_COLLAB_JH_A3_01___1		Summed up eXtracted Ion Current (XIC) of all isotopic clusters associated with the identified AA sequence. In case of a labeled experiment this is the total intensity of all the isotopic patterns in the label cluster.
Intensity 20160805_QE5_nLC7_MEM_COLLAB_JH_A3_01___2		Summed up eXtracted Ion Current (XIC) of all isotopic clusters associated with the identified AA sequence. In case of a labeled experiment this is the total intensity of all the isotopic patterns in the label cluster.
Intensity 20160805_QE5_nLC7_MEM_COLLAB_JH_A3_01___3		Summed up eXtracted Ion Current (XIC) of all isotopic clusters associated with the identified AA sequence. In case of a labeled experiment this is the total intensity of all the isotopic patterns in the label cluster.
Intensity 20160805_QE5_nLC7_MEM_COLLAB_JH_B1_01___1		Summed up eXtracted Ion Current (XIC) of all isotopic clusters associated with the identified AA sequence. In case of a labeled experiment this is the total intensity of all the isotopic patterns in the label cluster.
Intensity 20160805_QE5_nLC7_MEM_COLLAB_JH_B1_01___2		Summed up eXtracted Ion Current (XIC) of all isotopic clusters associated with the identified AA sequence. In case of a labeled experiment this is the total intensity of all the isotopic patterns in the label cluster.
Intensity 20160805_QE5_nLC7_MEM_COLLAB_JH_B1_01___3		Summed up eXtracted Ion Current (XIC) of all isotopic clusters associated with the identified AA sequence. In case of a labeled experiment this is the total intensity of all the isotopic patterns in the label cluster.
Intensity 20160805_QE5_nLC7_MEM_COLLAB_JH_B2_01___1		Summed up eXtracted Ion Current (XIC) of all isotopic clusters associated with the identified AA sequence. In case of a labeled experiment this is the total intensity of all the isotopic patterns in the label cluster.
Intensity 20160805_QE5_nLC7_MEM_COLLAB_JH_B2_01___2		Summed up eXtracted Ion Current (XIC) of all isotopic clusters associated with the identified AA sequence. In case of a labeled experiment this is the total intensity of all the isotopic patterns in the label cluster.
Intensity 20160805_QE5_nLC7_MEM_COLLAB_JH_B2_01___3		Summed up eXtracted Ion Current (XIC) of all isotopic clusters associated with the identified AA sequence. In case of a labeled experiment this is the total intensity of all the isotopic patterns in the label cluster.
Intensity 20160805_QE5_nLC7_MEM_COLLAB_JH_B3_01___1		Summed up eXtracted Ion Current (XIC) of all isotopic clusters associated with the identified AA sequence. In case of a labeled experiment this is the total intensity of all the isotopic patterns in the label cluster.
Intensity 20160805_QE5_nLC7_MEM_COLLAB_JH_B3_01___2		Summed up eXtracted Ion Current (XIC) of all isotopic clusters associated with the identified AA sequence. In case of a labeled experiment this is the total intensity of all the isotopic patterns in the label cluster.
Intensity 20160805_QE5_nLC7_MEM_COLLAB_JH_B3_01___3		Summed up eXtracted Ion Current (XIC) of all isotopic clusters associated with the identified AA sequence. In case of a labeled experiment this is the total intensity of all the isotopic patterns in the label cluster.
Intensity 20160805_QE5_nLC7_MEM_COLLAB_JH_B4_01___1		Summed up eXtracted Ion Current (XIC) of all isotopic clusters associated with the identified AA sequence. In case of a labeled experiment this is the total intensity of all the isotopic patterns in the label cluster.
Intensity 20160805_QE5_nLC7_MEM_COLLAB_JH_B4_01___2		Summed up eXtracted Ion Current (XIC) of all isotopic clusters associated with the identified AA sequence. In case of a labeled experiment this is the total intensity of all the isotopic patterns in the label cluster.
Intensity 20160805_QE5_nLC7_MEM_COLLAB_JH_B4_01___3		Summed up eXtracted Ion Current (XIC) of all isotopic clusters associated with the identified AA sequence. In case of a labeled experiment this is the total intensity of all the isotopic patterns in the label cluster.
Intensity 20160805_QE5_nLC7_MEM_COLLAB_JH_B5_01___1		Summed up eXtracted Ion Current (XIC) of all isotopic clusters associated with the identified AA sequence. In case of a labeled experiment this is the total intensity of all the isotopic patterns in the label cluster.
Intensity 20160805_QE5_nLC7_MEM_COLLAB_JH_B5_01___2		Summed up eXtracted Ion Current (XIC) of all isotopic clusters associated with the identified AA sequence. In case of a labeled experiment this is the total intensity of all the isotopic patterns in the label cluster.
Intensity 20160805_QE5_nLC7_MEM_COLLAB_JH_B5_01___3		Summed up eXtracted Ion Current (XIC) of all isotopic clusters associated with the identified AA sequence. In case of a labeled experiment this is the total intensity of all the isotopic patterns in the label cluster.
Intensity 20160805_QE5_nLC7_MEM_COLLAB_JH_C1_01___1		Summed up eXtracted Ion Current (XIC) of all isotopic clusters associated with the identified AA sequence. In case of a labeled experiment this is the total intensity of all the isotopic patterns in the label cluster.
Intensity 20160805_QE5_nLC7_MEM_COLLAB_JH_C1_01___2		Summed up eXtracted Ion Current (XIC) of all isotopic clusters associated with the identified AA sequence. In case of a labeled experiment this is the total intensity of all the isotopic patterns in the label cluster.
Intensity 20160805_QE5_nLC7_MEM_COLLAB_JH_C1_01___3		Summed up eXtracted Ion Current (XIC) of all isotopic clusters associated with the identified AA sequence. In case of a labeled experiment this is the total intensity of all the isotopic patterns in the label cluster.

Intensity 20160805_QE5_nLC7_MEM_COLLAB_JH_C2_01___1		Summed up eXtracted Ion Current (XIC) of all isotopic clusters associated with the identified AA sequence. In case of a labeled experiment this is the total intensity of all the isotopic patterns in the label cluster.
Intensity 20160805_QE5_nLC7_MEM_COLLAB_JH_C2_01___2		Summed up eXtracted Ion Current (XIC) of all isotopic clusters associated with the identified AA sequence. In case of a labeled experiment this is the total intensity of all the isotopic patterns in the label cluster.
Intensity 20160805_QE5_nLC7_MEM_COLLAB_JH_C2_01___3		Summed up eXtracted Ion Current (XIC) of all isotopic clusters associated with the identified AA sequence. In case of a labeled experiment this is the total intensity of all the isotopic patterns in the label cluster.
Intensity 20160805_QE5_nLC7_MEM_COLLAB_JH_C3_01___1		Summed up eXtracted Ion Current (XIC) of all isotopic clusters associated with the identified AA sequence. In case of a labeled experiment this is the total intensity of all the isotopic patterns in the label cluster.
Intensity 20160805_QE5_nLC7_MEM_COLLAB_JH_C3_01___2		Summed up eXtracted Ion Current (XIC) of all isotopic clusters associated with the identified AA sequence. In case of a labeled experiment this is the total intensity of all the isotopic patterns in the label cluster.
Intensity 20160805_QE5_nLC7_MEM_COLLAB_JH_C3_01___3		Summed up eXtracted Ion Current (XIC) of all isotopic clusters associated with the identified AA sequence. In case of a labeled experiment this is the total intensity of all the isotopic patterns in the label cluster.
Intensity 20160805_QE5_nLC7_MEM_COLLAB_JH_C4_01___1		Summed up eXtracted Ion Current (XIC) of all isotopic clusters associated with the identified AA sequence. In case of a labeled experiment this is the total intensity of all the isotopic patterns in the label cluster.
Intensity 20160805_QE5_nLC7_MEM_COLLAB_JH_C4_01___2		Summed up eXtracted Ion Current (XIC) of all isotopic clusters associated with the identified AA sequence. In case of a labeled experiment this is the total intensity of all the isotopic patterns in the label cluster.
Intensity 20160805_QE5_nLC7_MEM_COLLAB_JH_C4_01___3		Summed up eXtracted Ion Current (XIC) of all isotopic clusters associated with the identified AA sequence. In case of a labeled experiment this is the total intensity of all the isotopic patterns in the label cluster.
Intensity 20160805_QE5_nLC7_MEM_COLLAB_JH_C5_01___1		Summed up eXtracted Ion Current (XIC) of all isotopic clusters associated with the identified AA sequence. In case of a labeled experiment this is the total intensity of all the isotopic patterns in the label cluster.
Intensity 20160805_QE5_nLC7_MEM_COLLAB_JH_C5_01___2		Summed up eXtracted Ion Current (XIC) of all isotopic clusters associated with the identified AA sequence. In case of a labeled experiment this is the total intensity of all the isotopic patterns in the label cluster.
Intensity 20160805_QE5_nLC7_MEM_COLLAB_JH_C5_01___3		Summed up eXtracted Ion Current (XIC) of all isotopic clusters associated with the identified AA sequence. In case of a labeled experiment this is the total intensity of all the isotopic patterns in the label cluster.
Intensity 20160805_QE5_nLC7_MEM_COLLAB_JH_C6_01___1		Summed up eXtracted Ion Current (XIC) of all isotopic clusters associated with the identified AA sequence. In case of a labeled experiment this is the total intensity of all the isotopic patterns in the label cluster.
Intensity 20160805_QE5_nLC7_MEM_COLLAB_JH_C6_01___2		Summed up eXtracted Ion Current (XIC) of all isotopic clusters associated with the identified AA sequence. In case of a labeled experiment this is the total intensity of all the isotopic patterns in the label cluster.
Intensity 20160805_QE5_nLC7_MEM_COLLAB_JH_C6_01___3		Summed up eXtracted Ion Current (XIC) of all isotopic clusters associated with the identified AA sequence. In case of a labeled experiment this is the total intensity of all the isotopic patterns in the label cluster.
Intensity 20160805_QE5_nLC7_MEM_COLLAB_JH_C7_01___1		Summed up eXtracted Ion Current (XIC) of all isotopic clusters associated with the identified AA sequence. In case of a labeled experiment this is the total intensity of all the isotopic patterns in the label cluster.
Intensity 20160805_QE5_nLC7_MEM_COLLAB_JH_C7_01___2		Summed up eXtracted Ion Current (XIC) of all isotopic clusters associated with the identified AA sequence. In case of a labeled experiment this is the total intensity of all the isotopic patterns in the label cluster.
Intensity 20160805_QE5_nLC7_MEM_COLLAB_JH_C7_01___3		Summed up eXtracted Ion Current (XIC) of all isotopic clusters associated with the identified AA sequence. In case of a labeled experiment this is the total intensity of all the isotopic patterns in the label cluster.
Intensity 20160805_QE5_nLC7_MEM_COLLAB_JH_C8_01___1		Summed up eXtracted Ion Current (XIC) of all isotopic clusters associated with the identified AA sequence. In case of a labeled experiment this is the total intensity of all the isotopic patterns in the label cluster.
Intensity 20160805_QE5_nLC7_MEM_COLLAB_JH_C8_01___2		Summed up eXtracted Ion Current (XIC) of all isotopic clusters associated with the identified AA sequence. In case of a labeled experiment this is the total intensity of all the isotopic patterns in the label cluster.
Intensity 20160805_QE5_nLC7_MEM_COLLAB_JH_C8_01___3		Summed up eXtracted Ion Current (XIC) of all isotopic clusters associated with the identified AA sequence. In case of a labeled experiment this is the total intensity of all the isotopic patterns in the label cluster.

Intensity FL0145_JHendy_130416_MSQ525_CW10___1		Summed up eXtracted Ion Current (XIC) of all isotopic clusters associated with the identified AA sequence. In case of a labeled experiment this is the total intensity of all the isotopic patterns in the label cluster.
Intensity FL0145_JHendy_130416_MSQ525_CW10___2		Summed up eXtracted Ion Current (XIC) of all isotopic clusters associated with the identified AA sequence. In case of a labeled experiment this is the total intensity of all the isotopic patterns in the label cluster.
Intensity FL0145_JHendy_130416_MSQ525_CW10___3		Summed up eXtracted Ion Current (XIC) of all isotopic clusters associated with the identified AA sequence. In case of a labeled experiment this is the total intensity of all the isotopic patterns in the label cluster.
Intensity FL0145_JHendy_130416_MSQ525_CW11___1		Summed up eXtracted Ion Current (XIC) of all isotopic clusters associated with the identified AA sequence. In case of a labeled experiment this is the total intensity of all the isotopic patterns in the label cluster.
Intensity FL0145_JHendy_130416_MSQ525_CW11___2		Summed up eXtracted Ion Current (XIC) of all isotopic clusters associated with the identified AA sequence. In case of a labeled experiment this is the total intensity of all the isotopic patterns in the label cluster.
Intensity FL0145_JHendy_130416_MSQ525_CW11___3		Summed up eXtracted Ion Current (XIC) of all isotopic clusters associated with the identified AA sequence. In case of a labeled experiment this is the total intensity of all the isotopic patterns in the label cluster.
Intensity FL0145_JHendy_130416_MSQ525_CW18___1		Summed up eXtracted Ion Current (XIC) of all isotopic clusters associated with the identified AA sequence. In case of a labeled experiment this is the total intensity of all the isotopic patterns in the label cluster.
Intensity FL0145_JHendy_130416_MSQ525_CW18___2		Summed up eXtracted Ion Current (XIC) of all isotopic clusters associated with the identified AA sequence. In case of a labeled experiment this is the total intensity of all the isotopic patterns in the label cluster.
Intensity FL0145_JHendy_130416_MSQ525_CW18___3		Summed up eXtracted Ion Current (XIC) of all isotopic clusters associated with the identified AA sequence. In case of a labeled experiment this is the total intensity of all the isotopic patterns in the label cluster.
Intensity FL0145_JHendy_130416_MSQ525_CW20___1		Summed up eXtracted Ion Current (XIC) of all isotopic clusters associated with the identified AA sequence. In case of a labeled experiment this is the total intensity of all the isotopic patterns in the label cluster.
Intensity FL0145_JHendy_130416_MSQ525_CW20___2		Summed up eXtracted Ion Current (XIC) of all isotopic clusters associated with the identified AA sequence. In case of a labeled experiment this is the total intensity of all the isotopic patterns in the label cluster.
Intensity FL0145_JHendy_130416_MSQ525_CW20___3		Summed up eXtracted Ion Current (XIC) of all isotopic clusters associated with the identified AA sequence. In case of a labeled experiment this is the total intensity of all the isotopic patterns in the label cluster.
Intensity FL0145_JHendy_130416_MSQ525_CW21___1		Summed up eXtracted Ion Current (XIC) of all isotopic clusters associated with the identified AA sequence. In case of a labeled experiment this is the total intensity of all the isotopic patterns in the label cluster.
Intensity FL0145_JHendy_130416_MSQ525_CW21___2		Summed up eXtracted Ion Current (XIC) of all isotopic clusters associated with the identified AA sequence. In case of a labeled experiment this is the total intensity of all the isotopic patterns in the label cluster.
Intensity FL0145_JHendy_130416_MSQ525_CW21___3		Summed up eXtracted Ion Current (XIC) of all isotopic clusters associated with the identified AA sequence. In case of a labeled experiment this is the total intensity of all the isotopic patterns in the label cluster.
Intensity FL0145_JHendy_130416_MSQ525_CW23___1		Summed up eXtracted Ion Current (XIC) of all isotopic clusters associated with the identified AA sequence. In case of a labeled experiment this is the total intensity of all the isotopic patterns in the label cluster.
Intensity FL0145_JHendy_130416_MSQ525_CW23___2		Summed up eXtracted Ion Current (XIC) of all isotopic clusters associated with the identified AA sequence. In case of a labeled experiment this is the total intensity of all the isotopic patterns in the label cluster.
Intensity FL0145_JHendy_130416_MSQ525_CW23___3		Summed up eXtracted Ion Current (XIC) of all isotopic clusters associated with the identified AA sequence. In case of a labeled experiment this is the total intensity of all the isotopic patterns in the label cluster.
Intensity FL0145_JHendy_130416_MSQ525_CW8_160425165036___1		Summed up eXtracted Ion Current (XIC) of all isotopic clusters associated with the identified AA sequence. In case of a labeled experiment this is the total intensity of all the isotopic patterns in the label cluster.
Intensity FL0145_JHendy_130416_MSQ525_CW8_160425165036___2		Summed up eXtracted Ion Current (XIC) of all isotopic clusters associated with the identified AA sequence. In case of a labeled experiment this is the total intensity of all the isotopic patterns in the label cluster.
Intensity FL0145_JHendy_130416_MSQ525_CW8_160425165036___3		Summed up eXtracted Ion Current (XIC) of all isotopic clusters associated with the identified AA sequence. In case of a labeled experiment this is the total intensity of all the isotopic patterns in the label cluster.

Intensity FL0168_JHendy_MSQ525_170516_BK___1		Summed up eXtracted Ion Current (XIC) of all isotopic clusters associated with the identified AA sequence. In case of a labeled experiment this is the total intensity of all the isotopic patterns in the label cluster.
Intensity FL0168_JHendy_MSQ525_170516_BK___2		Summed up eXtracted Ion Current (XIC) of all isotopic clusters associated with the identified AA sequence. In case of a labeled experiment this is the total intensity of all the isotopic patterns in the label cluster.
Intensity FL0168_JHendy_MSQ525_170516_BK___3		Summed up eXtracted Ion Current (XIC) of all isotopic clusters associated with the identified AA sequence. In case of a labeled experiment this is the total intensity of all the isotopic patterns in the label cluster.
Intensity FL0168_JHendy_MSQ525_170516_CW1___1		Summed up eXtracted Ion Current (XIC) of all isotopic clusters associated with the identified AA sequence. In case of a labeled experiment this is the total intensity of all the isotopic patterns in the label cluster.
Intensity FL0168_JHendy_MSQ525_170516_CW1___2		Summed up eXtracted Ion Current (XIC) of all isotopic clusters associated with the identified AA sequence. In case of a labeled experiment this is the total intensity of all the isotopic patterns in the label cluster.
Intensity FL0168_JHendy_MSQ525_170516_CW1___3		Summed up eXtracted Ion Current (XIC) of all isotopic clusters associated with the identified AA sequence. In case of a labeled experiment this is the total intensity of all the isotopic patterns in the label cluster.
Intensity FL0168_JHendy_MSQ525_170516_CW10___1		Summed up eXtracted Ion Current (XIC) of all isotopic clusters associated with the identified AA sequence. In case of a labeled experiment this is the total intensity of all the isotopic patterns in the label cluster.
Intensity FL0168_JHendy_MSQ525_170516_CW10___2		Summed up eXtracted Ion Current (XIC) of all isotopic clusters associated with the identified AA sequence. In case of a labeled experiment this is the total intensity of all the isotopic patterns in the label cluster.
Intensity FL0168_JHendy_MSQ525_170516_CW10___3		Summed up eXtracted Ion Current (XIC) of all isotopic clusters associated with the identified AA sequence. In case of a labeled experiment this is the total intensity of all the isotopic patterns in the label cluster.
Intensity FL0168_JHendy_MSQ525_170516_CW2___1		Summed up eXtracted Ion Current (XIC) of all isotopic clusters associated with the identified AA sequence. In case of a labeled experiment this is the total intensity of all the isotopic patterns in the label cluster.
Intensity FL0168_JHendy_MSQ525_170516_CW2___2		Summed up eXtracted Ion Current (XIC) of all isotopic clusters associated with the identified AA sequence. In case of a labeled experiment this is the total intensity of all the isotopic patterns in the label cluster.
Intensity FL0168_JHendy_MSQ525_170516_CW2___3		Summed up eXtracted Ion Current (XIC) of all isotopic clusters associated with the identified AA sequence. In case of a labeled experiment this is the total intensity of all the isotopic patterns in the label cluster.
Intensity FL0168_JHendy_MSQ525_170516_CW3___1		Summed up eXtracted Ion Current (XIC) of all isotopic clusters associated with the identified AA sequence. In case of a labeled experiment this is the total intensity of all the isotopic patterns in the label cluster.
Intensity FL0168_JHendy_MSQ525_170516_CW3___2		Summed up eXtracted Ion Current (XIC) of all isotopic clusters associated with the identified AA sequence. In case of a labeled experiment this is the total intensity of all the isotopic patterns in the label cluster.
Intensity FL0168_JHendy_MSQ525_170516_CW3___3		Summed up eXtracted Ion Current (XIC) of all isotopic clusters associated with the identified AA sequence. In case of a labeled experiment this is the total intensity of all the isotopic patterns in the label cluster.
Intensity FL0168_JHendy_MSQ525_170516_CW4___1		Summed up eXtracted Ion Current (XIC) of all isotopic clusters associated with the identified AA sequence. In case of a labeled experiment this is the total intensity of all the isotopic patterns in the label cluster.
Intensity FL0168_JHendy_MSQ525_170516_CW4___2		Summed up eXtracted Ion Current (XIC) of all isotopic clusters associated with the identified AA sequence. In case of a labeled experiment this is the total intensity of all the isotopic patterns in the label cluster.
Intensity FL0168_JHendy_MSQ525_170516_CW4___3		Summed up eXtracted Ion Current (XIC) of all isotopic clusters associated with the identified AA sequence. In case of a labeled experiment this is the total intensity of all the isotopic patterns in the label cluster.
Intensity FL0168_JHendy_MSQ525_170516_CW5___1		Summed up eXtracted Ion Current (XIC) of all isotopic clusters associated with the identified AA sequence. In case of a labeled experiment this is the total intensity of all the isotopic patterns in the label cluster.
Intensity FL0168_JHendy_MSQ525_170516_CW5___2		Summed up eXtracted Ion Current (XIC) of all isotopic clusters associated with the identified AA sequence. In case of a labeled experiment this is the total intensity of all the isotopic patterns in the label cluster.
Intensity FL0168_JHendy_MSQ525_170516_CW5___3		Summed up eXtracted Ion Current (XIC) of all isotopic clusters associated with the identified AA sequence. In case of a labeled experiment this is the total intensity of all the isotopic patterns in the label cluster.

Intensity FL0168_JHendy_MSQ525_170516_CW6___1		Summed up eXtracted Ion Current (XIC) of all isotopic clusters associated with the identified AA sequence. In case of a labeled experiment this is the total intensity of all the isotopic patterns in the label cluster.
Intensity FL0168_JHendy_MSQ525_170516_CW6___2		Summed up eXtracted Ion Current (XIC) of all isotopic clusters associated with the identified AA sequence. In case of a labeled experiment this is the total intensity of all the isotopic patterns in the label cluster.
Intensity FL0168_JHendy_MSQ525_170516_CW6___3		Summed up eXtracted Ion Current (XIC) of all isotopic clusters associated with the identified AA sequence. In case of a labeled experiment this is the total intensity of all the isotopic patterns in the label cluster.
Intensity FL0168_JHendy_MSQ525_170516_CW7___1		Summed up eXtracted Ion Current (XIC) of all isotopic clusters associated with the identified AA sequence. In case of a labeled experiment this is the total intensity of all the isotopic patterns in the label cluster.
Intensity FL0168_JHendy_MSQ525_170516_CW7___2		Summed up eXtracted Ion Current (XIC) of all isotopic clusters associated with the identified AA sequence. In case of a labeled experiment this is the total intensity of all the isotopic patterns in the label cluster.
Intensity FL0168_JHendy_MSQ525_170516_CW7___3		Summed up eXtracted Ion Current (XIC) of all isotopic clusters associated with the identified AA sequence. In case of a labeled experiment this is the total intensity of all the isotopic patterns in the label cluster.
Intensity FL0168_JHendy_MSQ525_170516_CW8___1		Summed up eXtracted Ion Current (XIC) of all isotopic clusters associated with the identified AA sequence. In case of a labeled experiment this is the total intensity of all the isotopic patterns in the label cluster.
Intensity FL0168_JHendy_MSQ525_170516_CW8___2		Summed up eXtracted Ion Current (XIC) of all isotopic clusters associated with the identified AA sequence. In case of a labeled experiment this is the total intensity of all the isotopic patterns in the label cluster.
Intensity FL0168_JHendy_MSQ525_170516_CW8___3		Summed up eXtracted Ion Current (XIC) of all isotopic clusters associated with the identified AA sequence. In case of a labeled experiment this is the total intensity of all the isotopic patterns in the label cluster.
Intensity FL0168_JHendy_MSQ525_170516_CW9___1		Summed up eXtracted Ion Current (XIC) of all isotopic clusters associated with the identified AA sequence. In case of a labeled experiment this is the total intensity of all the isotopic patterns in the label cluster.
Intensity FL0168_JHendy_MSQ525_170516_CW9___2		Summed up eXtracted Ion Current (XIC) of all isotopic clusters associated with the identified AA sequence. In case of a labeled experiment this is the total intensity of all the isotopic patterns in the label cluster.
Intensity FL0168_JHendy_MSQ525_170516_CW9___3		Summed up eXtracted Ion Current (XIC) of all isotopic clusters associated with the identified AA sequence. In case of a labeled experiment this is the total intensity of all the isotopic patterns in the label cluster.
Reverse		When marked with '+', this particular peptide was found to be part of a protein derived from the reversed part of the protein sequence database. These should be removed for further data analysis.
Potential contaminant		When marked with '+', this particular peptide was found to be part of a commonly occurring contaminant. These should be removed for further data analysis.
id		A unique (consecutive) identifier for each row in the site table, which is used to cross-link the information in this file with the information stored in the other files.
Protein group IDs		The identifier of the protein-group this peptide sequence is associated with, which can be used to look up the extended protein information in the file 'proteinGroups.txt'.<br>As a single peptide can be linked to multiple proteins (e.g. in the case of razor-proteins), multiple id's can be stored here separated by a semicolon.<br>As a protein can be identified by multiple peptides, the same id can be found in different rows.
Positions		The positions of the modifications in the protein amino acid sequence.
Position		The position of the modification in the protein amino acid sequence.
Peptide IDs		Identifier(s) of the associated peptide sequence(s) summary, which can be found in the file 'peptides.txt'.
Mod. peptide IDs		Identifier(s) of the associated peptide sequence(s) summary, which can be found in the file 'modificationSpecificPeptides.txt'.
Evidence IDs		Identifier(s) for analyzed peptide evidence associated with the protein group referenced against the evidence table.
MS/MS IDs		The identifiers of the MS/MS scans identifying this peptide, referenced against the msms table.
Best localization evidence ID		
Best localization MS/MS ID		
Best localization raw file		
Best localization scan number		

Best score evidence ID		
Best score MS/MS ID		
Best score raw file		
Best score scan number		
Best PEP evidence ID		
Best PEP MS/MS ID		
Best PEP raw file		
Best PEP scan number		

Propionamide KSites

Name	Separator	Description
Proteins		Identifiers of proteins this site is associated with.
Positions within proteins		For each protein identifier in the 'Proteins' column you find here the position of the site in the respective protein sequence. The index of the first amino acid in the sequence is 1.
Leading proteins		
Protein		Identifier of the protein this peptide is associated with.
Fasta headers		Descriptions of proteins this peptide is associated with.
Localization prob		
Score diff		
PEP		The posterior error probability (PEP) of the best identified modified peptide containing this site.
Score		The Andromeda score of the best identified modified peptide containing this site.
Delta score		The Andromeda delta score of the best identified modified peptide containing this site.
Score for localization		The Andromeda score of the MS/MS spectrum used for calculating the localization score for this site.
Localization prob 20160805_QE5_nLC7_MEM_COLLAB_JH_A1_01		
Score diff 20160805_QE5_nLC7_MEM_COLLAB_JH_A1_01		
PEP 20160805_QE5_nLC7_MEM_COLLAB_JH_A1_01		
Score 20160805_QE5_nLC7_MEM_COLLAB_JH_A1_01		
Localization prob 20160805_QE5_nLC7_MEM_COLLAB_JH_A2_01		
Score diff 20160805_QE5_nLC7_MEM_COLLAB_JH_A2_01		
PEP 20160805_QE5_nLC7_MEM_COLLAB_JH_A2_01		
Score 20160805_QE5_nLC7_MEM_COLLAB_JH_A2_01		
Localization prob 20160805_QE5_nLC7_MEM_COLLAB_JH_A3_01		
Score diff 20160805_QE5_nLC7_MEM_COLLAB_JH_A3_01		
PEP 20160805_QE5_nLC7_MEM_COLLAB_JH_A3_01		
Score 20160805_QE5_nLC7_MEM_COLLAB_JH_A3_01		
Localization prob 20160805_QE5_nLC7_MEM_COLLAB_JH_B1_01		
Score diff 20160805_QE5_nLC7_MEM_COLLAB_JH_B1_01		
PEP 20160805_QE5_nLC7_MEM_COLLAB_JH_B1_01		
Score 20160805_QE5_nLC7_MEM_COLLAB_JH_B1_01		
Localization prob 20160805_QE5_nLC7_MEM_COLLAB_JH_B2_01		
Score diff 20160805_QE5_nLC7_MEM_COLLAB_JH_B2_01		

PEP 20160805_QE5_nLC7_MEM_COLLAB_JH_B2_01		
Score 20160805_QE5_nLC7_MEM_COLLAB_JH_B2_01		
Localization prob 20160805_QE5_nLC7_MEM_COLLAB_JH_B3_01		
Score diff 20160805_QE5_nLC7_MEM_COLLAB_JH_B3_01		
PEP 20160805_QE5_nLC7_MEM_COLLAB_JH_B3_01		
Score 20160805_QE5_nLC7_MEM_COLLAB_JH_B3_01		
Localization prob 20160805_QE5_nLC7_MEM_COLLAB_JH_B4_01		
Score diff 20160805_QE5_nLC7_MEM_COLLAB_JH_B4_01		
PEP 20160805_QE5_nLC7_MEM_COLLAB_JH_B4_01		
Score 20160805_QE5_nLC7_MEM_COLLAB_JH_B4_01		
Localization prob 20160805_QE5_nLC7_MEM_COLLAB_JH_B5_01		
Score diff 20160805_QE5_nLC7_MEM_COLLAB_JH_B5_01		
PEP 20160805_QE5_nLC7_MEM_COLLAB_JH_B5_01		
Score 20160805_QE5_nLC7_MEM_COLLAB_JH_B5_01		
Localization prob 20160805_QE5_nLC7_MEM_COLLAB_JH_C1_01		
Score diff 20160805_QE5_nLC7_MEM_COLLAB_JH_C1_01		
PEP 20160805_QE5_nLC7_MEM_COLLAB_JH_C1_01		
Score 20160805_QE5_nLC7_MEM_COLLAB_JH_C1_01		
Localization prob 20160805_QE5_nLC7_MEM_COLLAB_JH_C2_01		
Score diff 20160805_QE5_nLC7_MEM_COLLAB_JH_C2_01		
PEP 20160805_QE5_nLC7_MEM_COLLAB_JH_C2_01		
Score 20160805_QE5_nLC7_MEM_COLLAB_JH_C2_01		
Localization prob 20160805_QE5_nLC7_MEM_COLLAB_JH_C3_01		
Score diff 20160805_QE5_nLC7_MEM_COLLAB_JH_C3_01		
PEP 20160805_QE5_nLC7_MEM_COLLAB_JH_C3_01		
Score 20160805_QE5_nLC7_MEM_COLLAB_JH_C3_01		
Localization prob 20160805_QE5_nLC7_MEM_COLLAB_JH_C4_01		

Score diff 20160805_QE5_nLC7_MEM_COLLAB_JH_C4_01		
PEP 20160805_QE5_nLC7_MEM_COLLAB_JH_C4_01		
Score 20160805_QE5_nLC7_MEM_COLLAB_JH_C4_01		
Localization prob 20160805_QE5_nLC7_MEM_COLLAB_JH_C5_01		
Score diff 20160805_QE5_nLC7_MEM_COLLAB_JH_C5_01		
PEP 20160805_QE5_nLC7_MEM_COLLAB_JH_C5_01		
Score 20160805_QE5_nLC7_MEM_COLLAB_JH_C5_01		
Localization prob 20160805_QE5_nLC7_MEM_COLLAB_JH_C6_01		
Score diff 20160805_QE5_nLC7_MEM_COLLAB_JH_C6_01		
PEP 20160805_QE5_nLC7_MEM_COLLAB_JH_C6_01		
Score 20160805_QE5_nLC7_MEM_COLLAB_JH_C6_01		
Localization prob 20160805_QE5_nLC7_MEM_COLLAB_JH_C7_01		
Score diff 20160805_QE5_nLC7_MEM_COLLAB_JH_C7_01		
PEP 20160805_QE5_nLC7_MEM_COLLAB_JH_C7_01		
Score 20160805_QE5_nLC7_MEM_COLLAB_JH_C7_01		
Localization prob 20160805_QE5_nLC7_MEM_COLLAB_JH_C8_01		
Score diff 20160805_QE5_nLC7_MEM_COLLAB_JH_C8_01		
PEP 20160805_QE5_nLC7_MEM_COLLAB_JH_C8_01		
Score 20160805_QE5_nLC7_MEM_COLLAB_JH_C8_01		
Localization prob FL0145_JHendy_130416_MSQ525_CW10		
Score diff FL0145_JHendy_130416_MSQ525_CW10		
PEP FL0145_JHendy_130416_MSQ525_CW10		
Score FL0145_JHendy_130416_MSQ525_CW10		
Localization prob FL0145_JHendy_130416_MSQ525_CW11		
Score diff FL0145_JHendy_130416_MSQ525_CW11		
PEP FL0145_JHendy_130416_MSQ525_CW11		
Score FL0145_JHendy_130416_MSQ525_CW11		

Localization prob FL0145_JHendy_130416_MSQ525_CW18		
Score diff FL0145_JHendy_130416_MSQ525_CW18		
PEP FL0145_JHendy_130416_MSQ525_CW18		
Score FL0145_JHendy_130416_MSQ525_CW18		
Localization prob FL0145_JHendy_130416_MSQ525_CW20		
Score diff FL0145_JHendy_130416_MSQ525_CW20		
PEP FL0145_JHendy_130416_MSQ525_CW20		
Score FL0145_JHendy_130416_MSQ525_CW20		
Localization prob FL0145_JHendy_130416_MSQ525_CW21		
Score diff FL0145_JHendy_130416_MSQ525_CW21		
PEP FL0145_JHendy_130416_MSQ525_CW21		
Score FL0145_JHendy_130416_MSQ525_CW21		
Localization prob FL0145_JHendy_130416_MSQ525_CW23		
Score diff FL0145_JHendy_130416_MSQ525_CW23		
PEP FL0145_JHendy_130416_MSQ525_CW23		
Score FL0145_JHendy_130416_MSQ525_CW23		
Localization prob FL0145_JHendy_130416_MSQ525_CW8_160425165036		
Score diff FL0145_JHendy_130416_MSQ525_CW8_160425165036		
PEP FL0145_JHendy_130416_MSQ525_CW8_160425165036		
Score FL0145_JHendy_130416_MSQ525_CW8_160425165036		
Localization prob FL0168_JHendy_MSQ525_170516_BK		
Score diff FL0168_JHendy_MSQ525_170516_BK		
PEP FL0168_JHendy_MSQ525_170516_BK		
Score FL0168_JHendy_MSQ525_170516_BK		
Localization prob FL0168_JHendy_MSQ525_170516_CW1		
Score diff FL0168_JHendy_MSQ525_170516_CW1		
PEP FL0168_JHendy_MSQ525_170516_CW1		

Score FL0168_JHendy_MSQ525_170516_CW1		
Localization prob FL0168_JHendy_MSQ525_170516_CW10		
Score diff FL0168_JHendy_MSQ525_170516_CW10		
PEP FL0168_JHendy_MSQ525_170516_CW10		
Score FL0168_JHendy_MSQ525_170516_CW10		
Localization prob FL0168_JHendy_MSQ525_170516_CW2		
Score diff FL0168_JHendy_MSQ525_170516_CW2		
PEP FL0168_JHendy_MSQ525_170516_CW2		
Score FL0168_JHendy_MSQ525_170516_CW2		
Localization prob FL0168_JHendy_MSQ525_170516_CW3		
Score diff FL0168_JHendy_MSQ525_170516_CW3		
PEP FL0168_JHendy_MSQ525_170516_CW3		
Score FL0168_JHendy_MSQ525_170516_CW3		
Localization prob FL0168_JHendy_MSQ525_170516_CW4		
Score diff FL0168_JHendy_MSQ525_170516_CW4		
PEP FL0168_JHendy_MSQ525_170516_CW4		
Score FL0168_JHendy_MSQ525_170516_CW4		
Localization prob FL0168_JHendy_MSQ525_170516_CW5		
Score diff FL0168_JHendy_MSQ525_170516_CW5		
PEP FL0168_JHendy_MSQ525_170516_CW5		
Score FL0168_JHendy_MSQ525_170516_CW5		
Localization prob FL0168_JHendy_MSQ525_170516_CW6		
Score diff FL0168_JHendy_MSQ525_170516_CW6		
PEP FL0168_JHendy_MSQ525_170516_CW6		
Score FL0168_JHendy_MSQ525_170516_CW6		
Localization prob FL0168_JHendy_MSQ525_170516_CW7		
Score diff FL0168_JHendy_MSQ525_170516_CW7		

PEP FL0168_JHendy_MSQ525_170516_CW7		
Score FL0168_JHendy_MSQ525_170516_CW7		
Localization prob FL0168_JHendy_MSQ525_170516_CW8		
Score diff FL0168_JHendy_MSQ525_170516_CW8		
PEP FL0168_JHendy_MSQ525_170516_CW8		
Score FL0168_JHendy_MSQ525_170516_CW8		
Localization prob FL0168_JHendy_MSQ525_170516_CW9		
Score diff FL0168_JHendy_MSQ525_170516_CW9		
PEP FL0168_JHendy_MSQ525_170516_CW9		
Score FL0168_JHendy_MSQ525_170516_CW9		
Diagnostic peak		
Number of Propionamide K		Different numbers of Propionamide K on peptides that this site is involved in.
Amino acid		
Sequence window		
Modification window		
Peptide window coverage		
Propionamide K Probabilities		
Propionamide K Score diffs		
Position in peptide		
Charge		Charge state of the precursor ion.
Mass error [ppm]		Mass error of the recalibrated mass-over-charge value of the precursor ion in comparison to the predicted monoisotopic mass of the identified peptide sequence.
Intensity		Summed up eXtracted Ion Current (XIC) of all isotopic clusters associated with the identified AA sequence. In case of a labeled experiment this is the total intensity of all the isotopic patterns in the label cluster.
Intensity___1		Summed up eXtracted Ion Current (XIC) of all isotopic clusters associated with the identified AA sequence. In case of a labeled experiment this is the total intensity of all the isotopic patterns in the label cluster.
Intensity___2		Summed up eXtracted Ion Current (XIC) of all isotopic clusters associated with the identified AA sequence. In case of a labeled experiment this is the total intensity of all the isotopic patterns in the label cluster.
Intensity___3		Summed up eXtracted Ion Current (XIC) of all isotopic clusters associated with the identified AA sequence. In case of a labeled experiment this is the total intensity of all the isotopic patterns in the label cluster.
Ratio mod/base		
Intensity 20160805_QE5_nLC7_MEM_COLLAB_JH_A1_01		Summed up eXtracted Ion Current (XIC) of all isotopic clusters associated with the identified AA sequence. In case of a labeled experiment this is the total intensity of all the isotopic patterns in the label cluster.
Intensity 20160805_QE5_nLC7_MEM_COLLAB_JH_A2_01		Summed up eXtracted Ion Current (XIC) of all isotopic clusters associated with the identified AA sequence. In case of a labeled experiment this is the total intensity of all the isotopic patterns in the label cluster.
Intensity 20160805_QE5_nLC7_MEM_COLLAB_JH_A3_01		Summed up eXtracted Ion Current (XIC) of all isotopic clusters associated with the identified AA sequence. In case of a labeled experiment this is the total intensity of all the isotopic patterns in the label cluster.
Intensity 20160805_QE5_nLC7_MEM_COLLAB_JH_B1_01		Summed up eXtracted Ion Current (XIC) of all isotopic clusters associated with the identified AA sequence. In case of a labeled experiment this is the total intensity of all the isotopic patterns in the label cluster.

Intensity 20160805_QE5_nLC7_MEM_COLLAB_JH_B2_01		Summed up eXtracted Ion Current (XIC) of all isotopic clusters associated with the identified AA sequence. In case of a labeled experiment this is the total intensity of all the isotopic patterns in the label cluster.
Intensity 20160805_QE5_nLC7_MEM_COLLAB_JH_B3_01		Summed up eXtracted Ion Current (XIC) of all isotopic clusters associated with the identified AA sequence. In case of a labeled experiment this is the total intensity of all the isotopic patterns in the label cluster.
Intensity 20160805_QE5_nLC7_MEM_COLLAB_JH_B4_01		Summed up eXtracted Ion Current (XIC) of all isotopic clusters associated with the identified AA sequence. In case of a labeled experiment this is the total intensity of all the isotopic patterns in the label cluster.
Intensity 20160805_QE5_nLC7_MEM_COLLAB_JH_B5_01		Summed up eXtracted Ion Current (XIC) of all isotopic clusters associated with the identified AA sequence. In case of a labeled experiment this is the total intensity of all the isotopic patterns in the label cluster.
Intensity 20160805_QE5_nLC7_MEM_COLLAB_JH_C1_01		Summed up eXtracted Ion Current (XIC) of all isotopic clusters associated with the identified AA sequence. In case of a labeled experiment this is the total intensity of all the isotopic patterns in the label cluster.
Intensity 20160805_QE5_nLC7_MEM_COLLAB_JH_C2_01		Summed up eXtracted Ion Current (XIC) of all isotopic clusters associated with the identified AA sequence. In case of a labeled experiment this is the total intensity of all the isotopic patterns in the label cluster.
Intensity 20160805_QE5_nLC7_MEM_COLLAB_JH_C3_01		Summed up eXtracted Ion Current (XIC) of all isotopic clusters associated with the identified AA sequence. In case of a labeled experiment this is the total intensity of all the isotopic patterns in the label cluster.
Intensity 20160805_QE5_nLC7_MEM_COLLAB_JH_C4_01		Summed up eXtracted Ion Current (XIC) of all isotopic clusters associated with the identified AA sequence. In case of a labeled experiment this is the total intensity of all the isotopic patterns in the label cluster.
Intensity 20160805_QE5_nLC7_MEM_COLLAB_JH_C5_01		Summed up eXtracted Ion Current (XIC) of all isotopic clusters associated with the identified AA sequence. In case of a labeled experiment this is the total intensity of all the isotopic patterns in the label cluster.
Intensity 20160805_QE5_nLC7_MEM_COLLAB_JH_C6_01		Summed up eXtracted Ion Current (XIC) of all isotopic clusters associated with the identified AA sequence. In case of a labeled experiment this is the total intensity of all the isotopic patterns in the label cluster.
Intensity 20160805_QE5_nLC7_MEM_COLLAB_JH_C7_01		Summed up eXtracted Ion Current (XIC) of all isotopic clusters associated with the identified AA sequence. In case of a labeled experiment this is the total intensity of all the isotopic patterns in the label cluster.
Intensity 20160805_QE5_nLC7_MEM_COLLAB_JH_C8_01		Summed up eXtracted Ion Current (XIC) of all isotopic clusters associated with the identified AA sequence. In case of a labeled experiment this is the total intensity of all the isotopic patterns in the label cluster.
Intensity FL0145_JHendy_130416_MSQ525_CW10		Summed up eXtracted Ion Current (XIC) of all isotopic clusters associated with the identified AA sequence. In case of a labeled experiment this is the total intensity of all the isotopic patterns in the label cluster.
Intensity FL0145_JHendy_130416_MSQ525_CW11		Summed up eXtracted Ion Current (XIC) of all isotopic clusters associated with the identified AA sequence. In case of a labeled experiment this is the total intensity of all the isotopic patterns in the label cluster.
Intensity FL0145_JHendy_130416_MSQ525_CW18		Summed up eXtracted Ion Current (XIC) of all isotopic clusters associated with the identified AA sequence. In case of a labeled experiment this is the total intensity of all the isotopic patterns in the label cluster.
Intensity FL0145_JHendy_130416_MSQ525_CW20		Summed up eXtracted Ion Current (XIC) of all isotopic clusters associated with the identified AA sequence. In case of a labeled experiment this is the total intensity of all the isotopic patterns in the label cluster.
Intensity FL0145_JHendy_130416_MSQ525_CW21		Summed up eXtracted Ion Current (XIC) of all isotopic clusters associated with the identified AA sequence. In case of a labeled experiment this is the total intensity of all the isotopic patterns in the label cluster.
Intensity FL0145_JHendy_130416_MSQ525_CW23		Summed up eXtracted Ion Current (XIC) of all isotopic clusters associated with the identified AA sequence. In case of a labeled experiment this is the total intensity of all the isotopic patterns in the label cluster.
Intensity FL0145_JHendy_130416_MSQ525_CW8_160425165036		Summed up eXtracted Ion Current (XIC) of all isotopic clusters associated with the identified AA sequence. In case of a labeled experiment this is the total intensity of all the isotopic patterns in the label cluster.
Intensity FL0168_JHendy_MSQ525_170516_BK		Summed up eXtracted Ion Current (XIC) of all isotopic clusters associated with the identified AA sequence. In case of a labeled experiment this is the total intensity of all the isotopic patterns in the label cluster.
Intensity FL0168_JHendy_MSQ525_170516_CW1		Summed up eXtracted Ion Current (XIC) of all isotopic clusters associated with the identified AA sequence. In case of a labeled experiment this is the total intensity of all the isotopic patterns in the label cluster.

Intensity FL0168_JHendy_MSQ525_170516_CW10		Summed up eXtracted Ion Current (XIC) of all isotopic clusters associated with the identified AA sequence. In case of a labeled experiment this is the total intensity of all the isotopic patterns in the label cluster.
Intensity FL0168_JHendy_MSQ525_170516_CW2		Summed up eXtracted Ion Current (XIC) of all isotopic clusters associated with the identified AA sequence. In case of a labeled experiment this is the total intensity of all the isotopic patterns in the label cluster.
Intensity FL0168_JHendy_MSQ525_170516_CW3		Summed up eXtracted Ion Current (XIC) of all isotopic clusters associated with the identified AA sequence. In case of a labeled experiment this is the total intensity of all the isotopic patterns in the label cluster.
Intensity FL0168_JHendy_MSQ525_170516_CW4		Summed up eXtracted Ion Current (XIC) of all isotopic clusters associated with the identified AA sequence. In case of a labeled experiment this is the total intensity of all the isotopic patterns in the label cluster.
Intensity FL0168_JHendy_MSQ525_170516_CW5		Summed up eXtracted Ion Current (XIC) of all isotopic clusters associated with the identified AA sequence. In case of a labeled experiment this is the total intensity of all the isotopic patterns in the label cluster.
Intensity FL0168_JHendy_MSQ525_170516_CW6		Summed up eXtracted Ion Current (XIC) of all isotopic clusters associated with the identified AA sequence. In case of a labeled experiment this is the total intensity of all the isotopic patterns in the label cluster.
Intensity FL0168_JHendy_MSQ525_170516_CW7		Summed up eXtracted Ion Current (XIC) of all isotopic clusters associated with the identified AA sequence. In case of a labeled experiment this is the total intensity of all the isotopic patterns in the label cluster.
Intensity FL0168_JHendy_MSQ525_170516_CW8		Summed up eXtracted Ion Current (XIC) of all isotopic clusters associated with the identified AA sequence. In case of a labeled experiment this is the total intensity of all the isotopic patterns in the label cluster.
Intensity FL0168_JHendy_MSQ525_170516_CW9		Summed up eXtracted Ion Current (XIC) of all isotopic clusters associated with the identified AA sequence. In case of a labeled experiment this is the total intensity of all the isotopic patterns in the label cluster.
Ratio mod/base 20160805_QE5_nLC7_MEM_COLLAB_JH_A1_01		
Ratio mod/base 20160805_QE5_nLC7_MEM_COLLAB_JH_A2_01		
Ratio mod/base 20160805_QE5_nLC7_MEM_COLLAB_JH_A3_01		
Ratio mod/base 20160805_QE5_nLC7_MEM_COLLAB_JH_B1_01		
Ratio mod/base 20160805_QE5_nLC7_MEM_COLLAB_JH_B2_01		
Ratio mod/base 20160805_QE5_nLC7_MEM_COLLAB_JH_B3_01		
Ratio mod/base 20160805_QE5_nLC7_MEM_COLLAB_JH_B4_01		
Ratio mod/base 20160805_QE5_nLC7_MEM_COLLAB_JH_B5_01		
Ratio mod/base 20160805_QE5_nLC7_MEM_COLLAB_JH_C1_01		
Ratio mod/base 20160805_QE5_nLC7_MEM_COLLAB_JH_C2_01		
Ratio mod/base 20160805_QE5_nLC7_MEM_COLLAB_JH_C3_01		
Ratio mod/base 20160805_QE5_nLC7_MEM_COLLAB_JH_C4_01		
Ratio mod/base 20160805_QE5_nLC7_MEM_COLLAB_JH_C5_01		
Ratio mod/base 20160805_QE5_nLC7_MEM_COLLAB_JH_C6_01		
Ratio mod/base 20160805_QE5_nLC7_MEM_COLLAB_JH_C7_01		

Ratio mod/base 20160805_QE5_nLC7_MEM_COLLAB_JH_C8_01		
Ratio mod/base FL0145_JHendy_130416_MSQ525_CW10		
Ratio mod/base FL0145_JHendy_130416_MSQ525_CW11		
Ratio mod/base FL0145_JHendy_130416_MSQ525_CW18		
Ratio mod/base FL0145_JHendy_130416_MSQ525_CW20		
Ratio mod/base FL0145_JHendy_130416_MSQ525_CW21		
Ratio mod/base FL0145_JHendy_130416_MSQ525_CW23		
Ratio mod/base FL0145_JHendy_130416_MSQ525_CW8_160425165036		
Ratio mod/base FL0168_JHendy_MSQ525_170516_BK		
Ratio mod/base FL0168_JHendy_MSQ525_170516_CW1		
Ratio mod/base FL0168_JHendy_MSQ525_170516_CW10		
Ratio mod/base FL0168_JHendy_MSQ525_170516_CW2		
Ratio mod/base FL0168_JHendy_MSQ525_170516_CW3		
Ratio mod/base FL0168_JHendy_MSQ525_170516_CW4		
Ratio mod/base FL0168_JHendy_MSQ525_170516_CW5		
Ratio mod/base FL0168_JHendy_MSQ525_170516_CW6		
Ratio mod/base FL0168_JHendy_MSQ525_170516_CW7		
Ratio mod/base FL0168_JHendy_MSQ525_170516_CW8		
Ratio mod/base FL0168_JHendy_MSQ525_170516_CW9		
Intensity 20160805_QE5_nLC7_MEM_COLLAB_JH_A1_01___1		Summed up eXtracted Ion Current (XIC) of all isotopic clusters associated with the identified AA sequence. In case of a labeled experiment this is the total intensity of all the isotopic patterns in the label cluster.
Intensity 20160805_QE5_nLC7_MEM_COLLAB_JH_A1_01___2		Summed up eXtracted Ion Current (XIC) of all isotopic clusters associated with the identified AA sequence. In case of a labeled experiment this is the total intensity of all the isotopic patterns in the label cluster.
Intensity 20160805_QE5_nLC7_MEM_COLLAB_JH_A1_01___3		Summed up eXtracted Ion Current (XIC) of all isotopic clusters associated with the identified AA sequence. In case of a labeled experiment this is the total intensity of all the isotopic patterns in the label cluster.
Intensity 20160805_QE5_nLC7_MEM_COLLAB_JH_A2_01___1		Summed up eXtracted Ion Current (XIC) of all isotopic clusters associated with the identified AA sequence. In case of a labeled experiment this is the total intensity of all the isotopic patterns in the label cluster.
Intensity 20160805_QE5_nLC7_MEM_COLLAB_JH_A2_01___2		Summed up eXtracted Ion Current (XIC) of all isotopic clusters associated with the identified AA sequence. In case of a labeled experiment this is the total intensity of all the isotopic patterns in the label cluster.
Intensity 20160805_QE5_nLC7_MEM_COLLAB_JH_A2_01___3		Summed up eXtracted Ion Current (XIC) of all isotopic clusters associated with the identified AA sequence. In case of a labeled experiment this is the total intensity of all the isotopic patterns in the label cluster.

Intensity 20160805_QE5_nLC7_MEM_COLLAB_JH_A3_01___1		Summed up eXtracted Ion Current (XIC) of all isotopic clusters associated with the identified AA sequence. In case of a labeled experiment this is the total intensity of all the isotopic patterns in the label cluster.
Intensity 20160805_QE5_nLC7_MEM_COLLAB_JH_A3_01___2		Summed up eXtracted Ion Current (XIC) of all isotopic clusters associated with the identified AA sequence. In case of a labeled experiment this is the total intensity of all the isotopic patterns in the label cluster.
Intensity 20160805_QE5_nLC7_MEM_COLLAB_JH_A3_01___3		Summed up eXtracted Ion Current (XIC) of all isotopic clusters associated with the identified AA sequence. In case of a labeled experiment this is the total intensity of all the isotopic patterns in the label cluster.
Intensity 20160805_QE5_nLC7_MEM_COLLAB_JH_B1_01___1		Summed up eXtracted Ion Current (XIC) of all isotopic clusters associated with the identified AA sequence. In case of a labeled experiment this is the total intensity of all the isotopic patterns in the label cluster.
Intensity 20160805_QE5_nLC7_MEM_COLLAB_JH_B1_01___2		Summed up eXtracted Ion Current (XIC) of all isotopic clusters associated with the identified AA sequence. In case of a labeled experiment this is the total intensity of all the isotopic patterns in the label cluster.
Intensity 20160805_QE5_nLC7_MEM_COLLAB_JH_B1_01___3		Summed up eXtracted Ion Current (XIC) of all isotopic clusters associated with the identified AA sequence. In case of a labeled experiment this is the total intensity of all the isotopic patterns in the label cluster.
Intensity 20160805_QE5_nLC7_MEM_COLLAB_JH_B2_01___1		Summed up eXtracted Ion Current (XIC) of all isotopic clusters associated with the identified AA sequence. In case of a labeled experiment this is the total intensity of all the isotopic patterns in the label cluster.
Intensity 20160805_QE5_nLC7_MEM_COLLAB_JH_B2_01___2		Summed up eXtracted Ion Current (XIC) of all isotopic clusters associated with the identified AA sequence. In case of a labeled experiment this is the total intensity of all the isotopic patterns in the label cluster.
Intensity 20160805_QE5_nLC7_MEM_COLLAB_JH_B2_01___3		Summed up eXtracted Ion Current (XIC) of all isotopic clusters associated with the identified AA sequence. In case of a labeled experiment this is the total intensity of all the isotopic patterns in the label cluster.
Intensity 20160805_QE5_nLC7_MEM_COLLAB_JH_B3_01___1		Summed up eXtracted Ion Current (XIC) of all isotopic clusters associated with the identified AA sequence. In case of a labeled experiment this is the total intensity of all the isotopic patterns in the label cluster.
Intensity 20160805_QE5_nLC7_MEM_COLLAB_JH_B3_01___2		Summed up eXtracted Ion Current (XIC) of all isotopic clusters associated with the identified AA sequence. In case of a labeled experiment this is the total intensity of all the isotopic patterns in the label cluster.
Intensity 20160805_QE5_nLC7_MEM_COLLAB_JH_B3_01___3		Summed up eXtracted Ion Current (XIC) of all isotopic clusters associated with the identified AA sequence. In case of a labeled experiment this is the total intensity of all the isotopic patterns in the label cluster.
Intensity 20160805_QE5_nLC7_MEM_COLLAB_JH_B4_01___1		Summed up eXtracted Ion Current (XIC) of all isotopic clusters associated with the identified AA sequence. In case of a labeled experiment this is the total intensity of all the isotopic patterns in the label cluster.
Intensity 20160805_QE5_nLC7_MEM_COLLAB_JH_B4_01___2		Summed up eXtracted Ion Current (XIC) of all isotopic clusters associated with the identified AA sequence. In case of a labeled experiment this is the total intensity of all the isotopic patterns in the label cluster.
Intensity 20160805_QE5_nLC7_MEM_COLLAB_JH_B4_01___3		Summed up eXtracted Ion Current (XIC) of all isotopic clusters associated with the identified AA sequence. In case of a labeled experiment this is the total intensity of all the isotopic patterns in the label cluster.
Intensity 20160805_QE5_nLC7_MEM_COLLAB_JH_B5_01___1		Summed up eXtracted Ion Current (XIC) of all isotopic clusters associated with the identified AA sequence. In case of a labeled experiment this is the total intensity of all the isotopic patterns in the label cluster.
Intensity 20160805_QE5_nLC7_MEM_COLLAB_JH_B5_01___2		Summed up eXtracted Ion Current (XIC) of all isotopic clusters associated with the identified AA sequence. In case of a labeled experiment this is the total intensity of all the isotopic patterns in the label cluster.
Intensity 20160805_QE5_nLC7_MEM_COLLAB_JH_B5_01___3		Summed up eXtracted Ion Current (XIC) of all isotopic clusters associated with the identified AA sequence. In case of a labeled experiment this is the total intensity of all the isotopic patterns in the label cluster.
Intensity 20160805_QE5_nLC7_MEM_COLLAB_JH_C1_01___1		Summed up eXtracted Ion Current (XIC) of all isotopic clusters associated with the identified AA sequence. In case of a labeled experiment this is the total intensity of all the isotopic patterns in the label cluster.
Intensity 20160805_QE5_nLC7_MEM_COLLAB_JH_C1_01___2		Summed up eXtracted Ion Current (XIC) of all isotopic clusters associated with the identified AA sequence. In case of a labeled experiment this is the total intensity of all the isotopic patterns in the label cluster.
Intensity 20160805_QE5_nLC7_MEM_COLLAB_JH_C1_01___3		Summed up eXtracted Ion Current (XIC) of all isotopic clusters associated with the identified AA sequence. In case of a labeled experiment this is the total intensity of all the isotopic patterns in the label cluster.

Intensity 20160805_QE5_nLC7_MEM_COLLAB_JH_C2_01___1		Summed up eXtracted Ion Current (XIC) of all isotopic clusters associated with the identified AA sequence. In case of a labeled experiment this is the total intensity of all the isotopic patterns in the label cluster.
Intensity 20160805_QE5_nLC7_MEM_COLLAB_JH_C2_01___2		Summed up eXtracted Ion Current (XIC) of all isotopic clusters associated with the identified AA sequence. In case of a labeled experiment this is the total intensity of all the isotopic patterns in the label cluster.
Intensity 20160805_QE5_nLC7_MEM_COLLAB_JH_C2_01___3		Summed up eXtracted Ion Current (XIC) of all isotopic clusters associated with the identified AA sequence. In case of a labeled experiment this is the total intensity of all the isotopic patterns in the label cluster.
Intensity 20160805_QE5_nLC7_MEM_COLLAB_JH_C3_01___1		Summed up eXtracted Ion Current (XIC) of all isotopic clusters associated with the identified AA sequence. In case of a labeled experiment this is the total intensity of all the isotopic patterns in the label cluster.
Intensity 20160805_QE5_nLC7_MEM_COLLAB_JH_C3_01___2		Summed up eXtracted Ion Current (XIC) of all isotopic clusters associated with the identified AA sequence. In case of a labeled experiment this is the total intensity of all the isotopic patterns in the label cluster.
Intensity 20160805_QE5_nLC7_MEM_COLLAB_JH_C3_01___3		Summed up eXtracted Ion Current (XIC) of all isotopic clusters associated with the identified AA sequence. In case of a labeled experiment this is the total intensity of all the isotopic patterns in the label cluster.
Intensity 20160805_QE5_nLC7_MEM_COLLAB_JH_C4_01___1		Summed up eXtracted Ion Current (XIC) of all isotopic clusters associated with the identified AA sequence. In case of a labeled experiment this is the total intensity of all the isotopic patterns in the label cluster.
Intensity 20160805_QE5_nLC7_MEM_COLLAB_JH_C4_01___2		Summed up eXtracted Ion Current (XIC) of all isotopic clusters associated with the identified AA sequence. In case of a labeled experiment this is the total intensity of all the isotopic patterns in the label cluster.
Intensity 20160805_QE5_nLC7_MEM_COLLAB_JH_C4_01___3		Summed up eXtracted Ion Current (XIC) of all isotopic clusters associated with the identified AA sequence. In case of a labeled experiment this is the total intensity of all the isotopic patterns in the label cluster.
Intensity 20160805_QE5_nLC7_MEM_COLLAB_JH_C5_01___1		Summed up eXtracted Ion Current (XIC) of all isotopic clusters associated with the identified AA sequence. In case of a labeled experiment this is the total intensity of all the isotopic patterns in the label cluster.
Intensity 20160805_QE5_nLC7_MEM_COLLAB_JH_C5_01___2		Summed up eXtracted Ion Current (XIC) of all isotopic clusters associated with the identified AA sequence. In case of a labeled experiment this is the total intensity of all the isotopic patterns in the label cluster.
Intensity 20160805_QE5_nLC7_MEM_COLLAB_JH_C5_01___3		Summed up eXtracted Ion Current (XIC) of all isotopic clusters associated with the identified AA sequence. In case of a labeled experiment this is the total intensity of all the isotopic patterns in the label cluster.
Intensity 20160805_QE5_nLC7_MEM_COLLAB_JH_C6_01___1		Summed up eXtracted Ion Current (XIC) of all isotopic clusters associated with the identified AA sequence. In case of a labeled experiment this is the total intensity of all the isotopic patterns in the label cluster.
Intensity 20160805_QE5_nLC7_MEM_COLLAB_JH_C6_01___2		Summed up eXtracted Ion Current (XIC) of all isotopic clusters associated with the identified AA sequence. In case of a labeled experiment this is the total intensity of all the isotopic patterns in the label cluster.
Intensity 20160805_QE5_nLC7_MEM_COLLAB_JH_C6_01___3		Summed up eXtracted Ion Current (XIC) of all isotopic clusters associated with the identified AA sequence. In case of a labeled experiment this is the total intensity of all the isotopic patterns in the label cluster.
Intensity 20160805_QE5_nLC7_MEM_COLLAB_JH_C7_01___1		Summed up eXtracted Ion Current (XIC) of all isotopic clusters associated with the identified AA sequence. In case of a labeled experiment this is the total intensity of all the isotopic patterns in the label cluster.
Intensity 20160805_QE5_nLC7_MEM_COLLAB_JH_C7_01___2		Summed up eXtracted Ion Current (XIC) of all isotopic clusters associated with the identified AA sequence. In case of a labeled experiment this is the total intensity of all the isotopic patterns in the label cluster.
Intensity 20160805_QE5_nLC7_MEM_COLLAB_JH_C7_01___3		Summed up eXtracted Ion Current (XIC) of all isotopic clusters associated with the identified AA sequence. In case of a labeled experiment this is the total intensity of all the isotopic patterns in the label cluster.
Intensity 20160805_QE5_nLC7_MEM_COLLAB_JH_C8_01___1		Summed up eXtracted Ion Current (XIC) of all isotopic clusters associated with the identified AA sequence. In case of a labeled experiment this is the total intensity of all the isotopic patterns in the label cluster.
Intensity 20160805_QE5_nLC7_MEM_COLLAB_JH_C8_01___2		Summed up eXtracted Ion Current (XIC) of all isotopic clusters associated with the identified AA sequence. In case of a labeled experiment this is the total intensity of all the isotopic patterns in the label cluster.
Intensity 20160805_QE5_nLC7_MEM_COLLAB_JH_C8_01___3		Summed up eXtracted Ion Current (XIC) of all isotopic clusters associated with the identified AA sequence. In case of a labeled experiment this is the total intensity of all the isotopic patterns in the label cluster.

Intensity FL0145_JHendy_130416_MSQ525_CW10___1		Summed up eXtracted Ion Current (XIC) of all isotopic clusters associated with the identified AA sequence. In case of a labeled experiment this is the total intensity of all the isotopic patterns in the label cluster.
Intensity FL0145_JHendy_130416_MSQ525_CW10___2		Summed up eXtracted Ion Current (XIC) of all isotopic clusters associated with the identified AA sequence. In case of a labeled experiment this is the total intensity of all the isotopic patterns in the label cluster.
Intensity FL0145_JHendy_130416_MSQ525_CW10___3		Summed up eXtracted Ion Current (XIC) of all isotopic clusters associated with the identified AA sequence. In case of a labeled experiment this is the total intensity of all the isotopic patterns in the label cluster.
Intensity FL0145_JHendy_130416_MSQ525_CW11___1		Summed up eXtracted Ion Current (XIC) of all isotopic clusters associated with the identified AA sequence. In case of a labeled experiment this is the total intensity of all the isotopic patterns in the label cluster.
Intensity FL0145_JHendy_130416_MSQ525_CW11___2		Summed up eXtracted Ion Current (XIC) of all isotopic clusters associated with the identified AA sequence. In case of a labeled experiment this is the total intensity of all the isotopic patterns in the label cluster.
Intensity FL0145_JHendy_130416_MSQ525_CW11___3		Summed up eXtracted Ion Current (XIC) of all isotopic clusters associated with the identified AA sequence. In case of a labeled experiment this is the total intensity of all the isotopic patterns in the label cluster.
Intensity FL0145_JHendy_130416_MSQ525_CW18___1		Summed up eXtracted Ion Current (XIC) of all isotopic clusters associated with the identified AA sequence. In case of a labeled experiment this is the total intensity of all the isotopic patterns in the label cluster.
Intensity FL0145_JHendy_130416_MSQ525_CW18___2		Summed up eXtracted Ion Current (XIC) of all isotopic clusters associated with the identified AA sequence. In case of a labeled experiment this is the total intensity of all the isotopic patterns in the label cluster.
Intensity FL0145_JHendy_130416_MSQ525_CW18___3		Summed up eXtracted Ion Current (XIC) of all isotopic clusters associated with the identified AA sequence. In case of a labeled experiment this is the total intensity of all the isotopic patterns in the label cluster.
Intensity FL0145_JHendy_130416_MSQ525_CW20___1		Summed up eXtracted Ion Current (XIC) of all isotopic clusters associated with the identified AA sequence. In case of a labeled experiment this is the total intensity of all the isotopic patterns in the label cluster.
Intensity FL0145_JHendy_130416_MSQ525_CW20___2		Summed up eXtracted Ion Current (XIC) of all isotopic clusters associated with the identified AA sequence. In case of a labeled experiment this is the total intensity of all the isotopic patterns in the label cluster.
Intensity FL0145_JHendy_130416_MSQ525_CW20___3		Summed up eXtracted Ion Current (XIC) of all isotopic clusters associated with the identified AA sequence. In case of a labeled experiment this is the total intensity of all the isotopic patterns in the label cluster.
Intensity FL0145_JHendy_130416_MSQ525_CW21___1		Summed up eXtracted Ion Current (XIC) of all isotopic clusters associated with the identified AA sequence. In case of a labeled experiment this is the total intensity of all the isotopic patterns in the label cluster.
Intensity FL0145_JHendy_130416_MSQ525_CW21___2		Summed up eXtracted Ion Current (XIC) of all isotopic clusters associated with the identified AA sequence. In case of a labeled experiment this is the total intensity of all the isotopic patterns in the label cluster.
Intensity FL0145_JHendy_130416_MSQ525_CW21___3		Summed up eXtracted Ion Current (XIC) of all isotopic clusters associated with the identified AA sequence. In case of a labeled experiment this is the total intensity of all the isotopic patterns in the label cluster.
Intensity FL0145_JHendy_130416_MSQ525_CW23___1		Summed up eXtracted Ion Current (XIC) of all isotopic clusters associated with the identified AA sequence. In case of a labeled experiment this is the total intensity of all the isotopic patterns in the label cluster.
Intensity FL0145_JHendy_130416_MSQ525_CW23___2		Summed up eXtracted Ion Current (XIC) of all isotopic clusters associated with the identified AA sequence. In case of a labeled experiment this is the total intensity of all the isotopic patterns in the label cluster.
Intensity FL0145_JHendy_130416_MSQ525_CW23___3		Summed up eXtracted Ion Current (XIC) of all isotopic clusters associated with the identified AA sequence. In case of a labeled experiment this is the total intensity of all the isotopic patterns in the label cluster.
Intensity FL0145_JHendy_130416_MSQ525_CW8_160425165036___1		Summed up eXtracted Ion Current (XIC) of all isotopic clusters associated with the identified AA sequence. In case of a labeled experiment this is the total intensity of all the isotopic patterns in the label cluster.
Intensity FL0145_JHendy_130416_MSQ525_CW8_160425165036___2		Summed up eXtracted Ion Current (XIC) of all isotopic clusters associated with the identified AA sequence. In case of a labeled experiment this is the total intensity of all the isotopic patterns in the label cluster.
Intensity FL0145_JHendy_130416_MSQ525_CW8_160425165036___3		Summed up eXtracted Ion Current (XIC) of all isotopic clusters associated with the identified AA sequence. In case of a labeled experiment this is the total intensity of all the isotopic patterns in the label cluster.

Intensity FL0168_JHendy_MSQ525_170516_BK___1		Summed up eXtracted Ion Current (XIC) of all isotopic clusters associated with the identified AA sequence. In case of a labeled experiment this is the total intensity of all the isotopic patterns in the label cluster.
Intensity FL0168_JHendy_MSQ525_170516_BK___2		Summed up eXtracted Ion Current (XIC) of all isotopic clusters associated with the identified AA sequence. In case of a labeled experiment this is the total intensity of all the isotopic patterns in the label cluster.
Intensity FL0168_JHendy_MSQ525_170516_BK___3		Summed up eXtracted Ion Current (XIC) of all isotopic clusters associated with the identified AA sequence. In case of a labeled experiment this is the total intensity of all the isotopic patterns in the label cluster.
Intensity FL0168_JHendy_MSQ525_170516_CW1___1		Summed up eXtracted Ion Current (XIC) of all isotopic clusters associated with the identified AA sequence. In case of a labeled experiment this is the total intensity of all the isotopic patterns in the label cluster.
Intensity FL0168_JHendy_MSQ525_170516_CW1___2		Summed up eXtracted Ion Current (XIC) of all isotopic clusters associated with the identified AA sequence. In case of a labeled experiment this is the total intensity of all the isotopic patterns in the label cluster.
Intensity FL0168_JHendy_MSQ525_170516_CW1___3		Summed up eXtracted Ion Current (XIC) of all isotopic clusters associated with the identified AA sequence. In case of a labeled experiment this is the total intensity of all the isotopic patterns in the label cluster.
Intensity FL0168_JHendy_MSQ525_170516_CW10___1		Summed up eXtracted Ion Current (XIC) of all isotopic clusters associated with the identified AA sequence. In case of a labeled experiment this is the total intensity of all the isotopic patterns in the label cluster.
Intensity FL0168_JHendy_MSQ525_170516_CW10___2		Summed up eXtracted Ion Current (XIC) of all isotopic clusters associated with the identified AA sequence. In case of a labeled experiment this is the total intensity of all the isotopic patterns in the label cluster.
Intensity FL0168_JHendy_MSQ525_170516_CW10___3		Summed up eXtracted Ion Current (XIC) of all isotopic clusters associated with the identified AA sequence. In case of a labeled experiment this is the total intensity of all the isotopic patterns in the label cluster.
Intensity FL0168_JHendy_MSQ525_170516_CW2___1		Summed up eXtracted Ion Current (XIC) of all isotopic clusters associated with the identified AA sequence. In case of a labeled experiment this is the total intensity of all the isotopic patterns in the label cluster.
Intensity FL0168_JHendy_MSQ525_170516_CW2___2		Summed up eXtracted Ion Current (XIC) of all isotopic clusters associated with the identified AA sequence. In case of a labeled experiment this is the total intensity of all the isotopic patterns in the label cluster.
Intensity FL0168_JHendy_MSQ525_170516_CW2___3		Summed up eXtracted Ion Current (XIC) of all isotopic clusters associated with the identified AA sequence. In case of a labeled experiment this is the total intensity of all the isotopic patterns in the label cluster.
Intensity FL0168_JHendy_MSQ525_170516_CW3___1		Summed up eXtracted Ion Current (XIC) of all isotopic clusters associated with the identified AA sequence. In case of a labeled experiment this is the total intensity of all the isotopic patterns in the label cluster.
Intensity FL0168_JHendy_MSQ525_170516_CW3___2		Summed up eXtracted Ion Current (XIC) of all isotopic clusters associated with the identified AA sequence. In case of a labeled experiment this is the total intensity of all the isotopic patterns in the label cluster.
Intensity FL0168_JHendy_MSQ525_170516_CW3___3		Summed up eXtracted Ion Current (XIC) of all isotopic clusters associated with the identified AA sequence. In case of a labeled experiment this is the total intensity of all the isotopic patterns in the label cluster.
Intensity FL0168_JHendy_MSQ525_170516_CW4___1		Summed up eXtracted Ion Current (XIC) of all isotopic clusters associated with the identified AA sequence. In case of a labeled experiment this is the total intensity of all the isotopic patterns in the label cluster.
Intensity FL0168_JHendy_MSQ525_170516_CW4___2		Summed up eXtracted Ion Current (XIC) of all isotopic clusters associated with the identified AA sequence. In case of a labeled experiment this is the total intensity of all the isotopic patterns in the label cluster.
Intensity FL0168_JHendy_MSQ525_170516_CW4___3		Summed up eXtracted Ion Current (XIC) of all isotopic clusters associated with the identified AA sequence. In case of a labeled experiment this is the total intensity of all the isotopic patterns in the label cluster.
Intensity FL0168_JHendy_MSQ525_170516_CW5___1		Summed up eXtracted Ion Current (XIC) of all isotopic clusters associated with the identified AA sequence. In case of a labeled experiment this is the total intensity of all the isotopic patterns in the label cluster.
Intensity FL0168_JHendy_MSQ525_170516_CW5___2		Summed up eXtracted Ion Current (XIC) of all isotopic clusters associated with the identified AA sequence. In case of a labeled experiment this is the total intensity of all the isotopic patterns in the label cluster.
Intensity FL0168_JHendy_MSQ525_170516_CW5___3		Summed up eXtracted Ion Current (XIC) of all isotopic clusters associated with the identified AA sequence. In case of a labeled experiment this is the total intensity of all the isotopic patterns in the label cluster.

Intensity FL0168_JHendy_MSQ525_170516 _CW6___1		Summed up eXtracted Ion Current (XIC) of all isotopic clusters associated with the identified AA sequence. In case of a labeled experiment this is the total intensity of all the isotopic patterns in the label cluster.
Intensity FL0168_JHendy_MSQ525_170516 _CW6___2		Summed up eXtracted Ion Current (XIC) of all isotopic clusters associated with the identified AA sequence. In case of a labeled experiment this is the total intensity of all the isotopic patterns in the label cluster.
Intensity FL0168_JHendy_MSQ525_170516 _CW6___3		Summed up eXtracted Ion Current (XIC) of all isotopic clusters associated with the identified AA sequence. In case of a labeled experiment this is the total intensity of all the isotopic patterns in the label cluster.
Intensity FL0168_JHendy_MSQ525_170516 _CW7___1		Summed up eXtracted Ion Current (XIC) of all isotopic clusters associated with the identified AA sequence. In case of a labeled experiment this is the total intensity of all the isotopic patterns in the label cluster.
Intensity FL0168_JHendy_MSQ525_170516 _CW7___2		Summed up eXtracted Ion Current (XIC) of all isotopic clusters associated with the identified AA sequence. In case of a labeled experiment this is the total intensity of all the isotopic patterns in the label cluster.
Intensity FL0168_JHendy_MSQ525_170516 _CW7___3		Summed up eXtracted Ion Current (XIC) of all isotopic clusters associated with the identified AA sequence. In case of a labeled experiment this is the total intensity of all the isotopic patterns in the label cluster.
Intensity FL0168_JHendy_MSQ525_170516 _CW8___1		Summed up eXtracted Ion Current (XIC) of all isotopic clusters associated with the identified AA sequence. In case of a labeled experiment this is the total intensity of all the isotopic patterns in the label cluster.
Intensity FL0168_JHendy_MSQ525_170516 _CW8___2		Summed up eXtracted Ion Current (XIC) of all isotopic clusters associated with the identified AA sequence. In case of a labeled experiment this is the total intensity of all the isotopic patterns in the label cluster.
Intensity FL0168_JHendy_MSQ525_170516 _CW8___3		Summed up eXtracted Ion Current (XIC) of all isotopic clusters associated with the identified AA sequence. In case of a labeled experiment this is the total intensity of all the isotopic patterns in the label cluster.
Intensity FL0168_JHendy_MSQ525_170516 _CW9___1		Summed up eXtracted Ion Current (XIC) of all isotopic clusters associated with the identified AA sequence. In case of a labeled experiment this is the total intensity of all the isotopic patterns in the label cluster.
Intensity FL0168_JHendy_MSQ525_170516 _CW9___2		Summed up eXtracted Ion Current (XIC) of all isotopic clusters associated with the identified AA sequence. In case of a labeled experiment this is the total intensity of all the isotopic patterns in the label cluster.
Intensity FL0168_JHendy_MSQ525_170516 _CW9___3		Summed up eXtracted Ion Current (XIC) of all isotopic clusters associated with the identified AA sequence. In case of a labeled experiment this is the total intensity of all the isotopic patterns in the label cluster.
Occupancy 20160805_QE5_nLC7_MEM_COLL AB_JH_A1_01		
Occupancy ratio20160805_QE5_nLC7_MEM_C OLLAB_JH_A1_01		
Occupancy error scale 20160805_QE5_nLC7_MEM_COLL AB_JH_A1_01		
Occupancy 20160805_QE5_nLC7_MEM_COLL AB_JH_A2_01		
Occupancy ratio20160805_QE5_nLC7_MEM_C OLLAB_JH_A2_01		
Occupancy error scale 20160805_QE5_nLC7_MEM_COLL AB_JH_A2_01		
Occupancy 20160805_QE5_nLC7_MEM_COLL AB_JH_A3_01		
Occupancy ratio20160805_QE5_nLC7_MEM_C OLLAB_JH_A3_01		
Occupancy error scale 20160805_QE5_nLC7_MEM_COLL AB_JH_A3_01		
Occupancy 20160805_QE5_nLC7_MEM_COLL AB_JH_B1_01		
Occupancy ratio20160805_QE5_nLC7_MEM_C OLLAB_JH_B1_01		
Occupancy error scale 20160805_QE5_nLC7_MEM_COLL AB_JH_B1_01		

Occupancy 20160805_QE5_nLC7_MEM_COLLAB_JH_B2_01		
Occupancy ratio20160805_QE5_nLC7_MEM_COLLAB_JH_B2_01		
Occupancy error scale 20160805_QE5_nLC7_MEM_COLLAB_JH_B2_01		
Occupancy 20160805_QE5_nLC7_MEM_COLLAB_JH_B3_01		
Occupancy ratio20160805_QE5_nLC7_MEM_COLLAB_JH_B3_01		
Occupancy error scale 20160805_QE5_nLC7_MEM_COLLAB_JH_B3_01		
Occupancy 20160805_QE5_nLC7_MEM_COLLAB_JH_B4_01		
Occupancy ratio20160805_QE5_nLC7_MEM_COLLAB_JH_B4_01		
Occupancy error scale 20160805_QE5_nLC7_MEM_COLLAB_JH_B4_01		
Occupancy 20160805_QE5_nLC7_MEM_COLLAB_JH_B5_01		
Occupancy ratio20160805_QE5_nLC7_MEM_COLLAB_JH_B5_01		
Occupancy error scale 20160805_QE5_nLC7_MEM_COLLAB_JH_B5_01		
Occupancy 20160805_QE5_nLC7_MEM_COLLAB_JH_C1_01		
Occupancy ratio20160805_QE5_nLC7_MEM_COLLAB_JH_C1_01		
Occupancy error scale 20160805_QE5_nLC7_MEM_COLLAB_JH_C1_01		
Occupancy 20160805_QE5_nLC7_MEM_COLLAB_JH_C2_01		
Occupancy ratio20160805_QE5_nLC7_MEM_COLLAB_JH_C2_01		
Occupancy error scale 20160805_QE5_nLC7_MEM_COLLAB_JH_C2_01		
Occupancy 20160805_QE5_nLC7_MEM_COLLAB_JH_C3_01		
Occupancy ratio20160805_QE5_nLC7_MEM_COLLAB_JH_C3_01		
Occupancy error scale 20160805_QE5_nLC7_MEM_COLLAB_JH_C3_01		
Occupancy 20160805_QE5_nLC7_MEM_COLLAB_JH_C4_01		
Occupancy ratio20160805_QE5_nLC7_MEM_COLLAB_JH_C4_01		
Occupancy error scale 20160805_QE5_nLC7_MEM_COLLAB_JH_C4_01		
Occupancy 20160805_QE5_nLC7_MEM_COLLAB_JH_C5_01		
Occupancy ratio20160805_QE5_nLC7_MEM_COLLAB_JH_C5_01		
Occupancy error scale 20160805_QE5_nLC7_MEM_COLLAB_JH_C5_01		

Occupancy 20160805_QE5_nLC7_MEM_COLLAB_JH_C6_01		
Occupancy ratio20160805_QE5_nLC7_MEM_COLLAB_JH_C6_01		
Occupancy error scale 20160805_QE5_nLC7_MEM_COLLAB_JH_C6_01		
Occupancy 20160805_QE5_nLC7_MEM_COLLAB_JH_C7_01		
Occupancy ratio20160805_QE5_nLC7_MEM_COLLAB_JH_C7_01		
Occupancy error scale 20160805_QE5_nLC7_MEM_COLLAB_JH_C7_01		
Occupancy 20160805_QE5_nLC7_MEM_COLLAB_JH_C8_01		
Occupancy ratio20160805_QE5_nLC7_MEM_COLLAB_JH_C8_01		
Occupancy error scale 20160805_QE5_nLC7_MEM_COLLAB_JH_C8_01		
Occupancy FL0145_JHendy_130416_MSQ525_CW10		
Occupancy ratioFL0145_JHendy_130416_MSQ525_CW10		
Occupancy error scale FL0145_JHendy_130416_MSQ525_CW10		
Occupancy FL0145_JHendy_130416_MSQ525_CW11		
Occupancy ratioFL0145_JHendy_130416_MSQ525_CW11		
Occupancy error scale FL0145_JHendy_130416_MSQ525_CW11		
Occupancy FL0145_JHendy_130416_MSQ525_CW18		
Occupancy ratioFL0145_JHendy_130416_MSQ525_CW18		
Occupancy error scale FL0145_JHendy_130416_MSQ525_CW18		
Occupancy FL0145_JHendy_130416_MSQ525_CW20		
Occupancy ratioFL0145_JHendy_130416_MSQ525_CW20		
Occupancy error scale FL0145_JHendy_130416_MSQ525_CW20		
Occupancy FL0145_JHendy_130416_MSQ525_CW21		
Occupancy ratioFL0145_JHendy_130416_MSQ525_CW21		
Occupancy error scale FL0145_JHendy_130416_MSQ525_CW21		
Occupancy FL0145_JHendy_130416_MSQ525_CW23		
Occupancy ratioFL0145_JHendy_130416_MSQ525_CW23		
Occupancy error scale FL0145_JHendy_130416_MSQ525_CW23		

Occupancy FL0145_JHendy_130416_MSQ525_CW8_160425165036		
Occupancy ratioFL0145_JHendy_130416_MSQ525_CW8_160425165036		
Occupancy error scale FL0145_JHendy_130416_MSQ525_CW8_160425165036		
Occupancy FL0168_JHendy_MSQ525_170516_BK		
Occupancy ratioFL0168_JHendy_MSQ525_170516_BK		
Occupancy error scale FL0168_JHendy_MSQ525_170516_BK		
Occupancy FL0168_JHendy_MSQ525_170516_CW1		
Occupancy ratioFL0168_JHendy_MSQ525_170516_CW1		
Occupancy error scale FL0168_JHendy_MSQ525_170516_CW1		
Occupancy FL0168_JHendy_MSQ525_170516_CW10		
Occupancy ratioFL0168_JHendy_MSQ525_170516_CW10		
Occupancy error scale FL0168_JHendy_MSQ525_170516_CW10		
Occupancy FL0168_JHendy_MSQ525_170516_CW2		
Occupancy ratioFL0168_JHendy_MSQ525_170516_CW2		
Occupancy error scale FL0168_JHendy_MSQ525_170516_CW2		
Occupancy FL0168_JHendy_MSQ525_170516_CW3		
Occupancy ratioFL0168_JHendy_MSQ525_170516_CW3		
Occupancy error scale FL0168_JHendy_MSQ525_170516_CW3		
Occupancy FL0168_JHendy_MSQ525_170516_CW4		
Occupancy ratioFL0168_JHendy_MSQ525_170516_CW4		
Occupancy error scale FL0168_JHendy_MSQ525_170516_CW4		
Occupancy FL0168_JHendy_MSQ525_170516_CW5		
Occupancy ratioFL0168_JHendy_MSQ525_170516_CW5		
Occupancy error scale FL0168_JHendy_MSQ525_170516_CW5		
Occupancy FL0168_JHendy_MSQ525_170516_CW6		
Occupancy ratioFL0168_JHendy_MSQ525_170516_CW6		
Occupancy error scale FL0168_JHendy_MSQ525_170516_CW6		

Occupancy FL0168_JHendy_MSQ525_170516_CW7		
Occupancy ratioFL0168_JHendy_MSQ525_170516_CW7		
Occupancy error scale FL0168_JHendy_MSQ525_170516_CW7		
Occupancy FL0168_JHendy_MSQ525_170516_CW8		
Occupancy ratioFL0168_JHendy_MSQ525_170516_CW8		
Occupancy error scale FL0168_JHendy_MSQ525_170516_CW8		
Occupancy FL0168_JHendy_MSQ525_170516_CW9		
Occupancy ratioFL0168_JHendy_MSQ525_170516_CW9		
Occupancy error scale FL0168_JHendy_MSQ525_170516_CW9		
Reverse		When marked with '+', this particular peptide was found to be part of a protein derived from the reversed part of the protein sequence database. These should be removed for further data analysis.
Potential contaminant		When marked with '+', this particular peptide was found to be part of a commonly occurring contaminant. These should be removed for further data analysis.
id		A unique (consecutive) identifier for each row in the site table, which is used to cross-link the information in this file with the information stored in the other files.
Protein group IDs		The identifier of the protein-group this peptide sequence is associated with, which can be used to look up the extended protein information in the file 'proteinGroups.txt'.<br>As a single peptide can be linked to multiple proteins (e.g. in the case of razor-proteins), multiple id's can be stored here separated by a semicolon.<br>As a protein can be identified by multiple peptides, the same id can be found in different rows.
Positions		The positions of the modifications in the protein amino acid sequence.
Position		The position of the modification in the protein amino acid sequence.
Peptide IDs		Identifier(s) of the associated peptide sequence(s) summary, which can be found in the file 'peptides.txt'.
Mod. peptide IDs		Identifier(s) of the associated peptide sequence(s) summary, which can be found in the file 'modificationSpecificPeptides.txt'.
Evidence IDs		Identifier(s) for analyzed peptide evidence associated with the protein group referenced against the evidence table.
MS/MS IDs		The identifiers of the MS/MS scans identifying this peptide, referenced against the msms table.
Best localization evidence ID		
Best localization MS/MS ID		
Best localization raw file		
Best localization scan number		
Best score evidence ID		
Best score MS/MS ID		
Best score raw file		
Best score scan number		
Best PEP evidence ID		
Best PEP MS/MS ID		
Best PEP raw file		
Best PEP scan number		

Protein groups

The Protein Groups table contains information on the identified proteins in the processed raw-files. Each single row contains the group of proteins that could be reconstructed from a set of peptides.

Name	Separator	Description
Protein IDs		Identifier(s) of protein(s) contained in the protein group. They are sorted by number of identified peptides in descending order.
Majority protein IDs		These are the IDs of those proteins that have at least half of the peptides that the leading protein has.
Peptide counts (all)		Number of peptides associated with each protein in protein group, occurring in the order as the protein IDs occur in the 'Protein IDs' column. Here distinct peptide sequences are counted. Modified forms or different charges are counted as one peptide.
Peptide counts (razor+unique)		Number of peptides associated with each protein in protein group, occurring in the order as the protein IDs occur in the 'Protein IDs' column. Here distinct peptide sequences are counted. Modified forms or different charges are counted as one peptide.
Peptide counts (unique)		Number of peptides associated with each protein in protein group, occurring in the order as the protein IDs occur in the 'Protein IDs' column. Here distinct peptide sequences are counted. Modified forms or different charges are counted as one peptide.
Fasta headers		Fasta headers(s) of protein(s) contained within the group.
Number of proteins		Number of proteins contained within the group. This corresponds to the number of entries in the column 'Protein IDs'.
Peptides		The total number of peptide sequences associated with the protein group (i.e. for all the proteins in the group).
Razor + unique peptides		The total number of razor + unique peptides associated with the protein group (i.e. these peptides are shared with another protein group).
Unique peptides		The total number of unique peptides associated with the protein group (i.e. these peptides are not shared with another protein group).
Peptides 20160805_QE5_nLC7_MEM_COLLAB_JH_A1_01		Number of peptides (distinct peptide sequences) in experiment 20160805_QE5_nLC7_MEM_COLLAB_JH_A1_01
Peptides 20160805_QE5_nLC7_MEM_COLLAB_JH_A2_01		Number of peptides (distinct peptide sequences) in experiment 20160805_QE5_nLC7_MEM_COLLAB_JH_A2_01
Peptides 20160805_QE5_nLC7_MEM_COLLAB_JH_A3_01		Number of peptides (distinct peptide sequences) in experiment 20160805_QE5_nLC7_MEM_COLLAB_JH_A3_01
Peptides 20160805_QE5_nLC7_MEM_COLLAB_JH_B1_01		Number of peptides (distinct peptide sequences) in experiment 20160805_QE5_nLC7_MEM_COLLAB_JH_B1_01
Peptides 20160805_QE5_nLC7_MEM_COLLAB_JH_B2_01		Number of peptides (distinct peptide sequences) in experiment 20160805_QE5_nLC7_MEM_COLLAB_JH_B2_01
Peptides 20160805_QE5_nLC7_MEM_COLLAB_JH_B3_01		Number of peptides (distinct peptide sequences) in experiment 20160805_QE5_nLC7_MEM_COLLAB_JH_B3_01
Peptides 20160805_QE5_nLC7_MEM_COLLAB_JH_B4_01		Number of peptides (distinct peptide sequences) in experiment 20160805_QE5_nLC7_MEM_COLLAB_JH_B4_01
Peptides 20160805_QE5_nLC7_MEM_COLLAB_JH_B5_01		Number of peptides (distinct peptide sequences) in experiment 20160805_QE5_nLC7_MEM_COLLAB_JH_B5_01
Peptides 20160805_QE5_nLC7_MEM_COLLAB_JH_C1_01		Number of peptides (distinct peptide sequences) in experiment 20160805_QE5_nLC7_MEM_COLLAB_JH_C1_01
Peptides 20160805_QE5_nLC7_MEM_COLLAB_JH_C2_01		Number of peptides (distinct peptide sequences) in experiment 20160805_QE5_nLC7_MEM_COLLAB_JH_C2_01
Peptides 20160805_QE5_nLC7_MEM_COLLAB_JH_C3_01		Number of peptides (distinct peptide sequences) in experiment 20160805_QE5_nLC7_MEM_COLLAB_JH_C3_01
Peptides 20160805_QE5_nLC7_MEM_COLLAB_JH_C4_01		Number of peptides (distinct peptide sequences) in experiment 20160805_QE5_nLC7_MEM_COLLAB_JH_C4_01
Peptides 20160805_QE5_nLC7_MEM_COLLAB_JH_C5_01		Number of peptides (distinct peptide sequences) in experiment 20160805_QE5_nLC7_MEM_COLLAB_JH_C5_01

Peptides 20160805_QE5_nLC7_MEM_COLLAB_JH_C6_01		Number of peptides (distinct peptide sequences) in experiment 20160805_QE5_nLC7_MEM_COLLAB_JH_C6_01
Peptides 20160805_QE5_nLC7_MEM_COLLAB_JH_C7_01		Number of peptides (distinct peptide sequences) in experiment 20160805_QE5_nLC7_MEM_COLLAB_JH_C7_01
Peptides 20160805_QE5_nLC7_MEM_COLLAB_JH_C8_01		Number of peptides (distinct peptide sequences) in experiment 20160805_QE5_nLC7_MEM_COLLAB_JH_C8_01
Peptides FL0145_JHendy_130416_MSQ525_CW10		Number of peptides (distinct peptide sequences) in experiment FL0145_JHendy_130416_MSQ525_CW10
Peptides FL0145_JHendy_130416_MSQ525_CW11		Number of peptides (distinct peptide sequences) in experiment FL0145_JHendy_130416_MSQ525_CW11
Peptides FL0145_JHendy_130416_MSQ525_CW18		Number of peptides (distinct peptide sequences) in experiment FL0145_JHendy_130416_MSQ525_CW18
Peptides FL0145_JHendy_130416_MSQ525_CW20		Number of peptides (distinct peptide sequences) in experiment FL0145_JHendy_130416_MSQ525_CW20
Peptides FL0145_JHendy_130416_MSQ525_CW21		Number of peptides (distinct peptide sequences) in experiment FL0145_JHendy_130416_MSQ525_CW21
Peptides FL0145_JHendy_130416_MSQ525_CW23		Number of peptides (distinct peptide sequences) in experiment FL0145_JHendy_130416_MSQ525_CW23
Peptides FL0145_JHendy_130416_MSQ525_CW8_160425165036		Number of peptides (distinct peptide sequences) in experiment FL0145_JHendy_130416_MSQ525_CW8_160425165036
Peptides FL0168_JHendy_MSQ525_170516_BK		Number of peptides (distinct peptide sequences) in experiment FL0168_JHendy_MSQ525_170516_BK
Peptides FL0168_JHendy_MSQ525_170516_CW1		Number of peptides (distinct peptide sequences) in experiment FL0168_JHendy_MSQ525_170516_CW1
Peptides FL0168_JHendy_MSQ525_170516_CW10		Number of peptides (distinct peptide sequences) in experiment FL0168_JHendy_MSQ525_170516_CW10
Peptides FL0168_JHendy_MSQ525_170516_CW2		Number of peptides (distinct peptide sequences) in experiment FL0168_JHendy_MSQ525_170516_CW2
Peptides FL0168_JHendy_MSQ525_170516_CW3		Number of peptides (distinct peptide sequences) in experiment FL0168_JHendy_MSQ525_170516_CW3
Peptides FL0168_JHendy_MSQ525_170516_CW4		Number of peptides (distinct peptide sequences) in experiment FL0168_JHendy_MSQ525_170516_CW4
Peptides FL0168_JHendy_MSQ525_170516_CW5		Number of peptides (distinct peptide sequences) in experiment FL0168_JHendy_MSQ525_170516_CW5
Peptides FL0168_JHendy_MSQ525_170516_CW6		Number of peptides (distinct peptide sequences) in experiment FL0168_JHendy_MSQ525_170516_CW6
Peptides FL0168_JHendy_MSQ525_170516_CW7		Number of peptides (distinct peptide sequences) in experiment FL0168_JHendy_MSQ525_170516_CW7
Peptides FL0168_JHendy_MSQ525_170516_CW8		Number of peptides (distinct peptide sequences) in experiment FL0168_JHendy_MSQ525_170516_CW8
Peptides FL0168_JHendy_MSQ525_170516_CW9		Number of peptides (distinct peptide sequences) in experiment FL0168_JHendy_MSQ525_170516_CW9
Razor + unique peptides 20160805_QE5_nLC7_MEM_COLLAB_JH_A1_01		Number of razor + unique peptides (distinct peptide sequences) in experiment 20160805_QE5_nLC7_MEM_COLLAB_JH_A1_01
Razor + unique peptides 20160805_QE5_nLC7_MEM_COLLAB_JH_A2_01		Number of razor + unique peptides (distinct peptide sequences) in experiment 20160805_QE5_nLC7_MEM_COLLAB_JH_A2_01
Razor + unique peptides 20160805_QE5_nLC7_MEM_COLLAB_JH_A3_01		Number of razor + unique peptides (distinct peptide sequences) in experiment 20160805_QE5_nLC7_MEM_COLLAB_JH_A3_01
Razor + unique peptides 20160805_QE5_nLC7_MEM_COLLAB_JH_B1_01		Number of razor + unique peptides (distinct peptide sequences) in experiment 20160805_QE5_nLC7_MEM_COLLAB_JH_B1_01
Razor + unique peptides 20160805_QE5_nLC7_MEM_COLLAB_JH_B2_01		Number of razor + unique peptides (distinct peptide sequences) in experiment 20160805_QE5_nLC7_MEM_COLLAB_JH_B2_01
Razor + unique peptides 20160805_QE5_nLC7_MEM_COLLAB_JH_B3_01		Number of razor + unique peptides (distinct peptide sequences) in experiment 20160805_QE5_nLC7_MEM_COLLAB_JH_B3_01

Razor + unique peptides 20160805_QE5_nLC7_MEM_COLLAB_JH_B4_01		Number of razor + unique peptides (distinct peptide sequences) in experiment 20160805_QE5_nLC7_MEM_COLLAB_JH_B4_01
Razor + unique peptides 20160805_QE5_nLC7_MEM_COLLAB_JH_B5_01		Number of razor + unique peptides (distinct peptide sequences) in experiment 20160805_QE5_nLC7_MEM_COLLAB_JH_B5_01
Razor + unique peptides 20160805_QE5_nLC7_MEM_COLLAB_JH_C1_01		Number of razor + unique peptides (distinct peptide sequences) in experiment 20160805_QE5_nLC7_MEM_COLLAB_JH_C1_01
Razor + unique peptides 20160805_QE5_nLC7_MEM_COLLAB_JH_C2_01		Number of razor + unique peptides (distinct peptide sequences) in experiment 20160805_QE5_nLC7_MEM_COLLAB_JH_C2_01
Razor + unique peptides 20160805_QE5_nLC7_MEM_COLLAB_JH_C3_01		Number of razor + unique peptides (distinct peptide sequences) in experiment 20160805_QE5_nLC7_MEM_COLLAB_JH_C3_01
Razor + unique peptides 20160805_QE5_nLC7_MEM_COLLAB_JH_C4_01		Number of razor + unique peptides (distinct peptide sequences) in experiment 20160805_QE5_nLC7_MEM_COLLAB_JH_C4_01
Razor + unique peptides 20160805_QE5_nLC7_MEM_COLLAB_JH_C5_01		Number of razor + unique peptides (distinct peptide sequences) in experiment 20160805_QE5_nLC7_MEM_COLLAB_JH_C5_01
Razor + unique peptides 20160805_QE5_nLC7_MEM_COLLAB_JH_C6_01		Number of razor + unique peptides (distinct peptide sequences) in experiment 20160805_QE5_nLC7_MEM_COLLAB_JH_C6_01
Razor + unique peptides 20160805_QE5_nLC7_MEM_COLLAB_JH_C7_01		Number of razor + unique peptides (distinct peptide sequences) in experiment 20160805_QE5_nLC7_MEM_COLLAB_JH_C7_01
Razor + unique peptides 20160805_QE5_nLC7_MEM_COLLAB_JH_C8_01		Number of razor + unique peptides (distinct peptide sequences) in experiment 20160805_QE5_nLC7_MEM_COLLAB_JH_C8_01
Razor + unique peptides FL0145_JHendy_130416_MSQ525_CW10		Number of razor + unique peptides (distinct peptide sequences) in experiment FL0145_JHendy_130416_MSQ525_CW10
Razor + unique peptides FL0145_JHendy_130416_MSQ525_CW11		Number of razor + unique peptides (distinct peptide sequences) in experiment FL0145_JHendy_130416_MSQ525_CW11
Razor + unique peptides FL0145_JHendy_130416_MSQ525_CW18		Number of razor + unique peptides (distinct peptide sequences) in experiment FL0145_JHendy_130416_MSQ525_CW18
Razor + unique peptides FL0145_JHendy_130416_MSQ525_CW20		Number of razor + unique peptides (distinct peptide sequences) in experiment FL0145_JHendy_130416_MSQ525_CW20
Razor + unique peptides FL0145_JHendy_130416_MSQ525_CW21		Number of razor + unique peptides (distinct peptide sequences) in experiment FL0145_JHendy_130416_MSQ525_CW21
Razor + unique peptides FL0145_JHendy_130416_MSQ525_CW23		Number of razor + unique peptides (distinct peptide sequences) in experiment FL0145_JHendy_130416_MSQ525_CW23
Razor + unique peptides FL0145_JHendy_130416_MSQ525_CW8_160425165036		Number of razor + unique peptides (distinct peptide sequences) in experiment FL0145_JHendy_130416_MSQ525_CW8_160425165036
Razor + unique peptides FL0168_JHendy_MSQ525_170516_BK		Number of razor + unique peptides (distinct peptide sequences) in experiment FL0168_JHendy_MSQ525_170516_BK
Razor + unique peptides FL0168_JHendy_MSQ525_170516_CW1		Number of razor + unique peptides (distinct peptide sequences) in experiment FL0168_JHendy_MSQ525_170516_CW1
Razor + unique peptides FL0168_JHendy_MSQ525_170516_CW10		Number of razor + unique peptides (distinct peptide sequences) in experiment FL0168_JHendy_MSQ525_170516_CW10
Razor + unique peptides FL0168_JHendy_MSQ525_170516_CW2		Number of razor + unique peptides (distinct peptide sequences) in experiment FL0168_JHendy_MSQ525_170516_CW2
Razor + unique peptides FL0168_JHendy_MSQ525_170516_CW3		Number of razor + unique peptides (distinct peptide sequences) in experiment FL0168_JHendy_MSQ525_170516_CW3
Razor + unique peptides FL0168_JHendy_MSQ525_170516_CW4		Number of razor + unique peptides (distinct peptide sequences) in experiment FL0168_JHendy_MSQ525_170516_CW4
Razor + unique peptides FL0168_JHendy_MSQ525_170516_CW5		Number of razor + unique peptides (distinct peptide sequences) in experiment FL0168_JHendy_MSQ525_170516_CW5
Razor + unique peptides FL0168_JHendy_MSQ525_170516_CW6		Number of razor + unique peptides (distinct peptide sequences) in experiment FL0168_JHendy_MSQ525_170516_CW6
Razor + unique peptides FL0168_JHendy_MSQ525_170516_CW7		Number of razor + unique peptides (distinct peptide sequences) in experiment FL0168_JHendy_MSQ525_170516_CW7
Razor + unique peptides FL0168_JHendy_MSQ525_170516_CW8		Number of razor + unique peptides (distinct peptide sequences) in experiment FL0168_JHendy_MSQ525_170516_CW8

Razor + unique peptides FL0168_JHendy_MSQ525_170516_CW9		Number of razor + unique peptides (distinct peptide sequences) in experiment FL0168_JHendy_MSQ525_170516_CW9
Unique peptides 20160805_QE5_nLC7_MEM_COLLAB_JH_A1_01		Number of unique peptides (distinct peptide sequences) in experiment 20160805_QE5_nLC7_MEM_COLLAB_JH_A1_01
Unique peptides 20160805_QE5_nLC7_MEM_COLLAB_JH_A2_01		Number of unique peptides (distinct peptide sequences) in experiment 20160805_QE5_nLC7_MEM_COLLAB_JH_A2_01
Unique peptides 20160805_QE5_nLC7_MEM_COLLAB_JH_A3_01		Number of unique peptides (distinct peptide sequences) in experiment 20160805_QE5_nLC7_MEM_COLLAB_JH_A3_01
Unique peptides 20160805_QE5_nLC7_MEM_COLLAB_JH_B1_01		Number of unique peptides (distinct peptide sequences) in experiment 20160805_QE5_nLC7_MEM_COLLAB_JH_B1_01
Unique peptides 20160805_QE5_nLC7_MEM_COLLAB_JH_B2_01		Number of unique peptides (distinct peptide sequences) in experiment 20160805_QE5_nLC7_MEM_COLLAB_JH_B2_01
Unique peptides 20160805_QE5_nLC7_MEM_COLLAB_JH_B3_01		Number of unique peptides (distinct peptide sequences) in experiment 20160805_QE5_nLC7_MEM_COLLAB_JH_B3_01
Unique peptides 20160805_QE5_nLC7_MEM_COLLAB_JH_B4_01		Number of unique peptides (distinct peptide sequences) in experiment 20160805_QE5_nLC7_MEM_COLLAB_JH_B4_01
Unique peptides 20160805_QE5_nLC7_MEM_COLLAB_JH_B5_01		Number of unique peptides (distinct peptide sequences) in experiment 20160805_QE5_nLC7_MEM_COLLAB_JH_B5_01
Unique peptides 20160805_QE5_nLC7_MEM_COLLAB_JH_C1_01		Number of unique peptides (distinct peptide sequences) in experiment 20160805_QE5_nLC7_MEM_COLLAB_JH_C1_01
Unique peptides 20160805_QE5_nLC7_MEM_COLLAB_JH_C2_01		Number of unique peptides (distinct peptide sequences) in experiment 20160805_QE5_nLC7_MEM_COLLAB_JH_C2_01
Unique peptides 20160805_QE5_nLC7_MEM_COLLAB_JH_C3_01		Number of unique peptides (distinct peptide sequences) in experiment 20160805_QE5_nLC7_MEM_COLLAB_JH_C3_01
Unique peptides 20160805_QE5_nLC7_MEM_COLLAB_JH_C4_01		Number of unique peptides (distinct peptide sequences) in experiment 20160805_QE5_nLC7_MEM_COLLAB_JH_C4_01
Unique peptides 20160805_QE5_nLC7_MEM_COLLAB_JH_C5_01		Number of unique peptides (distinct peptide sequences) in experiment 20160805_QE5_nLC7_MEM_COLLAB_JH_C5_01
Unique peptides 20160805_QE5_nLC7_MEM_COLLAB_JH_C6_01		Number of unique peptides (distinct peptide sequences) in experiment 20160805_QE5_nLC7_MEM_COLLAB_JH_C6_01
Unique peptides 20160805_QE5_nLC7_MEM_COLLAB_JH_C7_01		Number of unique peptides (distinct peptide sequences) in experiment 20160805_QE5_nLC7_MEM_COLLAB_JH_C7_01
Unique peptides 20160805_QE5_nLC7_MEM_COLLAB_JH_C8_01		Number of unique peptides (distinct peptide sequences) in experiment 20160805_QE5_nLC7_MEM_COLLAB_JH_C8_01
Unique peptides FL0145_JHendy_130416_MSQ525_CW10		Number of unique peptides (distinct peptide sequences) in experiment FL0145_JHendy_130416_MSQ525_CW10
Unique peptides FL0145_JHendy_130416_MSQ525_CW11		Number of unique peptides (distinct peptide sequences) in experiment FL0145_JHendy_130416_MSQ525_CW11
Unique peptides FL0145_JHendy_130416_MSQ525_CW18		Number of unique peptides (distinct peptide sequences) in experiment FL0145_JHendy_130416_MSQ525_CW18
Unique peptides FL0145_JHendy_130416_MSQ525_CW20		Number of unique peptides (distinct peptide sequences) in experiment FL0145_JHendy_130416_MSQ525_CW20
Unique peptides FL0145_JHendy_130416_MSQ525_CW21		Number of unique peptides (distinct peptide sequences) in experiment FL0145_JHendy_130416_MSQ525_CW21
Unique peptides FL0145_JHendy_130416_MSQ525_CW23		Number of unique peptides (distinct peptide sequences) in experiment FL0145_JHendy_130416_MSQ525_CW23
Unique peptides FL0145_JHendy_130416_MSQ525_CW8_160425165036		Number of unique peptides (distinct peptide sequences) in experiment FL0145_JHendy_130416_MSQ525_CW8_160425165036
Unique peptides FL0168_JHendy_MSQ525_170516_BK		Number of unique peptides (distinct peptide sequences) in experiment FL0168_JHendy_MSQ525_170516_BK
Unique peptides FL0168_JHendy_MSQ525_170516_CW1		Number of unique peptides (distinct peptide sequences) in experiment FL0168_JHendy_MSQ525_170516_CW1
Unique peptides FL0168_JHendy_MSQ525_170516_CW10		Number of unique peptides (distinct peptide sequences) in experiment FL0168_JHendy_MSQ525_170516_CW10

Unique peptides FL0168_JHendy_MSQ525_170516_CW2		Number of unique peptides (distinct peptide sequences) in experiment FL0168_JHendy_MSQ525_170516_CW2
Unique peptides FL0168_JHendy_MSQ525_170516_CW3		Number of unique peptides (distinct peptide sequences) in experiment FL0168_JHendy_MSQ525_170516_CW3
Unique peptides FL0168_JHendy_MSQ525_170516_CW4		Number of unique peptides (distinct peptide sequences) in experiment FL0168_JHendy_MSQ525_170516_CW4
Unique peptides FL0168_JHendy_MSQ525_170516_CW5		Number of unique peptides (distinct peptide sequences) in experiment FL0168_JHendy_MSQ525_170516_CW5
Unique peptides FL0168_JHendy_MSQ525_170516_CW6		Number of unique peptides (distinct peptide sequences) in experiment FL0168_JHendy_MSQ525_170516_CW6
Unique peptides FL0168_JHendy_MSQ525_170516_CW7		Number of unique peptides (distinct peptide sequences) in experiment FL0168_JHendy_MSQ525_170516_CW7
Unique peptides FL0168_JHendy_MSQ525_170516_CW8		Number of unique peptides (distinct peptide sequences) in experiment FL0168_JHendy_MSQ525_170516_CW8
Unique peptides FL0168_JHendy_MSQ525_170516_CW9		Number of unique peptides (distinct peptide sequences) in experiment FL0168_JHendy_MSQ525_170516_CW9
Sequence coverage [%]		Percentage of the sequence that is covered by the identified peptides of the best protein sequence contained in the group.
Unique + razor sequence coverage [%]		Percentage of the sequence that is covered by the identified unique and razor peptides of the best protein sequence contained in the group.
Unique sequence coverage [%]		Percentage of the sequence that is covered by the identified unique peptides of the best protein sequence contained in the group.
Mol. weight [kDa]		Molecular weight of the leading protein sequence contained in the protein group.
Sequence length		The length of the leading protein sequence contained in the group.
Sequence lengths		The length of all sequences of the proteins contained in the group.
Q-value		This is the ratio of reverse to forward protein groups.
Score		Protein score which is derived from peptide posterior error probabilities.
Sequence coverage 20160805_QE5_nLC7_MEM_COLLAB_JH_A1_01 [%]		Percentage of the sequence that is covered by the identified peptides in this sample of the longest protein sequence contained within the group.
Sequence coverage 20160805_QE5_nLC7_MEM_COLLAB_JH_A2_01 [%]		Percentage of the sequence that is covered by the identified peptides in this sample of the longest protein sequence contained within the group.
Sequence coverage 20160805_QE5_nLC7_MEM_COLLAB_JH_A3_01 [%]		Percentage of the sequence that is covered by the identified peptides in this sample of the longest protein sequence contained within the group.
Sequence coverage 20160805_QE5_nLC7_MEM_COLLAB_JH_B1_01 [%]		Percentage of the sequence that is covered by the identified peptides in this sample of the longest protein sequence contained within the group.
Sequence coverage 20160805_QE5_nLC7_MEM_COLLAB_JH_B2_01 [%]		Percentage of the sequence that is covered by the identified peptides in this sample of the longest protein sequence contained within the group.
Sequence coverage 20160805_QE5_nLC7_MEM_COLLAB_JH_B3_01 [%]		Percentage of the sequence that is covered by the identified peptides in this sample of the longest protein sequence contained within the group.
Sequence coverage 20160805_QE5_nLC7_MEM_COLLAB_JH_B4_01 [%]		Percentage of the sequence that is covered by the identified peptides in this sample of the longest protein sequence contained within the group.
Sequence coverage 20160805_QE5_nLC7_MEM_COLLAB_JH_B5_01 [%]		Percentage of the sequence that is covered by the identified peptides in this sample of the longest protein sequence contained within the group.
Sequence coverage 20160805_QE5_nLC7_MEM_COLLAB_JH_C1_01 [%]		Percentage of the sequence that is covered by the identified peptides in this sample of the longest protein sequence contained within the group.
Sequence coverage 20160805_QE5_nLC7_MEM_COLLAB_JH_C2_01 [%]		Percentage of the sequence that is covered by the identified peptides in this sample of the longest protein sequence contained within the group.
Sequence coverage 20160805_QE5_nLC7_MEM_COLLAB_JH_C3_01 [%]		Percentage of the sequence that is covered by the identified peptides in this sample of the longest protein sequence contained within the group.
Sequence coverage 20160805_QE5_nLC7_MEM_COLLAB_JH_C4_01 [%]		Percentage of the sequence that is covered by the identified peptides in this sample of the longest protein sequence contained within the group.
Sequence coverage 20160805_QE5_nLC7_MEM_COLLAB_JH_C5_01 [%]		Percentage of the sequence that is covered by the identified peptides in this sample of the longest protein sequence contained within the group.

Sequence coverage 20160805_QE5_nLC7_MEM_COLLAB_JH_C6_01 [%]		Percentage of the sequence that is covered by the identified peptides in this sample of the longest protein sequence contained within the group.
Sequence coverage 20160805_QE5_nLC7_MEM_COLLAB_JH_C7_01 [%]		Percentage of the sequence that is covered by the identified peptides in this sample of the longest protein sequence contained within the group.
Sequence coverage 20160805_QE5_nLC7_MEM_COLLAB_JH_C8_01 [%]		Percentage of the sequence that is covered by the identified peptides in this sample of the longest protein sequence contained within the group.
Sequence coverage FL0145_JHendy_130416_MSQ525_CW10 [%]		Percentage of the sequence that is covered by the identified peptides in this sample of the longest protein sequence contained within the group.
Sequence coverage FL0145_JHendy_130416_MSQ525_CW11 [%]		Percentage of the sequence that is covered by the identified peptides in this sample of the longest protein sequence contained within the group.
Sequence coverage FL0145_JHendy_130416_MSQ525_CW18 [%]		Percentage of the sequence that is covered by the identified peptides in this sample of the longest protein sequence contained within the group.
Sequence coverage FL0145_JHendy_130416_MSQ525_CW20 [%]		Percentage of the sequence that is covered by the identified peptides in this sample of the longest protein sequence contained within the group.
Sequence coverage FL0145_JHendy_130416_MSQ525_CW21 [%]		Percentage of the sequence that is covered by the identified peptides in this sample of the longest protein sequence contained within the group.
Sequence coverage FL0145_JHendy_130416_MSQ525_CW23 [%]		Percentage of the sequence that is covered by the identified peptides in this sample of the longest protein sequence contained within the group.
Sequence coverage FL0145_JHendy_130416_MSQ525_CW8_160425165036 [%]		Percentage of the sequence that is covered by the identified peptides in this sample of the longest protein sequence contained within the group.
Sequence coverage FL0168_JHendy_MSQ525_170516_BK [%]		Percentage of the sequence that is covered by the identified peptides in this sample of the longest protein sequence contained within the group.
Sequence coverage FL0168_JHendy_MSQ525_170516_CW1 [%]		Percentage of the sequence that is covered by the identified peptides in this sample of the longest protein sequence contained within the group.
Sequence coverage FL0168_JHendy_MSQ525_170516_CW10 [%]		Percentage of the sequence that is covered by the identified peptides in this sample of the longest protein sequence contained within the group.
Sequence coverage FL0168_JHendy_MSQ525_170516_CW2 [%]		Percentage of the sequence that is covered by the identified peptides in this sample of the longest protein sequence contained within the group.
Sequence coverage FL0168_JHendy_MSQ525_170516_CW3 [%]		Percentage of the sequence that is covered by the identified peptides in this sample of the longest protein sequence contained within the group.
Sequence coverage FL0168_JHendy_MSQ525_170516_CW4 [%]		Percentage of the sequence that is covered by the identified peptides in this sample of the longest protein sequence contained within the group.
Sequence coverage FL0168_JHendy_MSQ525_170516_CW5 [%]		Percentage of the sequence that is covered by the identified peptides in this sample of the longest protein sequence contained within the group.
Sequence coverage FL0168_JHendy_MSQ525_170516_CW6 [%]		Percentage of the sequence that is covered by the identified peptides in this sample of the longest protein sequence contained within the group.
Sequence coverage FL0168_JHendy_MSQ525_170516_CW7 [%]		Percentage of the sequence that is covered by the identified peptides in this sample of the longest protein sequence contained within the group.
Sequence coverage FL0168_JHendy_MSQ525_170516_CW8 [%]		Percentage of the sequence that is covered by the identified peptides in this sample of the longest protein sequence contained within the group.
Sequence coverage FL0168_JHendy_MSQ525_170516_CW9 [%]		Percentage of the sequence that is covered by the identified peptides in this sample of the longest protein sequence contained within the group.
Intensity		Summed up eXtracted Ion Current (XIC) of all isotopic clusters associated with the identified AA sequence. In case of a labeled experiment this is the total intensity of all the isotopic patterns in the label cluster.
Intensity 20160805_QE5_nLC7_MEM_COLLAB_JH_A1_01		Summed up eXtracted Ion Current (XIC) of all isotopic clusters associated with the identified AA sequence. In case of a labeled experiment this is the total intensity of all the isotopic patterns in the label cluster.
Intensity 20160805_QE5_nLC7_MEM_COLLAB_JH_A2_01		Summed up eXtracted Ion Current (XIC) of all isotopic clusters associated with the identified AA sequence. In case of a labeled experiment this is the total intensity of all the isotopic patterns in the label cluster.
Intensity 20160805_QE5_nLC7_MEM_COLLAB_JH_A3_01		Summed up eXtracted Ion Current (XIC) of all isotopic clusters associated with the identified AA sequence. In case of a labeled experiment this is the total intensity of all the isotopic patterns in the label cluster.
Intensity 20160805_QE5_nLC7_MEM_COLLAB_JH_B1_01		Summed up eXtracted Ion Current (XIC) of all isotopic clusters associated with the identified AA sequence. In case of a labeled experiment this is the total intensity of all the isotopic patterns in the label cluster.

Intensity 20160805_QE5_nLC7_MEM_COLLAB_JH_B2_01		Summed up eXtracted Ion Current (XIC) of all isotopic clusters associated with the identified AA sequence. In case of a labeled experiment this is the total intensity of all the isotopic patterns in the label cluster.
Intensity 20160805_QE5_nLC7_MEM_COLLAB_JH_B3_01		Summed up eXtracted Ion Current (XIC) of all isotopic clusters associated with the identified AA sequence. In case of a labeled experiment this is the total intensity of all the isotopic patterns in the label cluster.
Intensity 20160805_QE5_nLC7_MEM_COLLAB_JH_B4_01		Summed up eXtracted Ion Current (XIC) of all isotopic clusters associated with the identified AA sequence. In case of a labeled experiment this is the total intensity of all the isotopic patterns in the label cluster.
Intensity 20160805_QE5_nLC7_MEM_COLLAB_JH_B5_01		Summed up eXtracted Ion Current (XIC) of all isotopic clusters associated with the identified AA sequence. In case of a labeled experiment this is the total intensity of all the isotopic patterns in the label cluster.
Intensity 20160805_QE5_nLC7_MEM_COLLAB_JH_C1_01		Summed up eXtracted Ion Current (XIC) of all isotopic clusters associated with the identified AA sequence. In case of a labeled experiment this is the total intensity of all the isotopic patterns in the label cluster.
Intensity 20160805_QE5_nLC7_MEM_COLLAB_JH_C2_01		Summed up eXtracted Ion Current (XIC) of all isotopic clusters associated with the identified AA sequence. In case of a labeled experiment this is the total intensity of all the isotopic patterns in the label cluster.
Intensity 20160805_QE5_nLC7_MEM_COLLAB_JH_C3_01		Summed up eXtracted Ion Current (XIC) of all isotopic clusters associated with the identified AA sequence. In case of a labeled experiment this is the total intensity of all the isotopic patterns in the label cluster.
Intensity 20160805_QE5_nLC7_MEM_COLLAB_JH_C4_01		Summed up eXtracted Ion Current (XIC) of all isotopic clusters associated with the identified AA sequence. In case of a labeled experiment this is the total intensity of all the isotopic patterns in the label cluster.
Intensity 20160805_QE5_nLC7_MEM_COLLAB_JH_C5_01		Summed up eXtracted Ion Current (XIC) of all isotopic clusters associated with the identified AA sequence. In case of a labeled experiment this is the total intensity of all the isotopic patterns in the label cluster.
Intensity 20160805_QE5_nLC7_MEM_COLLAB_JH_C6_01		Summed up eXtracted Ion Current (XIC) of all isotopic clusters associated with the identified AA sequence. In case of a labeled experiment this is the total intensity of all the isotopic patterns in the label cluster.
Intensity 20160805_QE5_nLC7_MEM_COLLAB_JH_C7_01		Summed up eXtracted Ion Current (XIC) of all isotopic clusters associated with the identified AA sequence. In case of a labeled experiment this is the total intensity of all the isotopic patterns in the label cluster.
Intensity 20160805_QE5_nLC7_MEM_COLLAB_JH_C8_01		Summed up eXtracted Ion Current (XIC) of all isotopic clusters associated with the identified AA sequence. In case of a labeled experiment this is the total intensity of all the isotopic patterns in the label cluster.
Intensity FL0145_JHendy_130416_MSQ525_CW10		Summed up eXtracted Ion Current (XIC) of all isotopic clusters associated with the identified AA sequence. In case of a labeled experiment this is the total intensity of all the isotopic patterns in the label cluster.
Intensity FL0145_JHendy_130416_MSQ525_CW11		Summed up eXtracted Ion Current (XIC) of all isotopic clusters associated with the identified AA sequence. In case of a labeled experiment this is the total intensity of all the isotopic patterns in the label cluster.
Intensity FL0145_JHendy_130416_MSQ525_CW18		Summed up eXtracted Ion Current (XIC) of all isotopic clusters associated with the identified AA sequence. In case of a labeled experiment this is the total intensity of all the isotopic patterns in the label cluster.
Intensity FL0145_JHendy_130416_MSQ525_CW20		Summed up eXtracted Ion Current (XIC) of all isotopic clusters associated with the identified AA sequence. In case of a labeled experiment this is the total intensity of all the isotopic patterns in the label cluster.
Intensity FL0145_JHendy_130416_MSQ525_CW21		Summed up eXtracted Ion Current (XIC) of all isotopic clusters associated with the identified AA sequence. In case of a labeled experiment this is the total intensity of all the isotopic patterns in the label cluster.
Intensity FL0145_JHendy_130416_MSQ525_CW23		Summed up eXtracted Ion Current (XIC) of all isotopic clusters associated with the identified AA sequence. In case of a labeled experiment this is the total intensity of all the isotopic patterns in the label cluster.
Intensity FL0145_JHendy_130416_MSQ525_CW8_160425165036		Summed up eXtracted Ion Current (XIC) of all isotopic clusters associated with the identified AA sequence. In case of a labeled experiment this is the total intensity of all the isotopic patterns in the label cluster.
Intensity FL0168_JHendy_MSQ525_170516_BK		Summed up eXtracted Ion Current (XIC) of all isotopic clusters associated with the identified AA sequence. In case of a labeled experiment this is the total intensity of all the isotopic patterns in the label cluster.
Intensity FL0168_JHendy_MSQ525_170516_CW1		Summed up eXtracted Ion Current (XIC) of all isotopic clusters associated with the identified AA sequence. In case of a labeled experiment this is the total intensity of all the isotopic patterns in the label cluster.

Intensity FL0168_JHendy_MSQ525_170516_CW10		Summed up eXtracted Ion Current (XIC) of all isotopic clusters associated with the identified AA sequence. In case of a labeled experiment this is the total intensity of all the isotopic patterns in the label cluster.
Intensity FL0168_JHendy_MSQ525_170516_CW2		Summed up eXtracted Ion Current (XIC) of all isotopic clusters associated with the identified AA sequence. In case of a labeled experiment this is the total intensity of all the isotopic patterns in the label cluster.
Intensity FL0168_JHendy_MSQ525_170516_CW3		Summed up eXtracted Ion Current (XIC) of all isotopic clusters associated with the identified AA sequence. In case of a labeled experiment this is the total intensity of all the isotopic patterns in the label cluster.
Intensity FL0168_JHendy_MSQ525_170516_CW4		Summed up eXtracted Ion Current (XIC) of all isotopic clusters associated with the identified AA sequence. In case of a labeled experiment this is the total intensity of all the isotopic patterns in the label cluster.
Intensity FL0168_JHendy_MSQ525_170516_CW5		Summed up eXtracted Ion Current (XIC) of all isotopic clusters associated with the identified AA sequence. In case of a labeled experiment this is the total intensity of all the isotopic patterns in the label cluster.
Intensity FL0168_JHendy_MSQ525_170516_CW6		Summed up eXtracted Ion Current (XIC) of all isotopic clusters associated with the identified AA sequence. In case of a labeled experiment this is the total intensity of all the isotopic patterns in the label cluster.
Intensity FL0168_JHendy_MSQ525_170516_CW7		Summed up eXtracted Ion Current (XIC) of all isotopic clusters associated with the identified AA sequence. In case of a labeled experiment this is the total intensity of all the isotopic patterns in the label cluster.
Intensity FL0168_JHendy_MSQ525_170516_CW8		Summed up eXtracted Ion Current (XIC) of all isotopic clusters associated with the identified AA sequence. In case of a labeled experiment this is the total intensity of all the isotopic patterns in the label cluster.
Intensity FL0168_JHendy_MSQ525_170516_CW9		Summed up eXtracted Ion Current (XIC) of all isotopic clusters associated with the identified AA sequence. In case of a labeled experiment this is the total intensity of all the isotopic patterns in the label cluster.
MS/MS count		
Only identified by site		When marked with '+', this particular protein group was identified only by a modification site.
Reverse		When marked with '+', this particular protein group contains no protein, made up of at least 50% of the peptides of the leading protein, with a peptide derived from the reversed part of the decoy database. These should be removed for further data analysis. The 50% rule is in place to prevent spurious protein hits to erroneously flag the protein group as reverse.
Potential contaminant		When marked with '+', this particular protein group was found to be a commonly occurring contaminant. These should be removed for further data analysis.
id		A unique (consecutive) identifier for each row in the proteinGroups table, which is used to cross-link the information in this file with the information stored in the other files.
Peptide IDs		Identifier(s) of the associated peptide sequence(s) summary, which can be found in the file 'peptides.txt'.
Peptide is razor		Indicates for each peptide ID if it is a razor or group unique peptide (true) or a non unique non razor peptide (false).
Mod. peptide IDs		
Evidence IDs		
MS/MS IDs		
Best MS/MS		The identifier of the best (in terms of quality) MS/MS scans identifying the peptides of this protein, referenced against the msms table.
Deamidation (NQ) site IDs		Identifier(s) for site(s) associated with the protein group, which show(s) evidence of the modification, referenced against the appropriate modification site file.
Hydroxyproline site IDs		Identifier(s) for site(s) associated with the protein group, which show(s) evidence of the modification, referenced against the appropriate modification site file.
Oxidation (M) site IDs		Identifier(s) for site(s) associated with the protein group, which show(s) evidence of the modification, referenced against the appropriate modification site file.
Propionamide K site IDs		Identifier(s) for site(s) associated with the protein group, which show(s) evidence of the modification, referenced against the appropriate modification site file.
Deamidation (NQ) site positions		Positions of the sites in the leading protein of this group.
Hydroxyproline site positions		Positions of the sites in the leading protein of this group.
Oxidation (M) site positions		Positions of the sites in the leading protein of this group.
Propionamide K site positions		Positions of the sites in the leading protein of this group.

All peptides

Name	Separator	Description
Raw file		Name of the raw file the spectral data was extracted from.
Type		The type of detection for the peptide. MULTI – A labeling multiplet was detected.<br>ISO – An isotope pattern was detected.
Charge		The charge state of the peptide.
m/z		The mass divided by the charge of the charged peptide.
Mass		The mass of the neutral peptide ((m/z-proton) * charge).
Uncalibrated m/z		m/z before recalibrations have been applied.
Resolution		The resolution of the peak detected for the peptide measured in Full Width at Half Maximum (FWHM).
Number of data points		The number of data points (peak centroids) collected for this peptide feature.
Number of scans		The number of MS scans that the 3d peaks of this peptide feature are overlapping with.
Number of isotopic peaks		The number of isotopic peaks contained in this peptide feature.
PIF		Short for Parent Ion Fraction; indicates the fraction the target peak makes up of the total intensity in the inclusion window.
Mass fractional part		The values after the radix point (ie value - floor(value)).
Mass deficit		Empirically derived deviation measure to the next nearest integer scaled to center around 0. Can be used to visually detect contaminants in a plot setting Mass against this value.<br><br>m*a+b – round(m*a+b)<br>m: the peptide mass<br>a: 0.999555<br>b: -0.10
Mass precision [ppm]		The precision of the mass detection of the peptide in parts-per-million.
Max intensity m/z 0		Summed up eXtracted Ion Current (XIC) of all isotopic clusters associated with the identified AA sequence. In case of a labeled experiment this is the total intensity of all the isotopic patterns in the label cluster.
Retention time		The retention time of the peak detected for the peptide measured in minutes.
Retention length		The total retention time width of the peak (last timepoint – first timepoint) in seconds.
Retention length (FWHM)		The full width at half maximum value retention time width of the peak in seconds.
Min scan number		The first scan number at which the peak was encountered.
Max scan number		The last scan number at which the peak was encountered.
Identified		When marked with '+' this particular MS/MS scan was identified as a peptide; when marked with '-' no identification was made.
MS/MS IDs		Unique identifier linking this identification to the MS/MS scans.
Sequence		The identified AA sequence of the peptide.
Length		The length of the sequence stored in the column “Sequence”.
Modifications		Post-translational modifications contained within the sequence. When no modifications exist, this is set to ‘unmodified’.<br><br>Note: This column only set when this MS/MS spectrum has been identified.
Modified sequence		Sequence representation of the peptide including location(s) of modified AAs.<br><br>Note: This column only set when this MS/MS spectrum has been identified.
Proteins		Identifiers of proteins this peptide is associated with.<br><br>Note: This column only set when this MS/MS spectrum has been identified.
Score		The score of the identification (higher is better).<br><br>Note: This column only set when this MS/MS spectrum has been identified.
Intensity		Summed up eXtracted Ion Current (XIC) of all isotopic clusters associated with the identified AA sequence. In case of a labeled experiment this is the total intensity of all the isotopic patterns in the label cluster.
Intensities		Elution profile.
Isotope pattern		Isotope pattern.

MS/MS Count		The number of MS/MS spectra recorded for the peptide.
MSMS Scan Numbers		The scan numbers where the MS/MS spectra were recorded.
MSMS Isotope Indices		Indices of the isotopic peaks that the MS/MS spectra reside on. A value of 0 corresponds to the monoisotopic peak.

MS scans

The msScans table contains information about the full scans, which can be used to verify data quality and generated useful statistics about the interaction between the samples and LC.

Name	Separator	Description
Raw file		The name of the RAW-file the mass spectral data originates from.
Scan number		The scan number (defined in the raw-file) at which the full scan was made.
Scan index		The consecutive index of this full scan.
Retention time		The retention time at which the full scan was made.
Cycle time		The total time (full scan including the tandem MS scans) this full scan has taken up.
Ion injection time		The total injection time that was required to capture the specified amount of ions. This value is limited by a maximum, which can be used to determine whether the time has maxed out (indicative of a bad acquisition).
Base peak intensity		The intensity of the most intense ion in the spectrum.
Total ion current		The total intensity acquired in the full scan.
MS/MS count		The number of tandem MS scans that were made based on this full scan (e.g. a top 10 method selects the top 10 most intense ions in the scan and fragments those).
Mass calibration		The applied mass correction in Th to the full scan.
Experiment		
Peak length		The average time between the start and the end of the peaks detected in the full scan.
Isotope pattern length		The average time between the start and the end of the isotope patterns detected in the full scan.
Multiplet length		The average time between the start and the end of the isotope patterns of the labeling multiplets detected in the full scan.
Peaks / s		The average number of peaks detected per second of chromatography.
Single peaks / s		The average number of single peaks detected per second of chromatography.
Isotope patterns / s		The average number of isotope patterns detected per second of chromatography.
Single isotope patterns / s		The average number of single isotope patterns detected per of second chromatography.
Multiplets / s		The average number of labeling multiplets detected per of second chromatography.
Identified multiplets / s		The percentage of labeling multiplets actually identified.
Multiplet identification rate [%]		The percentage of the detected labeling multiplets that were identified.
MS/MS / s		The average number of MS/MS events per second of chromatography.
Identified MS/MS / s		The average number of identified MS/MS events per second of chromatography.
MS/MS identification rate [%]		The percentage of tandem MS scans that were identified.
Intens Comp Factor		Taken from the Thermo RAW file.
CTCD Comp		Taken from the Thermo RAW file.
RawOvFtT		For Thermo Fisher only. TIC estimation done with the orbitrap cell.
AGC Fill		Taken from the Thermo RAW file.

MZ range

Name	Separator	Description
Raw file		The name of the RAW-file the mass spectral data was derived from.
m/z		The mass-over-charge value.
Peaks / Da		The average number of peaks detected per Dalton.
Single peaks / Da		The average number of single peaks detected per Dalton.
Isotope patterns / Da		The average number of isotope patterns detected per Dalton.
Single isotope patterns / Da		The average number of single isotope patterns detected per Dalton.
SILAC pairs / Da		The average number of SILAC pairs detected per Dalton.
Identified SILAC pairs / Da		The percentage of SILAC pairs actually identified.
SILAC identification rate [%]		The percentage of the detected SILAC pairs that were identified.
MS/MS / Da		The average number of MS/MS events per Dalton.
Identified MS/MS / Da		The average number of identified MS/MS events per Dalton.
Identification rate [%]		The percentage of tandem MS scans that were identified.

MS/MS scans

Name	Separator	Description
Raw file		Name of the RAW file the spectral MS/MS data was extracted from.
Scan number		RAW file derived scan number for the MS/MS spectrum.
Retention time		Time point along the elution profile at which the MS/MS data was recorded.
Ion injection time		The ion inject time for the MS/MS scan. This can be used to determine if this time equals to the maximum ion inject time, general indicative of a lower quality spectrum.
Total ion current		The total ion current of the MS/MS scan. For Thermo data this value is calculated by summing all the intensity values found in the mass spectral data, which is different from the Xcalibur reported TIC (Xcalibur TIC is about 25% of the value reported here).
Collision energy		The collision energy used for the fragmentation that resulted in this MS/MS scan.
Summations		For time of flight instruments only.
Base peak intensity		The intensity of the most intense ion in the spectrum.
Elapsed time		The time the MS/MS scan took to complete.
Identified		When marked with '+' this particular MS/MS scan was identified as a peptide; when marked with '-' no identification was made.
MS/MS IDs		Unique identifier linking this identification to the MS/MS scans.
Sequence		The identified AA sequence of the peptide.
Length		The length of the sequence stored in the column “Sequence”.
Filtered peaks		Number of peaks after the 'top X per 100 Da' filtering.
m/z		Recalibrated m/z of the precursor ion.
Mass		Charge corrected mass of the precursor ion.
Charge		Charge state of the precursor ion.
Type		The type of precursor ion as identified by MaxQuant. ISO – isotopic cluster.<br>PEAK – single peak.<br>MULTI – labeling cluster.
Fragmentation		The type of fragmentation used to create the MS/MS spectrum.<br>CID – Collision Induced Dissociation.<br>HCD – High energy Collision induced Dissociation.<br>ETD – Electron Transfer Dissociation.
Mass analyzer		The mass analyzer used to record the MS/MS spectrum. ITMS – Ion trap.<br>FTMS – Fourier transform ICR or orbitrap cell.<br>TOF – Time of flight.
Parent intensity fraction		The percentage the parent ion intensity makes up of the total intensity in the selection window.
Fraction of total spectrum		The percentage the parent ion intensity makes up of the total intensity of the whole MS spectrum.
Base peak fraction		The percentage the parent ion intensity in comparison to the highest peak in he MS spectrum.
Precursor full scan number		The full scan number where the precursor ion was selected for fragmentation.
Precursor intensity		The intensity of the precursor ion at the scannumber it was selected.
Precursor apex fraction		The fraction the intensity of the precursor ion makes up of the peak (apex) intensity.
Precursor apex offset		How many full scans the precursor ion is offset from the peak (apex) position.
Precursor apex offset time		How much time the precursor ion is offset from the peak (apex) position.
Scan event number		This number indicates which MS/MS scan this one is in the consecutive order of the MS/MS scans that are acquired after an MS scan.
Modifications		Post-translational modifications contained within the sequence. When no modifications exist, this is set to ‘unmodified’.<br><br>Note: This column only set when this MS/MS spectrum has been identified.
Modified sequence		Sequence representation of the peptide including location(s) of modified AAs.<br><br>Note: This column only set when this MS/MS spectrum has been identified.

Proteins		Identifiers of proteins this peptide is associated with.<br><br>Note: This column only set when this MS/MS spectrum has been identified.
Score		The score of the identification (higher is better).<br><br>Note: This column only set when this MS/MS spectrum has been identified.
Experiment		
Intens Comp Factor		Taken from the Thermo RAW file.
CTCD Comp		Taken from the Thermo RAW file.
RawOvFtT		For Thermo Fisher only. TIC estimation done with the orbitrap cell.
AGC Fill		Taken from the Thermo RAW file.
Scan index		Consecutive index of the MS/MS spectrum.
MS scan index		Consecutive index of the MS spectrum prior to this MS/MS spectrum.
MS scan number		Scan number of the MS spectrum prior to this MS/MS spectrum.

MS/MS

Name	Separator	Description
Raw file		The name of the RAW file the mass spectral data was read from.
Scan number		The RAW-file derived scan number of the MS/MS spectrum.
Scan index		The consecutive index of the MS/MS spectrum.
Sequence		The identified AA sequence of the peptide.
Length		The length of the sequence stored in the column "Sequence".
Missed cleavages		Number of missed enzymatic cleavages.
Modifications		Post-translational modifications contained within the identified peptide sequence.
Modified sequence		Sequence representation including the post-translational modifications (abbreviation of the modification in brackets before the modified AA). The sequence is always surrounded by underscore characters ('_').
Deamidation (NQ) Probabilities		Sequence representation of the peptide including PTM positioning probabilities ([0..1], where 1 is best match) for 'Deamidation (NQ)'.
Hydroxyproline Probabilities		Sequence representation of the peptide including PTM positioning probabilities ([0..1], where 1 is best match) for 'Hydroxyproline'.
Oxidation (M) Probabilities		Sequence representation of the peptide including PTM positioning probabilities ([0..1], where 1 is best match) for 'Oxidation (M)'.
Propionamide K Probabilities		Sequence representation of the peptide including PTM positioning probabilities ([0..1], where 1 is best match) for 'Propionamide K'.
Deamidation (NQ) Score Diffs		
Hydroxyproline Score Diffs		
Oxidation (M) Score Diffs		
Propionamide K Score Diffs		
Acetyl (Protein N-term)		
Deamidation (NQ)		
Gln->pyro-Glu		
Glu->pyro-Glu		
Hydroxyproline		
Oxidation (M)		
Propionamide K		
Propionamide N-term		
Proteins		The identifiers of the proteins the identified peptide is associated with.
Charge		The charge state of the precursor ion.
Fragmentation		The type of fragmentation used to create the MS/MS spectrum.<br>CID – Collision Induced Dissociation.<br>HCD – High energy Collision induced Dissociation.<br>ETD – Electron Transfer Dissociation.
Mass analyzer		The mass analyzer used to record the MS/MS spectrum. ITMS – Ion trap.<br>FTMS – Fourier transform ICR or orbitrap cell.<br>TOF – Time of flight.
Type		The type of precursor ion as identified by MaxQuant. ISO – isotopic cluster.<br>PEAK – single peak.<br>MULTI – labeling cluster.
Scan event number		
Isotope index		
m/z		The mass-over-charge of the precursor ion.
Mass		The charge corrected mass of the precursor ion.
Mass error [ppm]		Mass error of the recalibrated mass-over-charge value of the precursor ion in comparison to the predicted monoisotopic mass of the identified peptide sequence expressed in parts per million.
Mass error [Da]		Mass error of the recalibrated mass-over-charge value of the precursor ion in comparison to the predicted monoisotopic mass of the identified peptide sequence expressed in atomic mass units.
Simple mass error [ppm]		
Retention time		The uncalibrated retention time in minutes where the MS/MS spectrum has been acquired.

PEP		Posterior Error Probability of the identification. This value essentially operates as a p-value, where smaller is more significant.
Score		Andromeda score for the best associated MS/MS spectrum.
Delta score		Score difference to the second best identified peptide with a different amino acid sequence.
Score diff		Score difference to the second best positioning of modifications identified peptide with the same amino acid sequence.
Localization prob		
Combinatorics		Number of possible distributions of the modifications over the peptide sequence.
PIF		Short for Parent Ion Fraction; indicates the fraction the target peak makes up of the total intensity in the inclusion window.
Fraction of total spectrum		The percentage the parent ion intensity makes up of the total intensity of the whole spectrum.
Base peak fraction		The percentage the parent ion intensity in comparison to the highest peak in he MS spectrum.
Precursor Full ScanNumber		The full scannumber where the precursor ion was selected for fragmentation.
Precursor Intensity		The intensity of the precursor ion at the scannumber it was selected.
Precursor Apex Fraction		The fraction the intensity of the precursor ion makes up of the peak (apex) intensity.
Precursor Apex Offset		How many full scans the precursor ion is offset from the peak (apex) position.
Precursor Apex Offset Time		How much time the precursor ion is offset from the peak (apex) position.
Matches		The species of the peaks in the fragmentation spectrum after TopN filtering.
Intensities		The intensities of the peaks in the fragmentation spectrum after TopN filtering.
Mass Deviations [Da]		The mass deviation of each peak in the fragmentation spectrum in absolute mass units.
Mass Deviations [ppm]		The mass deviation of each peak in the fragmentation spectrum in parts per million.
Masses		The masses-over-charge of the peaks in the fragmentation spectrum.
Number of Matches		The number of peaks matching to the predicted fragmentation spectrum.
Intensity coverage		The fraction of intensity in the MS/MS spectrum that is annotated.
Peak coverage		The fraction of peaks in the MS/MS spectrum that are annotated.
Neutral loss level		How many neutral losses were applied to each fragment in the Andromeda scoring.
ETD identification type		For ETD spectra several different combinations of ion series are scored. Here the highest scoring combination is indicated
Reverse		When marked with '+', this particular peptide was found to be part of a protein derived from the reversed part of the decoy database. These should be removed for further data analysis.
All scores		
All sequences		
All modified sequences		
id		A unique (consecutive) identifier for each row in the msms table, which is used to cross-link the information in this file with the information stored in the other files.
Protein group IDs		The identifier of the protein-group this redundant peptide sequence is associated with, which can be used to look up the extended protein information in the file 'proteinGroups.txt'.<br>As a single peptide can be linked to multiple proteins (e.g. in the case of razor-proteins), multiple id's can be stored here separated by a semicolon.<br>As a protein can be identified by multiple peptides, the same id can be found in different rows.
Peptide ID		The identifier of the non-redundant peptide sequence.
Mod. peptide ID		Identifier of the associated modification summary stored in the file 'modificationSpecificPeptides.txt'.
Evidence ID		Identifier of the associated evidence stored in the file 'evidence.txt'.
Deamidation (NQ) site IDs		Identifier of the oxidation summary stored in the file 'Deamidation (NQ)Sites.txt'.
Hydroxyproline site IDs		Identifier of the oxidation summary stored in the file 'HydroxyprolineSites.txt'.
Oxidation (M) site IDs		Identifier of the oxidation summary stored in the file 'Oxidation (M)Sites.txt'.
Propionamide K site IDs		Identifier of the oxidation summary stored in the file 'Propionamide KSites.txt'.
